# Catalytic Asymmetric Ionic Hydrogenation of α‑Alkyl Styrenes

**DOI:** 10.1021/jacs.5c11514

**Published:** 2025-08-20

**Authors:** Wencke Leinung, Nobuya Tsuji, Michael Merher, Markus Leutzsch, Ravindra K. Raut, Benjamin List

**Affiliations:** † 28314 Max-Planck-Institut für Kohlenforschung, 45470 Mülheim an der Ruhr, Germany; ‡ Institute for Chemical Reaction Design and Discovery, 12810 Hokkaido University, Sapporo 001-0021, Japan

## Abstract

Over the past two decades, chemists have made significant advances in the field of catalytic asymmetric transfer hydrogenation of various unsaturated compounds with biomimetic hydrogen donors. The reduction of carbon–carbon double bonds, however, has been limited to activated substrate classes, such as enals, enones, nitroolefins, or α-(2-hydroxyaryl) styrenes. Here we report a highly enantioselective Brønsted acid-catalyzed ionic hydrogenation of α-alkyl styrenes using a hydrosilane in combination with a protic additive. Mechanistic and computational investigations support a pathway proceeding through a carbocation intermediate and a transient silylated catalyst species, with catalyst turnover dependent on the presence of a protic additive. Moreover, the exemplary stereoconvergent reduction of a trisubstituted alkene highlights the potential of organocatalytic approaches over traditional metal-catalyzed hydrogenations.

## Graphical abstract

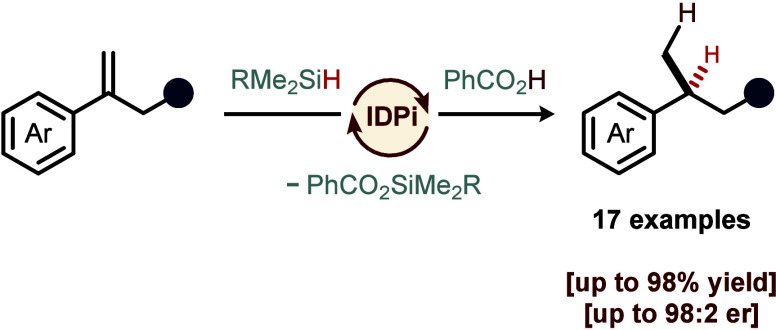

As one of the most pivotal transformations in chemical synthesis, enantioselective alkene hydrogenation finds broad applications in the synthesis of pharmaceuticals, agrochemicals, and fragrances.^1–8^ In the realm of asymmetric organocatalysis, numerous biomimetic transfer hydrogenations have emerged throughout the last two decades.^9–13^ For the reduction of both electron-poor (enals,^14–22^ enones,^23,24^ and nitroolefins^25–30^ ) and electron-rich^31^ alkenes, various organocatalytic concepts have been employed (Figure 1A). However, all methods require substrates bearing an activating group, and reports on stereoselective, metal-free transfer hydrogenations of electronically or structurally unbiased alkenes remain scarce. Typically proceeding under Brønsted acidic conditions, the reported examples involve the generation of a carbocation, necessitating distinct strategies for stereocontrol (Figure 1B).^32^ For instance, Gagosz and Chiba reported a diastereoselective intramolecular 1,5-hydride transfer of styrenes with an α-stereogenic center (substrate control).^33^ Another stereoselective approach involves the application of chiral hydride donors to trap the intermediate carbocation and was first showcased with a chiral hydrosilane in 1978 (reagent control).^34^ More recently, Qu and Oestreich applied chiral cyclohexadiene reagents for the enantioselective reduction of α-alkyl styrenes, mostly featuring a *p*-methoxy group.^35^ Achieving catalytic enantiofacial control over (benzylic) carbocations, formed via olefin protonation, has previously been explored with various C, N, and O nucleophiles.^36–42^ To the best of our knowledge, however, the asymmetric catalytic reaction of electronically or structurally unbiased alkenes with H nucleophiles remains elusive to this day. Here we report an enantioselective Brønsted acid-catalyzed ionic hydrogenation of simple α-alkyl styrenes using a hydrosilane in the presence of a proton source (Figure 1C).

**Figure 1:**
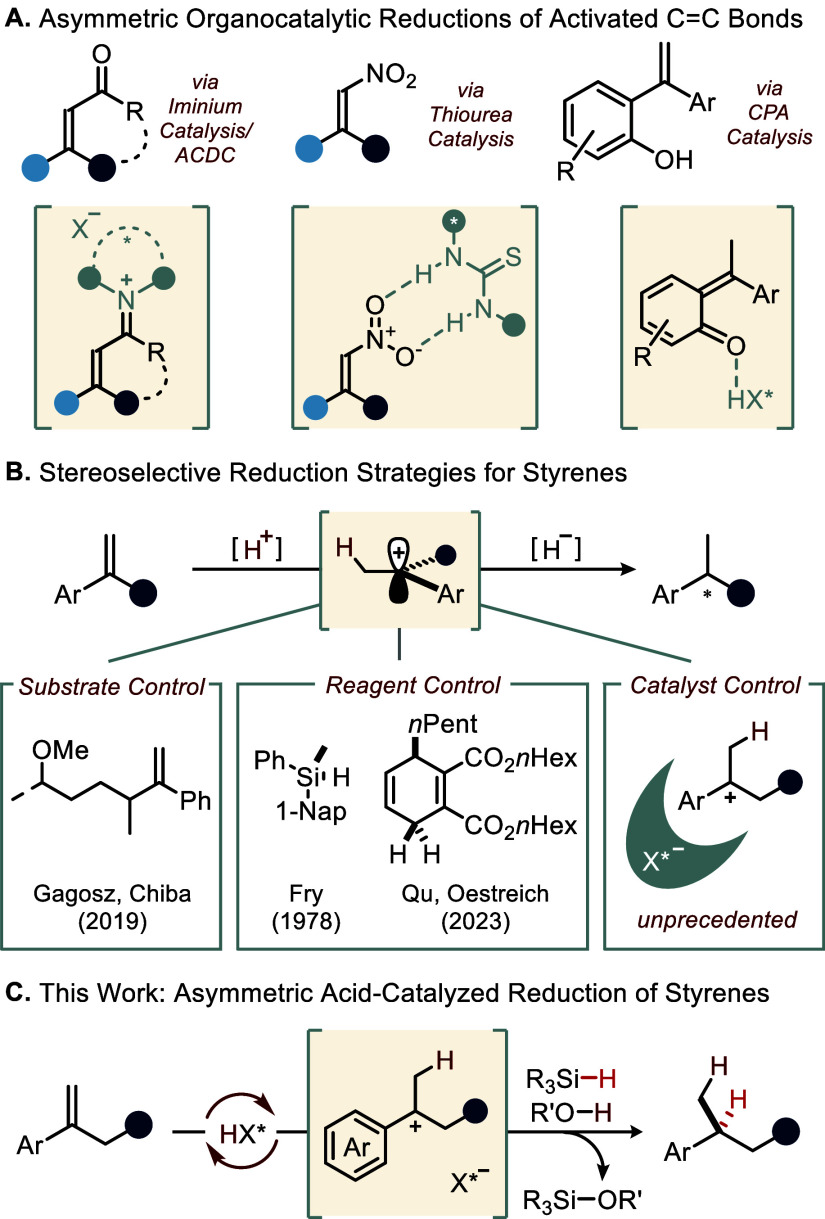
(A) Common substrates for asymmetric organocatalytic transfer hydrogenations. ACDC = asymmetric counteranion-directed catalysis. (B) Metal-free stereoselective strategies for the reduction of styrenes. (C) Our approach: IDPi-catalyzed ionic hydrogenation of α-alkyl styrenes.

We commenced our study with hex-1-en-2-ylbenzene (**1a**) as our model substrate using dimethylphenylsilane as a reductant in combination with benzoic acid as a stoichiometric proton source (Table 1). When using moderately acidic catalysts (p*K*_a_ ≥ 8.4 in MeCN),^43,44^ such as chiral phosphoric acids (CPAs), imidodiphosphates (IDPs), or disulfonimides (DSIs), styrene **1a** was solely converted to double-bond isomer **3a** (see the Supporting Information (SI) for details). With more acidic iminodiphosphorimidate (IDPi) catalysts (mostly 6.9 ≥ p*K*_a_ ≥ 2.0 in MeCN),^43,45^ formation of the desired hydrogenation product **2a** was observed. For instance, heteroaromatic IDPi **5a** furnished alkyl benzene **2a** in a moderate yield of 37% with low enantioselectivity (entry 1). A promising enantiomeric ratio of product **2a** (74:26 er) was obtained when the IDPi triflyl core was changed to a perfluorophenylsulfonyl group, albeit in a reduced yield of 19% (entry 2). Substitution of the 3,3′-positions with electron-rich benzo­[*b*]­thieno­[3,2-*d*]­thiophen-2-yl in **6b** not only restored the reactivity but also improved the enantioselectivity to 82.5:17.5 (entry 3).^45^ Remarkably, the installation of electron-withdrawing CF_3_ groups in catalyst **7b** afforded hydrogenation product **2a** in nearly quantitative yield with good enantioselectivity (entry 4). Finally, reducing the reaction temperature to −20 °C and using ethyldimethylsilane as an alternative reducing reagent led to excellent levels of enantiocontrol (entries 5 and 6). However, a decrease in reactivity, along with the formation of dimeric side product **4a**, was observed—both of which were mitigated by simply conducting the reaction neat in 10 equiv of silane (entry 7).

**Table 1:**
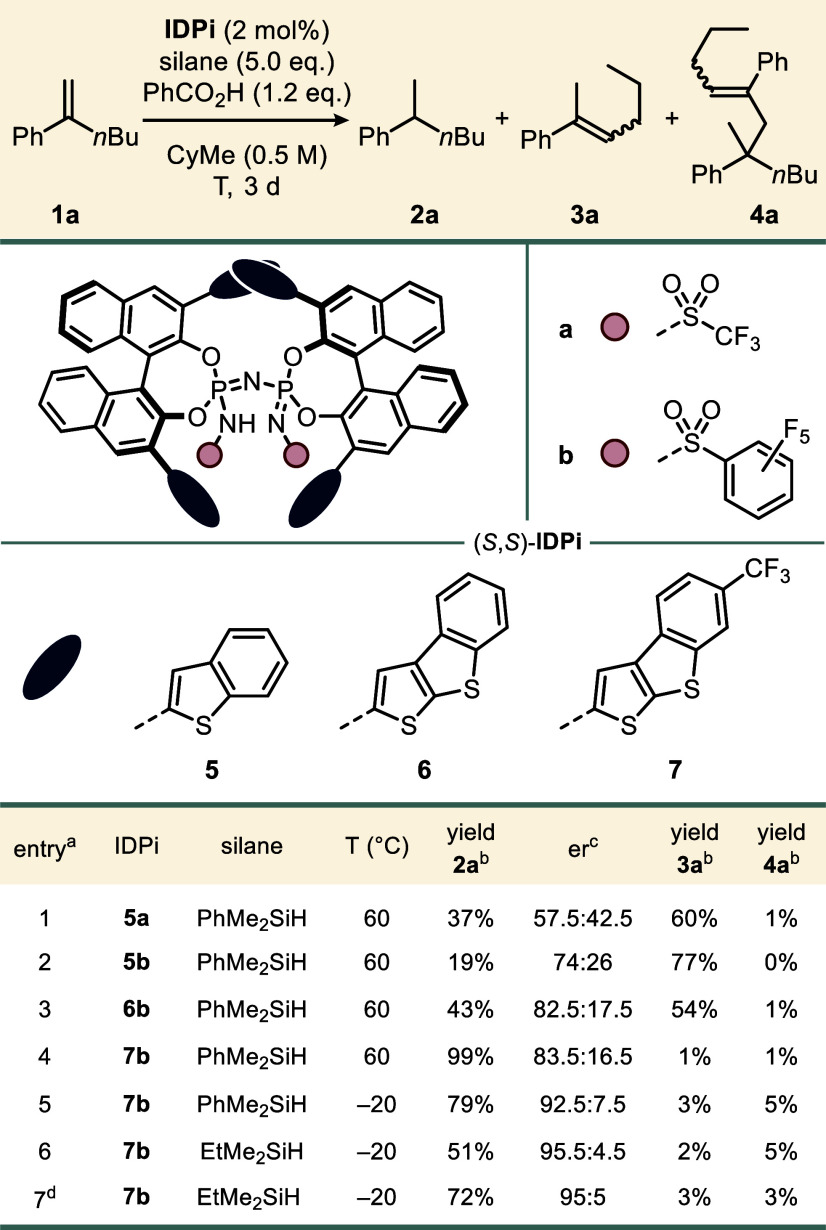
Reaction Development

a Reactions were performed on a 0.025 mmol scale.

b Determined via ^1^H NMR analysis using mesitylene as the internal standard.

c Determined via GC.

d Neat in 10 equiv of EtMe_2_SiH.

With the optimized reaction conditions in hand, we explored the scope of our ionic hydrogenation (Figure 2). Due to volatility of some products, both crude NMR and isolated yields were determined. After obtaining product **2a** in good yield with 95:5 er, we turned our attention to modifications of the aromatic substituent. Weakly electron-donating *p*-methyl substitution led to increased reactivity and slightly reduced selectivity. However, at lower temperatures, product **2b** was obtained in good yield with high optical purity. *m*-Alkyl and dimethyl substituents (**2b**–**2e**) were tolerated well, furnishing the products in very good yields with excellent enantiomeric ratios. Halogenated α-alkyl styrenes **1f** and **1g** featuring a fluoro or bromo group and electron-poor *m*-methoxylated substrate **1h** were efficiently reduced with very high enantioselectivity using the more reactive dimethylphenylsilane. When the ionic hydrogenation of styrene **1h** was scaled up to 5 mmol, the catalyst loading could be reduced to 1 mol % without comprising reactivity or selectivity of the reaction. Upon demethylation and reaction with 4-bromophenyl isocyanate, product **2h** was converted to carbamate **2i**, which led to the unambiguous identification of the absolute configuration by X-ray crystallography. Electron-rich 2-naphthyl- and 5-benzofuranyl-substituted products **2j** and **2k** were furnished in very good yields with 98:2 and 97:3 er, respectively.

**Figure 2:**
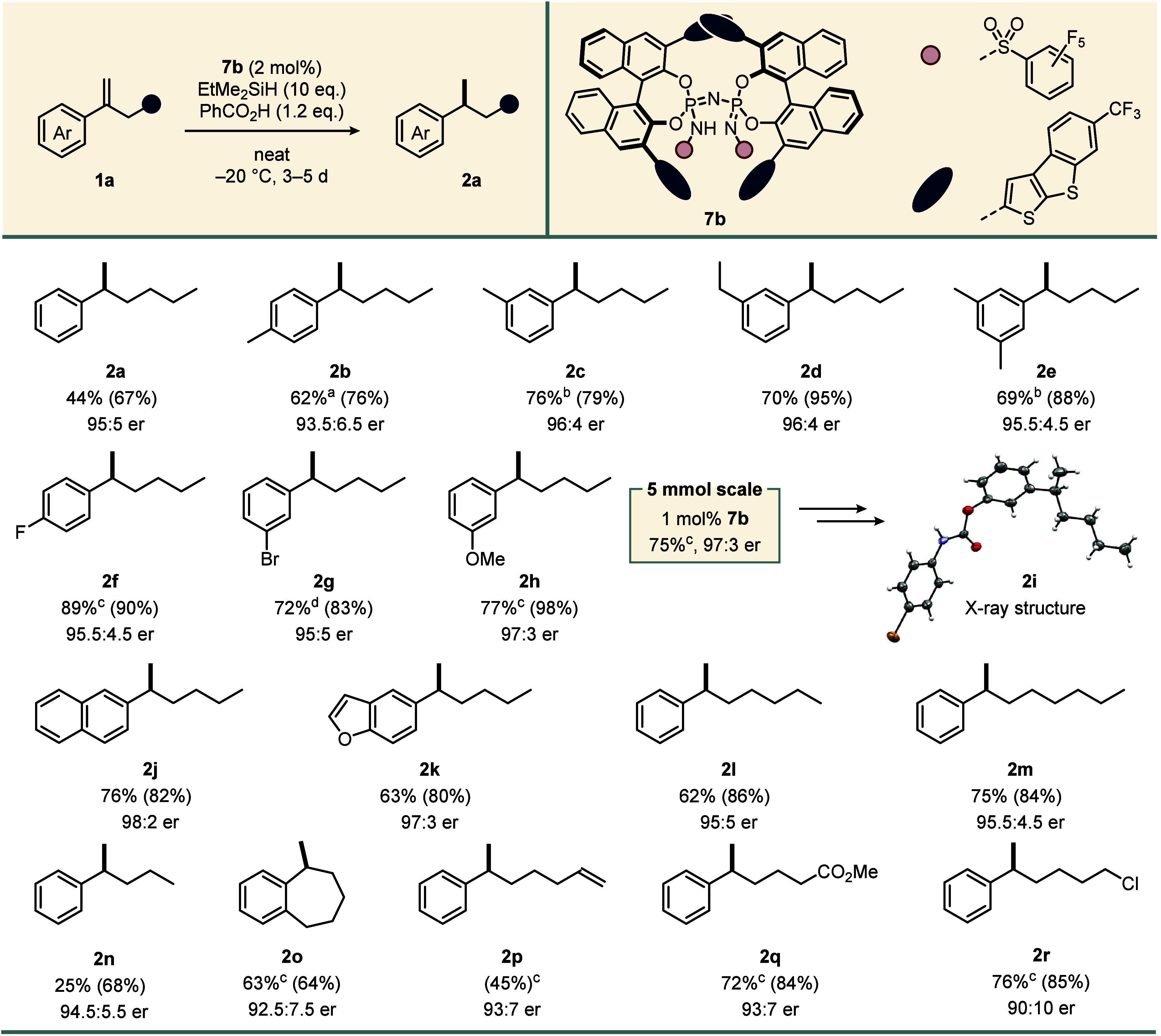
Scope of the ionic hydrogenation. All reactions were conducted on a 0.2 mmol scale. Yields are reported as isolated yields after column chromatography. ^1^H NMR yields, reported in parentheses, were determined using CH_2_Br_2_ as the internal standard. Notes: ^a^The reaction was performed at −50 °C. ^b^Using EtMe_2_SiH (5.0 equiv) in CyMe (0.5 M). ^c^The reaction was performed using PhMe_2_SiH (5.0 equiv) in CyMe (0.5 M). ^d^The reaction was performed at 10 °C using PhMe_2_SiH (10 equiv) under neat reaction conditions. See the SI for detailed reaction conditions.

Next, we focused on exploring modifications of the aliphatic α-chains of our substrates. Notably, styrenes with both prolonged and shortened alkyl groups (**1l**–**1n**) were well-tolerated with only minor effects on selectivity. Furthermore, although *o*-methyl substitution had previously led to a loss of reactivity (see the SI for details), cyclized styrene **1o** proved to be reactive within our established system. Finally, moieties susceptible to reducing conditions were introduced to probe the functional group tolerance of our ionic hydrogenation. Diene **1p** underwent selective reduction at its benzylic trisubstituted double bond, though the yield of **2p** was diminished by a cyclization side product (see the SI for details). Remarkably, subjecting methyl ester-containing styrene **1q** to our established protocol furnished **2q** in very high yield with good enantioselectivity, with the ester functionality remaining intact. As alkyl halides are known to undergo reduction in the presence of acids and silanes,^46^ we were pleased to find that chlorobutyl-substituted styrene **1r** was a suitable substrate for our methodology.

Mechanistically, we envisioned our reduction to proceed via a benzylic carbocation intermediate, formed upon styrene protonation by IDPi **7b**, followed by hydride transfer from the silane (Figure 3D). In that case, the same product enantiomer can be expected, irrespective of the employed styrene isomer **1** or (*E*)/(*Z*)-**3**. Indeed, when **1i** and (*E*)- and (*Z*)-**3i** were subjected to our reducing conditions at different temperatures, the same major enantiomer was obtained in all cases (Figure 3A). This feature addresses one of the major limitations of asymmetric metal-catalyzed alkene hydrogenations. The presence of both geometrical isomers of trisubstituted olefins typically leads to low stereoselectivities, as coordination to the metal catalyst is governed by the less substituted carbon atom of the alkene.^47^ Despite the challenging reactivity of trisubstituted alkenes in our organocatalytic approach (see the SI for details), the demonstrated stereoconvergent ionic hydrogenation of (*E*)- and (*Z*)-**3i** offers a promising foundation for future developments.

**Figure 3:**
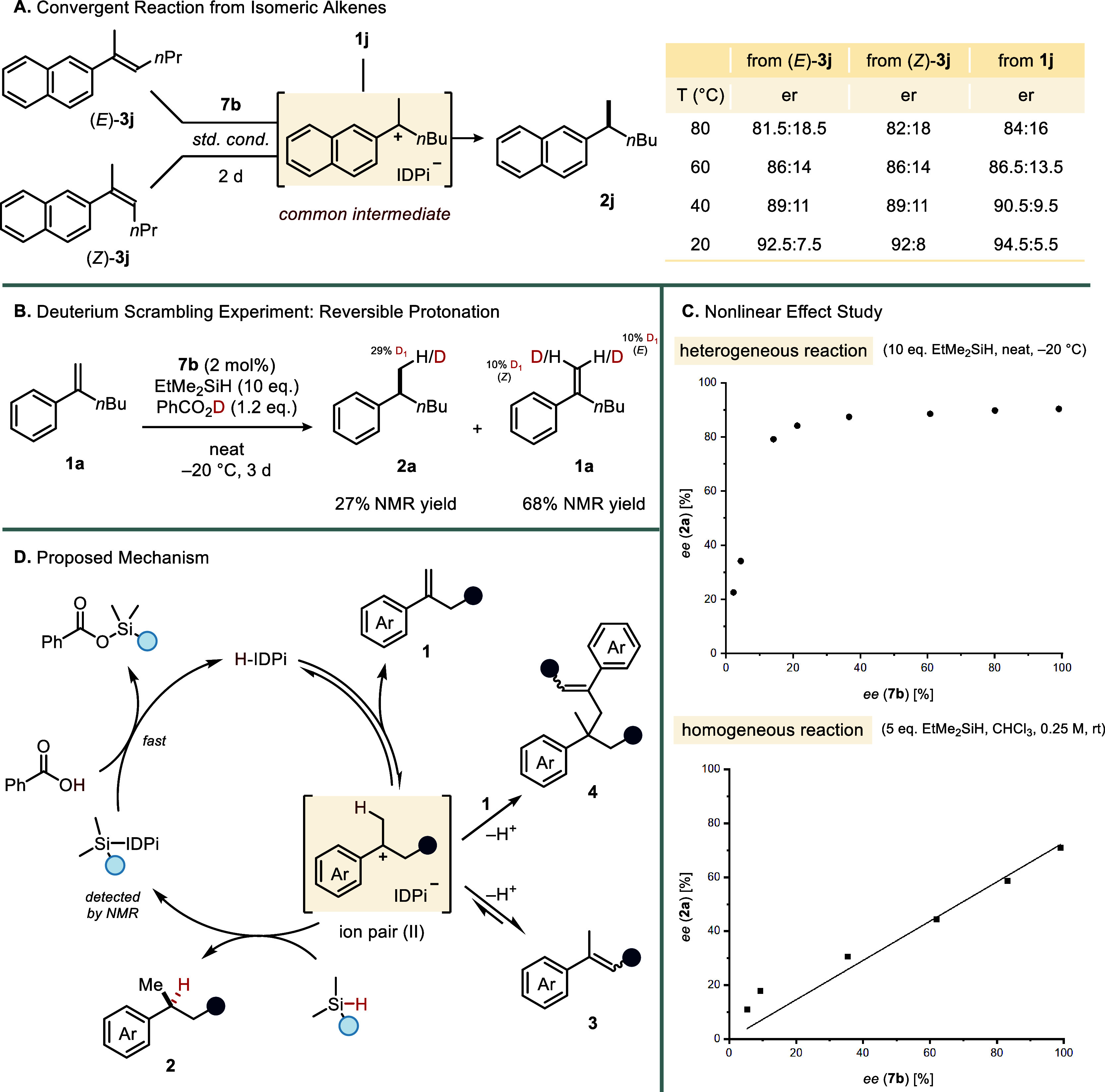
Mechanistic considerations. (A) Probe for a common benzylic carbocation intermediate. (B) Deuterium scrambling experiment with PhCO_2_D. (C) Nonlinear effect studies. (D) Proposed mechanism.

Prompted by evidence of the benzylic carbocation intermediate, we next examined whether the protonation step is reversible. When we conducted our experiment under the standard conditions with benzoic acid-*d*_1_, we observed partial deuteration of the starting material, which aligns with a reversible protonation equilibrium (Figure 3B). To investigate whether the proton source is possibly involved in the enantiodetermining step, the influence of an enantiopure chiral proton source was examined. In control experiments employing chiral α-methoxyphenylacetic acid or 2-phenylpropionic acid, no matched/mismatched scenario relative to **7b** was observed, and the same enantiomeric ratio of **2a** was obtained, indicating that the proton source does not participate in stereocontrol (see the SI for further details).

According to our proposed mechanism, hydride transfer from the silane to the ion pair (**II**) gives rise to product **2a** and the silylated IDPi catalyst. In the absence of a proton source, accumulation of this silylated species was confirmed by NMR spectroscopy. Probing the catalyst regeneration step by independent preparation of silylated IDPi **7b** and subsequent addition of benzoic acid revealed a fast and spontaneous protodesilylation regenerating **7b** along with the formation of silyl benzoate (see the SI for details).

Remarkably, a strong positive nonlinear effect ((+)-NLE) was observed for the catalytic reduction when scalemic mixtures of IDPi **7b** were used (Figure 3C). In addition, the reactivity diminished with decreasing enantiomeric purity of **7b**. This effect can be attributed to differences in solubility of homo- and heterochiral IDPi complexes, as evidenced by distinct enantiomeric ratios between the solid and liquid phases of the reaction. Homochiral complexes exhibited greater solubility, leading to enrichment of the liquid phase with optically pure **7b**, while the solid phase retained a lower enantiomeric ratio (see the SI for further details). In contrast to the heterogeneous conditions, a nearly linear correlation was observed under homogeneous reaction conditions, further substantiating our findings. Similar phenomena have previously been observed for amino acids^48,49^ and CPAs.^50^

Finally, we conducted computational studies involving alkene **1a**, EtMe_2_SiH, and IDPi **7b** to gain further insight into the reaction mechanism and the origin of the enantioselectivity. The resulting free energy diagram for the protonation and hydride transfer steps aligned well with the experimental observations (Figure 4A). In the pre-transition-state complex **I**, alkene **1a** is oriented for protonation by IDPi **7b**. Subsequent proton transfer via transition state **TS1** furnishes ion pair **II** featuring a carbocation intermediate. The endergonic nature of this step supports the reversibility of protonation, consistent with experimental data. In the presence of silane (**III**), the enantiodetermining hydride transfer occurs via **TS2** or **TS2′** to furnish complex **IV** or **IV′**, which eventually releases the product (*S*)-**2a** or (*R*)-**2a**. The calculated enantioselectivity (1.7 kcal/mol; Figure 4B) is in good agreement with the experimental value (1.6 kcal/mol).

**Figure 4:**
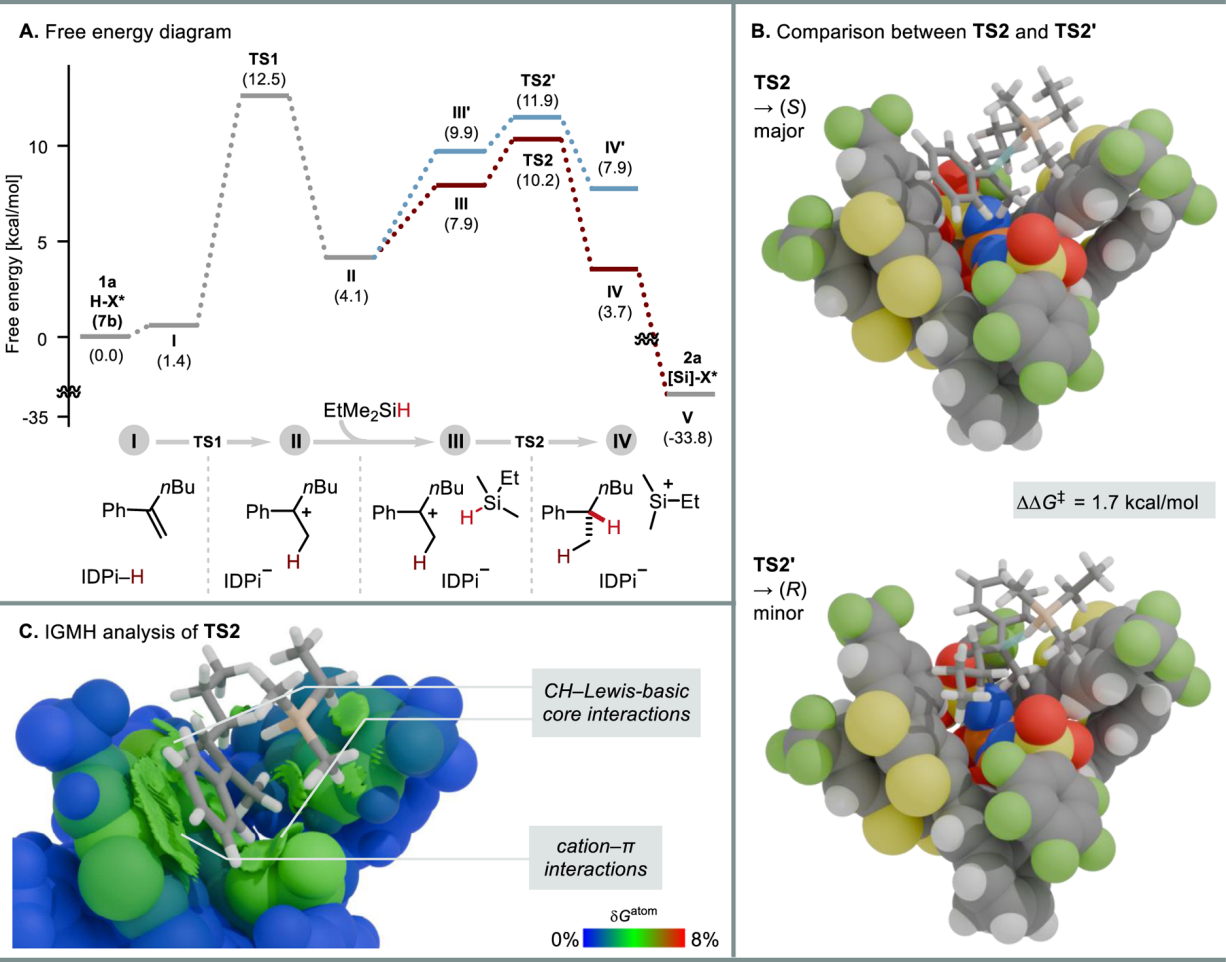
Computational details. (A) Energy diagram leading to the product **2a** at the SMD­(methylcyclohexane)-ωB97X-V/def2-TZVPP//r^2^SCAN-3c level of theory^52–54^ (253 K). **TS2** represents the transition state leading to the major enantiomer (*S*)-**2a**, while **TS2′** leads to the minor enantiomer (*R*)-**2a**. (B) Visualization of **TS2** and **TS2′**. (C) Visualization of noncovalent interactions between substrates and the catalyst in **TS2** using IGMH analysis. The sign­(λ_2_)­ρ-colored isosurface corresponds to δ*g* ^inter^ = 0.004 au, with a color scale range from −0.05 to 0.05 au The atoms of the anion are colored by δ*G* ^atom^ (%) using a scale from 0 to 8% to highlight their relative contributions.

Next, we turned our attention to the origin of the enantioselectivity. Distortion–interaction analysis^51^ suggests that the key factor governing the enantioselectivity is the interaction between the substrates and the chiral counteranion (Table S24). To visualize these noncovalent interactions, the independent gradient model based on Hirshfeld partition (IGMH)^55^ implemented in the Multiwfn program^56,57^ was used (Figure 4C). The transition state structure was divided into two fragments: the catalyst anion and the substrate–silane unit. Solid surfaces represent the interactions between these two fragments, illustrating multiple noncovalent interactions. Among these, interactions between the polarized C–H bonds of the substrate and the Lewis basic residues of the catalyst core become apparent. A particularly prominent interaction in **TS2** is a cation−π interaction between the heteroarene moiety of the IDPi anion and the phenyl ring of the cationic substrate—an interaction that is absent in **TS2′**. Notably, the geometries of the counteranion remain nearly identical in the competing transition states, presumably due to multiple noncovalent bonding interactions that help stabilize the conformations^45^ (see the SI for more details).

In summary, we have developed the first chemo- and enantioselective organocatalytic reduction of α-alkyl styrenes via ionic hydrogenation using hydrosilanes and benzoic acid. Confined and highly acidic IDPi **7b** was key to achieving reaction control and overcoming competing isomerization and dimerization pathways. Mechanistic investigations align with the generation of a benzylic carbocation intermediate, which is stabilized through various noncovalent interactions with the IDPi anion. The successful enantioselective reduction of trisubstituted alkene **3i**—independent of its geometrical purity—underscores the advantage of this approach over traditional metal-catalyzed hydrogenations. Efforts to expand the scope toward trisubstituted alkenes are currently underway.

## Supplementary Material



## Notes

Additional experimental details, compound synthesis and characterization, and computational details (PDF)
