# Supplementary material for: Catalytic Asymmetric Ionic Hydrogenation of α‑Alkyl Styrenes
Source: J Am Chem Soc. 2025 Aug 20;147(35):31463–9. doi: 10.1021/jacs.5c11514 (PMC12412155; doi:10.1021/jacs.5c11514)
Supplement: Supplementary file 1 [file ja5c11514_si_001.pdf]

Catalytic Asymmetric Ionic Hydrogenation of α-Alkyl Styrenes

Wencke Leinung[a], Nobuya Tsuji[b], Michael Merher[a], Markus Leutzsch[a], Ravindra K. Raut[b], Benjamin List[a,b]*

[a]Max-Planck-Institut für Kohlenforschung, 45470 Mülheim an der Ruhr, Germany

[b]Institute for Chemical Reaction Design and Discovery, Hokkaido University, Sapporo 001 – 0021, Japan

*Email: list@kofo.mpg.de

Table of Contents

1. General Information ... 3

2. Reaction Optimization ... 4

3. Synthesis of Styrenes ... 16

4. Asymmetric Reduction of Styrenes ... 28

5. Limitations of the method ... 42

6. Scale-Up Experiment and Derivatization for Absolute Configuration Assignment ... 44

7. Synthesis of IDPi Catalysts ... 47

8. Mechanistic Studies ... 59

8.1. Probe for a Common Benzylic Carbocation Intermediate ... 59

8.2. Deuterium Scrambling Experiment ... 61

8.3. Investigating the Influence of a Chiral Proton Source on Stereocontrol ... 63

8.4. Detection of Silylated IDPi Species ... 65

8.5. Experimental Probe of the Catalyst Regeneration Step ... 70

8.6. Non-Linear Effect (NLE) Study ... 74

9. NMR Spectra ... 76

10. GC Traces ... 139

11. Crystallographic Data ... 156

12. Computational Details 161

13. References 220

1. General Information

Unless otherwise stated, all reagents were purchased from commercial suppliers and used without further purification. All solvents used in the reactions were distilled from appropriate drying agents prior to use. Reactions were monitored by thin layer chromatography (TLC) on silica gel pre-coated plastic sheets (0.2 mm, Macherey-Nagel). Visualization was performed by irradiation with UV light at 254 nm and/or phosphomolybdic acid (PMA) or $KMnO_4$ staining. Column chromatography was conducted using Merck silica gel (60 Å, 230–400 mesh, particle size 0.040–0.063 mm) using technical grade solvents. ^{1}H, ^{13}C, ^{19}F, and ^{31}P NMR spectra were recorded on a Bruker Avance III 500 or Bruker NEO 600 MHz (equipped with a BBO CryoProbe) spectrometer in deuterated solvents. ^{1}H chemical shifts (δ) are reported in ppm relative to the protonated solvent resonance employed as the internal standard ($CDCl_3$ δ = 7.26, CD_2Cl_2 δ = 5.32, C_6D_6 δ = 7.16, $(CD_3)_2CO$) δ = 2.05). Data are reported as follows: chemical shift, multiplicity (s = singlet, d = doublet, t = triplet, q = quartet, p = pentet, s = sextet, h = heptet, m = multiplet), coupling constants (Hz) and integration. When data from a mixture of *E*/*Z* isomers were reported, signals of the minor isomer were assigned only if they were unambiguously resolved. ^{13}C chemical shifts are reported in ppm with the solvent resonance as the internal standard ($CDCl_3$ δ = 77.16, CD_2Cl_2 δ = 54.00, C_6D_6 δ = 128.06, $(CD_3)_2CO$ δ = 29.84). High-resolution mass spectra were obtained using a Bruker APEX III FTMS (7 T magnet). Optical rotations were determined with an Autopol IV polarimeter (Rudolph Research Analytical) at 589 nm (sodium D line) and 25 °C. Data are reported as follows: $[\alpha]_D^T$, concentration *c* (g/100 mL) and solvent. Enantiomeric ratios (er) were determined by GC or HPLC analysis using a chiral stationary phase column, indicated in each experiment, by comparing the samples with the corresponding racemic mixtures.

2. Reaction Optimization

Reaction Development: General Procedure 1 (GP1)

A 2 mL GC vial, equipped with a magnetic stirring bar, was charged with the chiral catalyst (2 mol%) and the proton source (0.030 mmol, 1.2 eq.) and then placed under argon. The respective solvent and hydrosilane (0.125 mmol, 5.0 eq.) were added sequentially, followed by the styrene (0.025 mmol, 1.0 eq.). The reaction was stirred at the indicated temperature for three days (for reactions at temperatures below 20 °C, the vial was cooled to –78 °C before substrate addition and then warmed to the respective temperature). The reaction was quenched by addition of pyridine (5 µL) followed by addition of mesitylene (3 µL) as internal standard. An aliquot of the mixture was taken and diluted with C_6D_6 for subsequent ^{1}H NMR analysis. The remaining solution was used for preparative thin layer chromatography (pentane) to purify the chiral product. Chiral GC analysis was performed to give the corresponding enantiomeric ratio.

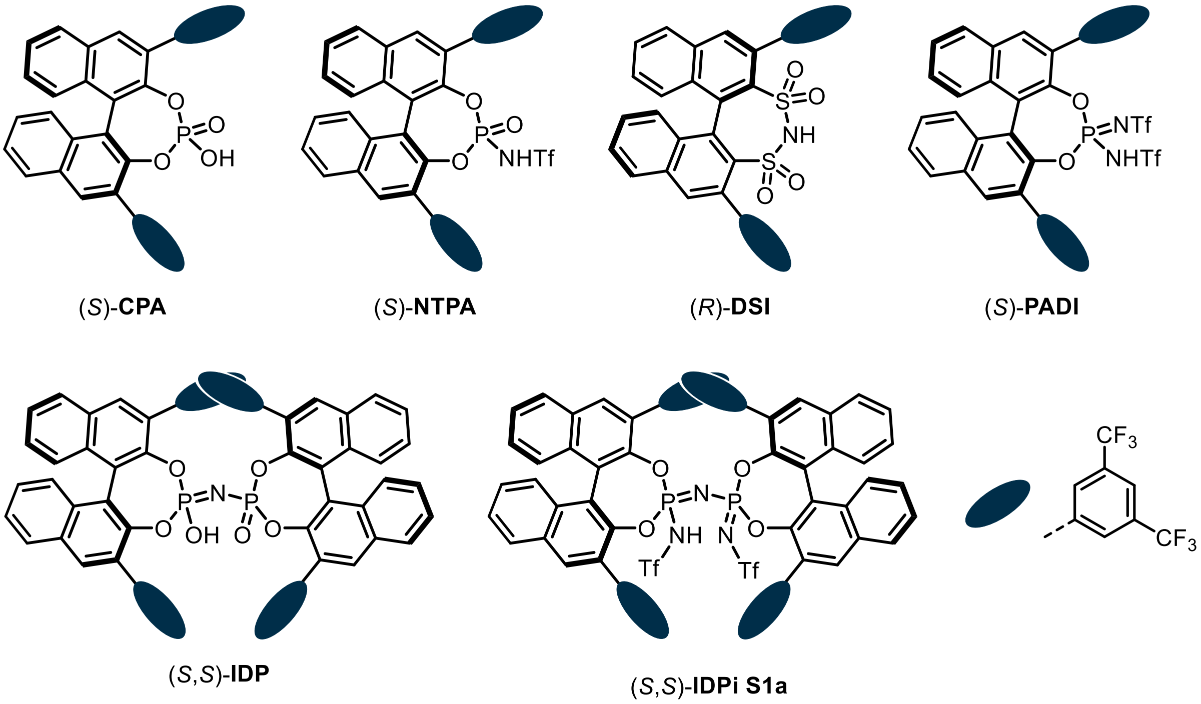

Figure S1: *Chiral Brønsted acids used in the reaction development.*

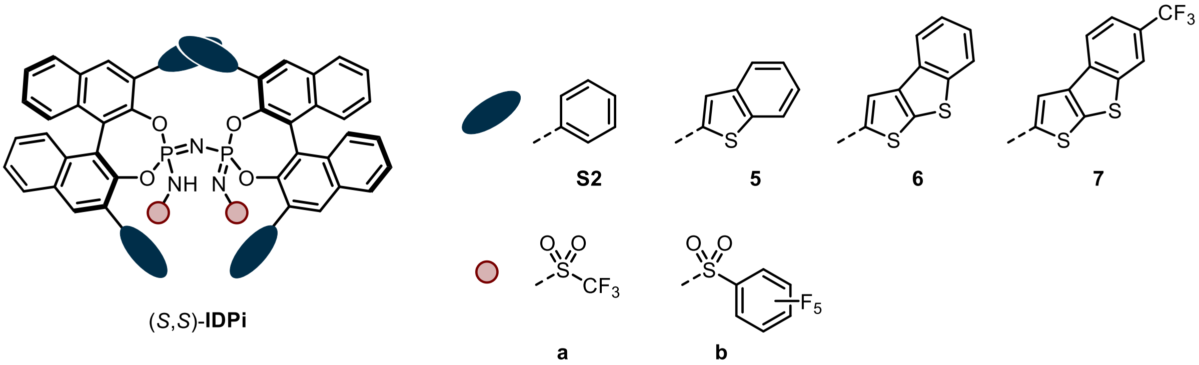

Figure S2: *(S,S)-IDPi catalysts used in the reaction development.*

Table S1: *Optimization of reaction conditions: catalyst, temperature, and silane.*

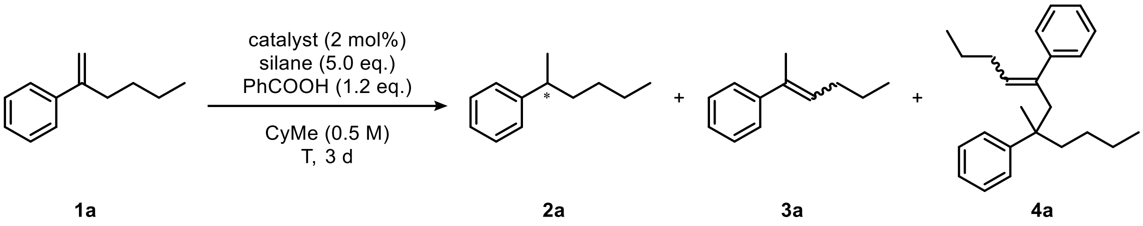

catalyst	temp.	silane	conv.	yield (2a)	er	yield (3a)	yield (4a)
CPA	60 °C	$PhMe_2SiH$	4%	0%	-	4%	0%
NTPA	60 C	$PhMe_2SiH$	95%	traces	-	94%	0%
DSI	60 °C	$PhMe_2SiH$	91%	0%	-	91%	0%
PADI	60 °C	$PhMe_2SiH$	100%	90%	50.5:49.5	0%	0%
IDP	60 °C	$PhMe_2SiH$	7%	0%	-	7%	0%
IDPi S1a	60 °C	$PhMe_2SiH$	99%	74%	44.5:55.5	15%	1%
S2a	60 °C	$PhMe_2SiH$	60%	4%	58:48	55%	0%
5a	60 C	$PhMe_2SiH$	98%	37%	57.5:42.5	60%	1%
5b	60 °C	$PhMe_2SiH$	97%	19%	74:26	77%	0%
6b	60 °C	$PhMe_2SiH$	98%	43%	82.5:17.5	54%	1%
7b	60 °C	$PhMe_2SiH$	>99%	99%	83.5:16.5	1%	1%
7b	60 °C	$PhMe_2SiH$	>99%	99%	83.5:16.5	1%	1%
7b	40 °C	$PhMe_2SiH$	>99%	84%	86.5:13.5	8%	2%

**7b**	20 °C	PhMe$_2$SiH	>99%	79%	89:11	14%	5%
**7b**	0 °C	PhMe$_2$SiH	>99%	82%	91:9	9%	5%
**7b**	–20 °C	PhMe$_2$SiH	98%	79%	92.5:7.5	3%	5%
**7b**	–40 °C	PhMe$_2$SiH	62%	48%	94:6	0%	7%
**7b**	–20 °C	PhMe$_2$SiH	98%	79%	92.5:7.5	3%	5%
**7b**	–20 °C	PhMeSiH$_2$	70%	26%	89.5:10.5	9%	17%
**7b**	–20 °C	PhSiH$_3$	44%	traces	n.d.	12%	17%
**7b**	–20 °C	Ph$_2$MeSiH	85%	43%	88.5:11.5	8%	17%
**7b**	–20 °C	Et$_3$SiH	67%	32%	94:6	5%	15%
**7b**	–20 °C	EtMe$_2$SiH	62%	51%	95.5:4.5	2%	5%
**7b**	EtMe$_2$SiH*	–20 °C	80%	72%	95:5	3%	3%

* 10 eq. silane used under neat reaction conditions.

Table S2: *Evaluation of reaction conditions: H$^+$ source, solvent, concentration.*

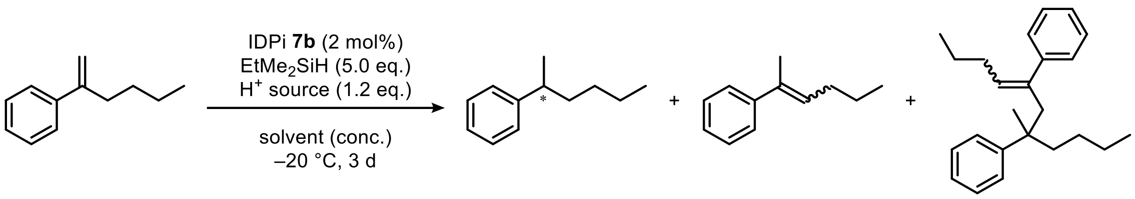

1a **2a** **3a** **4a**

H$^+$ source	solvent	conc.	conv.	yield (2a)	er	yield (3a)	yield (4a)
PhCOOH	CyMe	0.5 M	62%	51%	95.5:4.5	2%	5%
2,4,6-Me$_3$-C$_6$H$_2$OH	CyMe	0.5 M	79%	57%	92.5:7.5	4%	8%
HFIP	CyMe	0.5 M	>99%	68%	79:21	3%	12%
*i*PrOH	CyMe	0.5 M	0%	0%	-	0%	0%
H_2O	CyMe	0.5 M	0%	0%	-	0%	0%
AcOH	CyMe	0.5 M	22%	15%	95:5	1%	3%
PhCOOH	$CDCl_3$	0.5 M	68%	51%	90.5:9.5	7%	6%
PhCOOH	CD_2Cl_2	0.5 M	73%	48%	86:14	9%	8%
PhCOOH	THF	0.5 M	0%	0%	-	0%	0%

PhCOOH	Et_2O	0.5 M	45%	28%	93.5:6.5	7%	5%
PhCOOH	PhMe-*d8*	0.5 M	58%	38%	92:8	6%	8%
PhCOOH	*m*-xylene	0.5 M	43%	29%	93.5:6.5	6%	4%
PhCOOH	pentane	0.5 M	50%	27%	95.5:4.5	2%	10%
PhCOOH	hexanes	0.5 M	67%	36%	95:5	3%	14%
PhCOOH	CyMe	0.5 M	62%	51%	95.5:4.5	2%	5%
PhCOOH	CyMe	0.5 M	62%	51%	95.5:4.5	2%	5%
PhCOOH	CyMe	0.4 M	61%	41%	95.5:4.5	2%	7%
PhCOOH	CyMe	0.3 M	50%	41%	95.5:4.5	3%	3%
PhCOOH	CyMe	0.2 M	53%	36%	96:4	2%	4%
PhCOOH	CyMe	0.1 M	34%	24%	96:4	3%	1%

Reoptimization for other substrates

Table S3: *Reoptimization for substrate* ***1b****.*

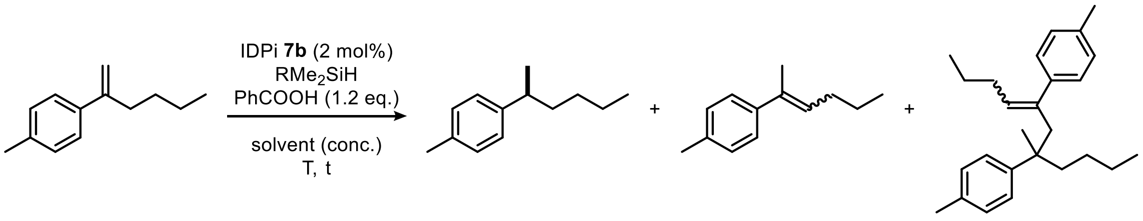

1b 2b 3b 4b

silane	solvent (conc.)	temp.	time	conv.	yield (2b)	er	yield (3b)	yield (4b)
$EtMe_2SiH$ (5 eq.)	CyMe (0.5 M)	–20 °C	3 d	98%	77%	87:13	4%	9%
$PhMe_2SiH$ (5 eq.)	CyMe (0.5 M)	–20 °C	3 d	>99%	60%	77.5:22.5	0%	13%
$EtMe_2SiH$ (10 eq.)	neat	–20 °C	3 d	96%	83%	88:12	8%	3%
$EtMe_2SiH$ (10 eq.)	neat	–30 °C	3 d	82%	60%	89.5:10.5	2%	10%
$EtMe_2SiH$ (10 eq.)	neat	–40 °C	3 d	95%	74%	91.5:8.5	2%	11%
$EtMe_2SiH$ (10 eq.)	neat	–50 °C	3 d	98%	79%	92:8	2%	9%
$EtMe_2SiH$ (10 eq.)	neat	–60 °C	3 d	19%	12%	94.5:5.5	0%	4%

Table S4: *Reoptimization for substrate* ***1c****.*

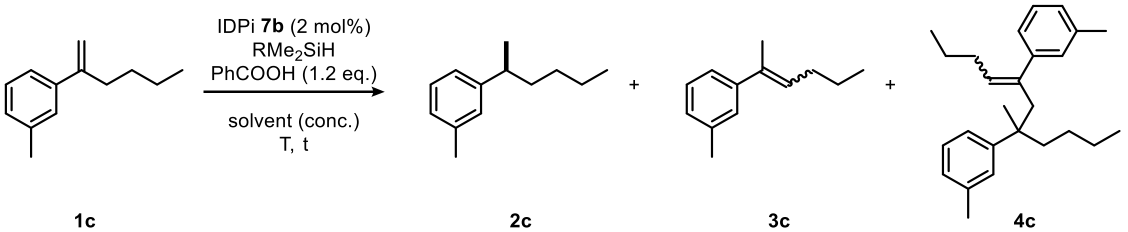

1c 2c 3c 4c

silane	solvent (conc.)	temp.	time	conv.	yield (2c)	er	yield (3c)	yield (4c)

$EtMe_2SiH$ (5 eq.)	CyMe (0.5 M)	–20 °C	3 d	91%	74%	96:4	4%	7%
$PhMe_2SiH$ (5 eq.)	CyMe (0.5 M)	–20 °C	3 d	100%	88%	94.5:5.5	1%	6%
$EtMe_2SiH$ (10 eq.)	neat	–20 °C	3 d	91%	86%	96:4	2%	2%

***Table S5:** Reoptimization for substrate **1d**.*

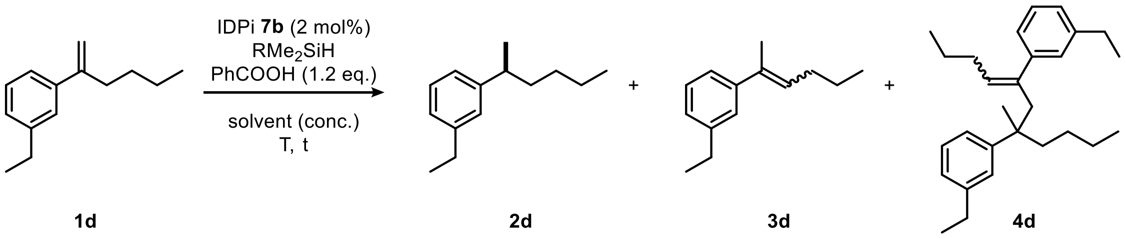

1d 2d 3d 4d

silane	solvent (conc.)	temp.	time	conv.	yield (2d)	er	yield (3d)	yield (4d)
$EtMe_2SiH$ (5 eq.)	CyMe (0.5 M)	–20 °C	3 d	77%	69%	96.5:3.5	2%	4%
$PhMe_2SiH$ (5 eq.)	CyMe (0.5 M)	–20 °C	3 d	>99%	93%	94.5:5.5	0%	4%
$EtMe_2SiH$ (10 eq.)	neat	–20 °C	3 d	>99%	95%	96.5:3.5	3%	1%

***Table S6:** Reoptimization for substrate **1e**.*

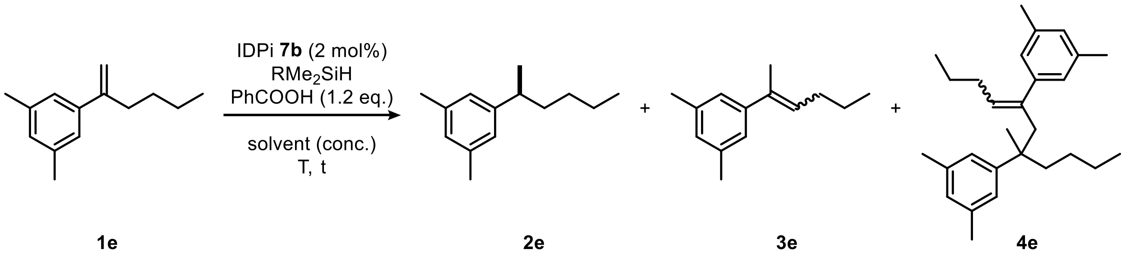

1e 2e 3e 4e

silane	solvent (conc.)	temp.	time	conv.	yield (2e)	er	yield (3e)	yield (4e)
$EtMe_2SiH$ (5 eq.)	CyMe (0.5 M)	–20 °C	3 d	99%	88%	96:4	3%	5%

$PhMe_2SiH$ (5 eq.)	CyMe (0.5 M)	–20 °C	3 d	>99%	93%	94:6	0%	4%

Table S7: *Reoptimization for substrate* ***1f****.*

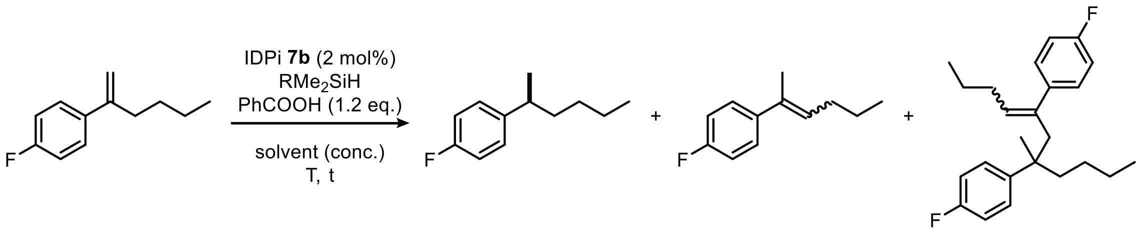

1f **2f** **3f** **4f**

silane	solvent (conc.)	temp.	time	conv.	yield (2f)	er	yield (3f)	yield (4f)
$EtMe_2SiH$ (5 eq.)	CyMe (0.5 M)	–20 °C	3 d	57%	46%	95.5:4.5	3%	4%
$PhMe_2SiH$ (5 eq.)	CyMe (0.5 M)	–20 °C	3 d	>99%	91%	95.5:4.5	1%	4%
$EtMe_2SiH$ (10 eq.)	neat	–20 °C	3 d	77%	69%	96:4	3%	3%

Table S8: *Reoptimization for substrate* ***1g****.*

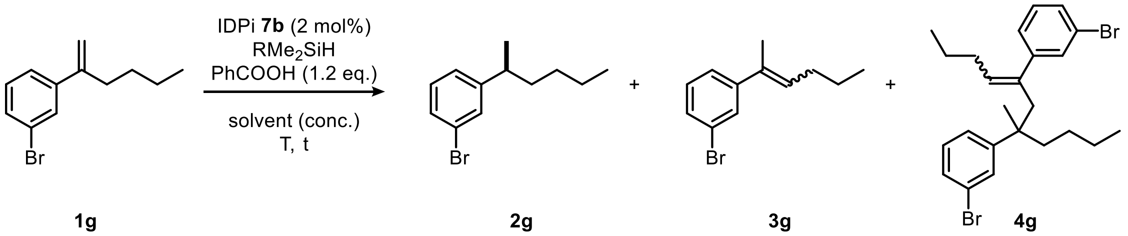

1g **2g** **3g** **4g**

silane	solvent (conc.)	temp.	time	conv.	yield (2g)	er	yield (3g)	yield (4g)
$EtMe_2SiH$ (5 eq.)	CyMe (0.5 M)	–20 °C	3 d	0%	0%	-	0%	0%
$PhMe_2SiH$ (5 eq.)	CyMe (0.5 M)	–20 °C	3 d	17%	13%	97.5:2.5	4%	0%
$PhMe_2SiH$ (5 eq.)	CyMe (0.5 M)	20 °C	3 d	52%	24%	95:5	28%	0%

$PhMe_2SiH$ (10 eq.)	neat	–20 °C	3 d	31%	29%	97:3	2%	0%
$PhMe_2SiH$ (10 eq.)	neat	10 °C	3 d	90%	75%	95:5	16%	0%

***Table S9:** Reoptimization for substrate **1h**.*

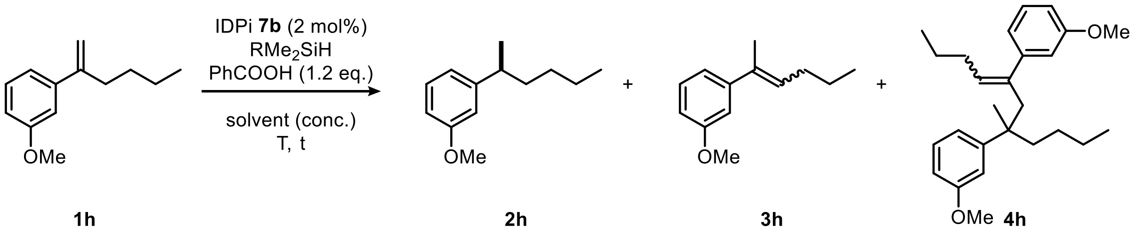

silane	solvent (conc.)	temp.	time	conv.	yield (2h)	er	yield (3h)	yield (4h)
$EtMe_2SiH$ (5 eq.)	CyMe (0.5 M)	–20 °C	3 d	38%	35%	98:2	3%	0%
$PhMe_2SiH$ (5 eq.)	CyMe (0.5 M)	–20 °C	6 d	>99%	99%	97:3	1%	0%

***Table S10:** Reoptimization for substrate **1j**.*

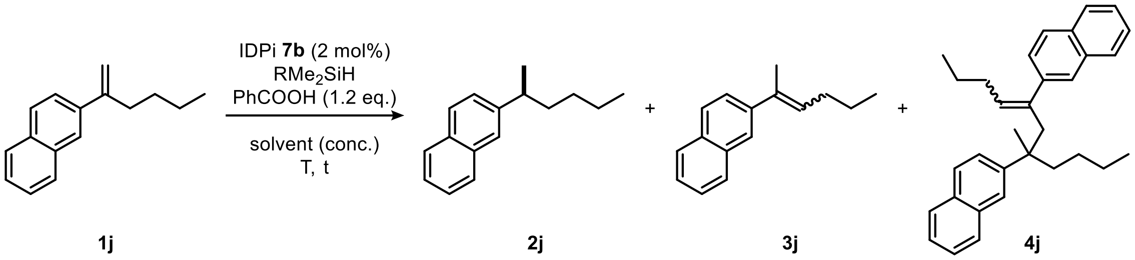

silane	solvent (conc.)	temp.	time	conv.	yield (2j)	er	yield (3j)	yield (4j)
$EtMe_2SiH$ (5 eq.)	CyMe (0.5 M)	–20 °C	3 d	>99%	67%	97:3	3%	21%
$PhMe_2SiH$ (5 eq.)	CyMe (0.5 M)	–20 °C	2 d	>99%	78%	95.5:4.5	1%	14%
$EtMe_2SiH$ (10 eq.)	neat	–20 °C	2 d	>99%	86%	97.5:2.5	3%	8%

Table S11: *Reoptimization for substrate* ***1k****.*

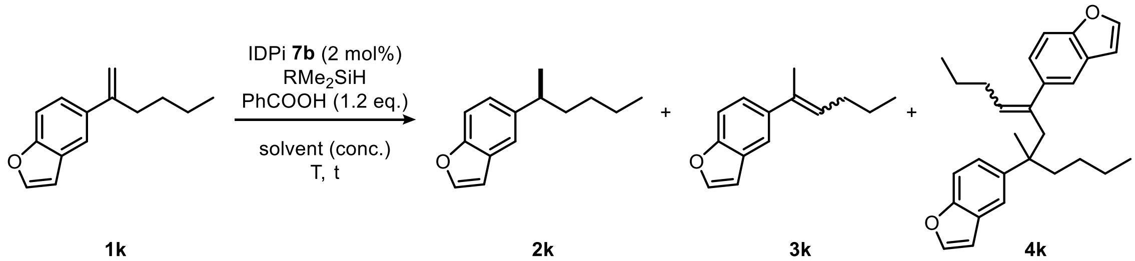

1k 2k 3k 4k

silane	solvent (conc.)	temp.	time	conv.	yield (2k)	er	yield (3k)	yield (4k)
$EtMe_2SiH$ (5 eq.)	CyMe (0.5 M)	–20 °C	3 d	>99%	66%	96:4	3%	10%
$PhMe_2SiH$ (5 eq.)	CyMe (0.5 M)	–20 °C	3 d	>99%	65%	94.5:5.5	1%	11%
$EtMe_2SiH$ (10 eq.)	neat	–20 °C	3 d	>99%	65%	96.5:3.5	0%	11%

Table S12: *Reoptimization for substrate* ***1l****.*

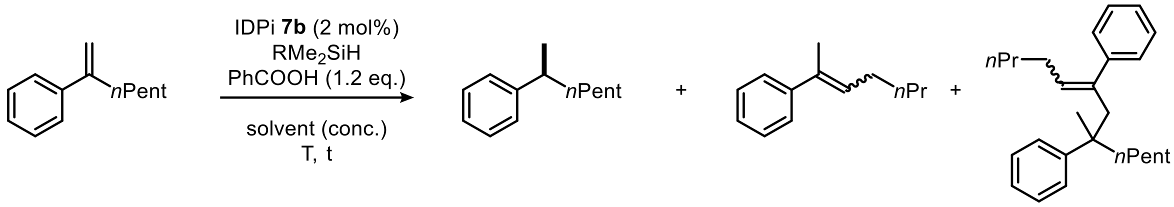

1l 2l 3l 4l

silane	solvent (conc.)	temp.	time	conv.	yield (2l)	er	yield (3l)	yield (4l)
$EtMe_2SiH$ (5 eq.)	CyMe (0.5 M)	–20 °C	3 d	65%	56%	94.5:5.5	4%	3%
$PhMe_2SiH$ (5 eq.)	CyMe (0.5 M)	–20 °C	3 d	98%	84%	92.5:7.5	4%	5%
$EtMe_2SiH$ (10 eq.)	neat	–20 °C	3 d	80%	73%	94:6	3%	2%

Table S13: *Reoptimization for substrate* ***1m***.

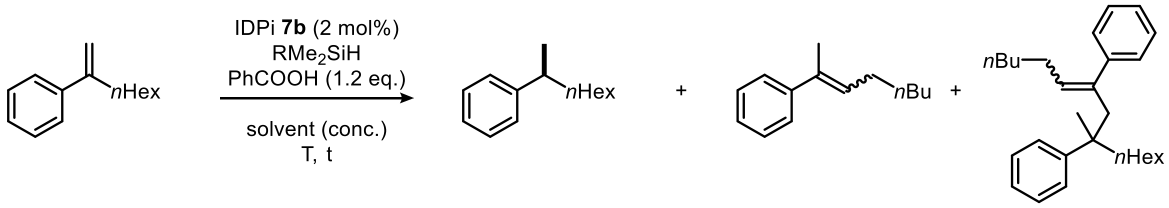

1m **2m** **3m** **4m**

silane	solvent (conc.)	temp.	time	conv.	yield (2m)	er	yield (3m)	yield (4m)
$EtMe_2SiH$ (5 eq.)	CyMe (0.5 M)	–20 °C	3 d	60%	45%	94:6	5%	5%
$PhMe_2SiH$ (5 eq.)	CyMe (0.5 M)	–20 °C	3 d	>99%	91%	92.5:7.5	2%	4%
$EtMe_2SiH$ (10 eq.)	neat	–20 °C	3 d	87%	83%	94.5:5.5	2%	1%

Table S14: *Reoptimization for substrate* ***1n***.

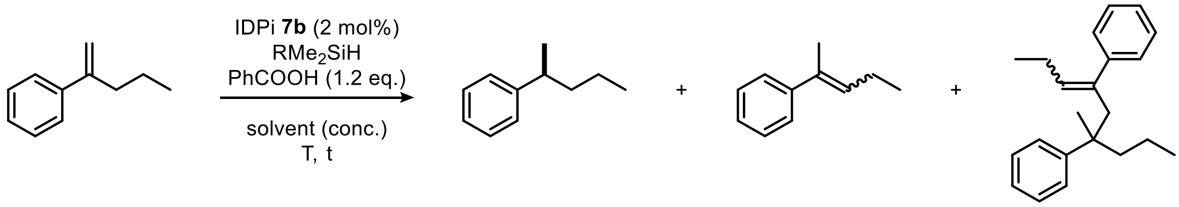

1n **2n** **3n** **4n**

silane	solvent (conc.)	temp.	time	conv.	yield (2n)	er	yield (3n)	yield (4n)
$EtMe_2SiH$ (5 eq.)	CyMe (0.5 M)	–20 °C	3 d	63%	40%	93:7	7%	9%
$PhMe_2SiH$ (5 eq.)	CyMe (0.5 M)	–20 °C	3 d	98%	77%	93.5:6.5	4%	8%
$EtMe_2SiH$ (10 eq.)	neat	–20 °C	3 d	70%	60%	93.5:6.5	3%	3%

*Table S15: Reoptimization for substrate **1o**.*

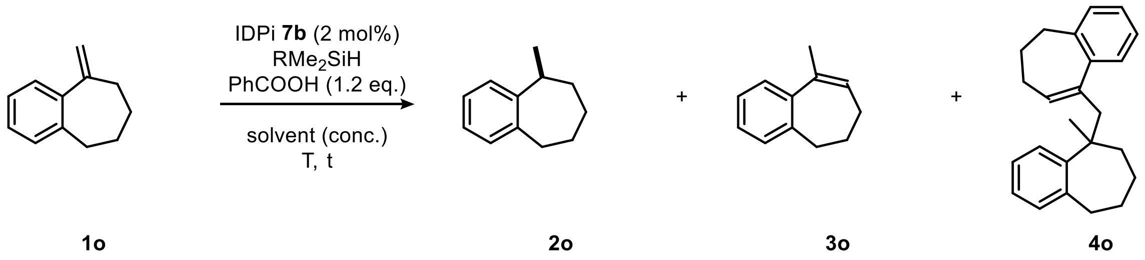

silane	solvent (conc.)	temp.	time	conv.	yield (2o)	er	yield (3o)	yield (4o)
$EtMe_2SiH$ (5 eq.)	CyMe (0.5 M)	–20 °C	3 d	45%	20%	91:9	25%	0%
$PhMe_2SiH$ (5 eq.)	CyMe (0.5 M)	–20 °C	3 d	81%	47%	92:8	34%	0%

*Table S16: Reoptimization for substrate **1p**.*

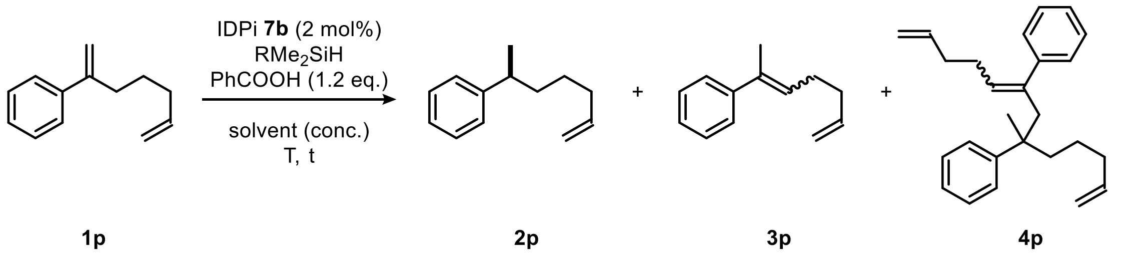

silane	solvent (conc.)	temp.	time	conv.	yield (2p)	er	yield (3p)	yield (4p)
$EtMe_2SiH$ (5 eq.)	CyMe (0.5 M)	–20 °C	3 d	>5%	traces	-	n.d.	n.d.
$PhMe_2SiH$ (5 eq.)	CyMe (0.5 M)	–20 °C	3 d	92%	42%	93.5:6.5	n.d.	n.d.
$EtMe_2SiH$ (10 eq.)	neat	–20 °C	3 d	23%	21%	95:5	n.d.	n.d.
$PhMe_2SiH$ (10 eq.)	neat	–20 °C	3 d	100%	53%	90.5:9.5	n.d.	n.d.

*Table S17: Reoptimization for substrate **1q**.*

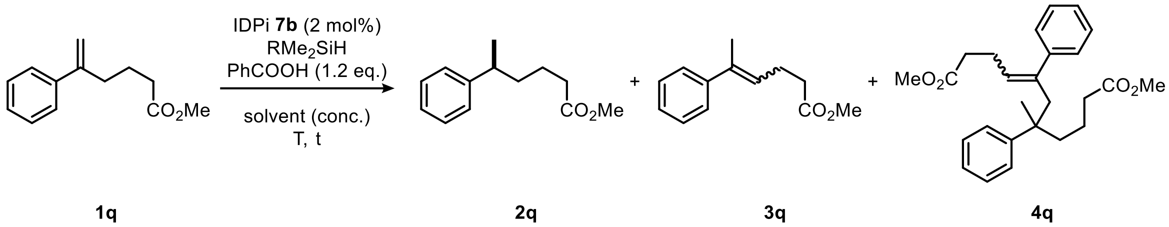

silane	solvent (conc.)	temp.	time	conv.	yield (2q)	er	yield (3q)	yield (4q)
$EtMe_2SiH$ (5 eq.)	CyMe (0.5 M)	–20 °C	3 d	70%	37%	90.5:9.5	12%	11%
$PhMe_2SiH$ (5 eq.)	CyMe (0.5 M)	–20 °C	3 d	99%	87%	92.5:7.5	3%	5%
$EtMe_2SiH$ (10 eq.)	neat	–20 °C	3 d	79%	58%	91:9	8%	7%

*Table S18: Reoptimization for substrate **1r**.*

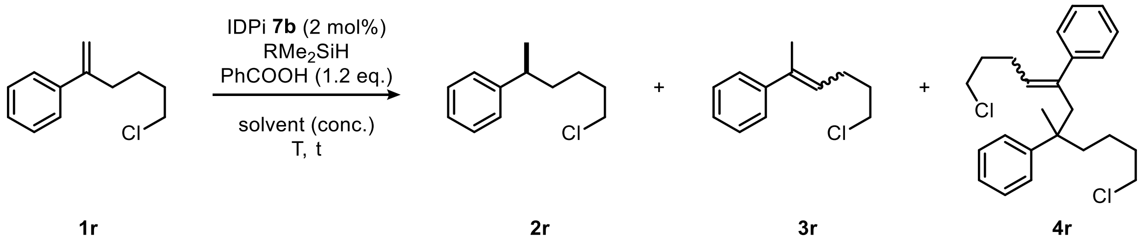

silane	solvent (conc.)	temp.	time	conv.	yield (2r)	er	yield (3r)	yield (4r)
$EtMe_2SiH$ (5 eq.)	CyMe (0.5 M)	–20 °C	3 d	42%	34%	90:10	7%	1%
$PhMe_2SiH$ (5 eq.)	CyMe (0.5 M)	–20 °C	3 d	86%	70%	90:10	6%	5%

3. Synthesis of Styrenes

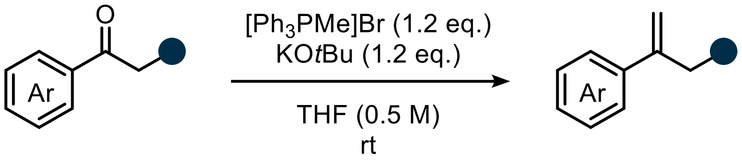

General Procedure 2 (GP2)

A flame-dried Schlenk flask under argon was charged with KO*t*Bu (1.2 eq.) and dry THF (0.5 M). $[Ph_3PMe]Br$ (1.2 eq.) was added to the suspension, and it was subsequently stirred at room temperature for 1 h. The corresponding ketone (1.0 eq.) was added and the resulting mixture was stirred for 1–18 h until TLC indicated full consumption of the starting material. The reaction was quenched by addition of water, followed by extraction of the aqueous phase with DCM (3x). The combined organic layers were washed with water, dried over anhydrous Na_2SO_4, filtered, and concentrated under reduced pressure. The crude was purified via flash column chromatography on silica gel.

hex-1-en-2-ylbenzene (1a)

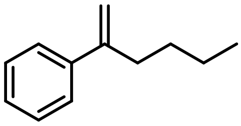

Prepared according to **GP2**, stirred for for 1 h, eluent: pentane, 1.86 g (96%), colorless oil.

$\boldsymbol{R}_F$ (100% hexanes) = 0.63.

^{1}H NMR (501 MHz, $CDCl_3$): δ = 7.43 – 7.39 (m, 2H), 7.36 – 7.30 (m, 2H), 7.26 (tt, *J* = 6.5, 1.4 Hz, 1H), 5.26 (d, *J* = 1.5 Hz, 1H), 5.06 (d, *J* = 1.4 Hz, 1H), 2.51 (td, *J* = 7.5, 1.3 Hz, 2H), 1.49 – 1.40 (m, 2H), 1.40 – 1.32 (m, 2H), 0.90 (t, *J* = 7.3 Hz, 3H).

^{13}C NMR (126 MHz, $CDCl_3$): δ = 148.9, 141.7, 128.4, 127.4, 126.3, 112.1, 35.2, 30.6, 22.6, 14.1.

HRMS *m/z* (GC-EI): calcd. For $C_{12}H_{16}$ ($[M]^+$): 160.124650; found: 160.124710.

1-(hex-1-en-2-yl)-4-methylbenzene (1b)

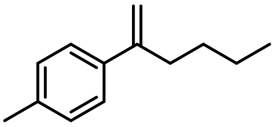

Prepared according to **GP2**, stirred for 18 h, eluent: pentane, 889 mg (97%), colorless oil.

***R*$_F$** (100% hexanes) = 0.59.

^{1}H NMR (501 MHz, $CDCl_3$): δ = 7.34 – 7.29 (m, 2H), 7.14 (d, *J* = 7.9 Hz, 2H), 5.24 (d, *J* = 1.7 Hz, 1H), 5.01 (d, *J* = 1.5 Hz, 1H), 2.49 (td, *J* = 7.5, 1.4 Hz, 2H), 2.35 (s, 3H), 1.49 – 1.39 (m, 2H), 1.39 – 1.31 (m, 2H), 0.90 (t, *J* = 7.3 Hz, 3H).

^{13}C NMR (126 MHz, $CDCl_3$): δ = 148.7, 138.7, 137.1, 129.1, 126.1, 111.4, 35.2, 30.7, 22.6, 21.2, 14.1.

HRMS *m/z* (GC-EI): calcd. For $C_{13}H_{18}$ ($[M]^+$): 174.140300; found: 174.140300.

1-(hex-1-en-2-yl)-3-methylbenzene (1c)

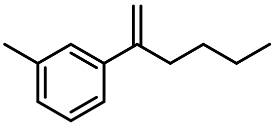

Prepared according to **GP2**, stirred for 18 h, eluent: pentane, 358 mg (99%), colorless oil.

***R*$_F$** (100% hexanes) = 0.59.

^{1}H NMR (501 MHz, $CDCl_3$): δ = 7.24 – 7.18 (m, 3H), 7.08 (ddd, *J* = 5.9, 2.7, 1.3 Hz, 1H), 5.24 (d, *J* = 1.6 Hz, 1H), 5.03 (q, *J* = 1.4 Hz, 1H), 2.53 – 2.46 (m, 2H), 2.36 (s, 3H), 1.48 – 1.39 (m, 2H), 1.39 – 1.30 (m, 2H), 0.90 (t, *J* = 7.2 Hz, 3H).

^{13}C NMR (126 MHz, $CDCl_3$): δ = 149.1, 141.7, 137.8, 128.2, 128.1, 127.0, 123.4, 111.9, 35.3, 30.6, 22.6, 21.7, 14.1.

HRMS *m/z* (GC-EI): calcd. For $C_{13}H_{18}$ ($[M]^+$): 174.140300; found: 174.140450.

1-ethyl-3-(hex-1-en-2-yl)benzene (1d)

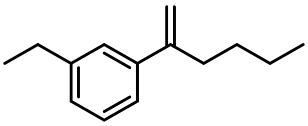

Prepared according to **GP2**, stirred for 18 h, eluent: pentane, 1.08 g (96%), colorless oil.

***R*$_F$** (100% hexanes) = 0.59.

^{1}H NMR (501 MHz, CD_2Cl_2): δ = 7.26 – 7.19 (m, 3H), 7.10 (dt, *J* = 6.7, 2.0 Hz, 1H), 5.24 (d, *J* = 1.7 Hz, 1H), 5.03 (q, *J* = 1.5 Hz, 1H), 2.65 (q, *J* = 7.6 Hz, 2H), 2.53 – 2.47 (m, 2H), 1.52 (s, 2H), 1.46 – 1.38 (m, 2H), 1.38 – 1.31 (m, 2H), 1.23 (t, *J* = 7.6 Hz, 3H), 0.90 (t, *J* = 7.2 Hz, 3H).

^{13}C NMR (126 MHz, CD_2Cl_2): δ = 149.5, 144.7, 141.9, 128.5, 127.2, 126.1, 123.8, 111.8, 35.4, 31.0, 29.3, 22.8, 15.9, 14.1.

HRMS *m/z* (GC-EI): calcd. For $C_{14}H_{20}$ ($[M]^+$): 188.155950; found: 188.155860.

1-(hex-1-en-2-yl)-3,5-dimethylbenzene (1e)

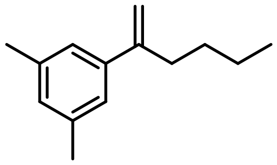

Prepared according to **GP2**, stirred for 18 h, eluent: pentane, 873 mg (94%), colorless oil.

***R*$_F$** (100% hexanes) = 0.56.

^{1}H NMR (501 MHz, CD_2Cl_2): δ = 7.03 – 6.99 (m, 2H), 6.91 (dt, *J* = 1.7, 0.9 Hz, 1H), 5.20 (d, *J* = 1.8 Hz, 1H), 5.00 (q, *J* = 1.4 Hz, 1H), 2.51 – 2.44 (m, 2H), 2.30 (d, *J* = 0.8 Hz, 6H), 1.46 – 1.37 (m, 2H), 1.37 – 1.30 (m, 2H), 0.89 (t, *J* = 7.2 Hz, 3H).

^{13}C NMR (126 MHz, CD_2Cl_2): δ = 149.6, 141.8, 138.0, 129.2, 124.3, 111.7, 35.5, 31.0, 22.8, 21.5, 14.1.

HRMS *m/z* (GC-EI): calcd. For $C_{14}H_{20}$ ($[M]^+$): 188.155950; found: 188.155830.

1-fluoro-4-(hex-1-en-2-yl)benzene (1f)

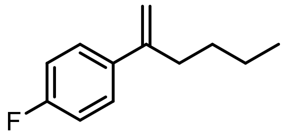

Prepared according to **GP2**, stirred for 18 h, eluent: pentane, 1.73 g (84%), colorless oil.

***R*$_F$** (100% hexanes) = 0.57.

^{1}H NMR (501 MHz, $CDCl_3$): δ = 7.39 – 7.33 (m, 2H), 7.03 – 6.97 (m, 2H), 5.20 (d, *J* = 1.4 Hz, 1H), 5.03 (q, *J* = 1.4 Hz, 1H), 2.50 – 2.44 (m, 2H), 1.45 – 1.38 (m, 2H), 1.38 – 1.30 (m, 2H), 0.90 (t, *J* = 7.2 Hz, 3H).

^{19}F NMR (471 MHz, $CDCl_3$): δ = –115.7 (s, 1F).

^{13}C NMR (126 MHz, $CDCl_3$): δ = 162.2 (d, *J* = 245.9 Hz), 147.7, 137.5 (d, *J* = 3.3 Hz), 127.6 (d, *J* = 7.9 Hz), 115.0 (d, *J* = 21.3 Hz), 111.9, 35.2, 30.3, 22.3, 13.9.

HRMS *m/z* (GC-EI): calcd. For $C_{12}H_{15}F_1$ ($[M]^+$): 178.115228; found: 178.115410.

1-bromo-3-(hex-1-en-2-yl)benzene (1g)

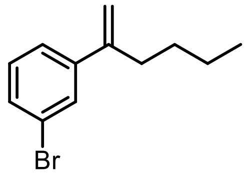

Prepared according to **GP2**, stirred for 18 h, eluent: pentane, 1.32 g (96%), colorless oil.

***R*F** (100% hexanes) = 0.82.

^{1}H NMR (501 MHz, $CDCl_3$): δ = 7.54 (t, *J* = 1.9 Hz, 1H), 7.39 (ddd, *J* = 7.9, 2.1, 1.1 Hz, 1H), 7.34 – 7.29 (m, 1H), 7.19 (t, *J* = 7.8 Hz, 1H), 5.25 (d, *J* = 1.4 Hz, 1H), 5.08 (q, *J* = 1.4 Hz, 1H), 2.49 – 2.43 (m, 2H), 1.46 – 1.38 (m, 2H), 1.38 – 1.30 (m, 2H), 0.90 (t, *J* = 7.2 Hz, 3H).

^{13}C NMR (126 MHz, $CDCl_3$): δ = 147.7, 143.9, 130.3, 129.9, 129.4, 124.9, 122.6, 113.4, 35.1, 30.4, 22.5, 14.0.

HRMS *m/z* (GC-EI): calcd. For $C_{12}H_{15}Br_1$ ($[M]^+$): 238.035175; found: 238.035510.

1-(hex-1-en-2-yl)-3-methoxybenzene (1h)

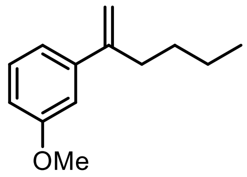

Prepared according to **GP2**, stirred for 18 h, eluent: pentane, 508 mg (72%), light yellow oil.

***R*F** (20% DCM, hexanes) = 0.42.

^{1}H NMR (501 MHz, $CDCl_3$): δ = 7.28 – 7.22 (m, 1H), 7.03 – 6.98 (m, 1H), 6.95 (q, *J* = 2.0 Hz, 1H), 6.82 (dt, *J* = 8.4, 2.2 Hz, 1H), 5.26 (d, *J* = 1.8 Hz, 1H), 5.07 – 5.01 (m, 1H), 3.83 (d, *J* = 1.7 Hz, 3H), 2.52 – 2.44 (m, 2H), 1.48 – 1.40 (m, 2H), 1.40 – 1.30 (m, 2H), 0.90 (td, *J* = 7.3, 1.7 Hz, 3H).

^{13}C NMR (126 MHz, $CDCl_3$): δ = 159.7, 148.8, 143.3, 129.3, 118.9, 112.5, 112.3, 112.3, 55.4, 35.3, 30.6, 22.6, 14.1.

HRMS *m/z* (GC-EI): calcd. For $C_{13}H_{15}O_1$ ($[M]^+$): 190.135215; found: 190.135120.

2-(hex-1-en-2-yl)naphthalene (1j)

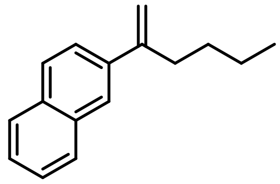

Prepared according to **GP2**, stirred for 18 h, eluent: pentane, 614 mg (99%), colorless oil.

***R*F** (100% hexanes) = 0.51.

^{1}H NMR (501 MHz, $CDCl_3$): δ = 7.86 – 7.78 (m, 4H), 7.59 (dd, *J* = 8.6, 1.8 Hz, 1H), 7.50 – 7.42 (m, 2H), 5.41 (d, *J* = 1.5 Hz, 1H), 5.17 (q, *J* = 1.4 Hz, 1H), 2.63 (td, *J* = 7.6, 1.3 Hz, 2H), 1.56 – 1.46 (m, 2H), 1.44 – 1.32 (m, 2H), 0.92 (t, *J* = 7.3 Hz, 3H).

^{13}C NMR (126 MHz, $CDCl_3$): δ = 148.7, 138.9, 133.6, 132.9, 128.3, 127.9, 127.7, 126.2, 125.8, 124.9, 124.8, 112.8, 35.2, 30.7, 22.6, 14.1.

HRMS *m/z* (GC-EI): calcd. For $C_{16}H_{18}$ ($[M]^+$): 210.140300; found: 210.140170.

Synthesis of 5-(hex-1-en-2-yl)benzofuran (1k)

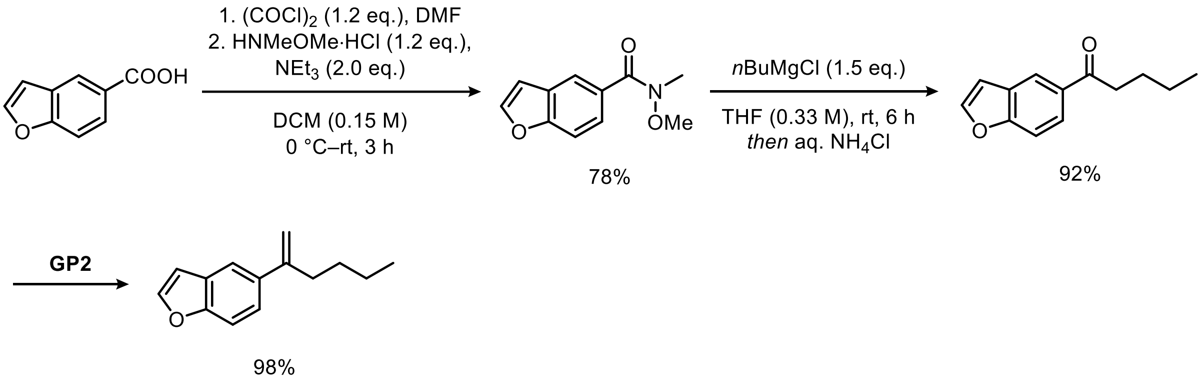

***N*-methoxy-*N*-methylbenzofuran-5-carboxamide (S3)**

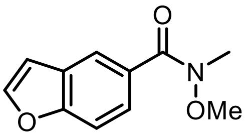

In a flame-dried Schlenk flask under argon, benzofuran-5-carboxylic acid (760 mg, 4.69 mmol, 1.00 eq.) was suspended in dry DCM (10 mL), cooled to 0 °C, and one drop of DMF was added. Oxalyl chloride (0.49 mL, 5.62 mmol, 1.20 eq.) was then added slowly at 0 °C. The reaction mixture was stirred at room temperature for 2 h until the suspension turned into a solution. The mixture was transferred to a 100 mL round-bottom flask and concentrated under reduced pressure. The resulting crude acid chloride was redissolved in dry DCM (5 mL, plus 5 mL for rinsing) and added to a second flame-dried Schlenk flask containing *N,O*-dimethylhydroxylamine hydrochloride (549 mg, 5.62 mmol, 1.20 eq.) in DCM (20 mL) at 0 °C. Et_3N (1.3 mL, 9.37 mmol, 2.00 eq.) was added dropwise at 0 °C, after which the mixture was warmed to room temperature and stirred overnight. The reaction was quenched with saturated NH_4Cl (30 mL) solution and extracted with DCM (3 x 20 mL). The combined organic layers were washed with water, dried over anhydrous Na_2SO_4, and concentrated. Purification by flash column chromatography on silica gel (30% EtOAc, cyclohexane) afforded the product as a yellow oil (754 mg, 78%).

^{1}**H NMR** (501 MHz, $CDCl_3$): δ = 7.99 (d, *J* = 1.7 Hz, 1H), 7.67 (td, *J* = 4.0, 1.7 Hz, 2H), 7.51 (dt, *J* = 8.6, 0.8 Hz, 1H), 6.82 (dd, *J* = 2.2, 1.0 Hz, 1H), 3.56 (s, 3H), 3.39 (s, 3H).

^{13}**C NMR** (126 MHz, $CDCl_3$): δ = 170.2, 156.1, 146.1, 129.0, 127.2, 125.1, 122.2, 111.1, 107.1, 61.1, 34.1.

HRMS *m/z* (GC-EI): calcd. For $C_{11}H_{11}O_3N_1Na_1$ ($[M+Na]^+$): 228.063113; found: 228.063100.

1-(benzofuran-5-yl)pentan-1-one (S4)

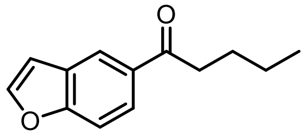

Weinreb amide **S3** (754 mg, 3.67 mmol, 1.00 eq.) was dissolved in dry THF (11 mL) and cooled to 0 °C. *n*-Butylmagnesium chloride (2 M in THF, 2.8 mL, 5.6 mmol, 1.52 eq.) was added dropwise at 0 °C, and the reaction mixture was then warmed to room temperature and stirred for 6 h. After completion, as monitored by TLC, saturated NH_4Cl solution was added, and the mixture was stirred at room temperature for 1 h. The aqueous phase was extracted with DCM (3 x 20 mL), and the combined organic layers were dried over anhydrous Na_2SO_4 and concentrated under reduced pressure. Purification by flash column chromatography on silica gel (0–4% MTBE in pentane) afforded the product as a white solid (682 mg, 92%).

^{1}**H NMR** (501 MHz, $CDCl_3$): δ = 8.26 (d, *J* = 1.8 Hz, 1H), 7.97 (dd, *J* = 8.7, 1.8 Hz, 1H), 7.69 (d, *J* = 2.2 Hz, 1H), 7.54 (dt, *J* = 8.7, 0.9 Hz, 1H), 6.86 (dd, *J* = 2.2, 0.9 Hz, 1H), 3.06 – 2.99 (m, 2H), 1.80 – 1.71 (m, 2H), 1.48 – 1.39 (m, 2H), 0.97 (t, *J* = 7.3 Hz, 3H).

^{13}**C NMR** (126 MHz, $CDCl_3$): δ = 200.3, 157.5, 146.5, 132.8, 127.6, 125.0, 122.4, 111.6, 107.4, 38.6, 26.9, 22.7, 14.1.

HRMS *m/z* (GC-EI): calcd. For $C_{13}H_{14}O_2$ ($[M]^+$): 202.098830; found: 202.098770.

5-(hex-1-en-2-yl)benzofuran (1k)

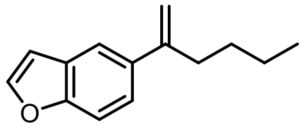

Prepared according to **GP2**, stirred for 18 h, eluent: pentane, 664 mg (98%), colorless oil.

$\boldsymbol{R}_{\mathbf{F}}$ (100% hexanes) = 0.43.

^{1}H NMR (501 MHz, $CDCl_3$): δ = 7.61 (dt, *J* = 2.1, 1.2 Hz, 2H), 7.45 (d, *J* = 8.6 Hz, 1H), 7.36 (d, *J* = 8.5 Hz, 1H), 6.76 (dt, *J* = 2.1, 1.0 Hz, 1H), 5.24 (s, 1H), 5.06 (s, 1H), 2.55 (t, *J* = 7.5 Hz, 2H), 1.49 – 1.41 (m, 2H), 1.41 – 1.30 (m, 2H), 0.90 (td, *J* = 7.3, 1.1 Hz, 3H).

^{13}C NMR (126 MHz, $CDCl_3$): δ = 154.6, 149.2, 145.4, 136.8, 127.5, 123.1, 118.8, 111.8, 111.1, 106.9, 35.8, 30.6, 22.6, 14.1.

HRMS *m/z* (GC-EI): calcd. For $C_{14}H_{16}O_1$ ($[M]^+$): 200.119565; found: 200.119510.

hept-1-en-2-ylbenzene (1l)

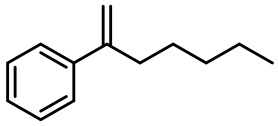

Prepared according to **GP2**, stirred for 18 h, eluent: pentane, 1.43 g (95%), colorless oil.

***R*$_F$** (100% hexanes) = 0.63.

^{1}H NMR (501 MHz, $CDCl_3$): δ = 7.43 – 7.39 (m, 2H), 7.35 – 7.30 (m, 2H), 7.26 (tt, *J* = 6.9, 1.4 Hz, 1H), 5.26 (d, *J* = 1.6 Hz, 1H), 5.06 (q, *J* = 1.4 Hz, 1H), 2.50 (td, *J* = 7.6, 1.3 Hz, 2H), 1.50 – 1.41 (m, 2H), 1.36 – 1.24 (m, 4H), 0.94 – 0.84 (m, 3H).

^{13}C NMR (126 MHz, $CDCl_3$): δ = 149.0, 141.7, 128.4, 127.4, 126.3, 112.1, 35.5, 31.7, 28.1, 22.6, 14.2.

HRMS *m/z* (GC-EI): calcd. For $C_{13}H_{18}$ ($[M]^+$): 174.140300; found: 174.140370.

oct-1-en-2-ylbenzene (1m)

Prepared according to **GP2**, stirred for 18 h, eluent: pentane, 893 mg (79%), colorless oil.

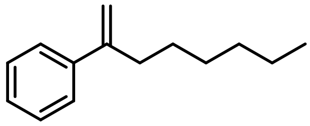

***R*$_F$** (100% hexanes) = 0.63.

^{1}H NMR (501 MHz, $CDCl_3$): δ = 7.42 – 7.36 (m, 2H), 7.34 – 7.27 (m, 2H), 7.27 – 7.21 (m, 1H), 5.24 (d, *J* = 1.5 Hz, 1H), 5.04 (q, *J* = 1.4 Hz, 1H), 2.48 (td, *J* = 7.5, 1.3 Hz, 2H), 1.48 – 1.38 (m, 2H), 1.36 – 1.29 (m, 2H), 1.29 – 1.19 (m, 4H), 0.89 – 0.82 (m, 3H).

^{13}C NMR (126 MHz, $CDCl_3$) δ 148.8, 141.5, 128.2, 127.2, 126.1, 111.9, 35.3, 31.6, 29.0, 28.2, 22.6, 14.0.

HRMS *m/z* (GC-EI): calcd. For $C_{14}H_{20}$ ($[M]^+$): 188.155950; found: 188.155900.

pent-1-en-2-ylbenzene (1n)

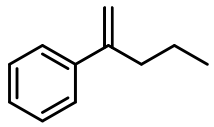

Prepared according to **GP2**, stirred for 18 h, eluent: pentane, 737 mg (82%), colorless oil.

***R*F** (100% hexanes) = 0.63.

^{1}H NMR (501 MHz, $CDCl_3$): δ = 7.43 – 7.39 (m, 2H), 7.35 – 7.30 (m, 2H), 7.29 – 7.24 (m, 1H), 5.28 (d, *J* = 1.6 Hz, 1H), 5.06 (q, *J* = 1.4 Hz, 1H), 2.49 (td, *J* = 7.5, 1.3 Hz, 2H), 1.55 – 1.43 (m, 2H), 0.93 (t, *J* = 7.4 Hz, 3H).

^{13}C NMR (126 MHz, $CDCl_3$): δ = 148.7, 141.6, 128.4, 127.4, 126.3, 126.3, 112.3, 37.6, 21.5, 13.9.

HRMS *m/z* (GC-EI): calcd. For $C_{11}H_{14}$ ($[M]^+$): 146.109000; found: 146.109020.

5-methylene-6,7,8,9-tetrahydro-5*H*-benzo[7]annulene (1o)

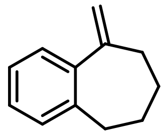

Prepared according to **GP2**, stirred for 18 h, eluent: pentane, 649 mg (88%), colorless oil.

***R*F** (100% hexanes) = 0.60.

^{1}H NMR (501 MHz, $CDCl_3$): δ = 7.23 – 7.19 (m, 1H), 7.19 – 7.13 (m, 2H), 7.12 – 7.07 (m, 1H), 5.11 (dt, *J* = 2.1, 1.0 Hz, 1H), 4.99 (d, *J* = 2.2 Hz, 1H), 2.80 – 2.75 (m, 2H), 2.43 – 2.36 (m, 2H), 1.84 (td, *J* = 7.4, 4.5 Hz, 2H), 1.78 – 1.72 (m, 2H).

^{13}C NMR (126 MHz, $CDCl_3$): δ = 153.0, 144.3, 140.4, 129.1, 128.3, 127.2, 126.2, 113.8, 36.7, 36.5, 31.6, 27.5.

HRMS *m/z* (GC-EI): calcd. For $C_{12}H_{14}$ ($[M]^+$): 158.109000; found: 158.109170.

Synthesis of hepta-1,6-dien-2-ylbenzene (1p)

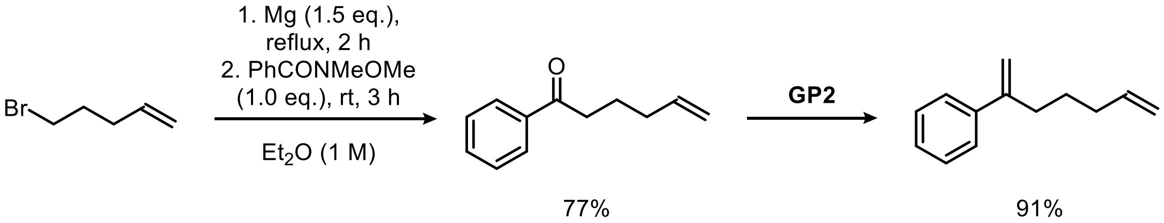

1-phenylhex-5-en-1-one (S5)

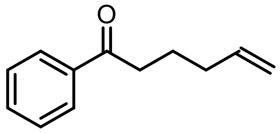

A flame-dried flask equipped with a reflux condenser under argon was charged with Mg (221 mg, 9.08 mmol, 1.50 eq.), Et_2O (8 mL), and a small crystal of I_2. The mixture was heated to reflux for 15 min until decolorized. 5-Bromopent-1-ene (0.93 mL, 7.87 mmol, 1.30 eq.) was added dropwise at room temperature, and the reaction was heated to reflux for 2 h. After cooling to room temperature, N-methoxy-N-methylbenzamide (1.00 g, 6.05 mmol, 1.00 eq.) was added, and the mixture was stirred at ambient temperature for an additional 3 h. The reaction was quenched with 10% HCl (x mL) and stirred for 30 min. The aqueous layer was extracted with DCM (3 × 30 mL), and the combined organic layers were washed with water, dried over anhydrous Na_2SO_4, and concentrated under reduced pressure. Purification by flash column chromatography on silica gel (1% MTBE in pentane) afforded the title compound as a colorless oil (811 mg, 77%).

^{1}H NMR (501 MHz, CD_2Cl_2) δ 7.97 – 7.92 (m, 2H), 7.60 – 7.53 (m, 1H), 7.50 – 7.44 (m, 2H), 5.85 (ddt, *J* = 17.0, 10.2, 6.7 Hz, 1H), 5.05 (dq, *J* = 17.2, 1.7 Hz, 1H), 4.99 (ddt, *J* = 10.2, 2.3, 1.3 Hz, 1H), 2.98 (t, *J* = 7.3 Hz, 2H), 2.19 – 2.12 (m, 2H), 1.82 (p, *J* = 7.4 Hz, 2H).

^{13}C NMR (126 MHz, CD_2Cl_2): δ = 200.3, 138.7, 137.6, 133.2, 128.9, 128.3, 115.2, 38.1, 33.6, 23.7.

HRMS *m/z* (GC-EI): calcd. For $C_{12}H_{14}O_1$ ($[M]^+$): 174.103915; found: 174.103970.

hepta-1,6-dien-2-ylbenzene (1p)

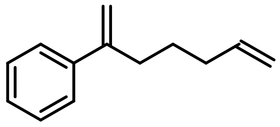

Prepared according to **GP2**, stirred for 4 h, eluent: pentane, 175 mg (91%), colorless oil.

***R*$_F$** (100% hexanes) = 0.51.

^{1}H NMR (501 MHz, $CDCl_3$): δ = 7.43 – 7.38 (m, 2H), 7.36 – 7.30 (m, 2H), 7.29 – 7.24 (m, 1H), 5.81 (ddt, *J* = 17.0, 10.2, 6.7 Hz, 1H), 5.28 (d, *J* = 1.5 Hz, 1H), 5.07 (q, *J* = 1.4 Hz, 1H), 5.01 (dq,

J = 17.2, 1.7 Hz, 1H), 4.96 (ddt, *J* = 10.2, 2.3, 1.2 Hz, 1H), 2.55 – 2.50 (m, 2H), 2.13 – 2.06 (m, 2H), 1.60 – 1.52 (m, 2H).

^{13}C NMR (126 MHz, $CDCl_3$): δ = 148.6, 141.5, 138.8, 128.4, 127.4, 126.3, 114.8, 112.5, 34.9, 33.5, 27.6.

HRMS *m/z* (GC-EI): calcd. For $C_{13}H_{16}$ ($[M]^+$): 172.124650; found: 172.124590.

methyl 5-phenylhex-5-enoate (1q)

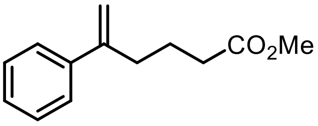

Prepared according to **GP2**, stirred for 4 h, eluent: 2% MTBE, pentane, 1.34 g (95%), colorless oil.

R R_F (5% EtOAc, hexanes) = 0.29.

^{1}H NMR (501 MHz, $CDCl_3$): δ = 7.42 – 7.38 (m, 2H), 7.36 – 7.30 (m, 2H), 7.29 – 7.24 (m, 1H), 5.30 (d, *J* = 1.4 Hz, 1H), 5.07 (q, *J* = 1.3 Hz, 1H), 3.66 (s, 3H), 2.55 (td, *J* = 7.5, 1.3 Hz, 2H), 2.34 (t, *J* = 7.4 Hz, 2H), 1.79 (p, *J* = 7.5 Hz, 2H).

^{13}C NMR (126 MHz, $CDCl_3$): δ = 174.1, 147.6, 141.0, 128.5, 127.6, 126.2, 113.1, 51.6, 34.7, 33.5, 23.5.

HRMS *m/z* (GC-EI): calcd. For $C_{13}H_{16}O_2$ ($[M]^+$): 204.114480; found: 204.114500.

Synthesis of (6-chlorohex-1-en-2-yl)benzene (1r)

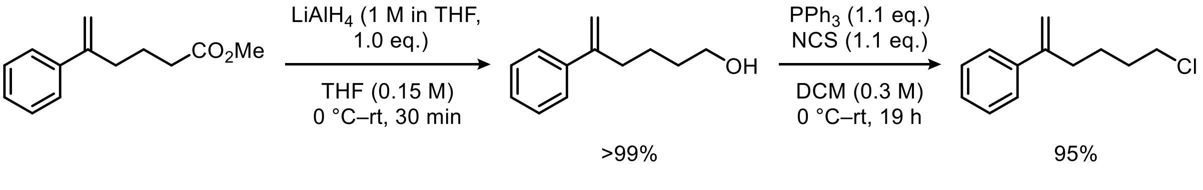

5-phenylhex-5-en-1-ol (S6)

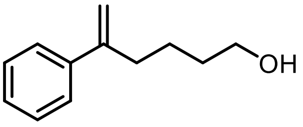

In a flame-dried Schlenk flask under argon methyl 5-phenylhex-5-enoate (**1p**) (806 mg, 3.95 mmol, 1.00 eq.) was dissolved in THF (26 mL) and cooled to 0 °C. $LiAlH_4$ (1 M in THF, 3.9 mL, 3.9 mmol, 0.99 eq.) was added dropwise and the resulting mixture was stirred at room temperature for 30 min. The reaction was quenched by the addition of 5% NaOH solution at 0 °C and subsequently extracted with DCM (3 x 30 mL). The combined organic layers were washed with

water, dried over anhydrous Na_2SO_4 and concentrated under reduced pressure. The colorless oil (695 mg, >99%) was used without further purification.

^{1}H NMR (501 MHz, $CDCl_3$): δ = 7.42 – 7.37 (m, 2H), 7.36 – 7.29 (m, 2H), 7.29 – 7.24 (m, 1H), 5.27 (d, *J* = 1.5 Hz, 1H), 5.07 (q, *J* = 1.4 Hz, 1H), 3.63 (t, *J* = 6.5 Hz, 2H), 2.54 (td, *J* = 7.4, 1.3 Hz, 2H), 1.64 – 1.57 (m, 2H), 1.57 – 1.47 (m, 2H), 1.34 (s, 1H).

^{13}C NMR (126 MHz, $CDCl_3$): δ = 148.5, 141.4, 128.4, 127.5, 126.3, 112.6, 63.0, 35.2, 32.5, 24.5.

HRMS *m/z* (GC-EI): calcd. For $C_{12}H_{16}O_1$ ($[M]^+$): 176.119565; found: 176.119470.

(6-chlorohex-1-en-2-yl)benzene (1r)

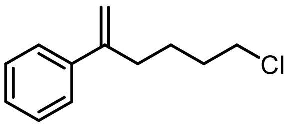

A flame-dried Schlenk flask under argon was charged with 5-phenylhex-5-en-1-ol (438 mg, 2.49 mmol, 1.00 eq.) and dry DCM (8.3 mL). At 0 °C PPh_3 (708 mg, 2.70 mmol, 1.09 eq.) and NCS (361 mg, 2.70 mmol, 1.09 eq.) were added and the mixture was subsequently stirred at room temperature for 19 h. After addition of water the aqueous phase was extracted with DCM (3 x 20 mL). The combined organic layers were washed with water, dried over anhydrous Na_2SO_4 and concentrated under reduced pressure. Purification via flash column chromatography on silica gel (100% pentane) furnished the title compound as a colorless oil (459 mg, 95%).

R_F (100% hexanes) = 0.29.

^{1}H NMR (501 MHz, $CDCl_3$): δ = 7.44 – 7.38 (m, 2H), 7.37 – 7.31 (m, 2H), 7.31 – 7.25 (m, 1H), 5.29 (s, 1H), 5.14 – 5.05 (m, 1H), 3.58 – 3.47 (m, 2H), 2.55 (t, *J* = 7.5 Hz, 2H), 1.87 – 1.75 (m, 2H), 1.69 – 1.56 (m, 2H).

^{13}C NMR (126 MHz, $CDCl_3$): δ = 148.1, 141.2, 128.5, 127.6, 126.3, 112.8, 45.0, 34.7, 32.3, 25.5.

HRMS *m/z* (GC-EI): calcd. For $C_{12}H_{15}Cl_1$ ($[M]^+$): 194.085678; found: 194.085630.

(*E*)- and (*Z*)-2-(hex-2-en-2-yl)naphthalene (3j)

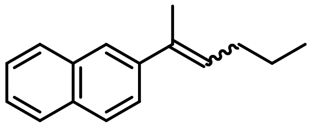

A flame-dried Schlenk flask under argon was charged with *n*-butyltriphenylphosphonium bromide (1.70 g, 4.26 mmol, 1.00 eq.) and anhydrous THF (8.5 mL). The suspension was cooled to 0 °C,

and *n*-BuLi (2.5 M in heptane, 1.7 mL, 4.26 mmol, 1.00 eq.) was added dropwise. After stirring the resulting solution at 0 °C for 15 min, 1-(naphthalen-2-yl)ethan-1-one (1.01 g, 5.11 mmol, 1.20 eq.) dissolved in 4 mL THF was added. The reaction was then warmed to room temperature and stirred overnight (18 h). By addition of saturated ammonium chloride solution, the reaction was quenched. The aqueous phase was extracted with Et_2O (3 x 30 mL), and the combined organic layers were washed with brine, dried over anhydrous Na_2SO_4 and concentrated under reduced pressure. Purification by flash column chromatography on silica gel (100% pentane) yielded the pure (*E*)-isomer (69 mg, 8%) and (*Z*)-isomer (82 mg, 9%) of **3j**, along with a mixed fraction (*E*/*Z* = 39:61, 222 mg, 25%), all as colorless oils.

(*E*)-isomer:

^{1}H NMR (501 MHz, $CDCl_3$): δ = 7.85 – 7.75 (m, 4H), 7.60 (dd, *J* = 8.6, 1.9 Hz, 1H), 7.49 – 7.39 (m, 2H), 5.97 (td, *J* = 7.2, 1.4 Hz, 1H), 2.25 (qd, *J* = 7.3, 1.0 Hz, 2H), 2.15 (q, *J* = 1.0 Hz, 3H), 1.54 (h, *J* = 7.4 Hz, 2H), 1.00 (t, *J* = 7.4 Hz, 3H).

^{13}C NMR (126 MHz, $CDCl_3$): δ = 141.4, 134.7, 133.7, 132.5, 129.4, 128.1, 127.7, 127.6, 126.1, 125.5, 124.6, 124.0, 31.2, 23.0, 16.0, 14.1.

HRMS *m/z* (GC-EI): calcd. For $C_{16}H_{18}$ ($[M]^+$): 210.140300; found: 210.140110.

(*Z*)-isomer:

^{1}H NMR (501 MHz, $CDCl_3$): δ = 7.86 – 7.78 (m, 3H), 7.63 (d, *J* = 1.7 Hz, 1H), 7.51 – 7.42 (m, 2H), 7.34 (dd, *J* = 8.4, 1.7 Hz, 1H), 5.56 (ddt, *J* = 7.4, 5.9, 1.5 Hz, 1H), 2.12 (q, *J* = 1.4 Hz, 3H), 2.01 (qd, *J* = 7.3, 1.3 Hz, 2H), 1.38 (h, *J* = 7.4 Hz, 2H), 0.85 (t, *J* = 7.4 Hz, 3H).

^{13}C NMR (126 MHz, $CDCl_3$): δ = 140.0, 136.2, 133.5, 132.3, 128.4, 128.0, 127.7, 127.6, 126.9, 126.6, 126.0, 125.6, 31.4, 25.8, 23.4, 14.0.

HRMS *m/z* (GC-EI): calcd. For $C_{16}H_{18}$ ($[M]^+$): 210.140300; found: 210.140400.

4. Asymmetric Reduction of Styrenes

Racemate Syntheses

For the racemate syntheses of compounds **2a–2e**, **2g**, **2l–2o**, **2q** the respective styrene (0.030 mmol, 1.00 eq.) was added to a GC vial filled with TFA (0.1 mL) and Et_3SiH (0.033 mmol, 1.10 eq.) and stirred at room temperature for 90 min to 24 h until TLC indicated full consumption of the starting material. Subsequent preparative thin-layer chromatography furnished the isolated racemic products that were used for the determination of the enantiomeric ratio by GC analysis.

For racemic compounds **2f**, **2h** and **2j** the respective styrene (0.030 mmol, 1.00 eq.) was added to a GC vial filled with $HNTf_2$ (0.2 M in DCM, 5 mol%), benzoic acid (0.036 mmol, 1.20 eq.), Et_3SiH (0.036 mmol, 1.20 eq.) and DCM (0.15 mL) and stirred at rt for 90 min to 24 h until TLC indicated full consumption of the starting material. Subsequent preparative thin-layer chromatography furnished the isolated racemic products that were used for the determination of the enantiomeric ratio by GC analysis.

For the racemate synthesis of compound **2k** the respective styrene (0.030 mmol, 1.00 eq.) was added to a GC vial filled with a 1:1 mixture of (*S*,*S*)- and (*R*,*R*)-IDPi **S1a** (2 mol%), benzoic acid (0.036 mmol, 1.20 eq.), $PhMe_2SiH$ (0.15 mmol, 5.0 eq.) and CyMe (0.06 mL) and stirred at rt for 24 h. Subsequent preparative thin-layer chromatography furnished the isolated racemic products that were used for the determination of the enantiomeric ratio by GC analysis.

To obtain racemic compound **2p** a literature procedure starting from 3-phenylbutanal was followed.[1]

For racemic compound **2r** the respective styrene (0.030 mmol, 1.00 eq.) was dissolved in dry THF (0.6 mL) in a GC vial. Pd/C (10%, 5 mol%) was added and the reaction mixture was stirred at room temperature under a H_2 atmosphere for 3 h. The mixture was filtered over Celite and concentrated to furnish the racemic product for subsequent GC analysis.

General Procedure 3 (GP3)

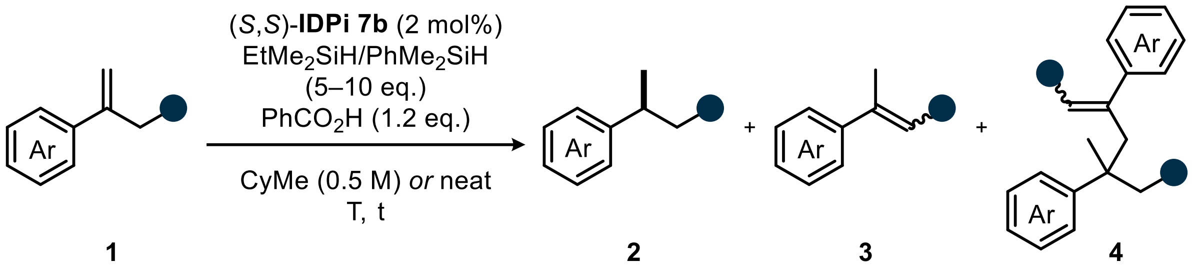

A 2 mL GC vial equipped with a magnetic stirring bar was charged with (*S*,*S*)-**IDPi 7b** (2 mol%), benzoic acid (0.24 mmol, 1.20 eq.) and then placed under argon. CyMe (0.4 mL) and the respective silane, $EtMe_2SiH$ or $PhMe_2SiH$ (1.0–2.0 mmol, 5.0–10 eq.), were added. After cooling the reaction mixture to –78 °C, the corresponding styrene (0.20 mmol, 1.00 eq.) was added via syringe. The cap was replaced under argon, and the reaction vial was stirred at the specified temperature for the indicated duration. The reaction was quenched by adding pyridine (5 µL), followed by the addition of dibromomethane (7 µL, 0.10 mmol, 0.50 eq.) as an internal standard. The composition of the reaction mixture was analyzed via ^1H NMR spectroscopy of an aliquot in C_6D_6. The crude reaction mixture was then directly purified by flash column chromatography on $AgNO_3$-impregnated silica gel.

For the spectral identification of desired product **2**, isomer **3**, and dimer **4** in the ^1H NMR spectra of the individual reaction crudes, these species were characterized in C_6D_6 using the model substrate hex-1-en-2-ylbenzene and subsequently assigned by analogy for other substrates.

hexan-2-ylbenzene (2a)

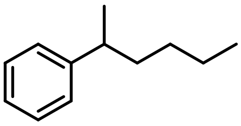

^1H NMR (501 MHz, C_6D_6): δ = 7.22 – 7.17 (m, 2H), 7.11 – 7.07 (m, 3H), 2.59 – 2.48 (m, 1H), 1.58 – 1.49 (m, 1H), 1.49 – 1.41 (m, 1H), 1.27 – 1.07 (m, 7H), 0.81 (t, *J* = 7.1 Hz, 3H).

^{13}C NMR (126 MHz, C_6D_6): δ = 148.0, 128.7, 127.3, 126.2, 40.4, 38.5, 30.3, 23.2, 22.7, 14.2.

HRMS *m/z* (GC-EI): calcd. For $C_{12}H_{18}$ ([M]$^+$): 162.140300; found: 162.140400.

hex-2-en-2-ylbenzene (3a)

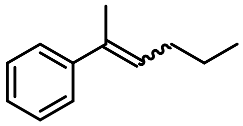

(*E*)-isomer:

^{1}H NMR (501 MHz, C_6D_6): δ = 7.37 – 7.33 (m, 2H), 7.21 – 7.17 (m, 2H), 7.13 – 7.08 (m, 1H), 5.79 (tq, *J* = 7.2, 1.4 Hz, 1H), 2.06 (qd, *J* = 7.2, 1.0 Hz, 2H), 1.91 (s, 3H), 1.43 – 1.34 (m, 2H), 0.89 (t, *J* = 7.3 Hz, 3H).

^{13}C NMR (126 MHz, C_6D_6): δ = 144.5, 135.4, 128.5, 128.5, 126.8, 126.1, 31.1, 23.2, 15.9, 14.1.

(*Z*)-isomer:

^{1}H NMR (501 MHz, C_6D_6): δ = 7.18 (d, *J* = 5.7 Hz, 4H), 7.12 – 7.05 (m, 1H), 5.45 (tq, *J* = 7.3, 1.4 Hz, 1H), 2.05 – 1.97 (m, 5H), 1.36 – 1.27 (m, 2H), 0.79 (t, *J* = 7.4 Hz, 3H).

^{13}C NMR (126 MHz, C_6D_6): δ = 142.7, 136.6, 128.4, 128.4, 126.8, 31.6, 25.8, 23.7, 14.0.

HRMS *m/z* (GC-EI): calcd. For $C_{12}H_{16}$ ($[M]^+$): 160.124650; found: 160.124810.

(7-methylundec-4-ene-5,7-diyl)dibenzene (4a)

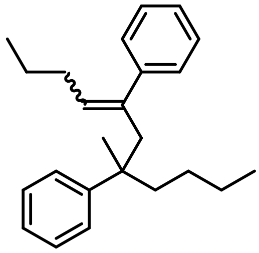

The dimer was isolated as a mixture of isomers with a ratio of *E*/*Z* ≈ 1:2.

^{1}H NMR (600 MHz, C_6D_6): δ = 7.24 – 7.21 (m, $2H_{min}$), 7.21 – 7.17 (m, $2H_{min}$, $2H_{maj}$), 7.15 – 7.08 (m, $4H_{min}$, $4H_{maj}$), 7.07 – 6.99 (m, $2H_{min}$, $4H_{maj}$), 5.57 (t, *J* = 7.2 Hz, $1H_{min}$), 5.26 (t, *J* = 7.4 Hz, $1H_{maj}$), 2.87 (d, *J* = 13.7 Hz, $1H_{min}$), 2.82 (d, *J* = 13.7 Hz, $1H_{min}$), 2.80 (d, *J* = 13.5 Hz, $1H_{maj}$), 2.72 (d, *J* = 13.5 Hz, $1H_{maj}$), 1.92 (q, *J* = 7.4 Hz, $2H_{maj}$), 1.91 – 1.81 (m, $2H_{min}$), 1.77 (td, *J* = 13.8, 4.2 Hz, $1H_{min}$), 1.68 (td, J = 13.8, 4.4 Hz, $1H_{maj}$), 1.49 – 1.41 (m, $1H_{min}$, $1H_{maj}$), 1.30 – 1.17 (m, $5H_{min}$, $5H_{maj}$), 1.15 – 0.99 (m, $3H_{min}$, $3H_{maj}$), 0.95 – 0.89 (m, $1H_{min}$, $1H_{maj}$), 0.84 (t, *J* = 7.4 Hz, $3H_{min}$), 0.74 (t, *J* = 7.2 Hz, $3H_{maj}$), 0.73 (t, *J* = 7.4 Hz, $3H_{maj}$), 0.72 (t, *J* = 7.2 Hz, $3H_{min}$).

^{13}C NMR (151 MHz, C_6D_6): δ = 148.0, 148.0, 146.7, 142.8, 138.7, 138.3, 133.9, 132.1, 129.1, 128.1, 128.0, 127.3, 127.0, 126.9, 126.3, 126.3, 125.7, 125.5, 53.2, 44.5, 43.1, 43.1, 42.5, 42.3, 31.6, 31.3, 26.9, 26.8, 24.6, 24.5, 23.8, 23.8, 23.6, 23.2, 14.2, 14.1, 14.1, 13.9.

HRMS *m/z* (GC-CI): calcd. For $C_{24}H_{36}N_1$ ($[M+NH_4]^+$): 338.284224; found: 338.284550.

Note: $AgNO_3$-impregnated silica gel was prepared by adding a solution of $AgNO_3$ (10 g in 10 mL of Millipore water) to 100 g of silica gel (Merck, 60 Å, 230−400 mesh, particle size 0.040−0.063 mm) suspended in 200 mL of Millipore water, yielding a 10% $AgNO_3$ on silica gel mixture. The suspension was shaken, and the water was evaporated under reduced pressure using a rotary evaporator at 55 °C in a dark environment. The resulting material was further dried in an oven at 100 °C for 48 hours.

(*S*)-hexan-2-ylbenzene (2a)

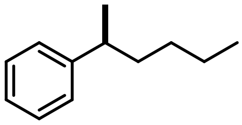

Prepared according to **GP3** using $EtMe_2SiH$ (10 eq.) under neat reaction conditions at –20 °C for 4 d, eluent: pentane, 14.5 mg (44%), colorless oil.

^1H NMR analysis of the crude: 76% conv., 67% **2a**, 3% **3a**, 3% **4a**.

$\boldsymbol{R_F}$ (100% hexanes) = 0.72.

^{1}H NMR (501 MHz, CD_2Cl_2): δ = 7.27 (t, *J* = 7.6 Hz, 2H), 7.20 – 7.13 (m, 3H), 2.67 (qt, *J* = 7.1, 7.1 Hz, 1H), 1.63 – 1.53 (m, 2H), 1.33 – 1.21 (m, 6H), 1.19 – 1.08 (m, 1H), 0.85 (t, *J* = 7.0 Hz, 2H).

^{13}C NMR (126 MHz, CD_2Cl_2): δ = 148.5, 128.6, 127.4, 126.1, 40.3, 38.5, 30.4, 23.2, 22.6, 14.2.

HRMS *m/z* (GC-EI): calcd. For $C_{12}H_{18}$ ($[M]^+$): 162.140300; found: 162.140400.

GC (30 m Cyclosil B, injection temperature: 220 °C, 90 °C iso 30 min, 1 °C/min, 100 °C iso 5 min, 8 °C/min, 220 °C iso 5 min, 0.5 bar H_2): t_{R1} = 38.6 min (major), t_{R2} = 39.5 min (minor), er = 95:5 (90% ee).

$[\boldsymbol{\alpha}]_{\boldsymbol{D}}^{\mathbf{25}}$ = +18.7 (c = 0.27, $CHCl_3$). Literature data for (*S*) enantiomer[2]: (89% ee) $[\boldsymbol{\alpha}]_{\boldsymbol{D}}^{\mathbf{20}}$ = +20.6 (c = 0.01, hexane).

(*S*)-1-(hexan-2-yl)-4-methylbenzene (2b)

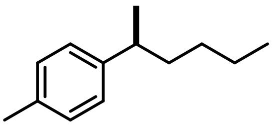

Prepared according to **GP3** using $EtMe_2SiH$ (10 eq.) under neat reaction conditions at –50 °C for 5 d, eluent: pentane, 21.8 mg (62%), colorless oil.

^{1}H NMR analysis of the crude: 98% conv., 76% **2b**, 1% **3b**, 10% **4b**.

***R*F** (100% hexanes) = 0.71.

^{1}H NMR (501 MHz, CD_2Cl_2): δ = 7.11 – 7.04 (m, 4H), 2.63 (qt, *J* = 7.0, 7.0 Hz, 1H), 2.30 (s, 3H), 1.58 – 1.50 (m, 2H), 1.33 – 1.18 (m, 6H), 1.16 – 1.06 (m, 1H), 0.85 (t, *J* = 7.2 Hz, 3H).

^{13}C NMR (126 MHz, CD_2Cl_2): δ = 145.4, 135.5, 129.2, 127.2, 39.9, 38.6, 30.4, 23.2, 22.7, 21.1, 14.2.

HRMS *m/z* (GC-EI): calcd. For $C_{13}H_{20}$ ($[M]^+$): 176.155950; found: 176.156030.

GC (30 m Cyclosil B, injection temperature: 220 °C, 90 °C iso 30 min, 1 °C/min, 100 °C iso 5 min, 8 °C/min, 220 °C iso 5 min, 0.5 bar H_2): t_{R1} = 51.1 min (major), t_{R2} = 51.4 min (minor), er = 93.5:6.5 (87% ee).

$[\alpha]_D^{25}$ = +18.9 (c = 0.27, $CHCl_3$).

(*S*)-1-(hexan-2-yl)-3-methylbenzene (2c)

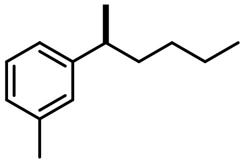

Prepared according to **GP3** using $EtMe_2SiH$ (5 eq.) in CyMe (0.5 M) at –20 °C for 3 d, eluent: pentane, 26.9 mg (76%), colorless oil.

^{1}H NMR analysis of the crude: 93% conv., 79% **2c**, 3% **3c**, 6% **4c**.

***R*F** (100% hexanes) = 0.72.

^{1}H NMR (501 MHz, CD_2Cl_2): δ = 7.15 (t, *J* = 7.5 Hz, 1H), 7.01 – 6.94 (m, 3H), 2.62 (qt, *J* = 7.1, 7.1 Hz, 1H), 2.32 (s, 3H), 1.58 – 1.52 (m, 2H), 1.33 – 1.17 (m, 6H), 1.17 – 1.08 (m, 1H), 0.85 (t, *J* = 7.1 Hz, 3H).

^{13}C NMR (126 MHz, CD_2Cl_2): δ = 148.5, 138.2, 128.5, 128.2, 126.8, 124.4, 40.3, 38.5, 30.4, 23.2, 22.6, 21.6, 14.2.

HRMS *m/z* (GC-EI): calcd. For $C_{13}H_{20}$ ($[M]^+$): 176.155950; found: 176.155980.

GC (25 m Lipodex-G, injection temperature: 220 °C, 80 °C iso 20 min, 8 °C/min, 220 °C iso 3 min, 0.6 bar H_2): t_{R1} = 16.7 min (minor), t_{R2} = 17.9 min (major), er = 96:4 (92% ee).

$[\alpha]_D^{25}$ = +19.0 (c = 0.26, $CHCl_3$).

(*S*)-1-ethyl-3-(hexan-2-yl)benzene (2d)

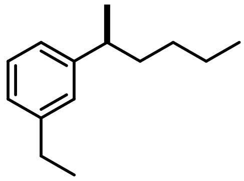

Prepared according to **GP3** using $EtMe_2SiH$ (10 eq.) under neat reaction conditions at –20 °C for 3 d, eluent: pentane, 26.5 mg (70%), colorless oil.

^{1}H NMR analysis of the crude: >99% conv., 95% **2d**, 4% **3d**, 1% **4d**.

$\boldsymbol{R}_\mathbf{F}$ (100% hexanes) = 0.72.

^{1}H NMR (501 MHz, CD_2Cl_2): δ = 7.18 (t, *J* = 7.4 Hz, 1H), 7.03 – 6.97 (m, 3H), 2.68 – 2.57 (m, 3H), 1.61 – 1.53 (m, 2H), 1.33 – 1.19 (m, 9H), 1.17 – 1.10 (m, 1H), 0.85 (t, *J* = 7.1 Hz, 3H).

^{13}C NMR (126 MHz, CD_2Cl_2): δ = 148.5, 144.7, 128.5, 127.1, 125.6, 124.6, 40.3, 38.5, 30.4, 29.3, 23.2, 22.6, 15.9, 14.2.

HRMS *m/z* (GC-EI): calcd. For $C_{14}H_{22}$ ($[M]^+$): 190.171600; found: 190.171460.

GC (25 m Lipodex-G, injection temperature: 220 °C, 75 °C iso 45 min, 8 °C/min, 220 °C iso 3 min, 0.6 bar H_2): t_{R1} = 35.6 min (minor), t_{R2} = 37.5 min (major), er = 96:4 (92% ee).

$[\alpha]_D^{25}$ = +18.1 (c = 0.38, $CHCl_3$).

(*S*)-1-(hexan-2-yl)-3,5-dimethylbenzene (2e)

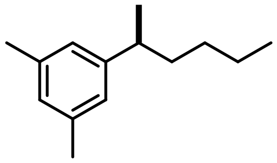

Prepared according to **GP3** using $EtMe_2SiH$ (5 eq.) in CyMe (0.5 M) at –20 °C for 3 d, eluent: pentane, 26.2 mg (69%), colorless oil.

^{1}H NMR analysis of the crude: 99% conv., 88% **2e**, 3% **3e**, 5% **4e**.

$\boldsymbol{R}_\mathbf{F}$ (100% hexanes) = 0.74.

^{1}H NMR (501 MHz, CD_2Cl_2): δ = 6.82 – 6.77 (m, 3H), 2.58 (qt, *J* = 7.1, 7.1 Hz, 1H), 2.27 (s, 6H), 1.59 – 1.49 (m, 2H), 1.33 – 1.09 (m, 7H), 0.86 (t, *J* = 7.2 Hz, 3H).

^{13}C NMR (126 MHz, CD_2Cl_2): δ = 148.4, 138.0, 127.6, 125.2, 40.2, 38.5, 30.5, 23.2, 22.6, 21.5, 14.2.

HRMS *m/z* (GC-EI): calcd. For $C_{14}H_{22}$ ($[M]^+$): 190.171600; found: 190.171750.

GC (25 m Lipodex-G, injection temperature: 220 °C, 70 °C iso 53 min, 8 °C/min, 220 °C iso 3 min, 0.6 bar H_2): t_{R1} = 45.7 min (minor), t_{R2} = 47.8 min (major), er = 95.5:4.5 (91% ee).

$[\alpha]_D^{25}$ = +17.7 (c = 0.37, $CHCl_3$).

(*S*)-1-fluoro-4-(hexan-2-yl)benzene (2f)

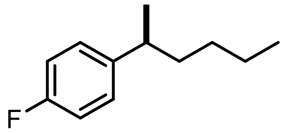

Prepared according to **GP3** using $PhMe_2SiH$ (5 eq.) in CyMe (0.5 M) at –20 °C for 3 d, eluent: pentane, 31.8 mg (89%), colorless oil.

^{1}H NMR analysis of the crude: 99% conv., 90% **2f**, 1% **3f**, 4% **4f**.

R

$\boldsymbol{R}_{\mathbf{F}}$ (100% hexanes) = 0.85.

^{1}H NMR (501 MHz, CD_2Cl_2): δ = 7.18 – 7.11 (m, 2H), 7.00 – 6.94 (m, 2H), 2.67 (qt, *J* = 7.1, 7.1 Hz, 1H), 1.59 – 1.49 (m, 2H), 1.33 – 1.16 (m, 6H), 1.15 – 1.06 (m, 1H), 0.85 (t, *J* = 7.2 Hz, 3H).

^{19}F{H} NMR (471 MHz, CD_2Cl_2): δ = -118.9.

^{13}C NMR (126 MHz, CD_2Cl_2): δ = 161.04 (d, *J* = 241.7 Hz), 143.76 (d, *J* = 3.1 Hz), 128.25 (d, *J* = 7.8 Hz), 114.67 (d, *J* = 20.9 Hz), 39.2, 38.1, 29.8, 22.7, 22.2, 13.7.

HRMS *m/z* (GC-EI): calcd. For $C_{12}H_{17}F$ ($[M]^+$): 180.130879; found: 180.130580.

GC (30 m Cyclosil B, injection temperature: 220 °C, 90 °C iso 30 min, 1 °C/min, 100 °C iso 5 min, 8 °C/min, 220 °C iso 5 min, 0.5 bar H_2): t_{R1} = 44.4 min (major), t_{R2} = 45.9 min (minor), er = 95.5:4.5 (91% ee).

$[\alpha]_D^{25}$ = +16.6 (c = 0.37, $CHCl_3$).

(*S*)-1-bromo-3-(hexan-2-yl)benzene (2g)

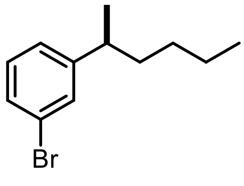

Prepared according to **GP3** using $PhMe_2SiH$ (10 eq.) under neat reaction conditions at 10 °C for 4 d, eluent: pentane, 34.5 mg (72%), colorless oil.

^{1}H NMR analysis of the crude: 99% conv., 83% **2g**, 17% **3g**, 0% **4g**.

$\boldsymbol{R}_{\mathbf{F}}$ (100% hexanes) = 0.79.

^{1}H NMR (501 MHz, CD_2Cl_2): δ = 7.34 (t, *J* = 1.8 Hz, 1H), 7.31 (ddd, *J* = 7.7, 2.0, 1.3 Hz, 1H), 7.19 – 7.11 (m, 2H), 2.65 (qt, *J* = 7.1, 7.1 Hz, 1H), 1.58 – 1.53 (m, 2H), 1.33 – 1.18 (m, 6H), 1.17 – 1.07 (m, 1H), 0.85 (t, *J* = 7.2 Hz, 3H).

^{13}C NMR (126 MHz, CD_2Cl_2): δ = 151.1, 130.5, 130.3, 129.1, 126.3, 122.6, 40.2, 38.3, 30.2, 23.1, 22.4, 14.2.

HRMS *m/z* (GC-EI): calcd. For $C_{12}H_{17}Br_1$ ($[M]^+$): 240.050825; found: 240.050860.

GC (25 m Lipodex-G, injection temperature: 220 °C, 85 °C iso 55 min, 8 °C/min, 220 °C iso 3 min, 0.6 bar H_2): t_{R1} = 47.6 min (minor), t_{R2} = 49.8 min (major), er = 95:5 (90% ee).

$[\alpha]_D^{25}$ = +18.2 (c = 0.39, $CHCl_3$).

(*S*)-1-(hexan-2-yl)-3-methoxybenzene (2h)

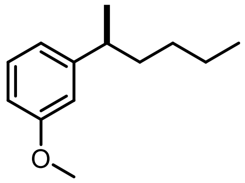

Prepared according to **GP3** using $PhMe_2SiH$ (5 eq.) in CyMe (0.5 M) at –20 °C for 3 d, eluent: pentane → 5% DCM, pentane, 29.8 mg (77%), colorless oil.

^{1}H NMR analysis of the crude: >99% conv., 98% **2h**, 2% **3h**, 0% **4h**.

***R*$_F$** (20% DCM, hexanes) = 0.38.

^{1}H NMR (501 MHz, CD_2Cl_2): δ = 7.19 (t, *J* = 7.8 Hz, 1H), 6.78 (dt, *J* = 7.6, 1.4 Hz, 1H), 6.74 – 6.68 (m, 2H), 3.78 (s, 3H), 2.64 (qt, *J* = 7.0, 7.0 Hz, 1H), 1.58 – 1.53 (m, 2H), 1.33 – 1.18 (m, 6H), 1.18 – 1.09 (m, 1H), 0.85 (t, *J* = 7.2 Hz, 3H).

^{13}C NMR (126 MHz, CD_2Cl_2): δ = 160.1, 150.3, 129.5, 119.8, 113.3, 111.1, 55.4, 40.4, 38.4, 30.4, 23.2, 22.5, 14.2.

HRMS *m/z* (GC-EI): calcd. For $C_{13}H_{20}O_1$ ($[M]^+$): 192.150865; found: 192.150990.

GC (25 m Lipodex-G, injection temperature: 220 °C, 90 °C iso 35 min, 8 °C/min, 220 °C iso 3 min, 0.6 bar H_2): t_{R1} = 30.4 min (minor), t_{R2} = 31.8 min (major), er = 97:3 (94% ee).

$[\alpha]_D^{25}$ = +20.3 (c = 0.33, $CHCl_3$).

(*S*)-2-(hexan-2-yl)naphthalene (2j)

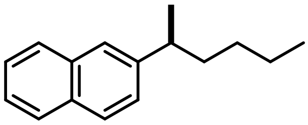

Prepared according to **GP3** using $EtMe_2SiH$ (10 eq.) under neat reaction conditions at –20 °C for 3 d, eluent: pentane, 32.1 mg (76%), colorless oil.

^{1}H NMR analysis of the crude: 99% conv., 82% **2j**, 1% **3j**, 9% **4j**.

$\boldsymbol{R_F}$ (100% hexanes) = 0.59.

^{1}H NMR (501 MHz, CD_2Cl_2): δ = 7.82 – 7.76 (m, 3H), 7.62 (d, *J* = 1.7 Hz, 1H), 7.43 (dddd, *J* = 17.7, 8.1, 6.8, 1.4 Hz, 2H), 7.37 (dd, *J* = 8.5, 1.8 Hz, 1H), 2.86 (qt, *J* = 7.0, 7.0 Hz, 1H), 1.73 – 1.60 (m, 2H), 1.35 – 1.23 (m, 6H), 1.21 – 1.10 (m, 1H), 0.85 (t, *J* = 7.1 Hz, 3H).

^{13}C NMR (126 MHz, CD_2Cl_2): δ = 146.0, 134.1, 132.6, 128.1, 127.9, 127.8, 126.3, 126.1, 125.5, 125.4, 40.5, 38.4, 30.4, 23.2, 22.5, 14.2.

HRMS *m/z* (GC-EI): calcd. For $C_{16}H_{20}$ ($[M]^+$): 212.155950; found: 212.155840.

GC (24 m Cyclodextrin-H, injection temperature: 220 °C, 100 °C iso 112 min, 8 °C/min, 180 °C iso 3 min, 0.5 bar H_2): t_{R1} = 98.8 min (major), t_{R2} = 104.1 min (minor), er = 98:2 (96% ee).

$[\boldsymbol{\alpha}]_{\boldsymbol{D}}^{\mathbf{25}}$ = +29.7 (c = 0.17, $CHCl_3$).

(*S*)-5-(hexan-2-yl)benzofuran (2k)

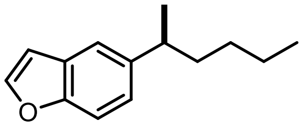

Prepared according to **GP3** using $EtMe_2SiH$ (10 eq.) under neat reaction conditions at –20 °C for 3 d, eluent: pentane, 25.8 mg (63%), colorless oil.

^{1}H NMR analysis of the crude: >99% conv., 80% **2k**, 0% **3k**, 11% **4k**.

$\boldsymbol{R_F}$ (100% hexanes) = 0.50.

^{1}H NMR (501 MHz, CD_2Cl_2): δ = 7.61 (d, *J* = 2.2 Hz, 1H), 7.43 – 7.37 (m, 2H), 7.13 (dd, *J* = 8.5, 1.8 Hz, 1H), 6.74 (dd, *J* = 2.2, 1.0 Hz, 1H), 2.78 (qt, *J* = 7.1, 7.1 Hz, 1H), 1.61 (q, *J* = 7.3 Hz, 2H), 1.32 – 1.20 (m, 6H), 1.16 – 1.08 (m, 1H), 0.84 (t, *J* = 7.2 Hz, 3H).

^{13}C NMR (126 MHz, CD_2Cl_2): δ = 154.0, 145.5, 143.1, 127.8, 124.0, 119.4, 111.1, 106.9, 40.3, 39.0, 30.4, 23.2, 23.1, 14.2.

HRMS *m/z* (GC-EI): calcd. For $C_{14}H_{18}O$ ($[M]^+$): 202.135215; found: 202.135430.

GC (24 m Cyclodextrin-H, injection temperature: 220 °C, 100 °C iso 45 min, 8 °C/min, 180 °C iso 3 min, 0.5 bar H_2): t_{R1} = 34.0 min (major), t_{R2} = 38.7 min (minor), er = 97:3 (94% ee).

$[\boldsymbol{\alpha}]_D^{25}$ = +20.4 (c = 0.28, $CHCl_3$).

(*S*)-heptan-2-ylbenzene (2l)

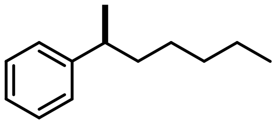

Prepared according to **GP3** using $EtMe_2SiH$ (10 eq.) under neat reaction conditions at –20 °C for 4 d, eluent: pentane, 21.9 mg (62%), colorless oil.

^{1}H NMR analysis of the crude: 92% conv., 86% **2l**, 3% **3l**, 2% **4l**.

***R*F** (100% hexanes) = 0.69.

^{1}H NMR (501 MHz, CD_2Cl_2): δ = 7.30 – 7.25 (m, 2H), 7.20 – 7.13 (m, 3H), 2.67 (qt, *J* = 7.1, 7.1 Hz, 1H), 1.62 – 1.50 (m, 2H), 1.30 – 1.20 (m, 8H), 1.20 – 1.07 (m, 1H), 0.92 – 0.80 (m, 3H).

^{13}C NMR (126 MHz, CD_2Cl_2): δ = 148.5, 128.6, 127.4, 126.1, 40.3, 38.8, 32.4, 27.8, 23.0, 22.6, 14.2.

HRMS *m/z* (GC-EI): calcd. For $C_{13}H_{20}$ ($[M]^+$): 176.1155950; found: 176.156030.

GC (29.5 m BGB-178/BGB-15, injection temperature: 220 °C, 65 °C iso 142 min, 8 °C/min, 230 °C iso 3 min, 0.6 bar H_2): t_{R1} = 128.4 min (major), t_{R2} = 133.3 min (minor), er = 95:5 (90% ee).

$[\boldsymbol{\alpha}]_D^{25}$ = +19.0 (c = 0.30, $CHCl_3$). Literature data for (*R*) enantiomer[3]: (99% ee) $[\boldsymbol{\alpha}]_D^{20}$ = –7.0 (c = 1.25, $CHCl_3$).

(*S*)-octan-2-ylbenzene (2m)

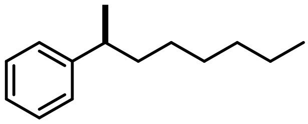

Prepared according to **GP3** using $EtMe_2SiH$ (10 eq.) under neat reaction conditions at –20 °C for 4 d, eluent: pentane, 28.2 mg (75%), colorless oil.

^{1}H NMR analysis of the crude: 94% conv., 84% **2m**, 3% **3m**, 3% **4m**.

***R*F** (100% hexanes) = 0.69.

^{1}H NMR (501 MHz, CD_2Cl_2): δ = 7.30 – 7.24 (m, 2H), 7.20 – 7.13 (m, 3H), 2.67 (qt, *J* = 7.1, 7.1 Hz, 1H), 1.61 – 1.53 (m, 2H), 1.30 – 1.18 (m, 10H), 1.18 – 1.10 (m, 1H), 0.86 (t, *J* = 7.0 Hz, 3H).

^{13}C NMR (126 MHz, CD_2Cl_2): δ = 148.6, 128.6, 127.4, 126.1, 40.3, 38.8, 32.2, 29.8, 28.1, 23.1, 22.6, 14.3.

HRMS *m/z* (GC-EI): calcd. For $C_{14}H_{22}$ ($[M]^+$): 190.171600; found: 190.171700.

GC (24 m Cyclodextrin-H, injection temperature: 220 °C, 90 °C iso 25 min, 8 °C/min, 180 °C, 0.5 bar H_2): t_{R1} = 19.0 min (major), t_{R2} = 20.5 min (minor), er = 95.5:4.5 (91% ee).

$[\alpha]_D^{25}$ = +20.3 (c = 0.39, $CHCl_3$).

(*S*)-pentan-2-ylbenzene (2n)

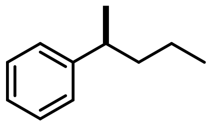

Prepared according to **GP3** using $EtMe_2SiH$ (10 eq.) under neat reaction conditions at –20 °C for 4 d, eluent: pentane, 7.2 mg (25%), colorless oil.

^{1}H NMR analysis of the crude: 79% conv., 68% **2n**, 4% **3n**, 3% **4n**.

$\boldsymbol{R}_{\mathbf{F}}$ (100% hexanes) = 0.69.

^{1}H NMR (501 MHz, CD_2Cl_2): δ = 7.30 – 7.24 (m, 2H), 7.20 – 7.13 (m, 3H), 2.69 (qt, *J* = 7.0, 7.0 Hz, 1H), 1.59 – 1.49 (m, 2H), 1.29 – 1.14 (m, 5H), 0.87 (t, *J* = 7.4 Hz, 3H).

^{13}C NMR (126 MHz, CD_2Cl_2): δ = 148.5, 128.6, 127.4, 126.1, 41.0, 40.0, 22.5, 21.2, 14.3.

HRMS *m/z* (GC-EI): calcd. For $C_{11}H_{16}$ ($[M]^+$): 148.124650; found: 148.124700.

GC (30 m Cyclosil B, injection temperature: 220 °C, 80 °C iso 30 min, 1 °C/min, 95 °C iso 5 min, 8 °C/min, 220 °C iso 5 min, 0.5 bar H_2): t_{R1} = 34.4 min (major), t_{R2} = 35.2 min (minor), er = 94.5:5.5 (89% ee).

$[\alpha]_D^{25}$ = +11.3 (c = 0.11, $CHCl_3$). Literature data for (*S*) enantiomer[4]: (2.5% ee) $[\alpha]_D^{20}$ = +0.49 (c = 3.51, hexane).

(*S*)-5-methyl-6,7,8,9-tetrahydro-5*H*-benzo[7]annulene (2o)

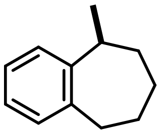

Prepared according to **GP3** using $PhMe_2SiH$ (5 eq.) in CyMe (0.5 M) at –20 °C for 4 d, eluent: pentane, 20.3 mg (63%), colorless oil.

^1H NMR analysis of the crude: >99% conv., 64% **2o**, 36% **3o**, 0% **4o**.

$\boldsymbol{R_F}$ (100% hexanes) = 0.65.

^{1}H NMR (501 MHz, CD_2Cl_2): δ = 7.17 (dd, *J* = 7.6, 1.2 Hz, 1H), 7.14 – 7.09 (m, 1H), 7.08 – 7.03 (m, 2H), 3.09 – 3.00 (m, 1H), 2.92 – 2.83 (m, 1H), 2.83 – 2.74 (m, 1H), 1.95 – 1.87 (m, 1H), 1.80 – 1.71 (m, 2H), 1.47 – 1.32 (m, 5H), 1.30 – 1.21 (m, 1H).

^{13}C NMR (126 MHz, CD_2Cl_2): δ = 147.1, 143.3, 129.6, 126.3, 126.0, 36.6, 36.5, 30.1, 28.4, 20.6.

HRMS *m/z* (GC-EI): calcd. For $C_{12}H_{16}$ ($[M]^+$): 160.124650; found: 160.124930.

GC (30 m Cyclosil B, injection temperature: 220 °C, 90 °C iso 30 min, 1 °C/min, 100 °C iso 5 min, 8 °C/min, 220 °C iso 5 min, 0.5 bar H_2): t_{R1} = 55.0 min (minor), t_{R2} = 55.6 min (major), er = 92.5:7.5 (85% ee).

$[\boldsymbol{\alpha}]_{\boldsymbol{D}}^{\mathbf{25}}$ = –6.9 (c = 0.20, $CHCl_3$). Literature data for (*S*) enantiomer[5]: (85% ee) $[\boldsymbol{\alpha}]_{\boldsymbol{D}}^{\mathbf{23}}$ = –9.4 (c = 5, $CHCl_3$).

(*S*)-hept-6-en-2-ylbenzene (2p)

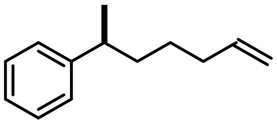

Prepared according to **GP3** using $PhMe_2SiH$ (5 eq.) in CyMe (0.5 M) at –20 °C for 4 d, eluent: pentane, 2.8 mg (8%), colorless oil.

Note: The reaction resulted in the formation of a cyclized side product (1-methyl-1,2,3,4-tetrahydro-1,1'-biphenyl)[6] that could not be separated from the desired compound. Consequently, the isolated sample contains approximately 10% of the cyclized species. The optical rotation reported below corresponds to this mixture.

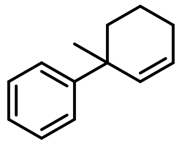

^1H NMR analysis of the crude: >99% conv., 45% **2p**.

$\boldsymbol{R_F}$ (100% hexanes) = 0.65.

^{1}H NMR (501 MHz, CD_2Cl_2): δ = 7.32 – 7.26 (m, 2H), 7.22 – 7.15 (m, 3H), 5.79 (ddt, *J* = 16.9, 10.2, 6.7 Hz, 1H), 4.98 (ddt, *J* = 17.1, 1.7, 1.7 Hz, 1H), 4.92 (ddt, *J* = 10.2, 2.3, 1.2 Hz, 1H), 2.70 (qt, *J* = 7.0, 7.0 Hz, 1H), 2.11 – 1.98 (m, 2H), 1.65 – 1.55 (m, 2H), 1.41 – 1.30 (m, 1H), 1.30 – 1.23 (m, 4H).

^{1}H NMR (501 MHz, C_6D_6): δ = 7.21 – 7.17 (m, 2H), 7.11 – 7.05 (m, 3H), 5.69 (ddt, *J* = 17.0, 10.2, 6.7 Hz, 1H), 5.02 – 4.91 (m, 2H), 2.56 – 2.46 (m, 1H), 1.96 – 1.85 (m, 2H), 1.55 – 1.37 (m, 2H), 1.31 – 1.16 (m, 2H), 1.14 (d, *J* = 6.9 Hz, 3H).

^{13}C NMR (126 MHz, CD_2Cl_2): δ = 148.3, 139.5, 128.7, 127.4, 126.2, 114.4, 40.3, 38.3, 34.2, 27.5, 22.5.

HRMS *m/z* (API-ES): calcd. For $C_{13}H_{18}$ ($[M]^+$): 174.140300; found: 174.140330.

GC (30 m Cyclosil B, injection temperature: 220 °C, 110 °C iso 60 min, 8 °C/min, 220 °C iso 5 min, 0.5 bar H_2): t_{R1} = 33.6 min (major), t_{R2} = 34.3 min (minor), er = 93:7 (86% ee).

$[\alpha]_D^{25}$ = +14.3 (c = 0.14, $CHCl_3$).

methyl (*S*)-5-phenylhexanoate (2q)

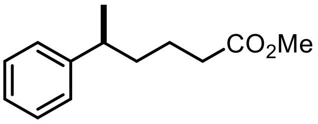

Prepared according to **GP3** using $PhMe_2SiH$ (5 eq.) in CyMe (0.5 M) at –20 °C for 4 d, eluent: pentane → 3% MTBE, pentane, 30.0 mg (72%), colorless oil.

^{1}H NMR analysis of the crude: >99% conv., 84% **2q**, 4% **3q**, 8% **4q**.

***R*$_F$** (5% EtOAc, hexanes) = 0.49.

^{1}H NMR (501 MHz, CD_2Cl_2): δ = 7.31 – 7.25 (m, 2H), 7.20 – 7.15 (m, 3H), 3.61 (s, 3H), 2.69 (qt, *J* = 7.0, 7.0 Hz, 1H), 2.28 – 2.23 (m, 2H), 1.63 – 1.51 (m, 4H), 1.51 – 1.40 (m, 1H), 1.23 (d, *J* = 7.0 Hz, 3H).

^{13}C NMR (126 MHz, CD_2Cl_2): δ = 174.2, 147.8, 128.7, 127.4, 126.3, 51.6, 40.1, 38.0, 34.4, 23.6, 22.5.

HRMS *m/z* (API-ES): calcd. For $C_{13}H_{18}O_2Na_1$ ($[M+Na]^+$): 229.119899; found: 229.119960.

GC (25 m Ivadex-7/PS086, injection temperature: 220 °C, 100 °C iso 62 min, 8 °C/min, 220 °C, 0.6 bar H_2): t_{R1} = 53.4 min (major), t_{R2} = 56.8 min (minor), er = 93:7 (86% ee).

$[\alpha]_D^{25}$ = +14.8 (c = 0.26, $CHCl_3$).

(*S*)-(6-chlorohexan-2-yl)benzene (2r)

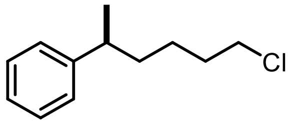

Prepared according to **GP3** using $PhMe_2SiH$ (5 eq.) in CyMe (0.5 M) at –20 °C for 4 d, eluent: pentane → 1% MTBE, pentane, 29.7 mg (76%), colorless oil.

^{1}H NMR analysis of the crude: >99% conv., 85% **2r**, 6% **3r**, 5% **4r**.

***R*$_F$** (100% hexanes) = 0.41.

^{1}H NMR (501 MHz, CD_2Cl_2): δ = 7.31 – 7.26 (m, 2H), 7.20 – 7.15 (m, 3H), 3.49 (t, *J* = 6.8 Hz, 2H), 2.69 (qt, *J* = 7.1, 7.1 Hz, 1H), 1.81 – 1.64 (m, 2H), 1.66 – 1.56 (m, 2H), 1.43 – 1.33 (m, 1H), 1.31 – 1.25 (m, 2H), 1.23 (d, *J* = 6.9 Hz, 3H).

^{13}C NMR (126 MHz, CD_2Cl_2): δ = 148.0, 128.7, 127.4, 126.2, 45.6, 40.2, 37.9, 33.1, 25.5, 22.5.

HRMS *m/z* (GC-EI): calcd. For $C_{12}H_{17}Cl_1$ ($[M]^+$): 196.101328; found: 196.101320.

GC (25 m Hydrodex-beta-TBDAc-CD, injection temperature: 220 °C, 90 °C iso 220 min, 8 °C/min, 220 °C iso 3 min, 0.6 bar H_2): t_{R1} = 194.2 min (major), t_{R2} = 204.2 min (minor), er = 90:10 (80% ee).

$[\alpha]_D^{25}$ = +17.4 (c = 0.25, $CHCl_3$).

5. Limitations of the method

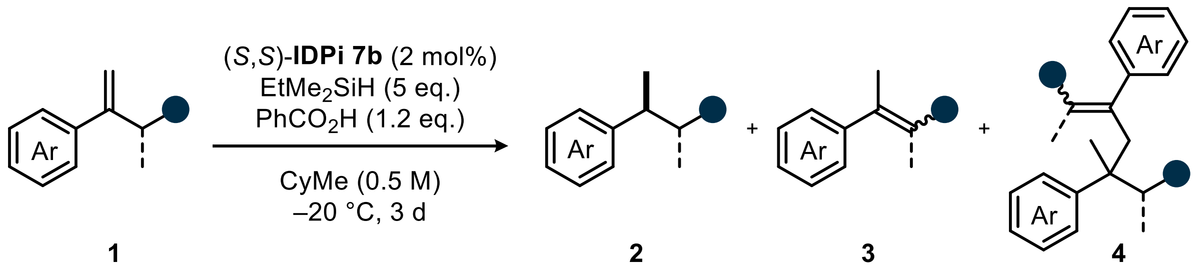

A 2 mL GC vial equipped with a magnetic stirring bar was charged with (*S*,*S*)-**IDPI 7b** (2 mol%), benzoic acid (0.030 mmol, 1.2 eq.) and then placed under argon. CyMe (0.05 mL) and $EtMe_2SiH$ (0.125 mmol 5.0 eq.), were added. After cooling the reaction mixture to –78 °C, the corresponding styrene (0.025 mmol, 1.0 eq.) was added via syringe. The cap was replaced under argon, and the reaction vial was stirred at –20 °C for 3 d. The reaction was quenched by adding pyridine (5 µL), followed by the addition of mesitylene (3 µL, 0.022 mmol, 0.86 eq.) as an internal standard. The composition of the reaction mixture was analyzed via ^{1}H NMR spectroscopy of an aliquot in C_6D_6. The crude reaction mixture was then directly purified by preparative thin layer chromatography to furnish the chiral product. Chiral GC analysis was performed to give the corresponding enantiomeric ratio.

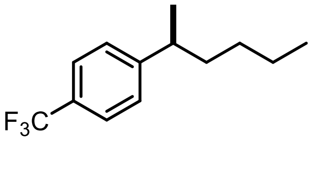

42% conv.
42% **3r**

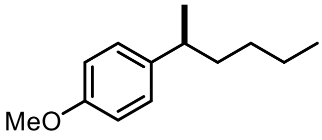

100% conv.[c]
44% **2s**, 75.5:24.5 er
0% **3s**, 19% **4s**

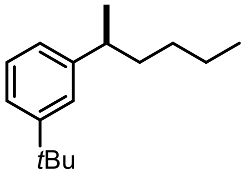

100% conv.[a]
84% **2t**, 82:18 er
3% **3t**, 9% **4t**

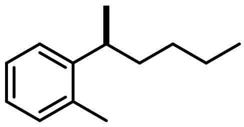

35% conv.[a,b]
35% **3u**

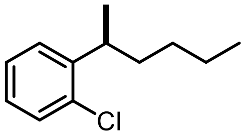

no reaction[c]

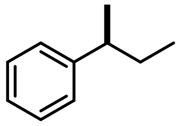

19% conv.[c]
16% **2v**, 64.5:35.5 er
0% **3v**, 1% **4v**

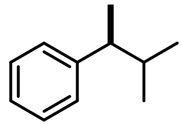

50% conv.[b,c]
28% **2w**, 65.5:34.5 er

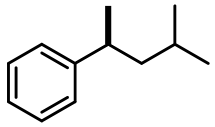

no reaction[b]

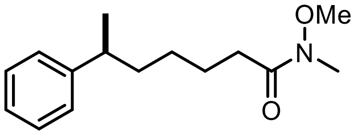

no reaction

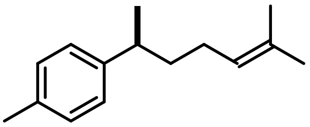

96% conv.[c,d]
65% **2x**, 78:22 er
2% **3x**, 11% **4x**

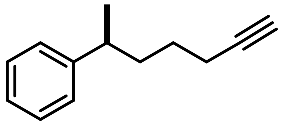

no reaction[c]

from aliphatic 1,1-disubstituted alkene:

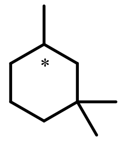

<5% **2y**
≤57:43 er

***Figure S3:** Representative limitations of the method. a: 5 eq. $PhMe_2SiH$ used. b: at room temperature. c: 10 eq. $EtMe_2SiH$ used under neat reaction conditions. d: at –30 °C.*

6. Scale-Up Experiment and Derivatization for Absolute Configuration Assignment

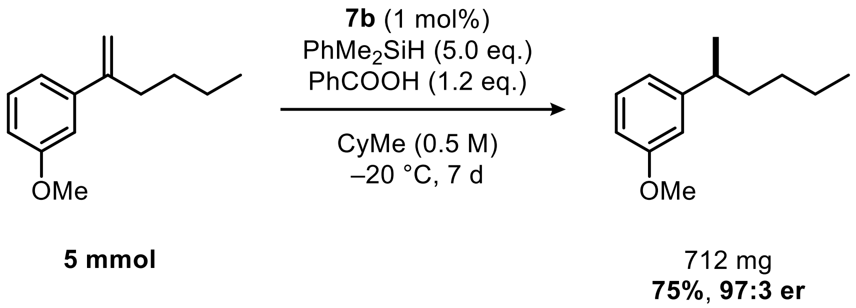

An oven-dried Schlenk flask under argon atmosphere was charged with IDPi **7b** (106.5 mg, 1 mol%), benzoic acid (722 mg, 5.91 mmol, 1.19 eq.) , and CyMe (9.9 mL), then cooled to –20 °C. $PhMe_2SiH$ (3.8 mL, 25 mmol, 5.0 eq.) was added, followed by the dropwise addition of styrene **1h** (945 mg, 4.97 mmol, 1.00 eq.). The reaction mixture was stirred at –20 °C for 7 d, and then quenched by adding pyridine (40 μL) while maintaining –20 °C for an additional 15 min. The crude mixture was directly loaded onto a silica column. The product **2h** was eluted first (100% pentane to 5% DCM, pentane) and obtained as a colorless oil (712 mg, 75%, 97:3 er), followed by the catalyst (100% DCM). To remove residual benzoic acid, a basic extraction with 3% NaOH and DCM was performed. Acidification via filtration over DOWEX 50WX8 (H-form, eluted with DCM) furnished the recovered catalyst **7b** (94 mg, 88%) as a beige solid.

Derivatization of 2h

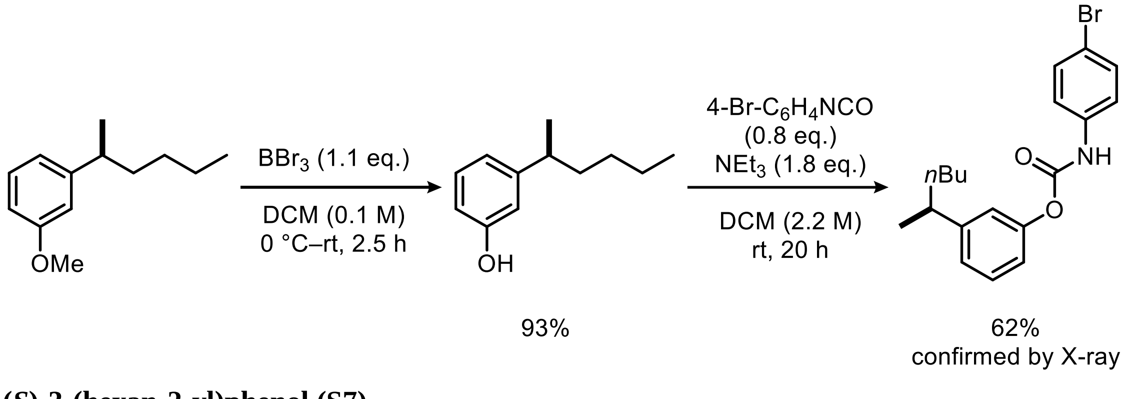

(*S*)-3-(hexan-2-yl)phenol (S7)

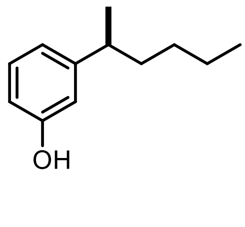

In a flame-dried Schlenk flask under argon, **2h** (449 mg, 2.33 mmol, 1.00 eq.) was dissolved in dry DCM (20 mL) and cooled to 0 °C. BBr_3 (1 M in DCM, 2.6 mL, 2.6 mmol, 1.11 eq.) was added dropwise and the resulting solution was then stirred at room temperature for 2.5 h until TLC

indicated full consumption of the starting material. The reaction was quenched by adding water (20 mL) at 0 °C. The aqueous layer was subsequently extracted with DCM (2 x 20 mL), the combined organic phases were washed with water, dried over anhydrous Na_2SO_4, and concentrated under reduced pressure. Purification via flash column chromatography on silica gel (5–10% EtOAc, pentane) furnished the title compound **S7** (386 mg, 93%) as a light yellow oil.

^{1}H NMR (501 MHz, CD_2Cl_2): δ = 7.13 (t, *J* = 7.8 Hz, 1H), 6.75 (dt, *J* = 7.5, 1.3 Hz, 1H), 6.66 (t, *J* = 2.1 Hz, 1H), 6.63 (ddd, *J* = 8.0, 2.6, 1.0 Hz, 1H), 4.83 (s, 1H), 2.62 (qt, *J* = 7.0, 7.0 Hz, 1H), 1.57 – 1.49 (m, 2H), 1.34 – 1.18 (m, 6H), 1.18 – 1.07 (m, 1H), 0.85 (t, *J* = 7.2 Hz, 3H).

^{13}C NMR (126 MHz, CD_2Cl_2) δ 156.1, 150.6, 129.7, 119.9, 114.2, 112.9, 40.2, 38.4, 30.3, 23.2, 22.5, 14.2.

HRMS *m/z* (GC-EI): calcd. For $C_{12}H_{18}O_1$ ($[M]^+$): 178.135215; found: 178.135460.

(*S*)-3-(hexan-2-yl)phenyl (4-bromophenyl)carbamate (2i)

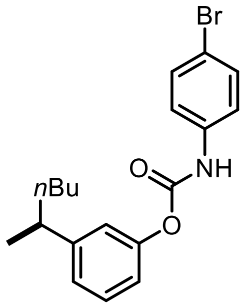

A GC vial was charged with phenol **S7** (47.5 mg, 0.266 mmol, 1.25 eq.), DCM (0.1 mL) and NEt_3 (54 µL, 0.39 mmol, 1.8 eq.). 4-Bromophenylisocyanate (42.2 mg, 0.213 mmol, 1.00 eq.) was added, and the resulting mixture was stirred at room temperature for 20 h. The crude material was directly loaded onto a silica column and eluted (2–10% EtOAc, pentane) to furnish carbamate **2i** (50.0 mg, 62%) as a white solid.

^{1}H NMR (501 MHz, CD_2Cl_2): δ = 7.48 – 7.43 (m, 2H), 7.40 – 7.34 (m, 2H), 7.34 – 7.29 (m, 1H), 7.14 – 7.08 (m, 2H), 7.04 – 6.98 (m, 2H), 2.71 (qt, *J* = 7.0, 7.0 Hz, 1H), 1.65 – 1.50 (m, 2H), 1.36 – 1.22 (m, 6H), 1.22 – 1.10 (m, 1H), 0.87 (t, *J* = 7.1 Hz, 3H).

^{13}C NMR (126 MHz, CD_2Cl_2) δ 152.0, 151.0, 150.5, 137.3, 132.4, 129.5, 125.0, 120.5, 119.3, 40.2, 38.4, 30.3, 23.1, 22.3, 14.2.

HRMS *m/z* (GC-EI): calcd. For $C_{19}H_{22}O_2N_1Na_1Br_1$ ($[M+Na]^+$): 398.072623; found: 398.072280.

Recrystallization for X-ray crystallography: The white solid was suspended in hexanes and heated to reflux. Additional hexanes were added dropwise until complete dissolution. The solution was then allowed to cool slowly to room temperature. crystals suitable for X-ray analysis were obtained, and the (*S*)-configuration was assigned based on the data.

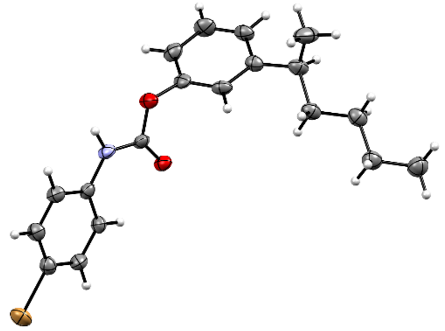

Validation of Absolute Configuration through Comparison with Reported $[\alpha]_D^{25}$ Values

The measured $[\alpha]_D^{25}$ values for products **2a**, **2n**, and **2o** align with literature values for the corresponding (*S*)-enantiomers. For substrate **2l**, the literature reports the specific optical rotation of the (*R*)-enantiomer, which is opposite to the value measured in this work for the (*S*)-enantiomer (see characterization in Chapter 3).

For **2b**, literature data contradict our assigned (*S*)-configuration:

$[\alpha]_D^{25}$ = +18.9 (c = 0.27, $CHCl_3$, 87% ee). Literature data for (*R*) enantiomer[7]: (85% ee) $[\alpha]_D^{24}$ = +4.9 (c = 1.0, $CHCl_3$).

However, based on our X-Ray analysis and consistent optical rotation measurements across four compounds, we conclude that our assignment is correct.

7. Synthesis of IDPi Catalysts

2-bromobenzo[*b*]thieno[3,2-*d*]thiophene (S8)

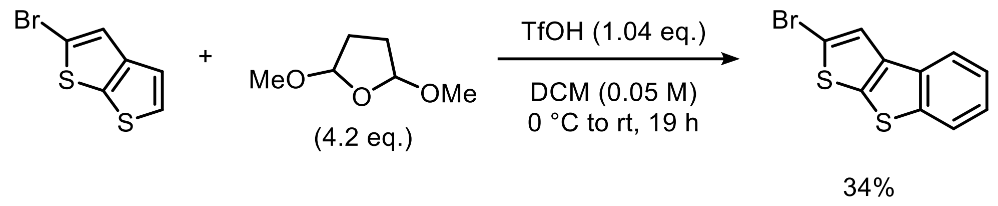

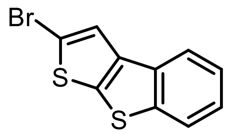

The synthesis was carried out following a modified procedure reported by Rafiq *et al.*[8] In a flame-dried Schlenk flask under argon, 2-bromothieno[2,3-*b*]thiophene (1.24 g, 5.65 mmol, 1.00 eq.) was dissolved in dry DCM (115 mL) and 2,5-dimethoxytetrahydrofuran (1.6 mL, 12.4 mmol, 2.20 eq.). The solution was cooled to 0 °C, and TfOH (0.52 mL, 5.88 mmol, 1.04 eq.) was added dropwise. After stirring for 15 min at 0 °C, the reaction mixture was warmed to room temperature and stirred for an additional 2.5 h. 2,5-Dimethoxyfuran (1.5 mL, 11.3 mmol, 2.00 eq.) was then added, and the reaction was stirred for a further 16 h. The reaction was quenched by the addition of aqueous $NaHCO_3$ solution (60 mL). The aqueous layer was extracted with DCM (3 × 40 mL), and the combined organic layers were washed with water, dried over anhydrous Na_2SO_4, and concentrated under reduced pressure. Purification by flash column chromatography on silica gel (100% hexanes) afforded the title compound (516 mg, 5.65 mmol, 34%) as a white solid.

R$_F$ (100% hexanes) = 0.61.

^{1}H NMR (501 MHz, CD_2Cl_2): δ = 7.89 (d, *J* = 7.8 Hz, 1H), 7.84 (d, *J* = 8.0 Hz, 1H), 7.57 (s, 1H), 7.46 – 7.41 (m, 1H), 7.37 (ddd, *J* = 8.4, 7.2, 1.3 Hz, 1H).

^{13}C NMR (126 MHz, CD_2Cl_2): δ = 143.2, 141.3, 137.3, 132.5, 125.2, 125.0, 123.5, 122.9, 121.9, 112.9.

HRMS *m*/*z* (GC-EI): calcd. for $C_{10}H_5S_2Br_1$ ($[M]^+$): 267.901070; found: 267.901440.

2-bromo-6-(trifluoromethyl)benzo[*b*]thieno[3,2-*d*]thiophene (S9)

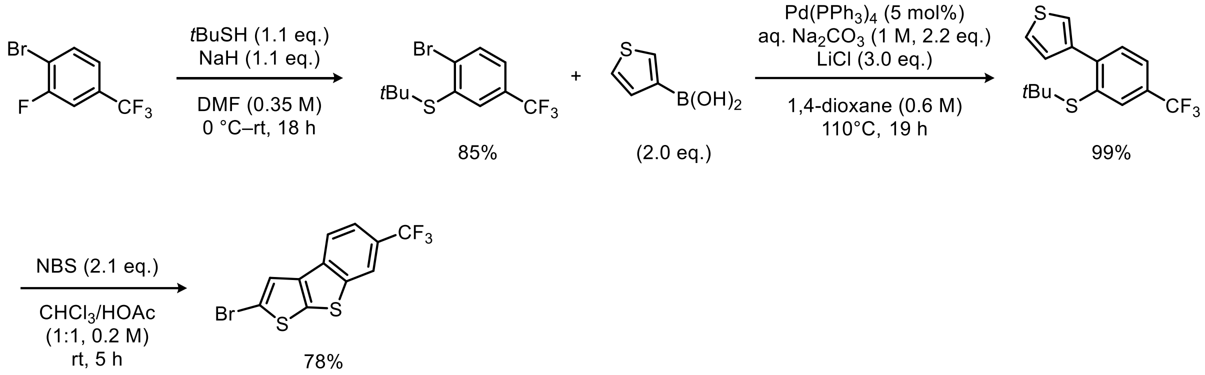

(2-bromo-5-(trifluoromethyl)phenyl)(tert-butyl)sulfane (S10)

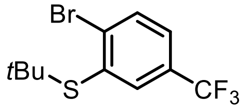

A flame-dried Schlenk flask under argon was charged with NaH (60% in mineral oil, 905 mg, 22.6 mmol, 1.10 eq.) and dry DMF (60 mL), and cooled to 0 °C. After dropwise addition of *tert*-butylthiol (2.5 mL, 23 mmol, 1.1 eq.) the reaction mixture was stirred for 1 h. 1-Bromo-2-fluoro-4-(trifluoromethyl)benzene (4.99 g, 20.5 mmol, 1.00 eq.) was added in one portion at 0 °C. The reaction was allowed to warm to room temperature and stirred for 18 h until TLC indicated full consumption of the starting material. It was then diluted with MTBE and quenched with 10% HCl. The aqueous layer was extracted with MTBE (2 x 100 mL), and the combined organic phases were washed with brine (4 x 100 mL), dried over anhydrous Na_2SO_4 and concentrated under reduced pressure. Purification via flash column chromatography on silica gel (100% hexanes) furnished the title compound as a colorless oil (5.44 g, 85%).

$\boldsymbol{R}_F$ (100% hexanes) = 0.51.

^{1}H NMR (501 MHz, CD_2Cl_2): δ = 7.91 (dd, *J* = 2.3, 0.8 Hz, 1H), 7.84 (dd, *J* = 8.4, 0.9 Hz, 1H), 7.45 (ddd, *J* = 8.4, 2.4, 0.8 Hz, 1H), 1.36 (s, 9H).

^{19}F NMR (471 MHz, CD_2Cl_2): δ = –63.2 (s, 3F).

^{13}C NMR (151 MHz, CD_2Cl_2): δ = 136.9 (q, *J* = 1.5 Hz), 136.4, 135.9 (q, *J* = 3.8 Hz), 134.5, 130.0 (q, *J* = 32.8 Hz), 126.9 (q, *J* = 3.6 Hz), 124.1 (q, *J* = 271.9 Hz), 49.6, 31.2.

HRMS *m/z* (GC-EI): calcd. For $C_{11}H_{12}S_1F_3Br_1$ ($[M]^+$): 311.978983; found: 311.979340.

3-(2-(tert-butylthio)-4-(trifluoromethyl)phenyl)thiophene (S11)

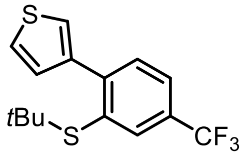

A Schlenk flask was charged with (2-bromo-5-(trifluoromethyl)phenyl)(tert-butyl)sulfane (5.27 g, 16.8 mmol, 1.00 eq.), 3-thienylboronic acid (4.31 g, 33.7 mmol, 2.00 eq.), LiCl (2.14 g, 50.5 mmol, 3.00 eq.), aqueous Na_2CO_3 (2 M, 18.5 mL, 37.0 mmol, 2.20 eq.) and 1,4-dioxane (28 mL). Subsequently the suspension was sparged with argon for 30 min, $Pd(PPh_3)_4$ (5 mol%, 970 mg) was added under argon, and the mixture was heated to 110 °C for 19 h. After addition of water the aqueous phase was extracted with DCM (3 x 75 mL). The combined organic layers were washed with water, dried over anhydrous Na_2SO_4 and concentrated under reduced pressure. Purification via flash column chromatography on silica gel (100% hexanes) furnished the title compound as a white solid (5.27 g, 99%).

$\boldsymbol{R}_\mathbf{F}$ (100% hexanes) = 0.37.

^{1}H NMR (501 MHz, CD_2Cl_2): δ = 7.94 (d, *J* = 2.0 Hz, 1H), 7.64 (dd, *J* = 8.2, 2.0 Hz, 1H), 7.60 (d, *J* = 8.1 Hz, 1H), 7.50 (dd, *J* = 3.0, 1.4 Hz, 1H), 7.36 (dd, *J* = 5.0, 2.9 Hz, 1H), 7.34 (dd, *J* = 5.0, 1.4 Hz, 1H), 1.08 (s, 9H).

^{19}F NMR (471 MHz, CD_2Cl_2): δ = –62.9 (s, 3F).

^{13}C NMR (151 MHz, CD_2Cl_2): δ = 146.81 (q, *J* = 1.4 Hz), 141.3, 136.41 (q, *J* = 3.8 Hz), 133.0, 131.3, 130.3, 129.25 (q, *J* = 32.5 Hz), 125.91 (q, *J* = 3.7 Hz), 125.8, 124.8, 124.4 (q, *J* = 272.1 Hz), 48.6, 31.0.

13C NMR (151 MHz, CD2Cl2) δ 146.81 (q, J = 1.4 Hz), 136.41 (q, J = 3.8 Hz), 129.25 (q, J = 32.5 Hz), 125.91 (q, J = 3.7 Hz).

HRMS *m/z* (GC-EI): calcd. For $C_{15}H_{15}S_2F_3$ ($[M]^+$): 316.056181; found: 316.056670.

2-bromo-6-(trifluoromethyl)benzo[*b*]thieno[3,2-*d*]thiophene (S9)

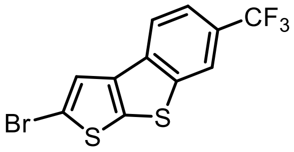

In a flame-dried Schlenk flask under argon 3-(2-(tert-butylthio)-4-(trifluoromethyl)phenyl)thiophene (5.14 g, 16.3 mmol, 1.00 eq.) was dissolved in dry $CHCl_3$ (40 mL) and AcOH (40 mL). *N*-Bromosuccinimide (6.51 g, 36.6 mmol, 2.25 eq.) was added, and the reaction mixture was stirred at room temperature for 5 h. The reaction was then quenched with water, and the aqueous phase was extracted with DCM (3 x 75 mL). The combined organic layers were washed with 3% NaOH solution and water, dried over anhydrous Na_2SO_4 and concentrated under reduced pressure. Purification by flash column chromatography on silica gel (100% hexanes) afforded a white solid containing 90% of the desired title compound (4.76 g, 90% purity, 78% yield). The material was suitable for use in the subsequent Suzuki coupling without significantly affecting the reaction outcome. Alternatively, recrystallization from hexanes/DCM provided the pure title compound as a white solid.

***R*F** (100% hexanes) = 0.57.

^{1}H NMR (501 MHz, $CDCl_3$): δ = 8.06 (s, 1H), 7.85 (d, *J* = 8.3 Hz, 1H), 7.63 (dd, *J* = 8.4, 1.6 Hz, 1H), 7.49 (s, 1H).

^{19}F NMR (471 MHz, $CDCl_3$): δ = –61.4 (s, 3F).

^{13}C NMR (126 MHz, $CDCl_3$): δ = 142.6, 140.2, 139.4, 134.4, 126.8 (q, *J* = 32.7 Hz), 124.42 (q, *J* = 272.3 Hz), 122.4, 121.8 (q, *J* = 3.4 Hz), 121.6, 120.6 (q, *J* = 4.3 Hz), 113.8.

HRMS *m/z* (ESI): calcd. For $C_{11}H_4S_2F_3Br_1$ ($[M]^+$): 335.888456; found: 335.888030.

Synthesis of substituted (*S*)-BINOLs

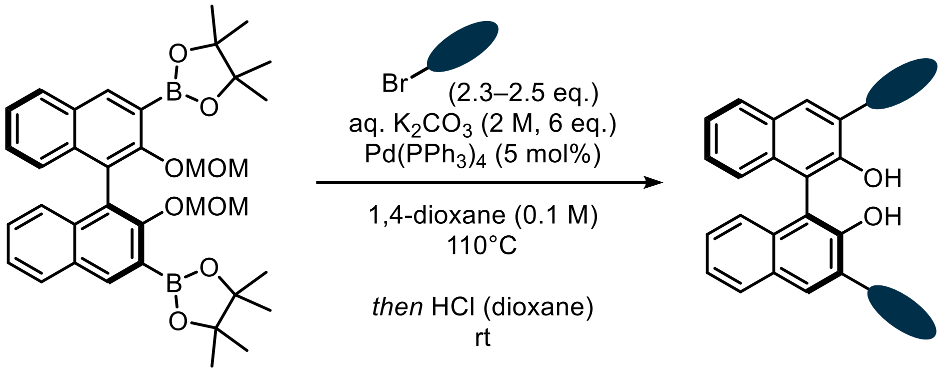

(*S*)-MOM-BINOL Bpin ester

(*S*)-3,3'-bis(benzo[*b*]thiophen-2-yl)-[1,1'-binaphthalene]-2,2'-diol (S12)

A Schlenk flask was charged (*S*)-MOM-BINOL Bpin ester (500 mg, 0.798 mmol, 1.00 eq.), 2-bromobenzothiophene (425 mg, 2.00 mmol, 2.50 eq.), aqueous K_2CO_3 (2 M, 2.4 mL, 4.8 mmol, 6.0 eq.) and 1,4-dioxane (24 mL). Subsequently the suspension was sparged with argon for 30 min, $Pd(PPh_3)_4$ (5 mol%, 46 mg) was added under argon, and the mixture was heated to 110 °C overnight. After addition of saturated NH_4Cl solution the aqueous phase was extracted with DCM (3 x 50 mL). The combined organic layers were washed with water, dried over anhydrous Na_2SO_4 and concentrated under reduced pressure. The crude material was dissolved in HCl (4 M in 1,4-dioxane, 2 mL) and stirred at room temperature overnight. The reaction mixture was diluted with 10% HCl and DCM. The aqueous phase was washed with DCM (3 x 25 mL), the combined organic layers were washed with water, dried over anhydrous Na_2SO_4 and concentrated under reduced pressure. Purification via flash column chromatography on silica gel (2% ethyl acetate, pentane) furnished the title compound as a white solid (307 mg, 70%).

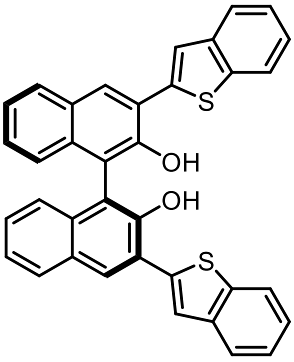

***R*$_F$** (50% DCM, hexanes) = 0.52.

^{1}H NMR (600 MHz, CD_2Cl_2): δ = 8.45 (d, *J* = 0.8 Hz, 2H), 8.03 – 7.98 (m, 4H), 7.92 – 7.88 (m, 2H), 7.87 – 7.80 (m, 2H), 7.43 (ddd, *J* = 8.1, 6.8, 1.2 Hz, 2H), 7.41 – 7.35 (m, 4H), 7.33 (ddd, *J* = 8.2, 6.8, 1.3 Hz, 2H), 7.19 (ddt, *J* = 8.5, 1.4, 0.8 Hz, 2H), 5.81 (d, *J* = 0.6 Hz, 2H).

^{13}C NMR (151 MHz, CD_2Cl_2): δ = 150.5, 140.9, 140.1, 139.6, 133.4, 131.3, 129.8, 129.1, 128.3, 125.2, 125.1, 124.9, 124.7, 124.4, 124.3, 123.8, 122.3, 112.7.

HRMS *m/z* (ESI): calcd. For $C_{36}H_{21}S_2O_2$ ([M–H]$^-$): 549.098850; found: 549.099530.

$[\alpha]_D^{25}$ = +127.7 (c = 0.13, $CHCl_3$).

(*S*)-3,3'-bis(benzo[*b*]thieno[3,2-*d*]thiophen-2-yl)-[1,1'-binaphthalene]-2,2'-diol (S13)

A Schlenk flask was charged (*S*)-MOM-BINOL Bpin ester (216 mg, 0.345 mmol, 1.00 eq.), 2-bromobenzo[*b*]thieno[3,2-*d*]thiophene (214 mg, 0.80 mmol, 2.31 eq.), aqueous K_2CO_3 (2 M, 1.0 mL, 2.1 mmol, 6.0 eq.) and 1,4-dioxane (3.4 mL). Subsequently the suspension was sparged with argon for 30 min, $Pd(PPh_3)_4$ (5 mol%, 20 mg) was added under argon, and the mixture was heated to 110 °C overnight. After addition of saturated NH_4Cl solution the aqueous phase was extracted with DCM (3 x 50 mL). The combined organic layers were washed with water, dried over anhydrous Na_2SO_4 and concentrated under reduced pressure. The crude material was dissolved in HCl (4 M in 1,4-dioxane, 2 mL) and stirred at room temperature overnight. The reaction mixture was diluted with 10% HCl and DCM. The aqueous phase was washed with DCM (3 x 25 mL), the combined organic layers were washed with water, dried over anhydrous Na_2SO_4 and concentrated under reduced pressure. Purification via flash column chromatography on silica gel (30% DCM, pentane) furnished the title compound as a light yellow solid (195 mg, 85%).

***R*$_F$** (50% DCM, hexanes) = 0.53.

^{1}H NMR (600 MHz, CD_2Cl_2): δ = 8.43 (d, *J* = 0.8 Hz, 2H), 8.23 (s, 2H), 8.01 (tdd, *J* = 7.9, 1.2, 0.6 Hz, 4H), 7.88 (ddd, *J* = 8.0, 1.1, 0.7 Hz, 2H), 7.47 – 7.43 (m, 4H), 7.41 – 7.33 (m, 4H), 7.19 (dd, *J* = 8.4, 1.0 Hz, 2H).

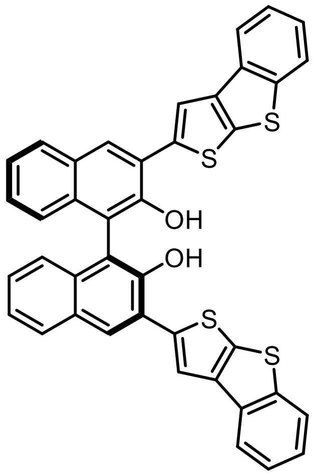

^{13}C NMR (151 MHz, CD_2Cl_2): δ = 150.1, 144.0, 142.6, 142.0, 138.7, 133.3, 133.1, 129.9, 129.9, 129.0, 128.2, 125.3, 125.2, 124.9, 124.4, 124.1, 123.6, 122.0, 119.5, 112.7.

HRMS *m/z* (ESI): calcd. For $C_{40}H_{21}S_4O_2$ ([M–H]$^-$): 661.04299; found: 661.04392.

$[\alpha]_D^{25}$ = +150.5 (c = 0.16, $CHCl_3$).

(*S*)-3,3'-bis(6-(trifluoromethyl)benzo[*b*]thieno[3,2-*d*]thiophen-2-yl)-[1,1'-binaphthalene]-2,2'-diol (S14)

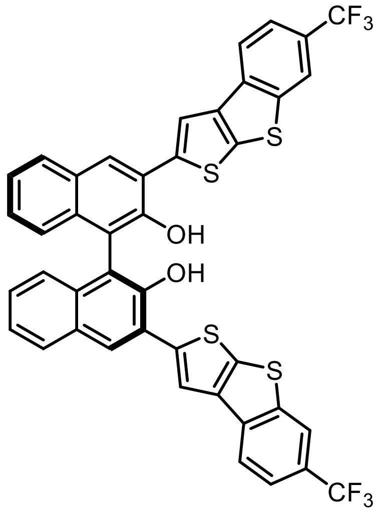

A Schlenk flask was charged (*S*)-MOM-BINOL Bpin ester (1.50 g, 2.40 mmol, 1.00 eq.), 2-bromo-6-(trifluoromethyl)benzo[*b*]thieno[3,2-*d*]thiophene (2.07 g, 5.51 mmol, 2.30 eq.), aqueous K_2CO_3 (2 M, 7.2 mL, 14 mmol, 6.0 eq.) and 1,4-dioxane (24 mL). Subsequently the suspension was sparged with argon for 30 min, $Pd(PPh_3)_4$ (5 mol%, 139 mg) was added under argon, and the mixture was heated to 110 °C for 2 d. After addition of saturated NH_4Cl solution the aqueous phase was extracted with DCM (3 x 50 mL). The combined organic layers were washed with water, dried over anhydrous Na_2SO_4 and concentrated under reduced pressure. The crude material was dissolved in HCl (4 M in 1,4-dioxane, 4 mL) and stirred at room temperature for 26 h. The reaction mixture was diluted with 10% HCl and DCM. The aqueous phase was washed with DCM (3 x 25 mL), the combined organic layers were washed with water, dried over anhydrous Na_2SO_4 and concentrated under reduced pressure. Purification via flash column chromatography on silica gel (30% DCM, hexanes) furnished the title compound as a yellow solid (1.68 g, 88%).

$\boldsymbol{R}_F$ (30% DCM, hexanes) = 0.26.

^{1}H NMR (501 MHz, $(CD_3)_2CO$): δ = 8.77 (s, 2H), 8.56 (s, 2H), 8.53 (s, 2H), 8.42 (s, 2H), 8.33 (d, *J* = 8.3 Hz, 2H), 8.03 (d, *J* = 8.1 Hz, 2H), 7.78 (dd, *J* = 8.4, 1.7 Hz, 2H), 7.38 (ddd, *J* = 8.0, 6.8, 1.2 Hz, 2H), 7.29 (ddd, *J* = 8.2, 6.8, 1.3 Hz, 2H), 7.11 (d, *J* = 8.1 Hz, 2H).

^{19}F NMR (471 MHz, $(CD_3)_2CO$): δ = –61.7 (s, 6F).

^{13}C NMR (126 MHz, $(CD_3)_2CO$): δ = 151.9, 144.8, 144.5, 142.2, 141.8, 136.6, 134.8, 130.1, 129.3, 128.9, 127.9, 127.0, 126.8, 126.7, 126.5, 126.2, 125.3, 125.1, 124.9, 124.6, 123.0, 122.5, 122.4, 122.4, 122.4, 122.3, 121.7, 121.6, 121.6, 121.6, 119.1, 114.9.

HRMS *m/z* (ESI): calcd. For $C_{42}H_{19}S_4F_6O_2$ ($[M–H]^-$): 797.01776; found: 797.01830.

$[\boldsymbol{\alpha}]_{\boldsymbol{D}}^{\mathbf{25}}$ = +142.0 (c = 0.30, $CHCl_3$).

Synthesis of IDPi catalysts

IDPi catalysts **S1a**[9], **S2a**[10], **5a**[11], **5b**[11], and **6b**[11] were synthesized according to reported procedures and their NMR-spectroscopic data was in agreement with the literature data.

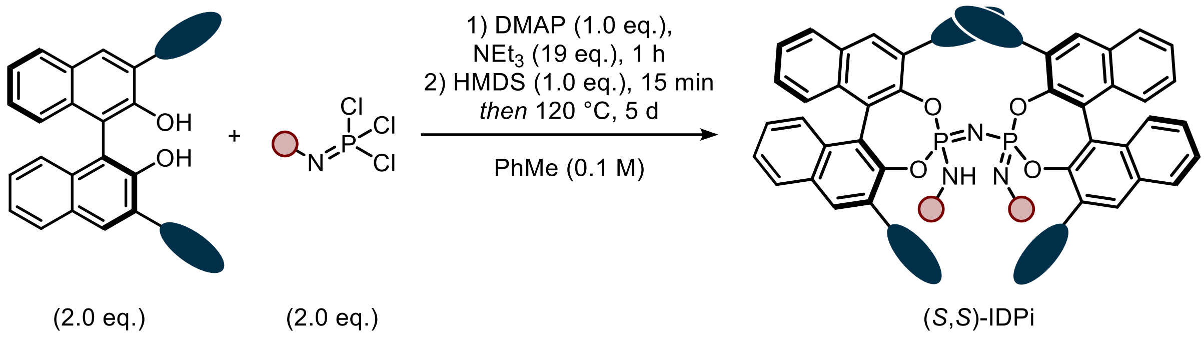

(*S*,*S*)-(benzothiophen-2-yl)-CF_3-IDPi (5a)

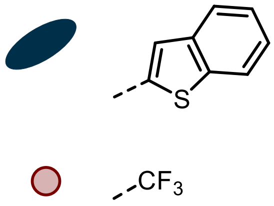

(*S*)-BINOL **S12** (180 mg, 0.327 mmol, 2.00 eq.) was added to a flame-dried Schlenk flask under argon and dried at 50 °C under high vacuum overnight. The flask was then placed under argon. $P(NSO_2CF_3)Cl_3$ (52 µL, 0.325 mmol, 2.00 eq.), toluene (3.3 mL) and NEt_3 (0.45 mL, 3.26 mmol, 20.0 eq.) were added and the reaction mixture was stirred at room temperature for 1 h. Subsequently, HMDS (34.0 µL, 0.163 mmol, 1.00 eq.) was added, the mixture was stirred at room temperature for 15 min, and then heated to 120 °C for 3 d. After cooling to room temperature the mixture was diluted with DCM and quenched by addition of 1 M HCl. The aqueous phase was extracted with DCM (3 x 25 mL), the combined organic layers were washed with 1 M HCl and water, dried over anhydrous Na_2SO_4 and concentrated under reduced pressure. Purification via flash column chromatography on silica gel (1st column: 0–3% EtOAc, DCM, 2nd column: 20–30% EtOAc, hexanes) afforded a white solid. The product was acidified by dissolving it in DCM and vividly stirring it with 6 M HCl for 10 min. The organic layer was concentrated under reduced pressure and dried under high vacuum, furnishing the title compound as an off-white solid (168 mg, 70%).

R$_F$ (50% EtOAc, hexanes) = 0.29.

^{1}H NMR (501 MHz, CD_2Cl_2): δ = 8.32 (s, 2H), 8.05 (d, *J* = 8.3 Hz, 2H), 8.01 (d, *J* = 8.2 Hz, 2H), 7.85 (ddd, *J* = 8.0, 6.7, 1.1 Hz, 2H), 7.83 – 7.78 (m, 2H), 7.73 (dd, *J* = 6.9, 2.1 Hz, 2H), 7.66 (ddd, *J* = 8.2, 6.8, 1.2 Hz, 2H), 7.61 – 7.52 (m, 4H), 7.44 (d, *J* = 7.8 Hz, 2H), 7.42 – 7.37 (m, 2H), 7.37 – 7.31 (m, 6H), 7.31 – 7.25 (m, 4H), 7.03 (s, 2H), 6.90 (td, *J* = 7.5, 1.2 Hz, 2H), 6.85 (td, *J* = 7.6, 1.3 Hz, 2H), 6.81 (s, 2H).

^{19}F NMR (471 MHz, CD_2Cl_2): δ = -78.53.

^{31}P NMR (203 MHz, CD_2Cl_2): δ = -15.79.

^{13}C NMR (151 MHz, CD_2Cl_2): δ = 143.74, 143.71, 143.68, 142.8, 142.71, 142.68, 140.9, 140.4, 140.2, 139.9, 137.3, 137.2, 132.4, 132.14, 132.08, 132.0, 131.1, 130.4, 129.6, 129.1, 128.1, 127.9, 127.6, 127.4, 127.3, 127.24, 127.18, 127.15, 126.53, 126.52, 126.51, 126.37, 126.35, 126.3, 125.2, 125.11, 125.06, 124.99, 124.95, 124.72, 124.70, 124.62, 124.59, 124.0, 123.92, 123.91, 123.90, 122.87, 122.61, 122.61, 122.60, 121.97, 121.95, 120.7, 118.6, 116.5.

HRMS *m/z* (ESI): calcd. For $C_{74}H_{40}S_6F_6O_8N_3P_2$ ($[M–H]^-$): 1466.05246; found: 1466.05376.

$[\alpha]_D^{25}$ = +377.1 (c = 0.32, $CHCl_3$).

(*S*,*S*)-(benzothiophen-2-yl)-C_6F_5-IDPi (5b)

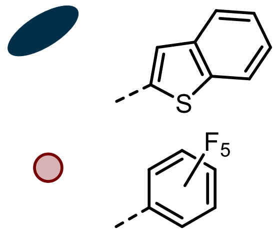

(*S*)-BINOL **S12** (210 mg, 0.381 mmol, 2.04 eq.) was added to a flame-dried Schlenk flask under argon and dried at 50 °C under high vacuum overnight. The flask was then placed under argon. $P(NSO_2C_6F_5)Cl_3$ (144 mg, 0.377 mmol, 2.01 eq.), toluene (3.7 mL) and NEt_3 (0.52 mL, 3.73 mmol, 19.9 eq.) were added and the reaction mixture was stirred at room temperature for 1 h. Subsequently, HMDS (39.0 µL, 0.187 mmol, 1.00 eq.) was added, the mixture was stirred at room temperature for 15 min, and then heated to 120 °C for 3 d. After cooling to room temperature the mixture was diluted with DCM and quenched by addition of 1 M HCl. The aqueous phase was extracted with DCM (3 x 25 mL), the combined organic layers were washed with 1 M HCl and water, dried over anhydrous Na_2SO_4 and concentrated under reduced pressure. Purification via flash column chromatography on silica gel (1st column: 0–4% EtOAc, DCM, 2nd column: 10–40% EtOAc, hexanes) afforded a white solid. The product was acidified by dissolving it in DCM and vividly stirring it with 6 M HCl for 20 min. The organic layer was

concentrated under reduced pressure and dried under high vacuum, furnishing the title compound as an off-white solid (181 mg, 58%).

***R*F** (50% EtOAc, hexanes) = 0.32.

^{1}H NMR (501 MHz, CD_2Cl_2): δ = 8.26 (s, 2H), 8.01 (dd, *J* = 17.6, 8.2 Hz, 4H), 7.89 – 7.84 (m, 2H), 7.82 – 7.75 (m, 4H), 7.61 – 7.53 (m, 4H), 7.43 (d, *J* = 8.6 Hz, 2H), 7.38 – 7.29 (m, 8H), 7.25 (d, *J* = 7.7 Hz, 2H), 7.23 – 7.16 (m, 4H), 7.09 (s, 2H), 6.80 – 6.69 (m, 6H).

^{19}F NMR (471 MHz, CD_2Cl_2): δ = -136.59 (d, *J* = 21.4 Hz, 4F), -146.07 (t, *J* = 21.7 Hz, 2F), -159.60 (t, *J* = 21.0 Hz, 4F).

^{31}P NMR (203 MHz, CD_2Cl_2): δ = -16.89.

^{13}C NMR (126 MHz, CD_2Cl_2): δ = 145.4, 144.8, 143.43, 143.39, 143.35, 142.8, 142.5, 140.9, 140.4, 139.8, 139.2, 138.6, 137.0, 136.9, 136.6, 132.4, 132.2, 131.9, 131.8, 130.13, 130.09, 129.4, 129.0, 128.1, 127.8, 127.6, 127.5, 127.3, 127.0, 126.5, 125.9, 125.84, 125.82, 125.4, 125.2, 125.0, 124.9, 124.8, 124.5, 123.9, 123.8, 122.6, 121.8, 121.7, 117.7.

HRMS *m/z* (ESI): calcd. For $C_{74}H_{40}S_6F_6O_8N_3P_2$ ($[M–H]^-$): 1662.04607; found: 1662.04538.

$[\alpha]_D^{25}$ = +329.6 (c = 0.26, $CHCl_3$).

(*S*,*S*)-(benzo[*b*]thieno[3,2-*d*]thiophen-2-yl)-C_6F_5-IDPi (6b)

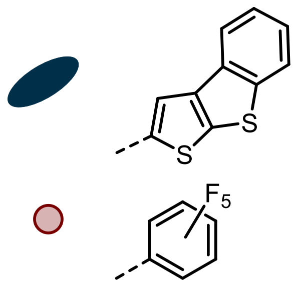

(*S*)-BINOL **S13** (158 mg, 0.238 mmol, 2.01 eq.) was added to a flame-dried Schlenk flask under argon and dried at 50 °C under high vacuum overnight. The flask was then placed under argon. $P(NSO_2C_6F_5)Cl_3$ (92 mg, 0.24 mmol, 2.03 eq.), toluene (2.4 mL) and NEt_3 (0.33 mL, 2.37 mmol, 19.9 eq.) were added and the reaction mixture was stirred at room temperature for 1 h. Subsequently, HMDS (24.7 µL, 0.119 mmol, 1.00 eq.) was added, the mixture was stirred at room temperature for 15 min, and then heated to 120 °C for 3 d. After cooling to room temperature the mixture was diluted with DCM and quenched by addition of 1 M HCl. The aqueous phase was extracted with DCM (3 x 25 mL), the combined organic layers were washed with 1 M HCl and water, dried over anhydrous Na_2SO_4 and concentrated under reduced pressure. Purification via flash column chromatography on silica gel (1st column: 0–3% EtOAc, DCM, 2nd column: 10–20% EtOAc,

hexanes) afforded a light yellow solid. The product was acidified by filtration over a short plug of DOWEX 50WX8 (H-form, eluted with DCM). The product was concentrated under reduced pressure and dried under high vacuum, furnishing the title compound as a light yellow solid (95 mg, 42%).

$\boldsymbol{R_F}$ (50% EtOAc, hexanes) = 0.44.

^{1}H NMR (600 MHz, CD_2Cl_2): δ = 8.18 (s, 2H), 8.06 – 7.98 (m, 4H), 7.89 (d, *J* = 8.2 Hz, 2H), 7.78 (d, *J* = 7.9 Hz, 2H), 7.77 – 7.66 (m, 3H), 7.58 (ddd, *J* = 8.1, 6.8, 1.1 Hz, 2H), 7.55 – 7.43 (m, 6H), 7.43 – 7.23 (m, 13H), 7.20 – 7.13 (m, 2H), 6.84 (t, *J* = 7.4 Hz, 2H), 6.66 (t, *J* = 7.4 Hz, 2H).

^{19}F NMR (471 MHz, CD_2Cl_2): δ = -136.22 (s, 4F), -146.71 (s, 2F), -160.11 (s, 4F).

^{31}P NMR (203 MHz, CD_2Cl_2): δ = -15.95.

^{13}C NMR (151 MHz, CD_2Cl_2): δ = 145.0, 143.6, 143.4, 143.2, 142.91, 142.87, 142.8, 142.7, 142.3, 134.0, 139.6, 138.7, 138.2, 136.5, 133.1, 132.9, 132.3, 131.84, 131.79, 131.4, 129.6, 129.3, 128.6, 127.9, 127.7, 127.41, 127.37, 127.2, 127.1, 127.0, 126.9, 126.1, 125.1, 124.7, 124.4, 124.1, 123.6, 123.1, 123.0, 122.9, 122.5, 121.7, 120.9, 118.7.

HRMS *m/z* (ESI): calcd. For $C_{92}H_{40}S_{10}F_{10}O_8N_3P_2$ ($[M–H]^-$): 1885.93435; found: 1885.93518.

$[\alpha]_D^{25}$ = +451.1 (c = 0.12, $CHCl_3$).

(*S*,*S*)-(6-(trifluoromethyl)benzo[*b*]thieno[3,2-*d*]thiophen-2-yl)-C_6F_5-IDPi (7b)

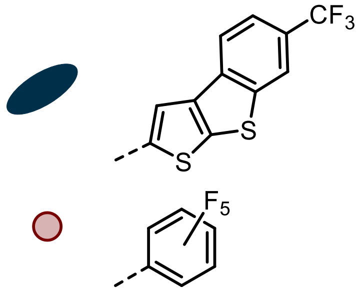

(*S*)-BINOL **S14** (1.01 g, 1.23 mmol, 2.01 eq.) was added to a flame-dried Schlenk flask under argon and dried at 50 °C under high vacuum overnight. The flask was then placed under argon. $P(NSO_2C_6F_5)Cl_3$ (481 mg, 1.26 mmol, 2.00 eq.), DMAP (77 mg, 0.6 mmol, 1.0 eq.), toluene (12.6 mL) and NEt_3 (1.7 mL, 12 mmol, 19 eq.) were added and the reaction mixture was stirred at room temperature for 1 h. Subsequently, HMDS (131 μL, 0.63 mmol, 1.00 eq.) was added, the mixture was stirred at room temperature for 15 min, and then heated to 120 °C for 5 d. After cooling to room temperature the mixture was diluted with DCM and quenched by addition of 1 M HCl. The aqueous phase was extracted with DCM (3 x 25 mL), the combined organic layers were washed

with 1 M HCl and water, dried over anhydrous Na_2SO_4 and concentrated under reduced pressure. Purification via flash column chromatography on silica gel (1st column: 30% to 60% EtOAc, hexanes, 2nd column: 80% DCM, hexanes to 100% DCM to 2% EtOAc, DCM) afforded a white solid. The product was acidified by dissolving it in DCM and vividly stirring it with 6 M HCl for 20 min. The organic layer was concentrated under reduced pressure and dried under high vacuum, furnishing the title compound as a beige solid (424 mg, 31%).

***R*_F** (70% EtOAc, hexanes) = 0.26.

^{1}H NMR (501 MHz, CD_2Cl_2): δ = 8.27 (s, 2H), 8.12 – 8.01 (m, 6H), 7.85 – 7.73 (m, 6H), 7.67 – 7.59 (m, 6H), 7.57 – 7.47 (m, 6H), 7.41 – 7.32 (m, 4H), 7.20 (d, *J* = 8.4 Hz, 2H), 7.12 (s, 2H), 6.81 (dd, *J* = 8.0, 1.7 Hz, 2H), 5.66 (s, 2H).

^{19}F NMR (471 MHz, CD_2Cl_2): δ = –61.7 (s, 6F), –61.8 (s, 6F), –136.0 (d, *J* = 20.3 Hz, 4F), –145.6 (s, 2F), –159.6 (t, *J* = 20.2 Hz, 4F).

^{31}P NMR (203 MHz, CD_2Cl_2): δ = –16.6.

^{13}C NMR (151 MHz, CD_2Cl_2): δ = 145.1, 145.0, 144.6, 143.30, 143.28, 142.9, 142.68, 142.65, 142.6, 142.3, 142.24, 142.20, 142.0, 141.7, 141.2, 140.61, 140.55, 140.5, 138.4, 138.3, 136.7, 136.6, 136.5, 135.2, 135.0, 132.3, 131.9, 131.8, 131.5, 130.1, 129.8, 128.8, 128.2, 128.04, 128.01, 127.8, 127.7, 127.5, 127.40, 127.36, 127.2, 127.0, 126.8, 126.6, 126.42, 126.39, 126.2, 126.0, 125.9, 125.7, 125.5, 125.4, 125.09, 125.08, 125.07, 124.1, 123.7, 123.6, 123.2, 123.0, 122.3, 122.01, 121.99, 121.97, 121.94, 121.88, 121.8, 121.2, 121.14, 121.12, 121.10, 120.9, 120.62, 120.59, 120.57, 120.5, 119.71, 119.68, 119.66, 119.6, 118.6, 117.2.

HRMS *m/z* (ESI): calcd. For $C_{96}H_{36}S_{10}F_{22}O_8N_3P_2$ ($[M–H]^-$): 2157.883923; found: 2157.883720.

LC (10 mm Chiralpak QN-AX, 4.0 mm i.d., MeOH/AcOH/NH_4OAc = 98:2:0.5 (v/v/w), 1.0 mL/min, 2.1 MPa, RT, 254 nm): t_{R1} = 2.62 min (minor), t_{R2} = 5.39 min (major), er = 99.5:0.5 (99% ee).

$[\boldsymbol{\alpha}]_{\boldsymbol{D}}^{\mathbf{25}}$ = +398.1 (c = 0.31, $CHCl_3$).

8. Mechanistic Studies

8.1. Probe for a Common Benzylic Carbocation Intermediate

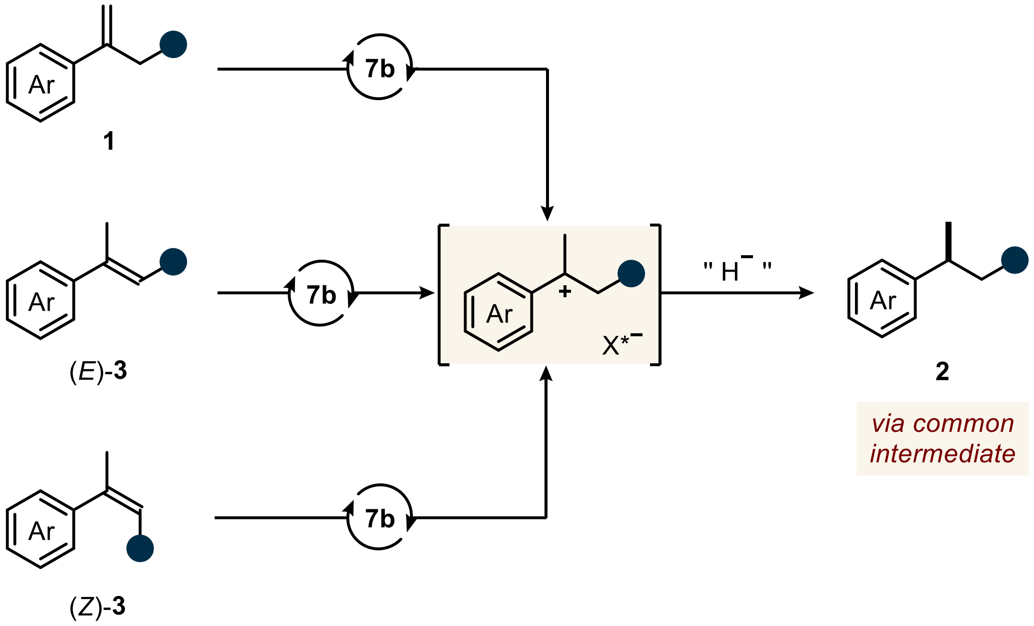

If the styrene reduction proceeds via protonation to generate a benzylic carbocation intermediate, followed by hydride transfer from the silane to yield chiral product **2**, the same enantiomer is expected regardless of the initial alkene isomer (**1** or (*E*)-**3** or (*Z*)-**3**), as the intermediate loses its stereochemical information.

Substrates with trisubstituted double bonds ((*E*)-**3** and (*Z*)-**3**) were found to exhibit lower reactivity, requiring higher temperatures than **1** (see Table S19).

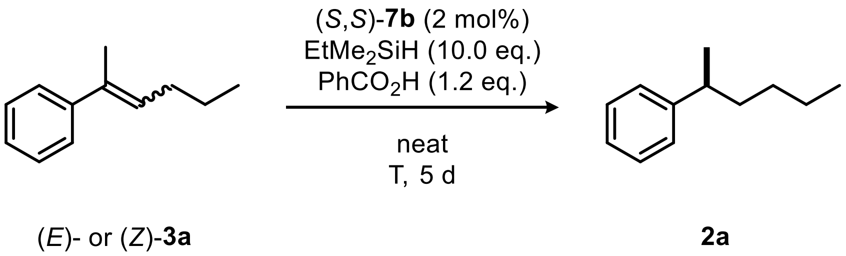

Table S19: *Synthesis of* ***2a*** *from different stereoisomeric alkenes* (E)*-**3a**, or* (Z)*-**3a** at different temperatures.*

	from (*E*)-**3a**		from (*Z*)-**3a**	
**temp.**	**yield**	**er**	**yield**	**er**
80 °C	95%	79.5:20.5	49%	79.5:20.5
60 °C	64%	81.5:18.5	12%	81:19
40 °C	traces	n.d.	traces	n.d.
20 °C	traces	n.d.	traces	n.d.

The reduction of electron-rich styrenes, such as **3j**, proceeded more readily, leading to the selection of the naphthyl derivative as a model substrate. Subjecting **1j**, (*E*)-**3j**, or (*Z*)-**3j** to the reaction conditions according to **GP1** (using 10 eq. $EtMe_2SiH$ under neat reaction conditions for 2 d) gave the following results (see Table S20).

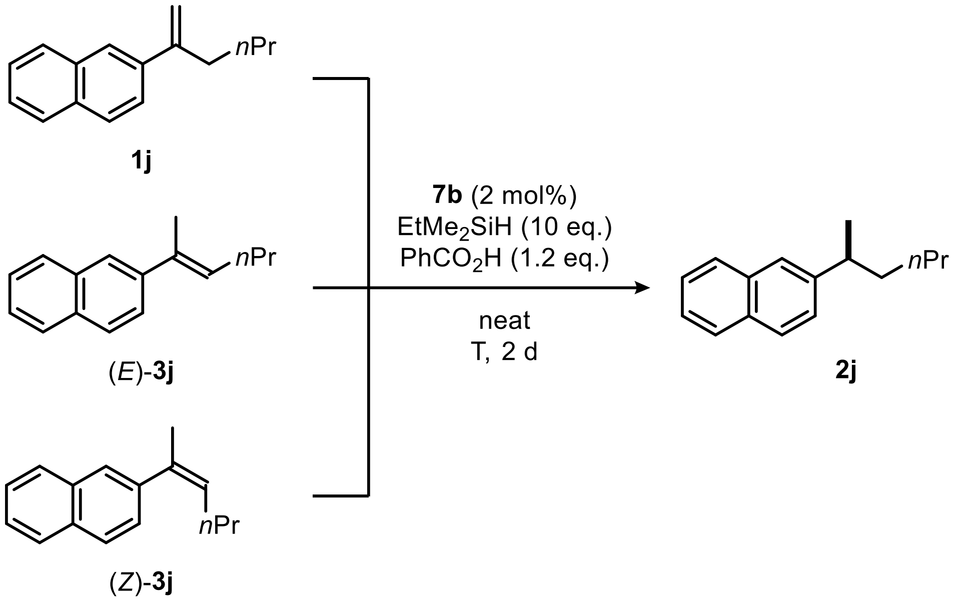

Table S20: *Synthesis of **2j** from different isomeric alkenes **1j**, (*E*)-**3j**, or (*Z*)-**3j** at different temperatures.*

	from **1j**		from (*E*)-**3j**		from (*Z*)-**3j**	
**temp.**	**yield**	**er**	**yield**	**er**	**yield**	**er**
80 °C	87%	84:16	>99%	81.5:18.5	>99%	82:18
60 °C	83%	86.5:13.5	>99%	86:14	94%	86:14
40 °C	79%	90.5:9.5	>99%	89:11	43%	89:11
20 °C	78%	94.5:5.5	59%	92.5:7.5	3%	92:8

1j is generally more reactive than (*E*)-**3j** and (*Z*)-**3j**; however, lower product yields are obtained at higher temperatures due to unproductive side reactions. While isomerization is negligible, dimerization to **4j** significantly reduces the product yield. Notably, dimerization does not occur for (*E*)-**3j** and (*Z*)-**3j**. Furthermore, the data indicates (*E*)-**3j** to be more reactive than the corresponding (*Z*)-isomer.

Remarkably, the same product enantiomer was obtained in all three cases with comparable enantioenrichment, suggesting a common intermediate.

8.2. Deuterium Scrambling Experiment

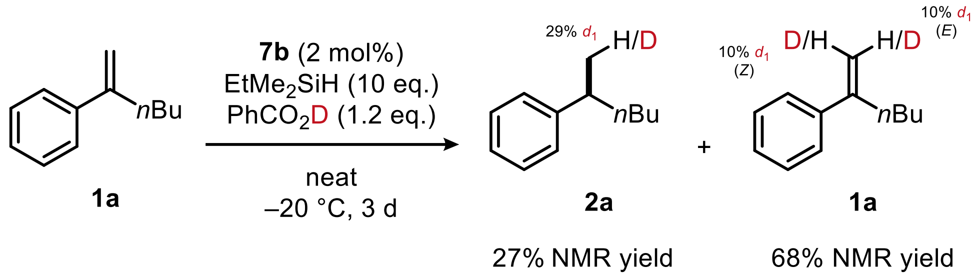

A 2 mL GC vial, equipped with a magnetic stirring bar, was charged with the IDPi **7b** (2 mol%) and benzoic acid-d_1 (0.030 mmol, 1.2 eq.), and then placed under argon. $EtMe_2SiH$ (0.25 mmol, 10 eq.) was added, and the vial was cooled to –78 °C. After addition of styrene **1a** (0.025 mmol, 1.0 eq.), the reaction was stirred at –20 °C for 3 d. The reaction was quenched by addition of pyridine (5 µL) followed by addition of mesitylene (3 µL) as internal standard. An aliquot of the mixture was taken and diluted with C_6D_6 for subsequent ^1H, ^2H, and ^{13}C NMR analysis.

Product Analysis (2a)

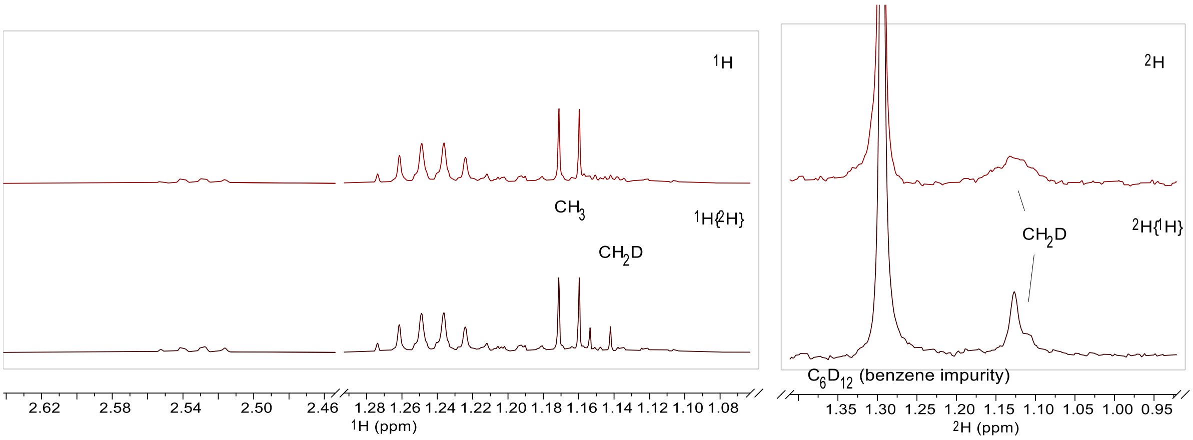

Figure S5: *Selected region of the ^1H and ^2H NMR spectra from the crude reaction mixture for the product analysis.*

Integration based on ^1H NMR was challenging due to signal overlap with other components. Therefore, the ratio was determined using the relative integral of the neighboring ^{13}C signal, which differs between the two species.

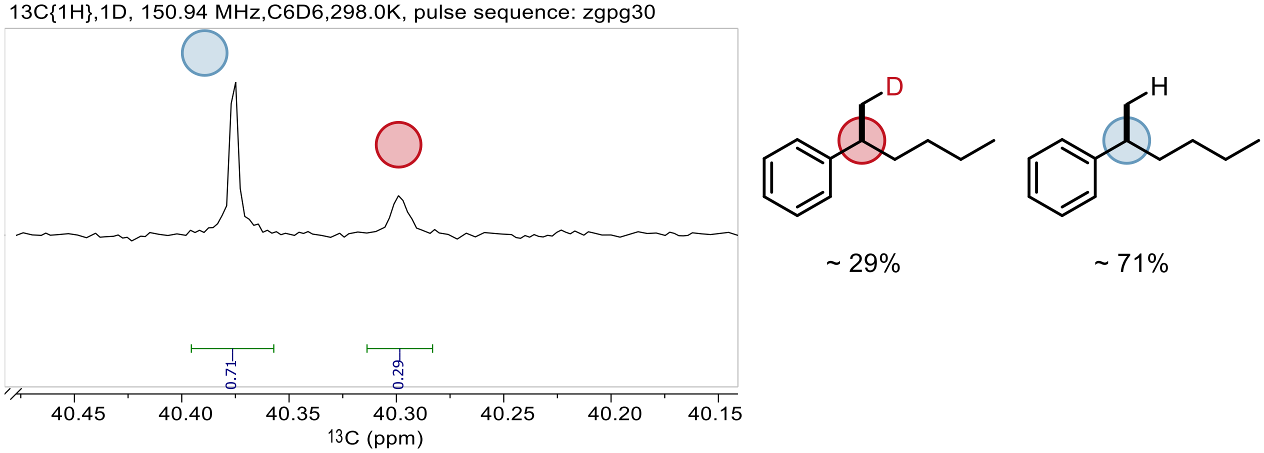

Figure S6: *Selected region of the ^{13}C NMR spectrum from the crude reaction mixture for the product analysis.*

Starting Material Analysis (1a)

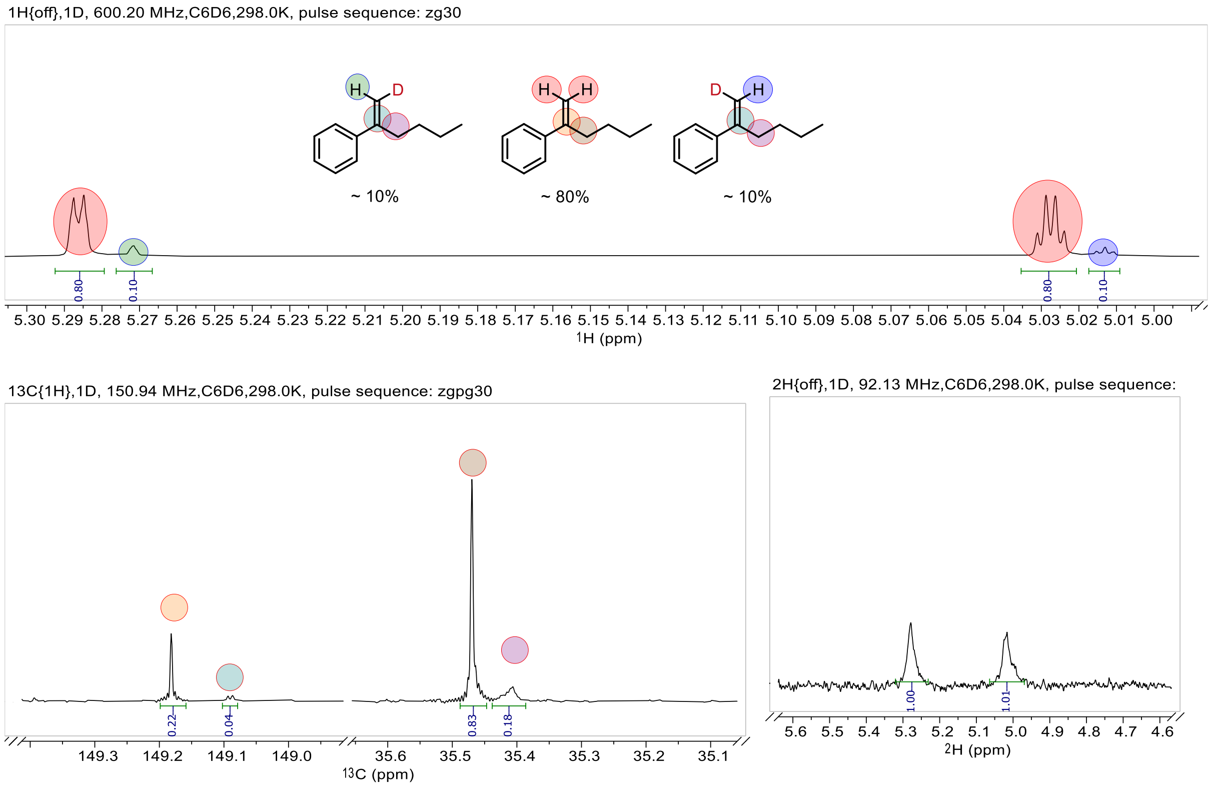

Figure S7: *Selected region of the ^{1}H, ^{2}H, ^{13}C NMR spectra from the reaction crude for the starting material analysis.*

The partial deuteration of the starting material **1a** suggests a reversible protonation equilibrium before the hydride transfer step.

8.3. Investigating the Influence of a Chiral Proton Source on Stereocontrol

To investigate whether the proton source, required for catalyst turnover, is possibly involved in the enantiodetermining step, the influence of an enantiopure chiral proton source was examined. If the proton source were involved beyond catalyst regeneration, a trimolecular transition state could be envisioned, comprising the ion pair formed between styrene **1a** and chiral IDPi **7b**, the hydrosilane, and the proton source. In this case, the enantiomer of the proton source could induce a matched or mismatched scenario relative to (*S*,*S*)-**7b**, leading to two distinct diastereomeric transition states and, consequently, different enantiomeric ratios of the product **2a**. Conversely, if the proton source solely facilitates catalyst turnover without influencing the transition state, the enantiomeric ratio of **2a** should remain unchanged, thereby excluding its role in the enantiodeterming step.

For this experiment, (*R*)- or (*S*)-α-methoxyphenylacetic acid **S15** was employed as a proton source according to **GP1**, with $EtMe_2SiH$ (5.0 eq.) in CyMe (0.5 M) at room temperature for 1 d (see Table S21).

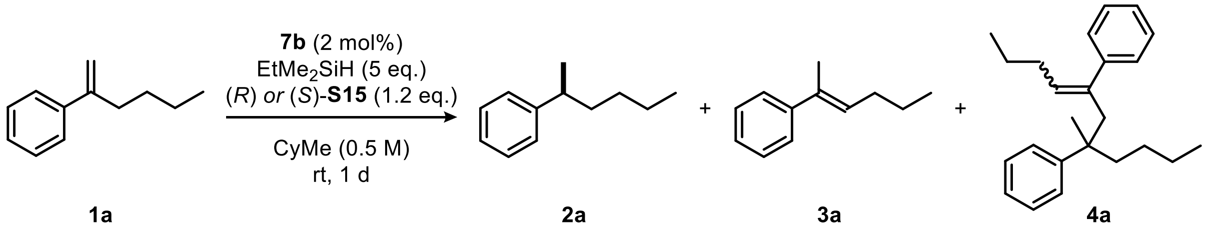

1a **2a** **3a** **4a**

Table S21: *Application of an Enantiopure Proton Source: (*R*)- or (*S*)-α-Methoxyphenylacetic Acid (**S15**) with (*S,S*)-**7b**.*

H^+ Source	yield (2a)	er	yield (3a)	yield (4a)
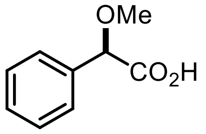 (*R*)-**S15**	51%	89.5:10.5	34%	7%

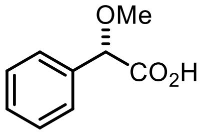 (*S*)-**S15**	51%	89.5:10.5	34%	7%

With both the (*R*)- and the (*S*)-enantiomer of **S15**, the same enantiomeric ratio for **2a** of 89.5:10.5 was obtained, which suggests the proton source to not be involved in the enantiodetermining step.

In a second experiment, (*S*)-(+)-phenylpropionic acid was used as an enantiopure proton source in the reduction of styrene with either (*R*,*R*)-**7b** or (*S*,*S*)-**7b** as the catalyst. In both cases, the major enantiomer of the product was inverted depending on the catalyst enantiomer, as expected. Importantly, the enantiomeric excess remained nearly identical across both reactions (see Table S22), indicating that the chiral proton source does not influence the enantiodetermining step and excluding a matched/mismatched scenario between catalyst and carboxylic acid.

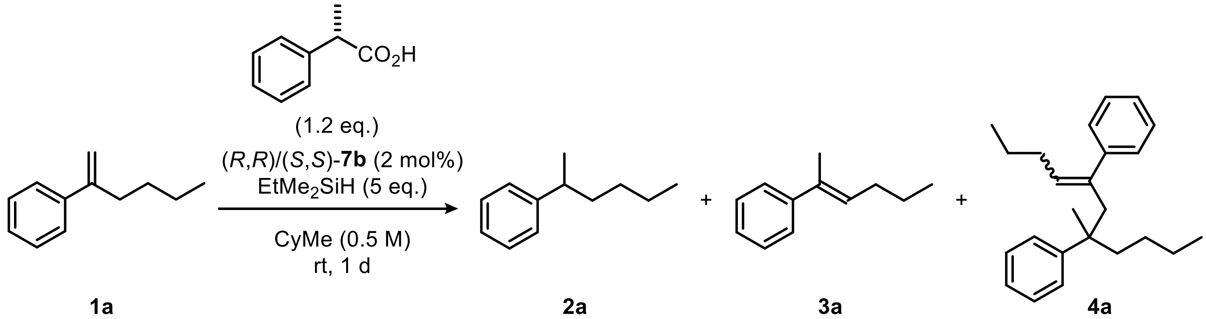

1a **2a** **3a** **4a**

Table S22: *Application of an Enantiopure Proton Source: (*S*)-(+)-Phenylpropionic Acid with (*R,R*)- or (*S,S*)-**7b**.*

IDPi 7b	yield (2a)	er	yield (3a)	yield (4a)
(*R*,*R*)-**7b**	54%	9.5:90.5	33%	6%
(*S*,*S*)-**7b**	54%	90:10	32%	6%

8.4. Detection of Silylated IDPi Species

To detect the silylated IDPi as a catalytic intermediate via NMR spectroscopy, slightly modified reaction conditions were employed. To ensure a homogeneous reaction medium, the experiment was conducted at higher dilution in dry CD_2Cl_2. Additionally, exclusion of proton sources (e.g., benzoic acid or water) was critical to prevent rapid protodesilylation of the intermediate species. To facilitate the detection of low-concentration species, the catalyst loading was increased.

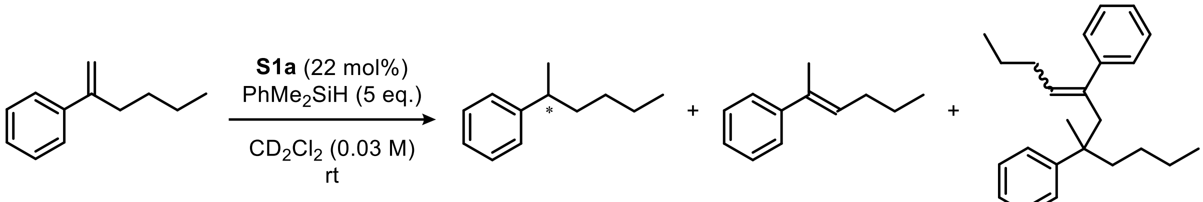

To this end, a dry 3 mm NMR tube under argon was charged with IDPi **S1a** (2.2 mg, 1.2 µmol, 22 mol%), dry CD_2Cl_2 (0.16 mL) and $PhMe_2SiH$ (4.2 µL, 27 µmol, 5.0 eq.). Initial ^{1}H and ^{31}P NMR spectra were recorded. Subsequently, styrene **1a** (1.0 µL, 5.5 µmol, 1.0 eq.) was added, and the reaction was monitored at room temperature.

For acquisition of a reference spectrum of the silylated IDPi **S1a**, a dry 3 mm NMR tube under argon was charged with IDPi **S1a** (2.9 mg, 1.6 µmol, 1.0 eq.), dry CD_2Cl_2 (0.16 mL) and dimethyl(2-methylallyl)(phenyl)silane (3.5 µL, 16 µmol, 10 eq.). ^{1}H and ^{31}P NMR spectra were recorded immediately.

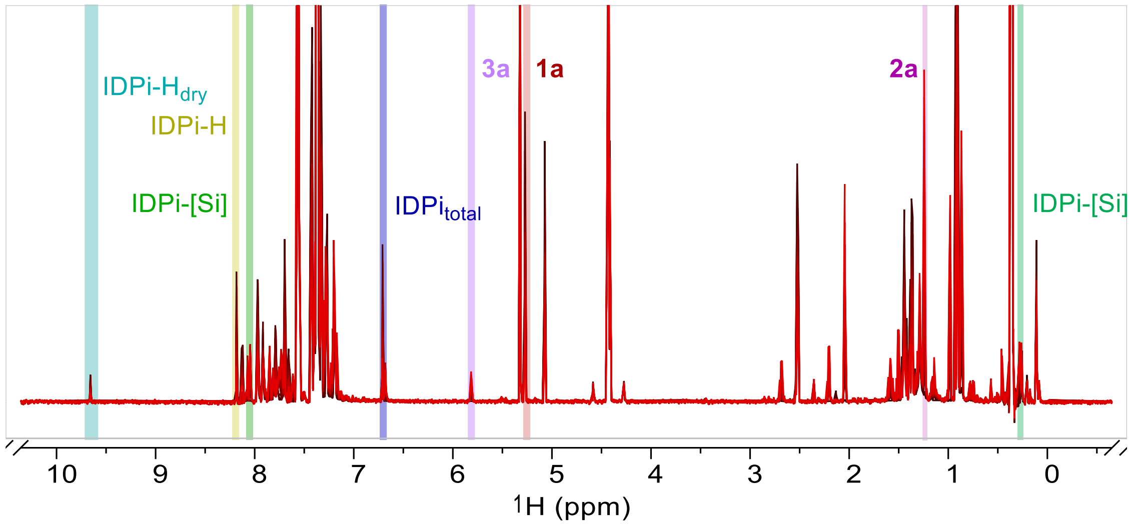

Figure S8: *Superimposed ^{1}H NMR spectra at selected time points, with highlighted regions used for integration of the respective reaction components. The integrals were used to plot the reaction progress.*

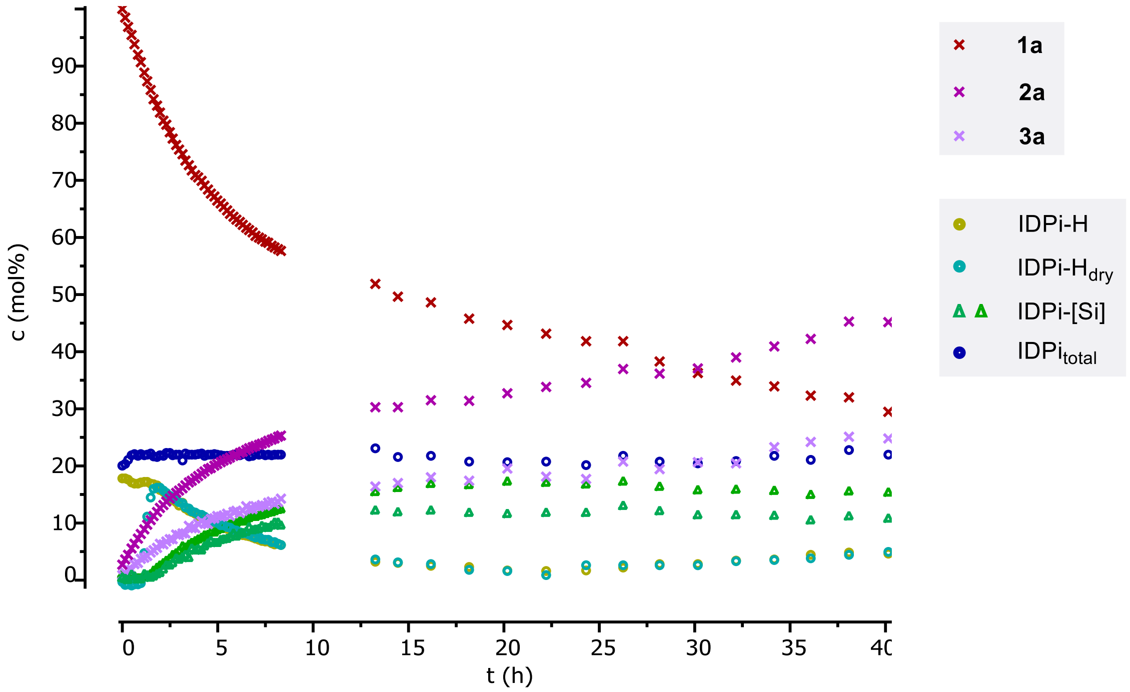

Figure S9: *Reaction progress plot showing the concentration (mol%) of different reaction components over time (h), derived from ^{1}H NMR spectra.*

In the reaction progress plot (Figure S9) derived from the ^{1}H NMR spectra, the initial ~1-hour period shows no signal corresponding to the dry, protic IDPi (IDPi-H$_{dry}$, cyan circles). Only after this period, at around 10% conversion of styrene **1a**, does the signal begin to appear. Simultaneously, a new catalytic species emerges and continues to increase over time (IDPi-[Si], green triangles), whose spectrum matches that of independently prepared silylated IDPi **S1a** (see Figure S12). After approximately 2 hours, the signal of the protic (dry) IDPi decreases again (IDPi-H and IDPi-H$_{dry}$), while that of the silylated IDPi continues to grow.

This initial phase represents a self-drying cycle in which residual water (likely trapped in the IDPi pocket, approx. 0.1 equiv) is scavenged by silyl groups formed as byproducts during the reduction of styrene **1a**. These silyl groups may initially be trapped by the IDPi anion, but the silylated IDPi is rapidly protodesilylated in the presence of water. Once the system is dry, the protic IDPi signal declines as the anion captures silyl groups and can no longer undergo protodesilylation.

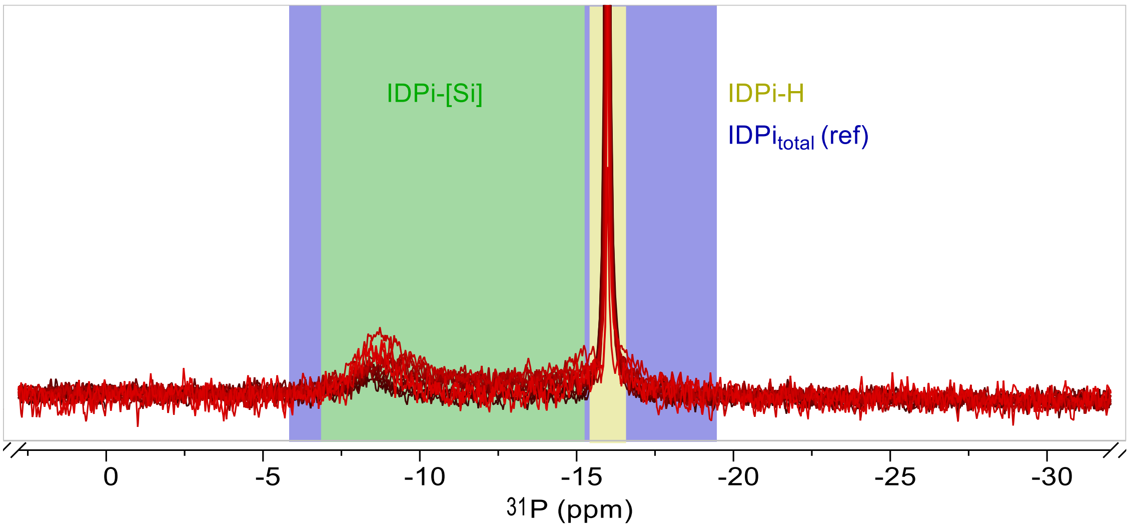

Figure S10: *Superimposed ^{31}P NMR spectra at selected time points, with highlighted regions used for integration of the respective reaction components. The integrals were used to plot the reaction progress. The ^{31}P NMR spectra were normalized to the integral of the whole region (as the sample was removed from the magnet and retuned at later time points).*

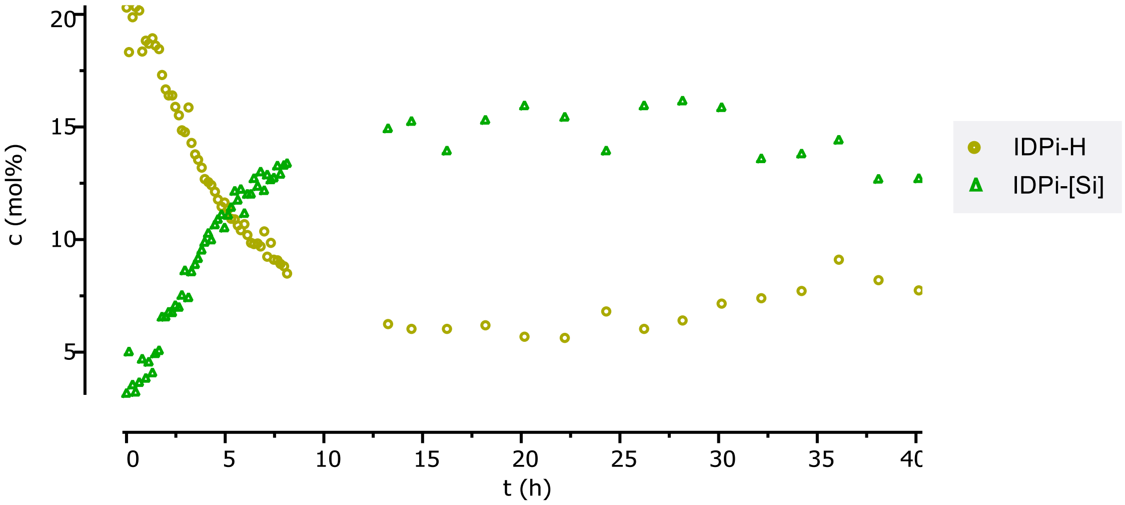

Figure S11: *Reaction progress plot showing the concentration (mol%) of catalytic species over time (h), derived from ^{31}P NMR spectra.*

From the reaction progress plot derived from the ^{31}P NMR spectra (Figure S11), similar conclusions can be drawn.

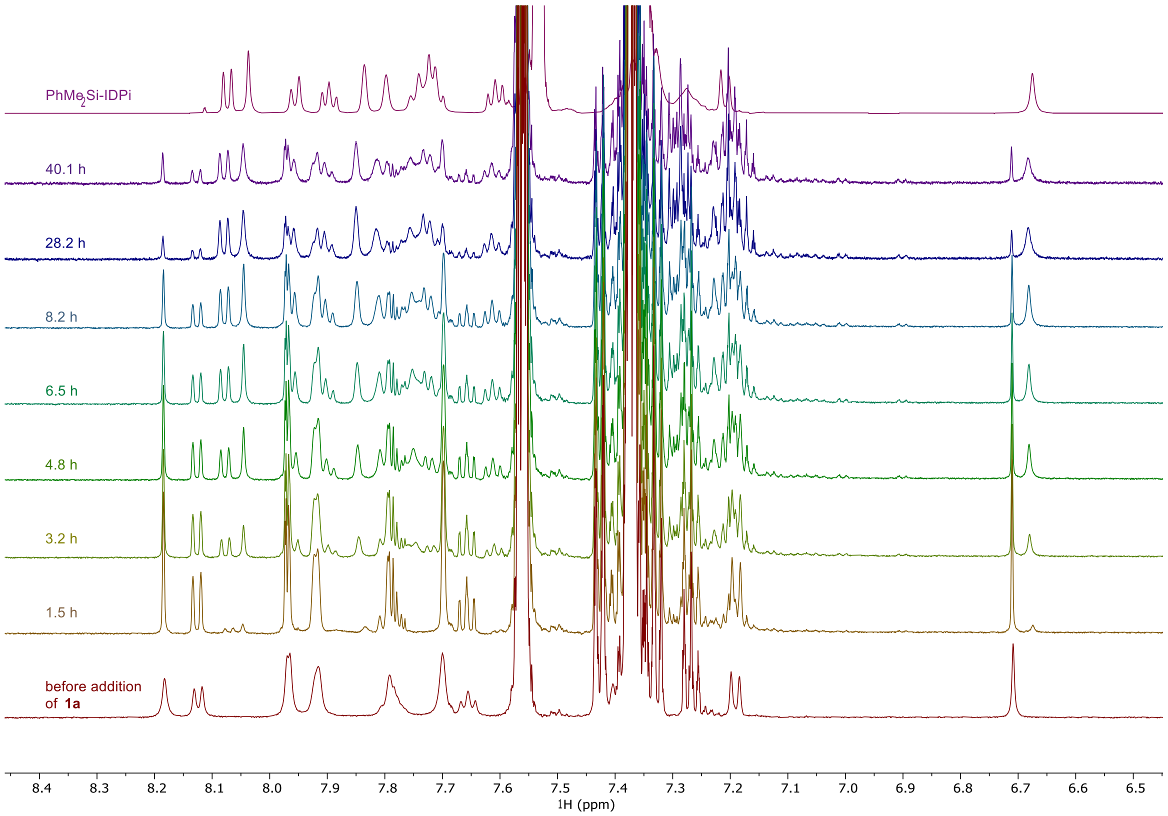

***Figure S12**: Selected region of the stacked ¹H NMR spectra recorded during the reaction, along with the reference spectrum of silylated IDPi **S1a** (top trace).*

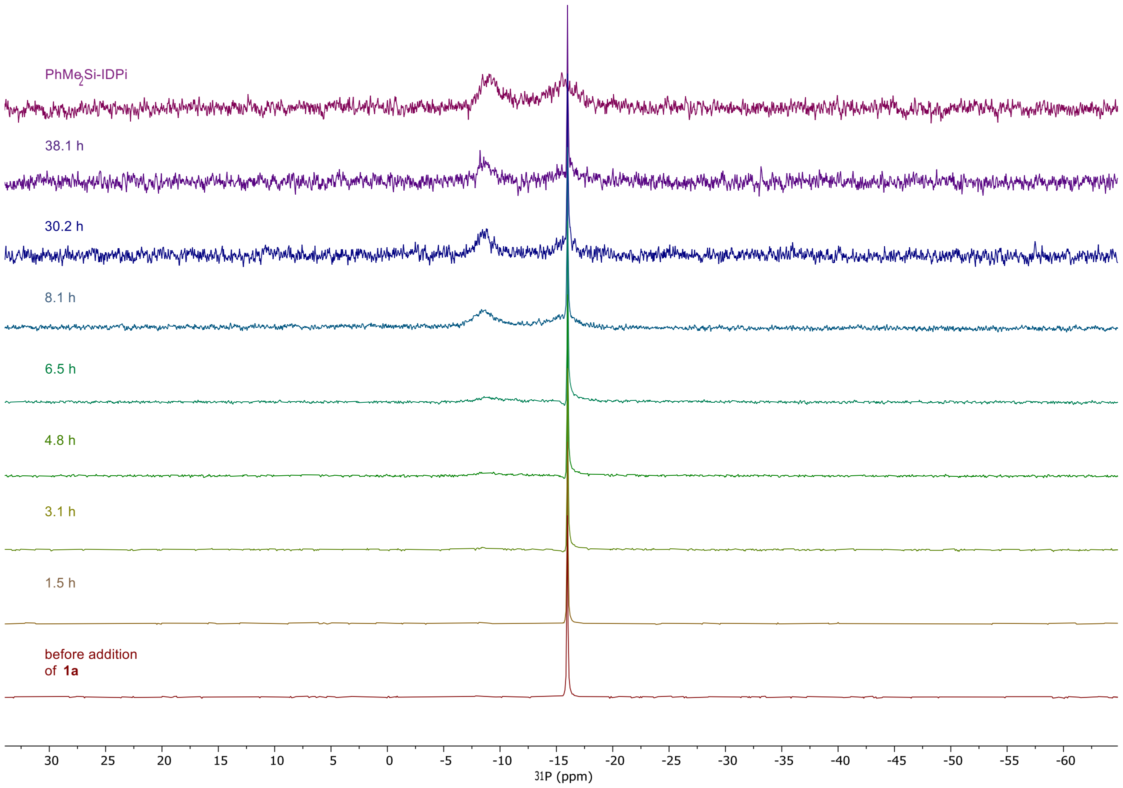

Figure S13*: Selected region of the stacked ^{31}P NMR spectra recorded during the reaction, along with the reference spectrum of silylated IDPi* ***S1a*** *(top trace).*

8.5. Experimental Probe of the Catalyst Regeneration Step

To simulate the catalyst regeneration step of the catalytic cycle (see Figure S4), silylation of IDPi **7b** was performed using dimethyl(2-methylallyl)(phenyl)silane[1]. Upon addition of benzoic acid, the regeneration of the protonated IDPi catalyst was monitored.

In a flame-dried 5 mm NMR tube under argon, IDPi **7b** (15.2 mg) was dissolved in dry CD_2Cl_2 (0.7 mL), and the initial ^1H and ^{31}P NMR spectra were recorded. Dimethyl(2-methylallyl)(phenyl)silane (0.8 µL, 0.5 eq. or 1.5 µL, 1.0 eq.) was then added sequentially, with NMR spectra recorded after each addition. Upon addition of an excess of silane (4.0 eq.), the NMR spectrometer was cooled stepwise to 233 K, with spectra recorded after each interval. At room temperature, a stock solution of benzoic acid (0.25 M in CD_2Cl_2, 2.8 µL, 1.0 eq.) was added incrementally, and NMR spectra were recorded after each addition.

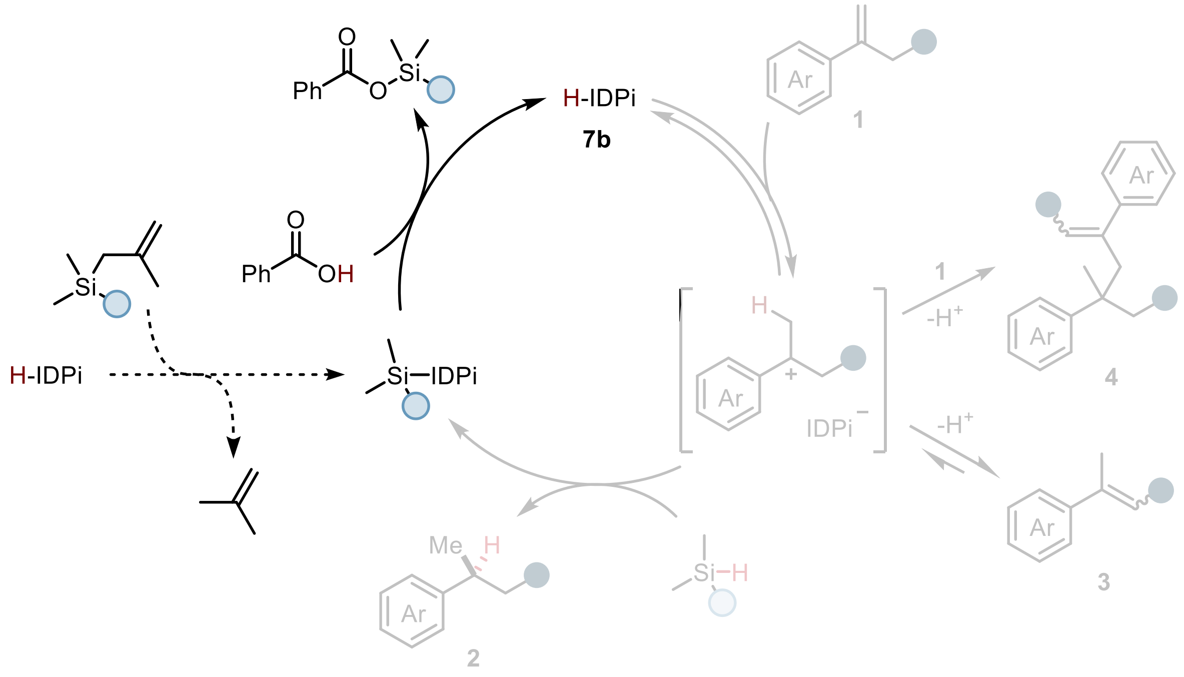

Figure S14: *Proposed catalytic cycle with emphasis on the catalyst regeneration step, along with an alternative method for generating the silylated catalyst intermediate, used in a mechanistic experiment to simulate part of the cycle.*

---

[1] Note: The choice of silane was based on availability in our laboratory. $PhMe_2SiH$ was used as the reducing agent for many of our substrates (8 out of 17 examples). We do not expect this variation to affect the qualitative outcome of the study and therefore consider the results representative of our ionic hydrogenation methodology.

Upon addition of dimethyl(2-methylallyl)(phenyl)silane to **7b**, broad signals in the ^{1}H NMR spectrum were observed. The sharp singlet in the ^{31}P spectrum, originally caused by IDPi **7b**, disappeared, giving rise to three broad signals.

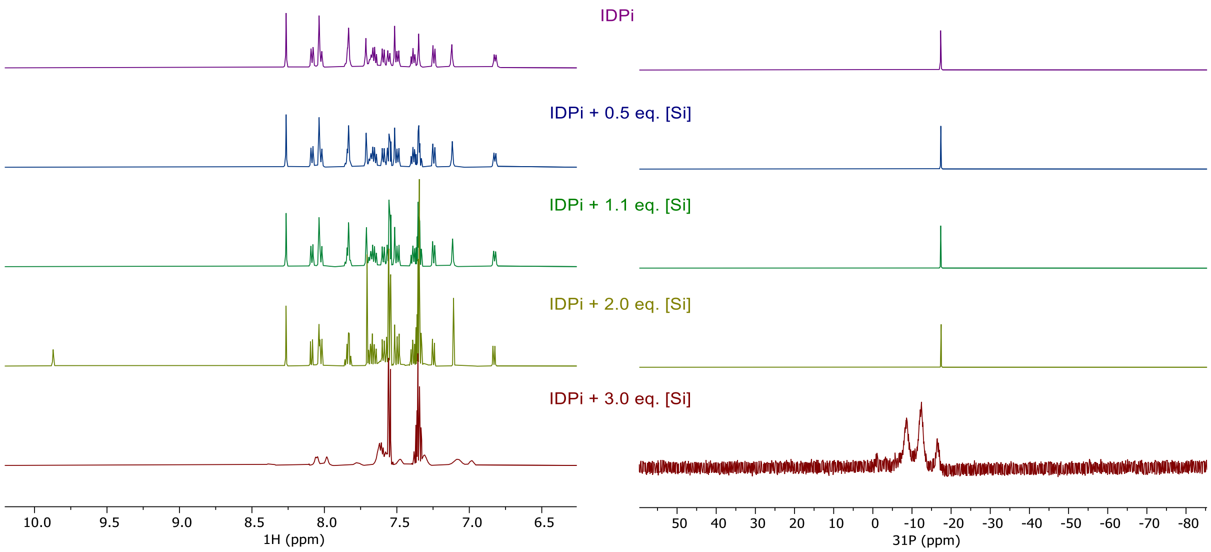

Figure S15: *Stacked ^{1}H and ^{31}P NMR spectra of IDPi* ***7b*** *upon sequential addition of increasing amounts of dimethyl(2-methylallyl)(phenyl)silane at room temperature.*

Cooling the NMR tube to 233 K within the spectrometer did not yield sharp signals; however, partial resolution was observed at 253 K, resulting in a pattern resembling a doublet of doublets, suggesting the presence of the silylated catalyst.

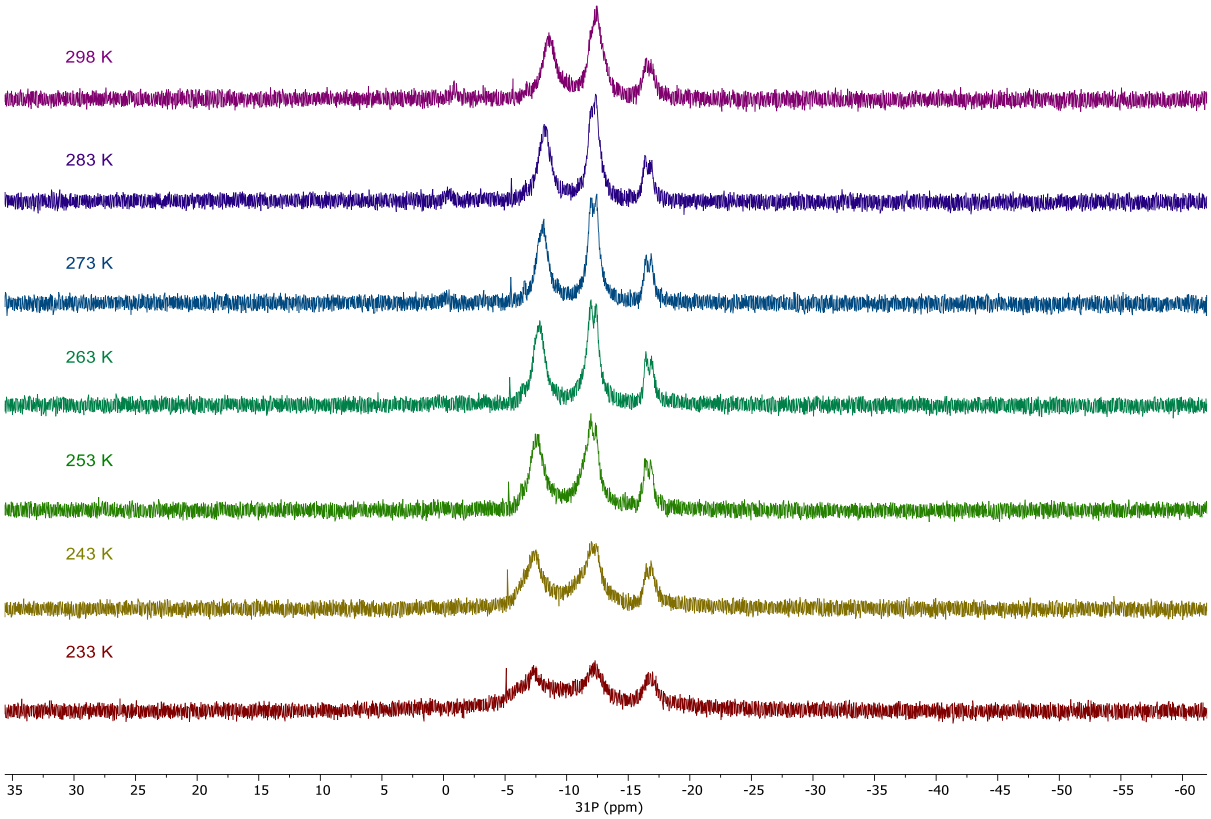

Figure S16: *Stacked ^{31}P NMR spectra of silylated IDPi species recorded at progressively lower temperatures, reaching 233 K.*

Upon addition of benzoic acid to the mixture, the protonated IDPi was immediately regenerated, as confirmed by both the ^{1}H and ^{31}P spectra. Simultaneously, the formation of $PhCO_2SiPhMe_2$ was observed, as evidenced by ^{1}H NMR data.[12] The experiment suggests that catalyst turnover is rapid.

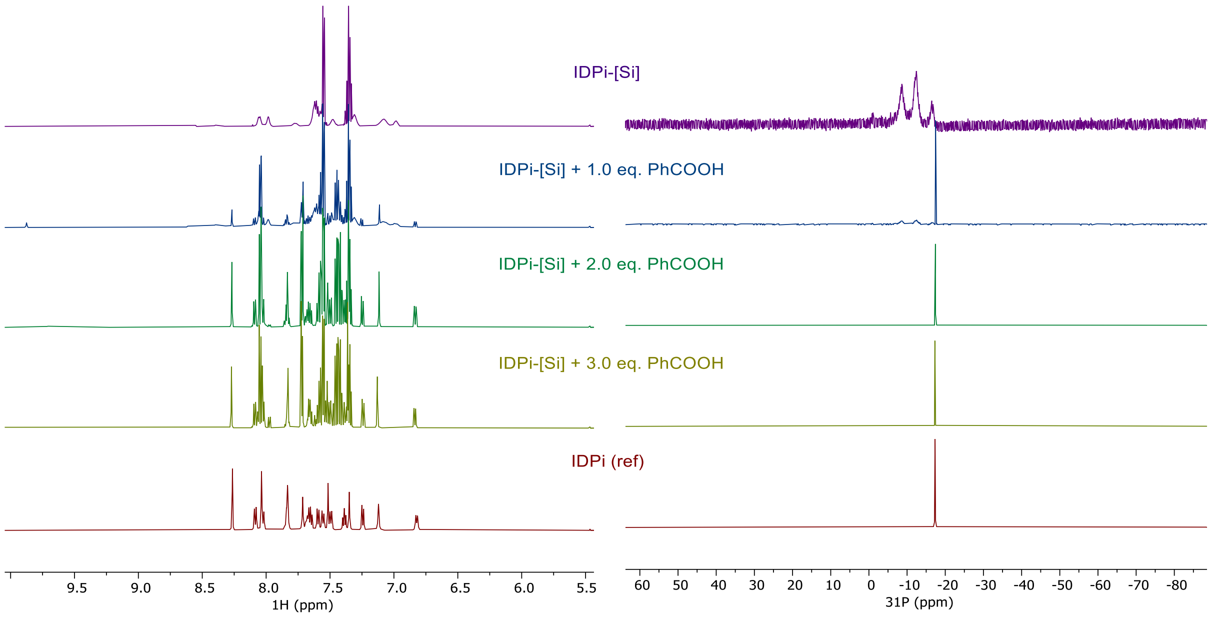

Figure S17: *Stacked* ^{1}*H and* ^{31}*P NMR spectra of silylated IDPi* ***7b*** *upon sequential addition of increasing amounts of benzoic acid at room temperature.*

8.6. Non-Linear Effect (NLE) Study

As the reaction is heterogeneous with respect to the catalyst (and benzoic acid) in the reaction medium, the presence of a non-linear effect was investigated. According to **GP1**, NLE experiments were set up using styrene **1a**, $EtMe_2SiH$ (10 eq.), benzoic acid (1.2 eq.) and scalemic mixtures of IDPi **7b** (2 mol%) at –20 °C for 4–5 d. The enantiomeric excess of the product **2b** was subsequently plotted against the enantiomeric excess of the catalyst.

ee (7b)	yield (2a)	ee (2a)
2%	1%	23%
4%	1%	34%
14%	10%	79%
21%	15%	84%
37%	33%	88%
61%	53%	89%
80%	65%	90%
99%	59%	90%

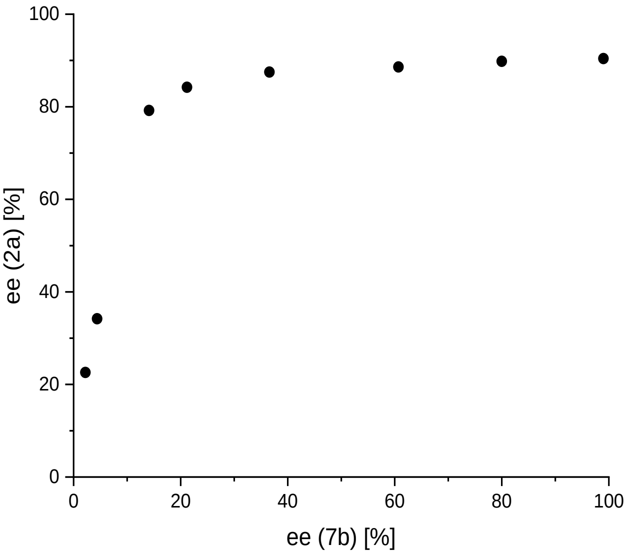

Figure S18: *Investigation of non-linear effects in the heterogeneous (neat in 10 eq.* $EtMe_2SiH$*) catalytic reaction of* ***1a*** *with scalemic mixtures of* ***7b****.*

The plot demonstrates a strong positive non-linear effect ((+)-NLE). In addition, the NMR data show that the enantiopure catalyst produced a good yield of **2a**, which decreases as the optical purity of the catalyst diminishes.

To probe the potential origin of the (+)-NLE, experiments with scalemic mixtures of **7b** (18%, 36%, 61% *ee*) were conducted as previously, using $EtMe_2SiH$ (10.0 eq.) under neat reaction conditions on a 0.05 mmol scale. After 3 d, the mixture was centrifuged, and the liquid phase was separated using a Hamilton syringe. The solid phase was dissolved in DCM, and both fractions were purified by preparative thin-layer chromatography (20% EtOAc, hexanes) to isolate **7b**. The enantiomeric excess of the catalyst in both the liquid and solid phases was determined by chiral HPLC analysis.

For the employed scalemic mixtures with 18%, 36%, and 61% *ee*, the enantiomeric excess of **7b** in solution was larger than 90% in all three cases, whereas the catalyst in the solid phases exhibited

lower optical purity (see Figure S19). This phenomenon may be attributed to differences in the solubility of heterochiral and homochiral IDPi complexes, a trend that has previously been observed for amino acids[13,14] and chiral phosphoric acids[15].

These findings were corroborated by NLE experiments conducted under homogeneous reaction conditions (in $CHCl_3$, 0.25 M, at room temperature). In contrast to the hetereogeneous catalytic reaction, a linear relationship was found (see Figure S20). Additionally, the reactivity of **1a** was found to be comparable across the different scalemic mixtures.

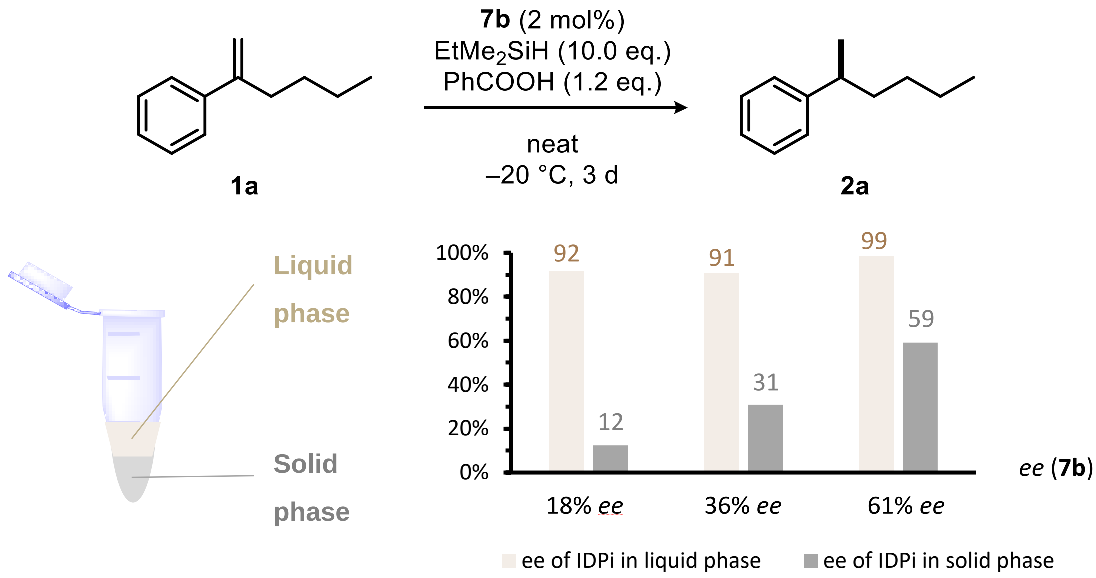

Figure S19: *Analysis of the enantiomeric excess of **7b** in solution and solid phases.*

ee (7b)	yield (2a)	ee (2a)
5%	41%	11%
9%	51%	18%
35%	53%	31%
62%	51%	44%
83%	53%	59%
99%	51%	71%

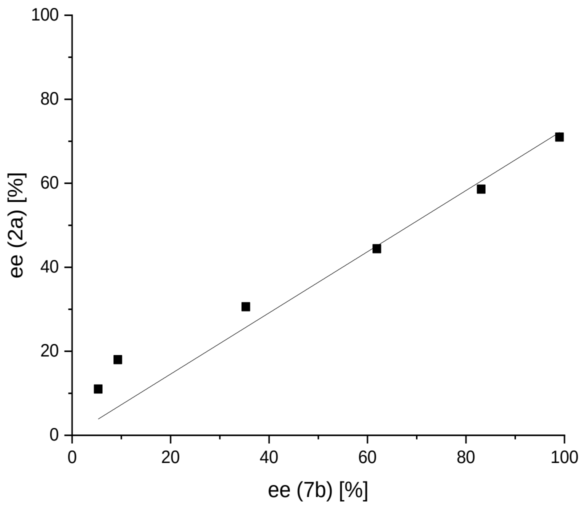

Figure S20: *Investigation of non-linear effects in the homogeneous (with 5 eq. $EtMe_2SiH$, in $CHCl_3$, c = 0.25 M at room temperature) catalytic reaction of **1a** with scalemic mixtures of **7b**.*

9. NMR Spectra

^{1}H NMR spectrum of **1a**

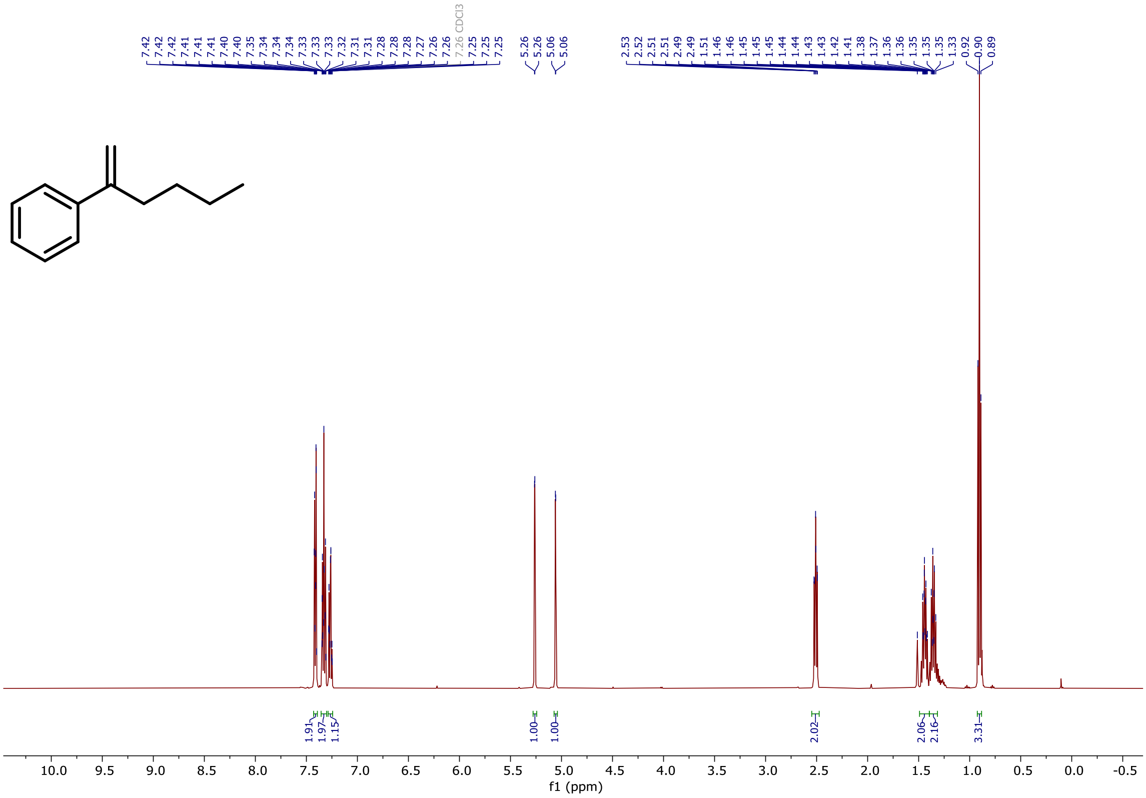

^{13}C NMR spectrum of **1a**

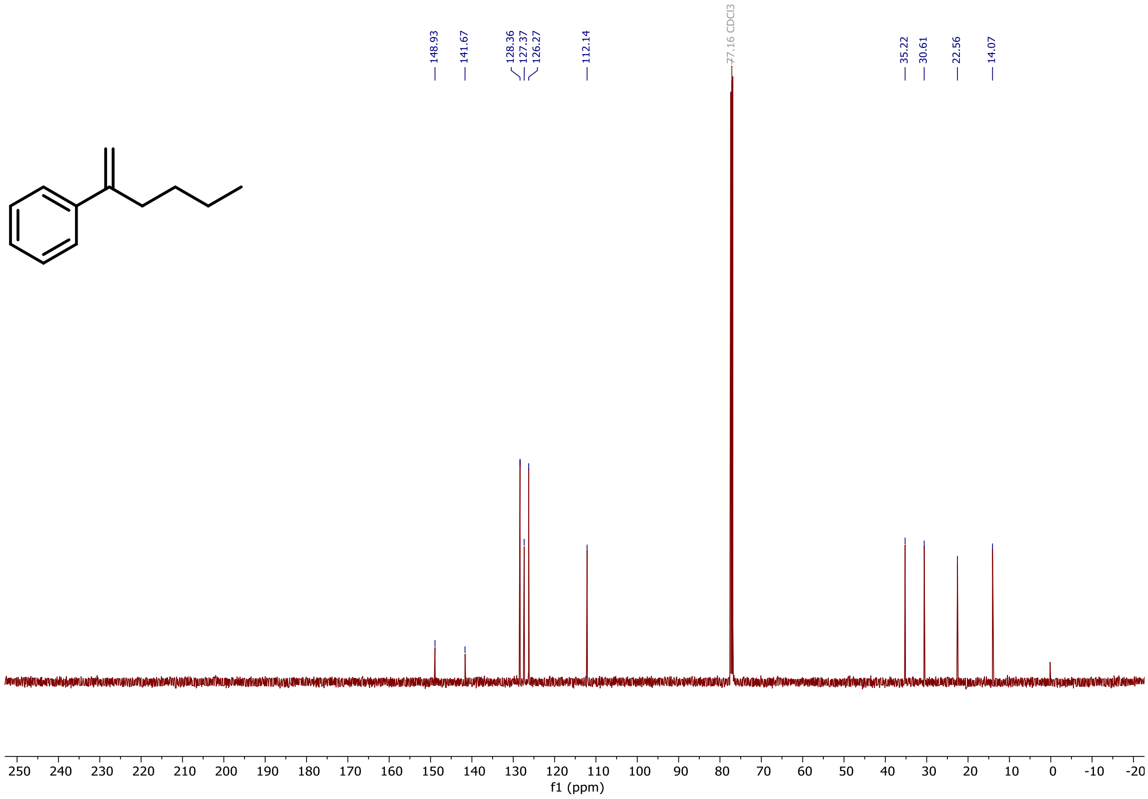

^{1}H NMR spectrum of **1b**

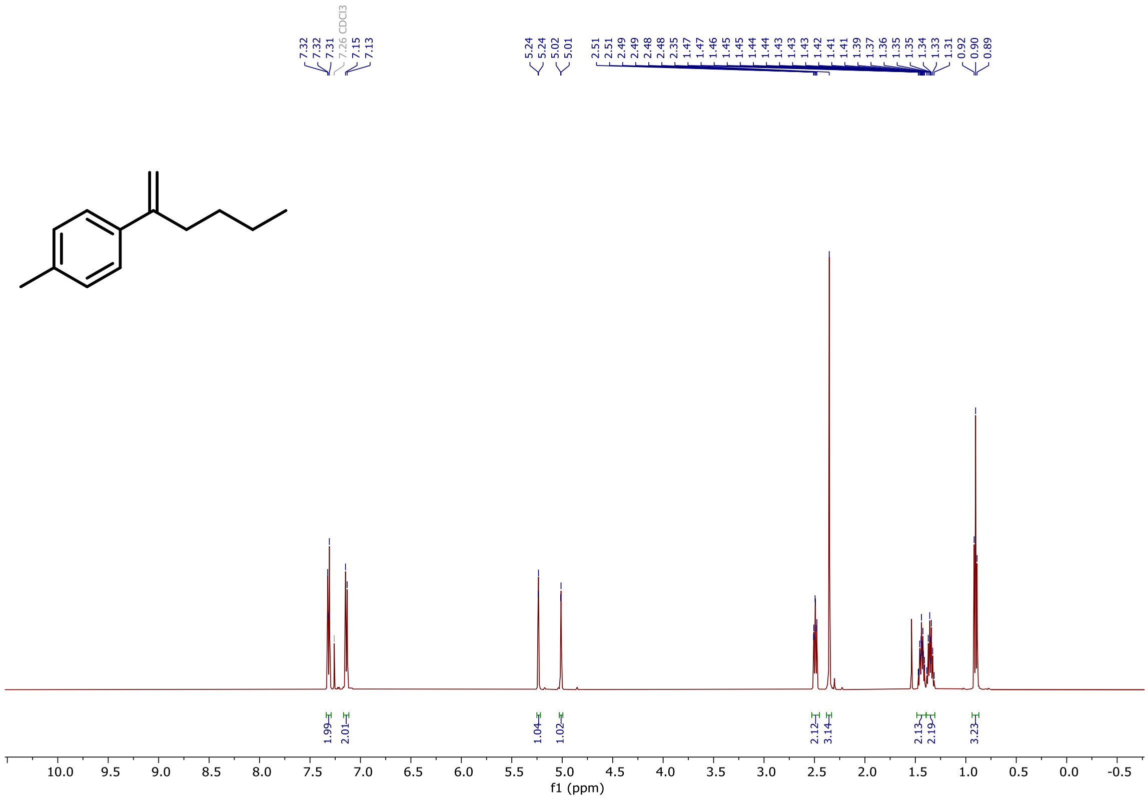

^{13}C NMR spectrum of **1b**

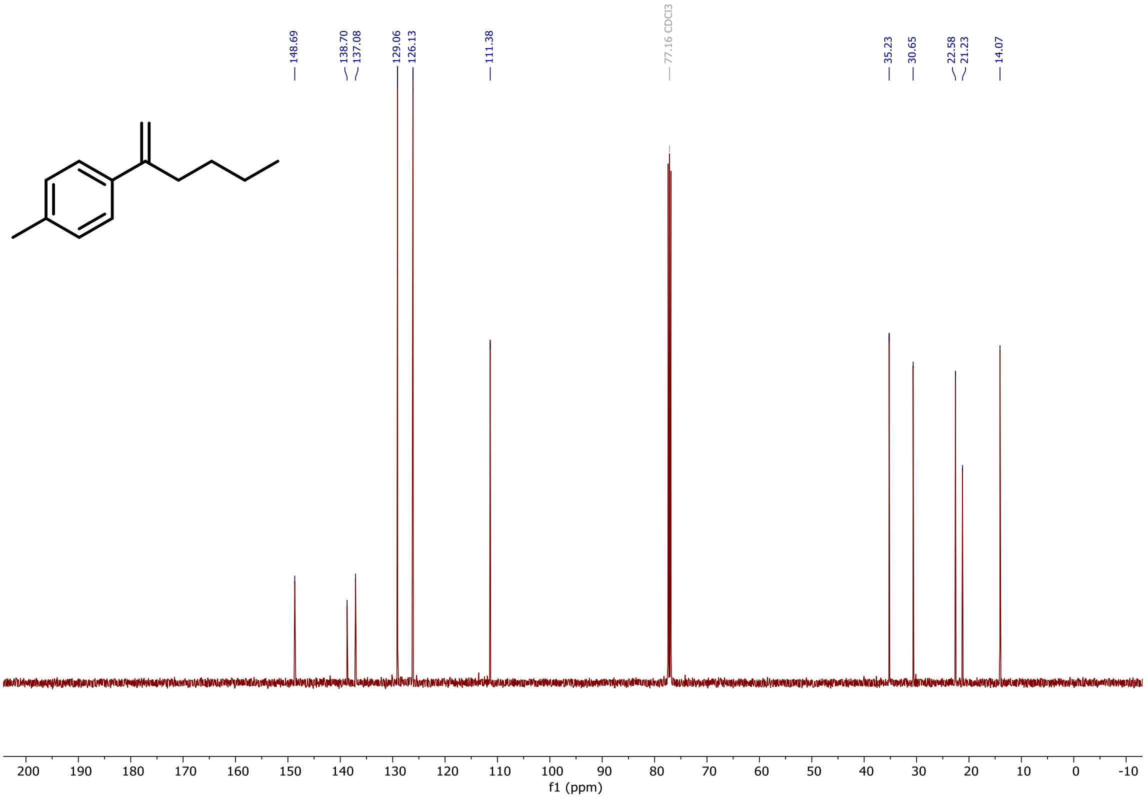

^{1}H NMR spectrum of **1c**

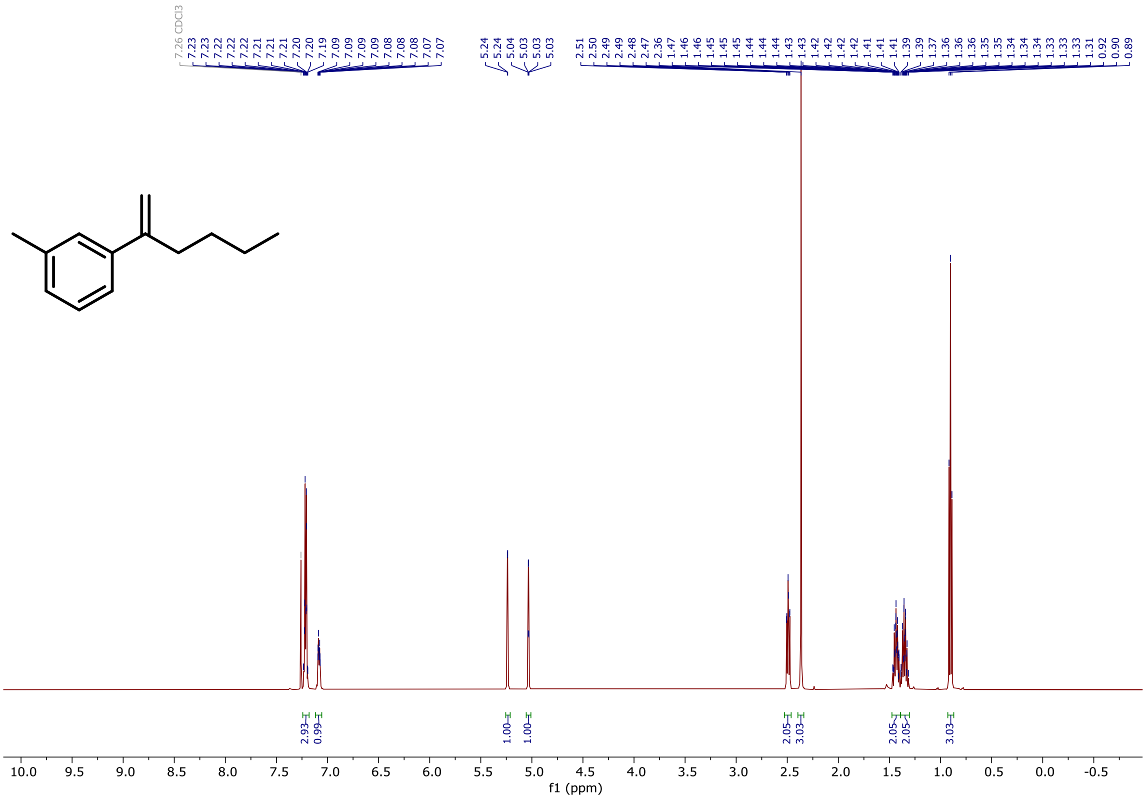

^{13}C NMR spectrum of **1c**

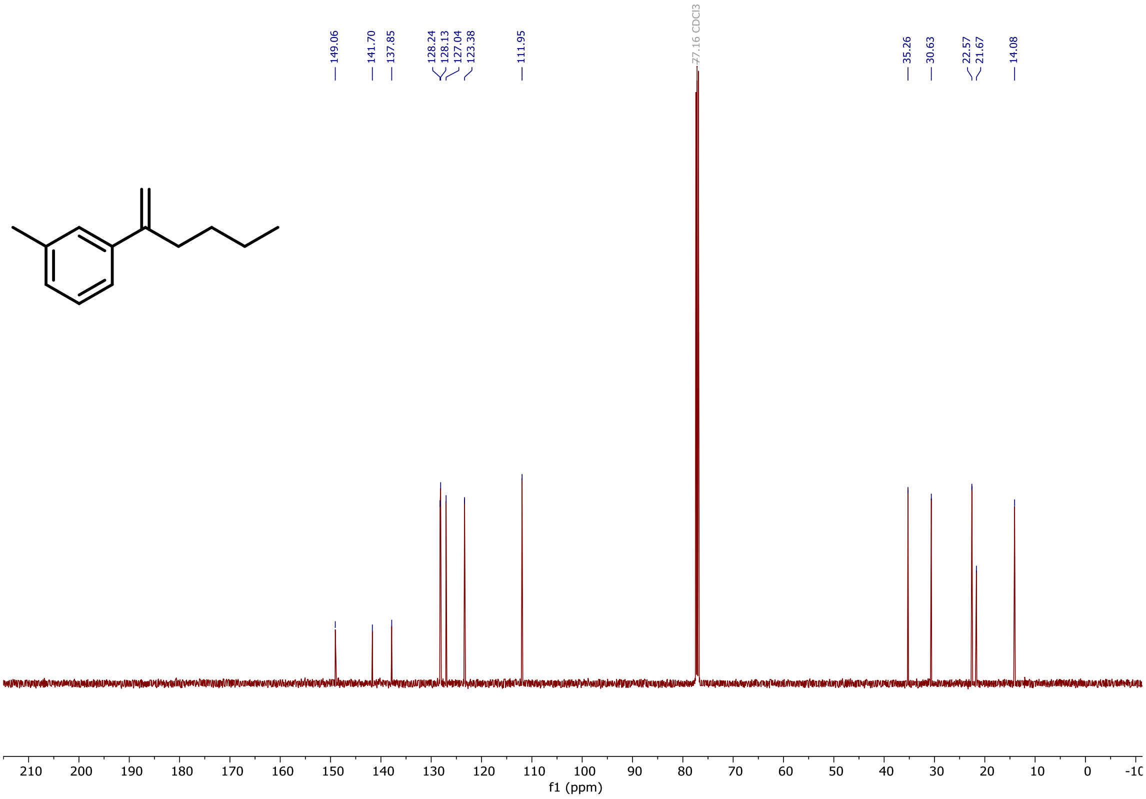

^{1}H NMR spectrum of **1d**

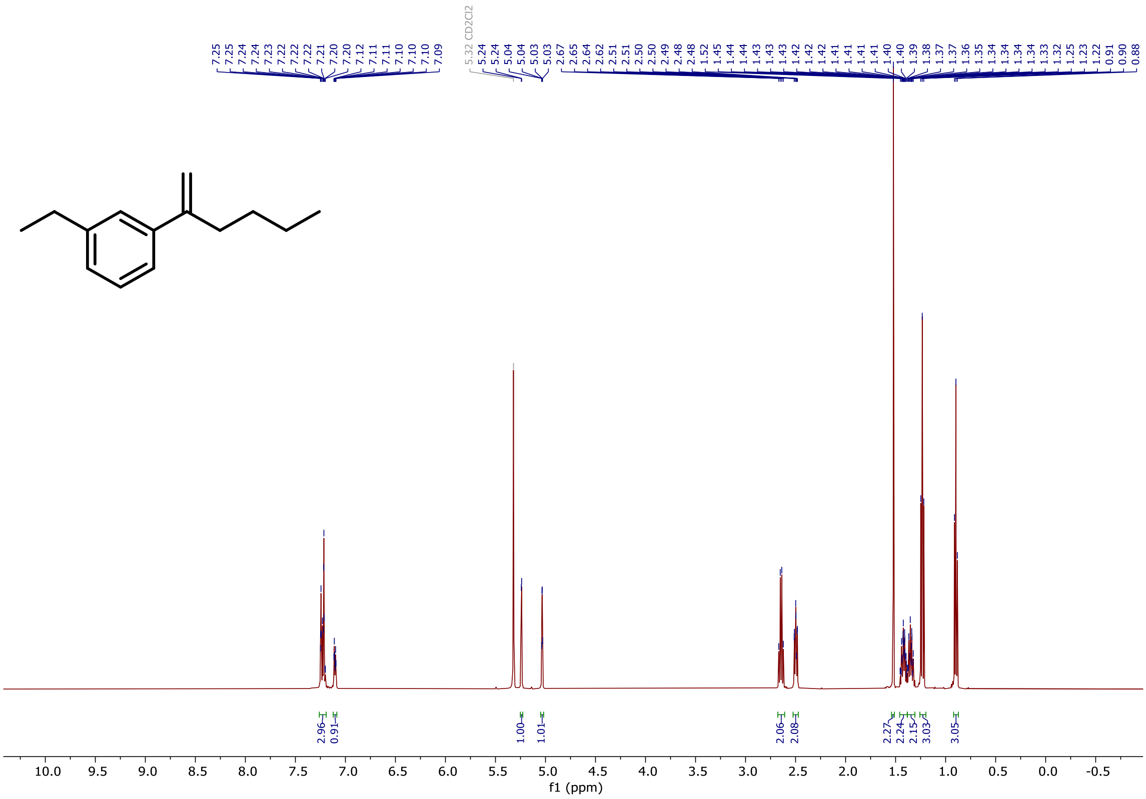

^{13}C NMR spectrum of **1d**

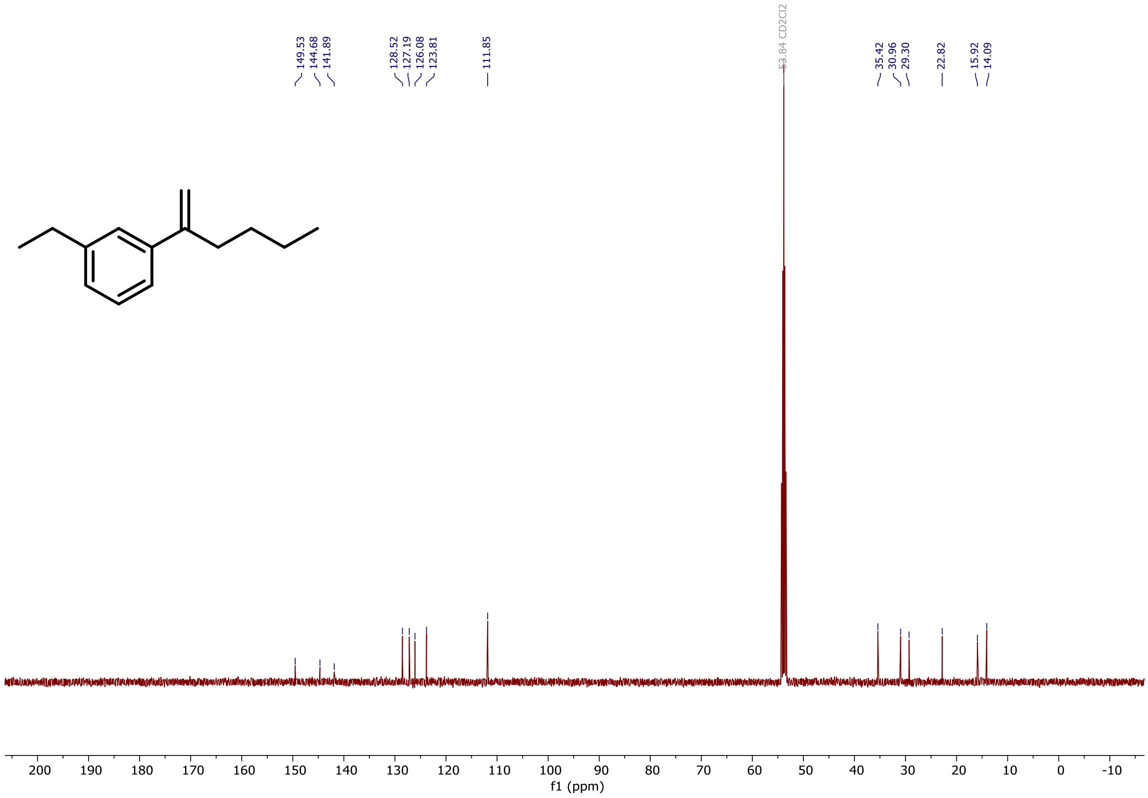

^{1}H NMR spectrum of **1e**

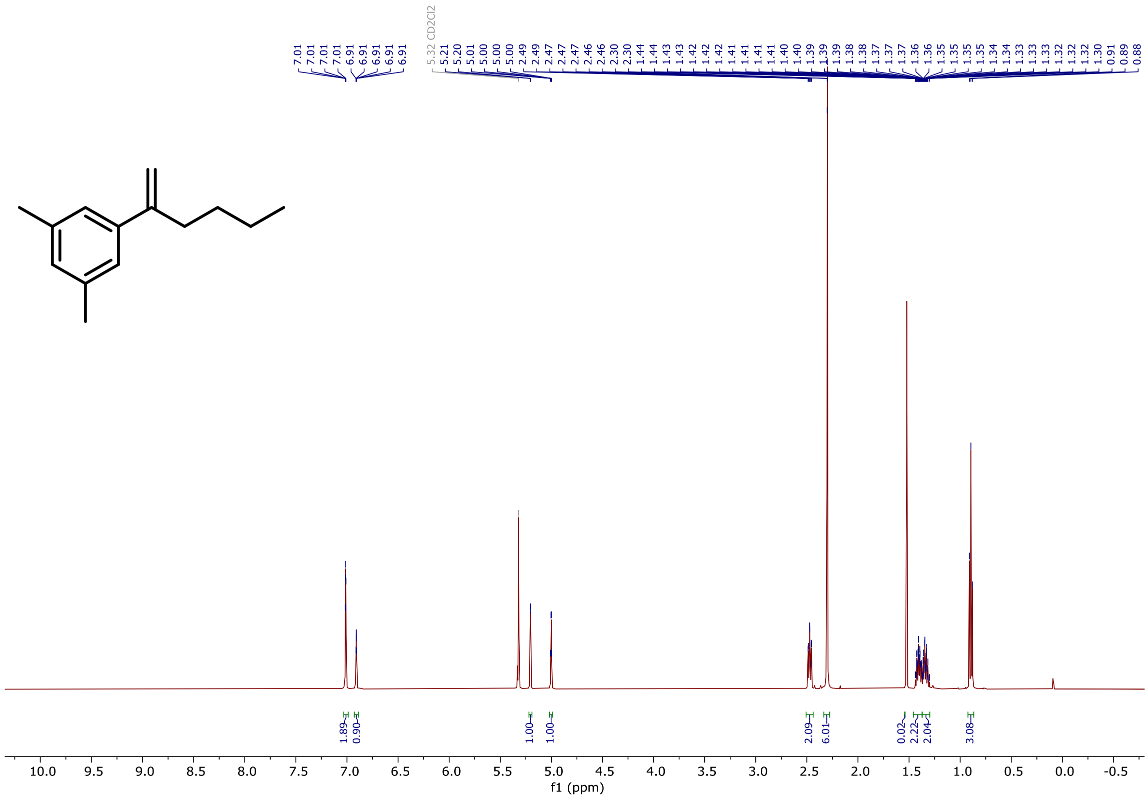

^{13}C NMR spectrum of **1e**

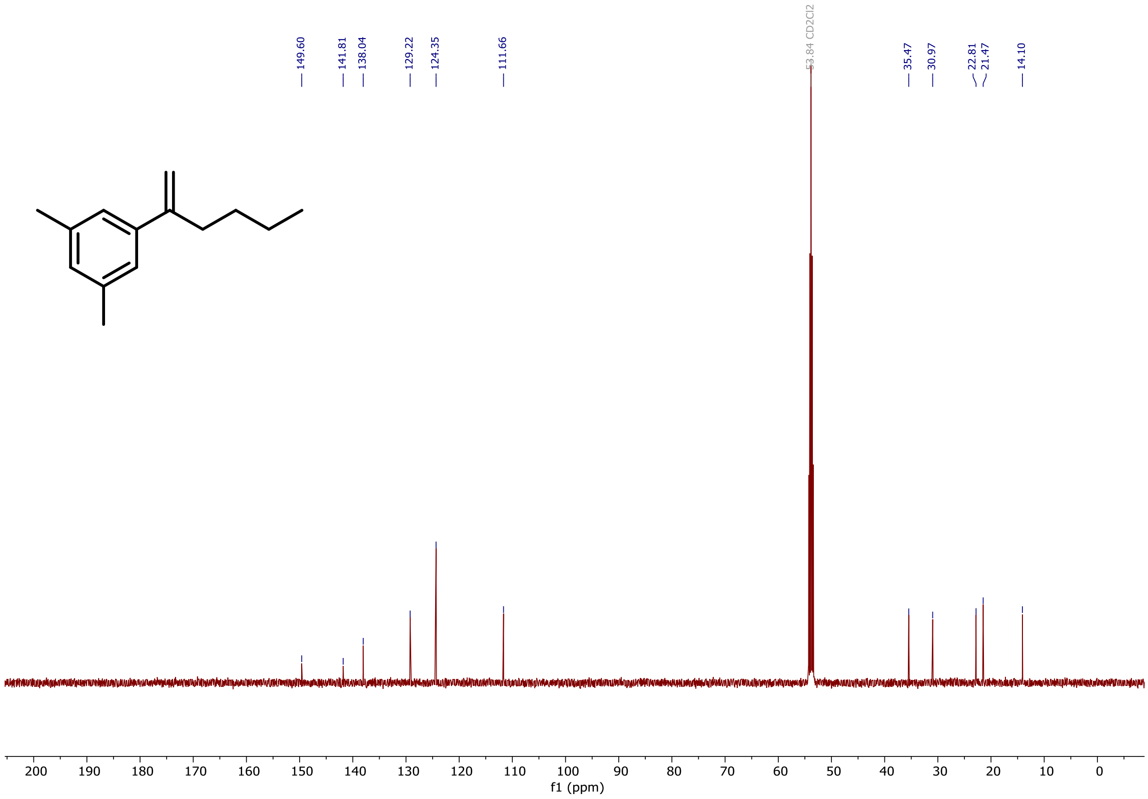

^{1}H NMR spectrum of **1f**

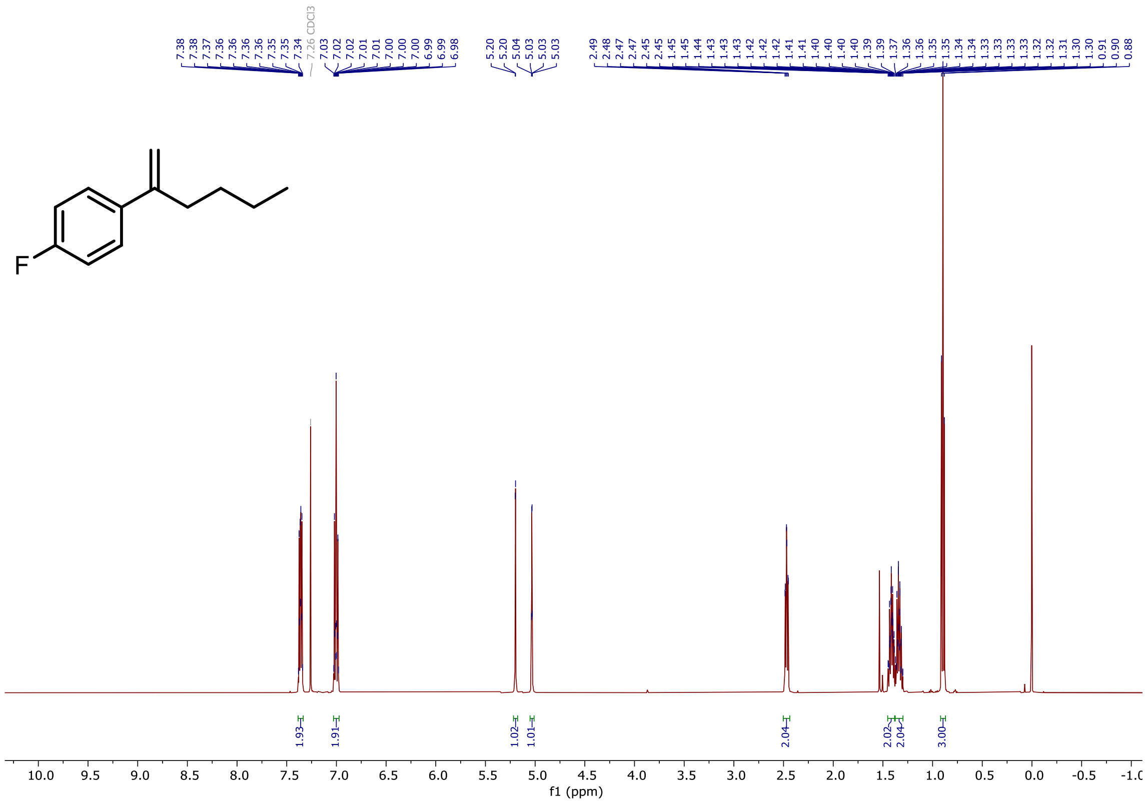

^{19}F NMR spectrum of **1f**

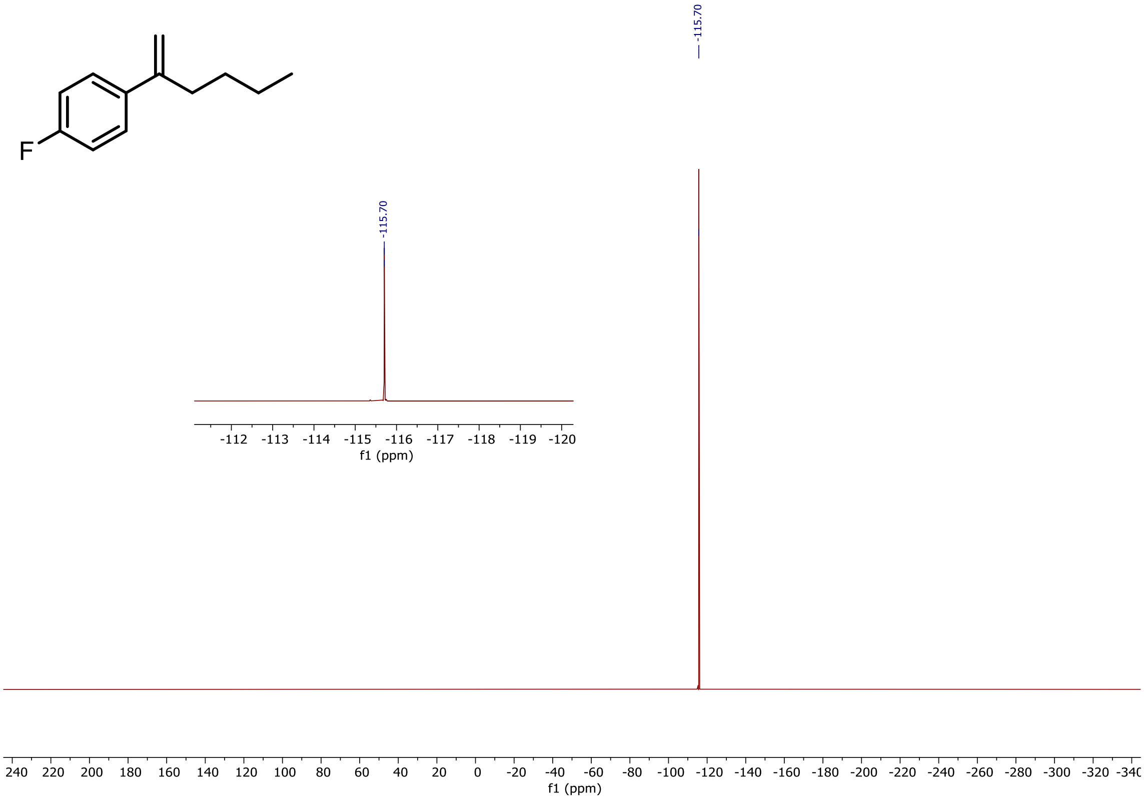

^{13}C NMR spectrum of **1f**

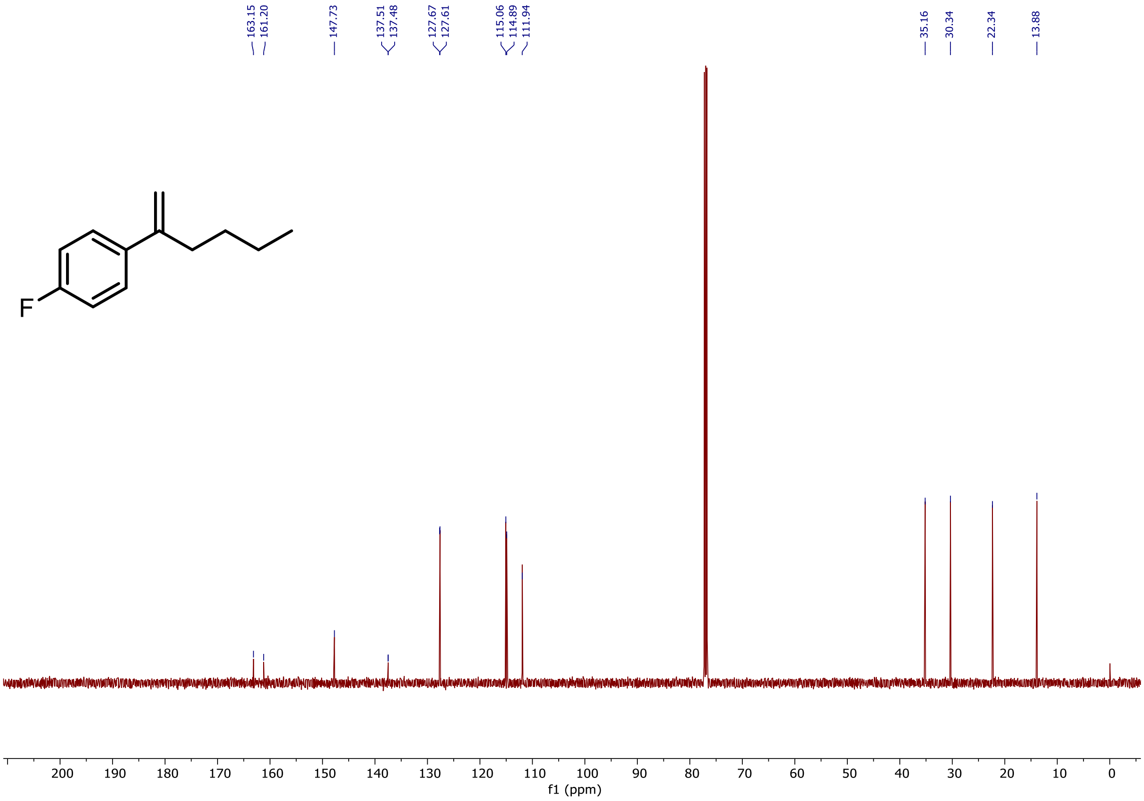

^{1}H NMR spectrum of **1g**

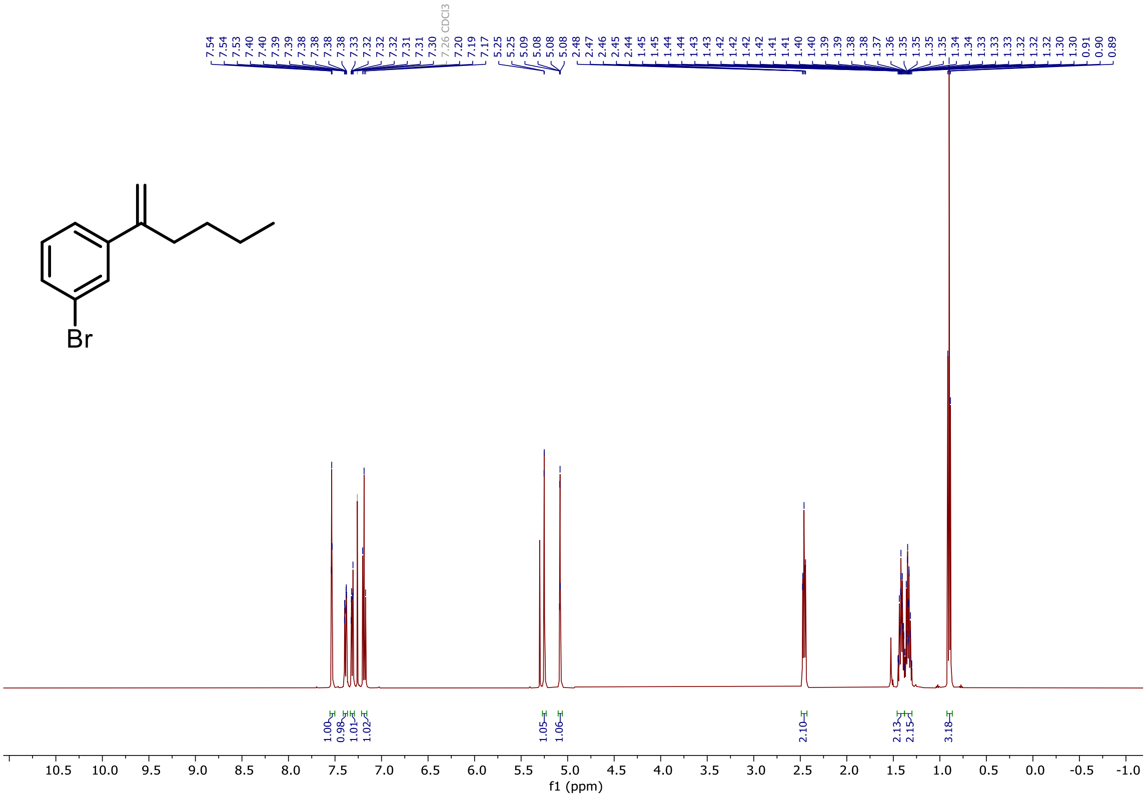

^{13}C NMR spectrum of **1g**

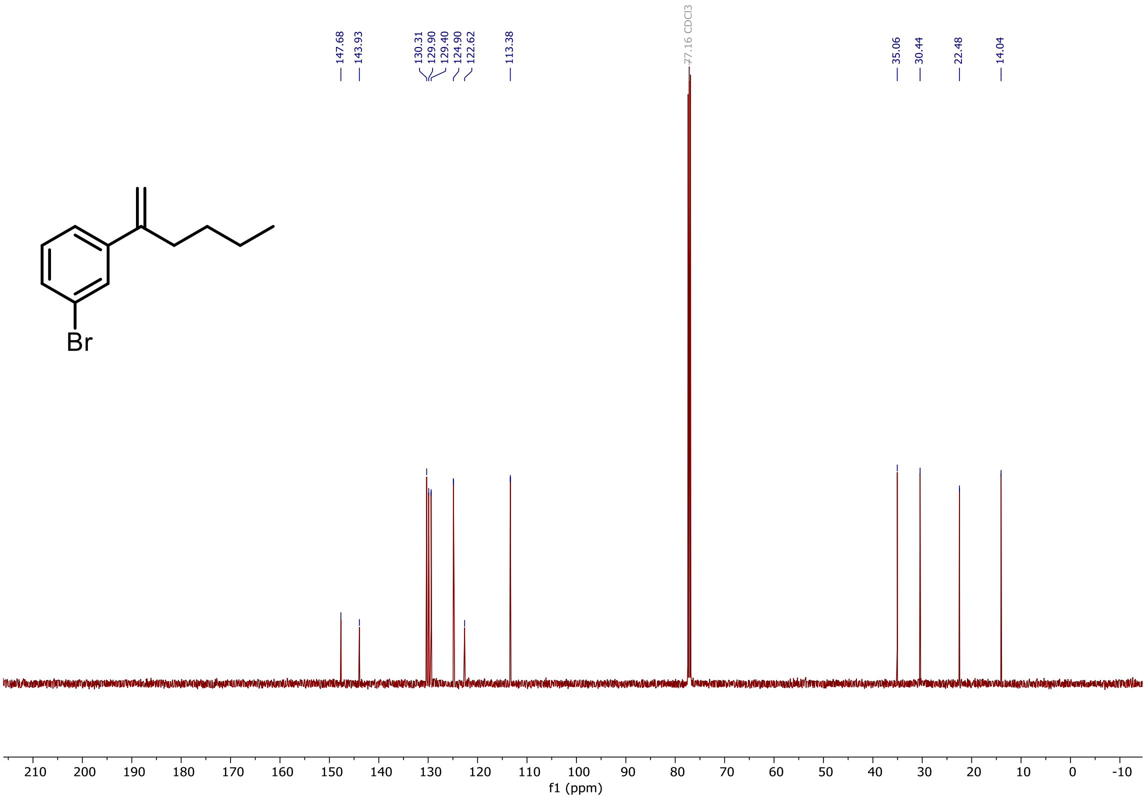

^{1}H NMR spectrum of **1h**

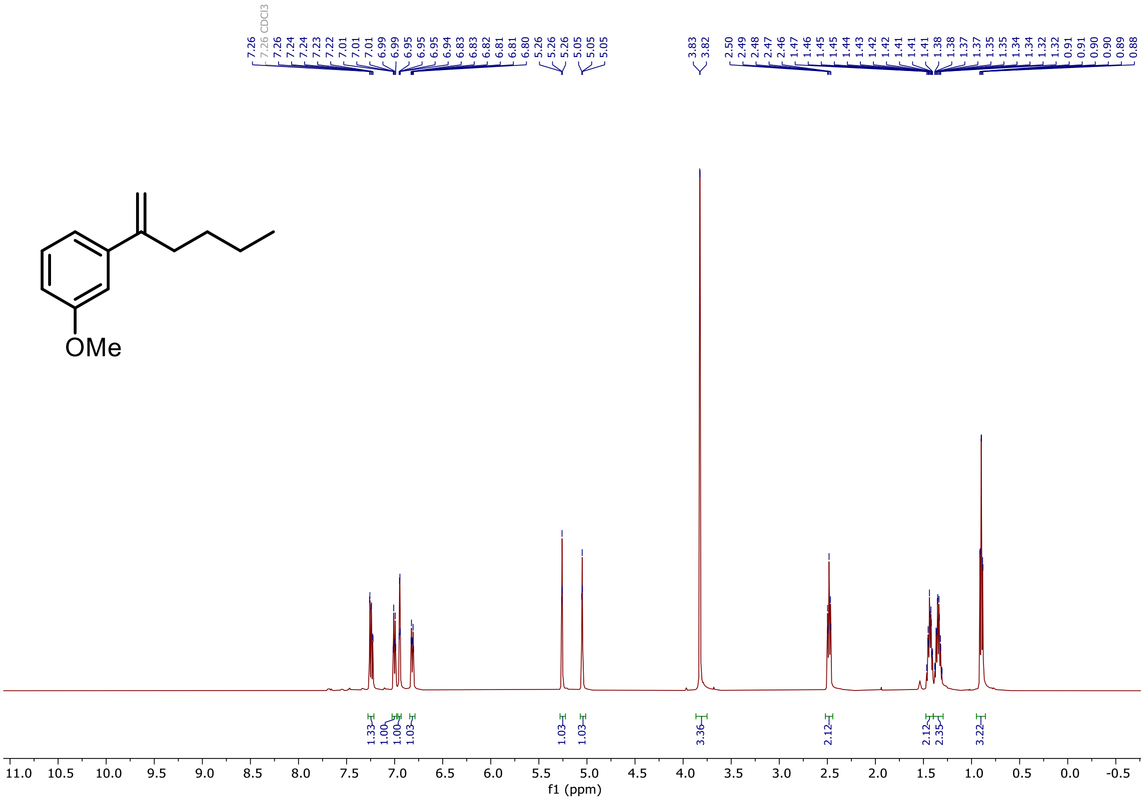

^{13}C NMR spectrum of **1h**

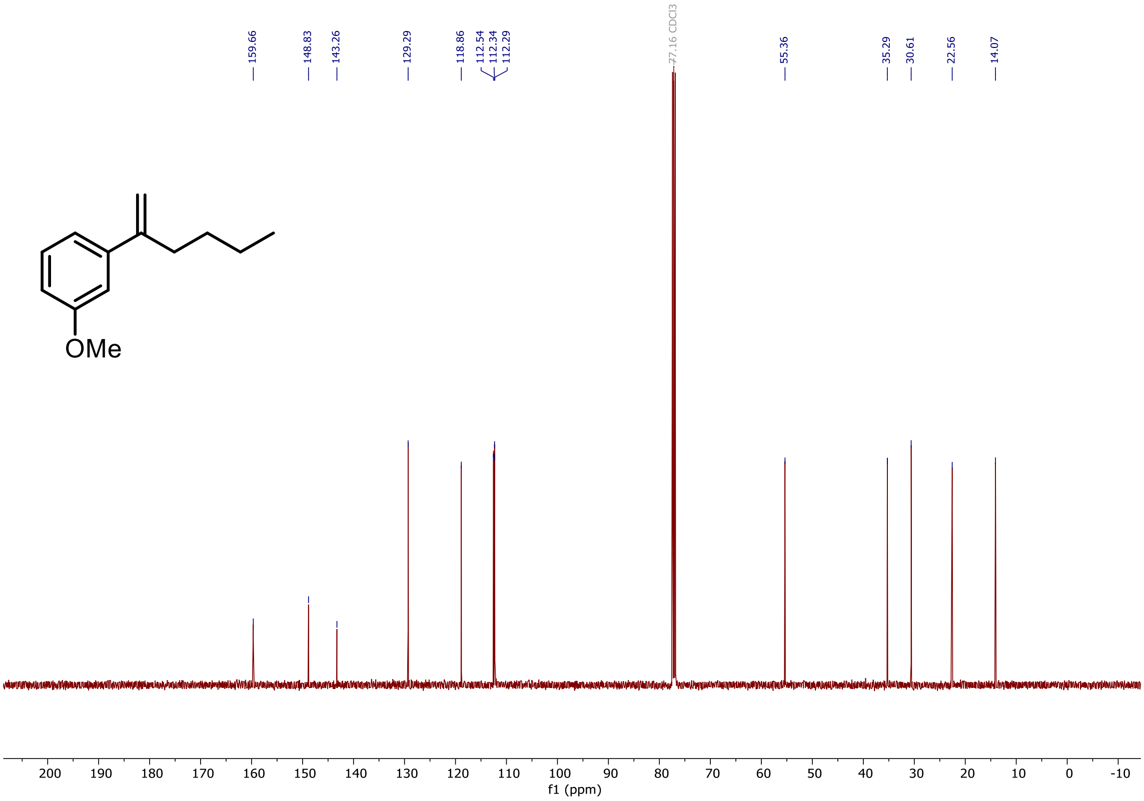

^{1}H NMR spectrum of **1j**

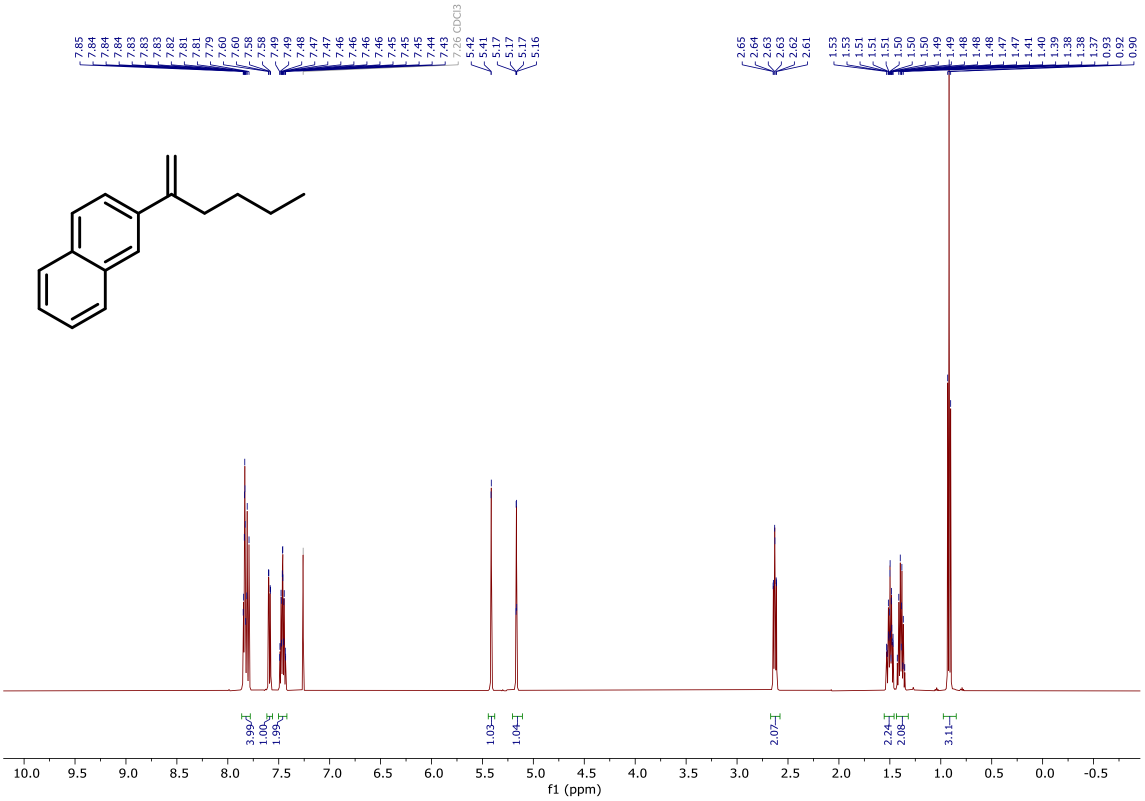

^{13}C NMR spectrum of **1j**

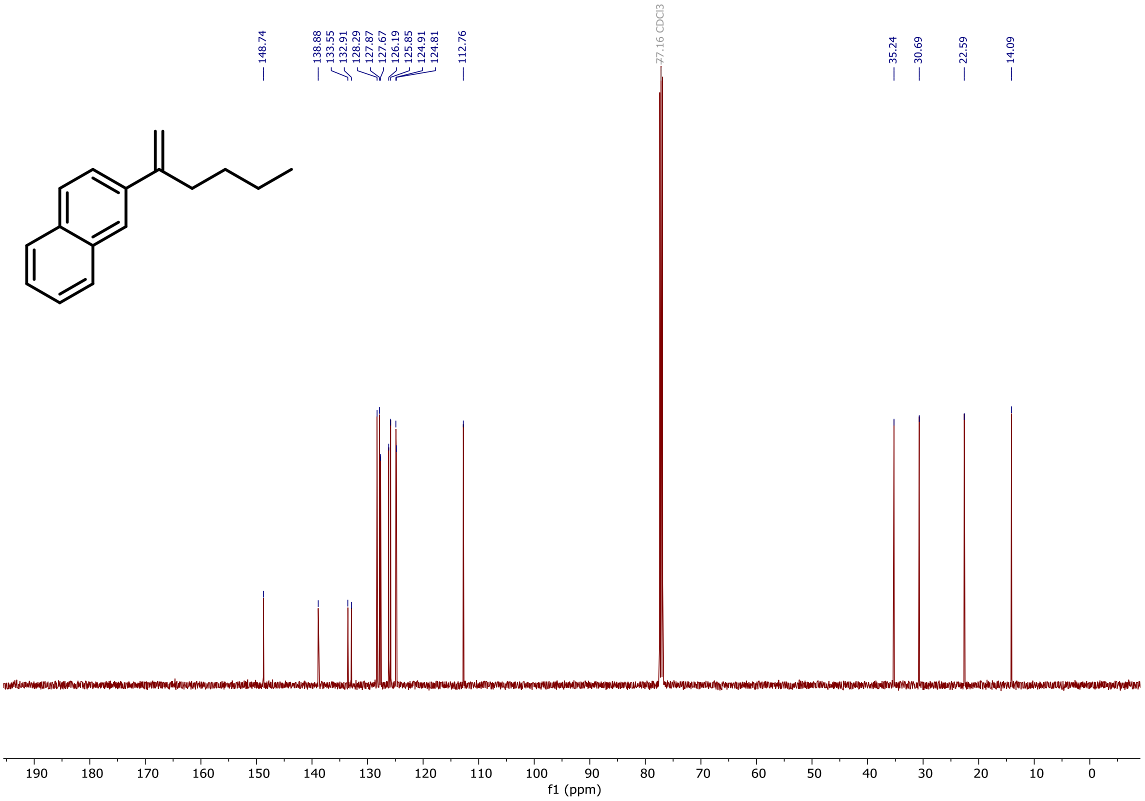

^{1}H NMR spectrum of **S3**

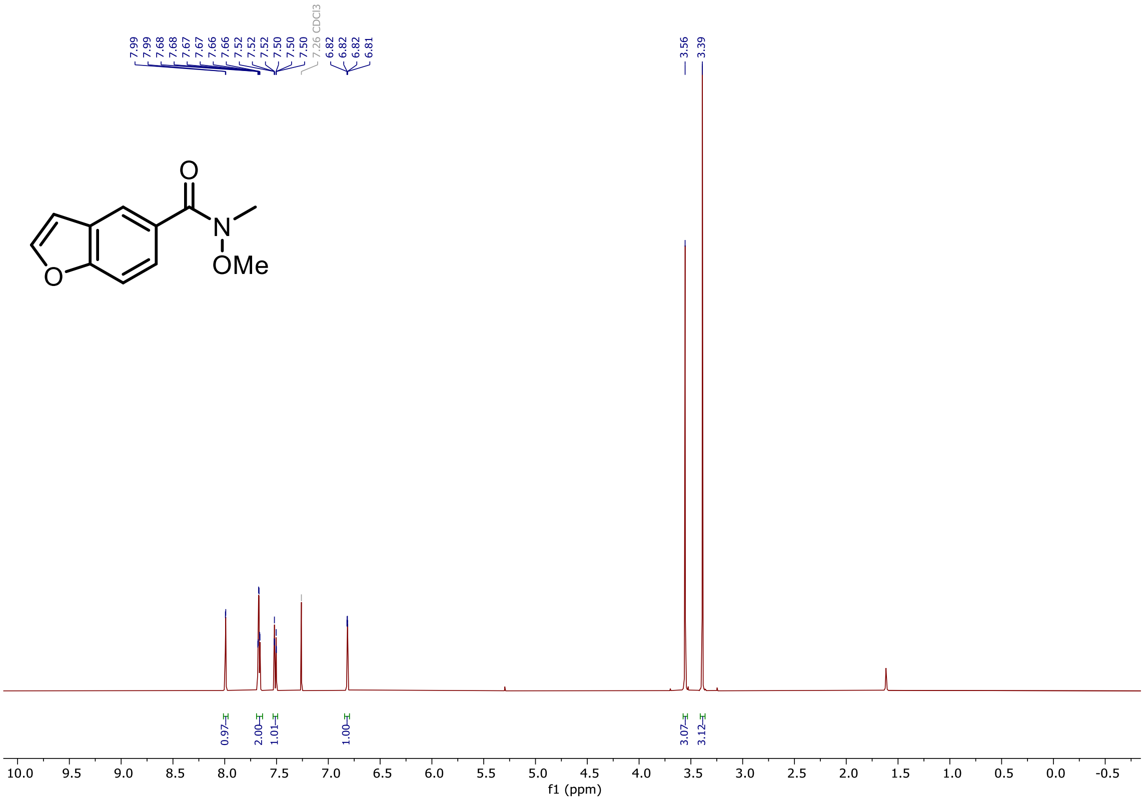

^{13}C NMR spectrum of **S3**

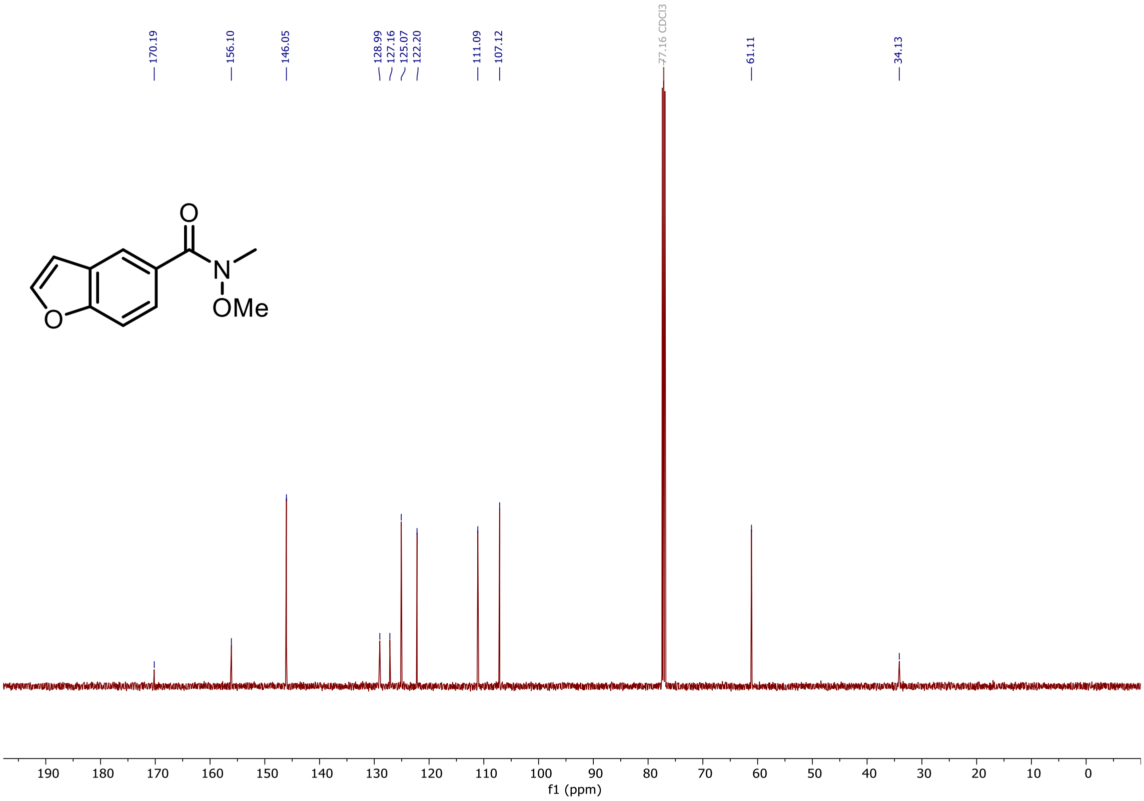

^{1}H NMR spectrum of **S4**

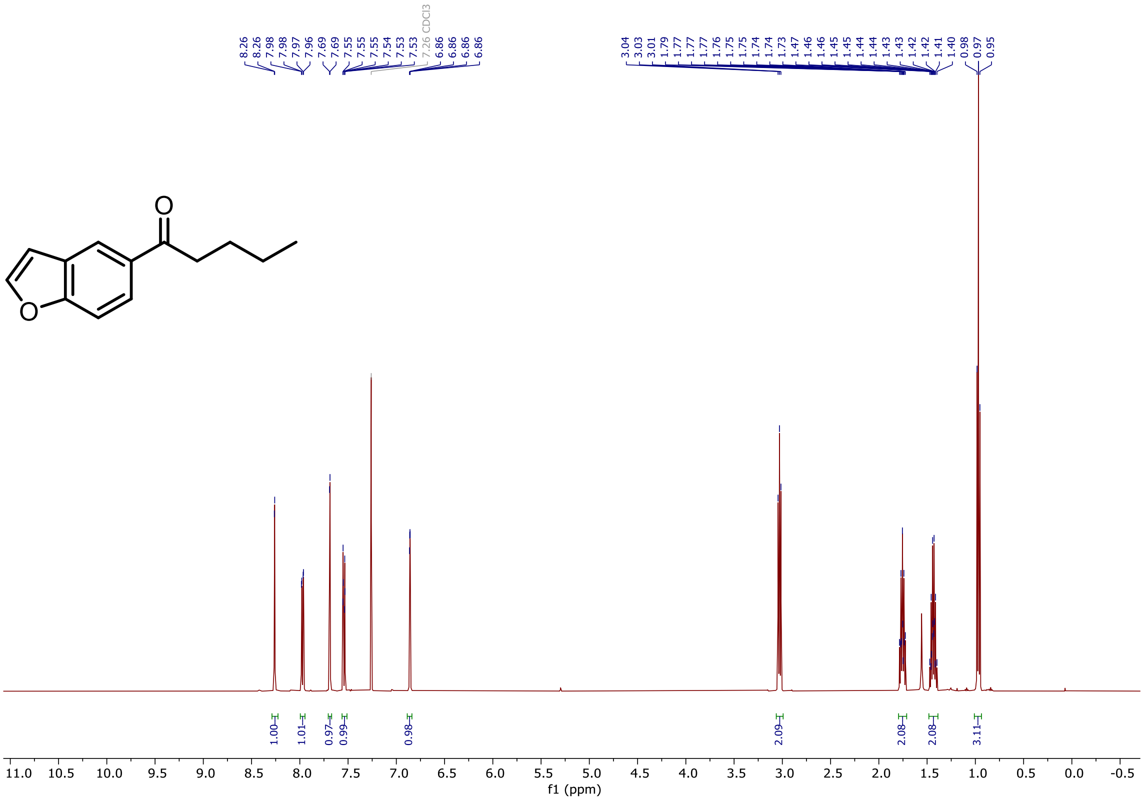

^{13}C NMR spectrum of **S4**

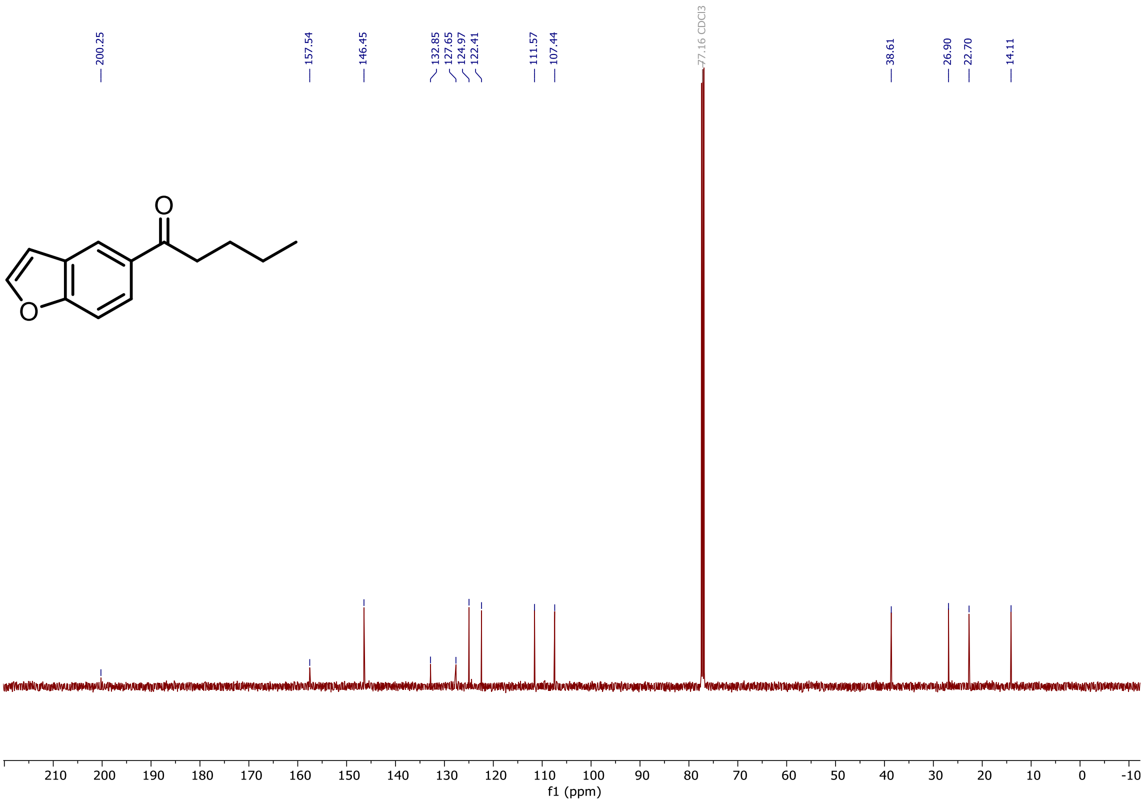

^{1}H NMR spectrum of **1k**

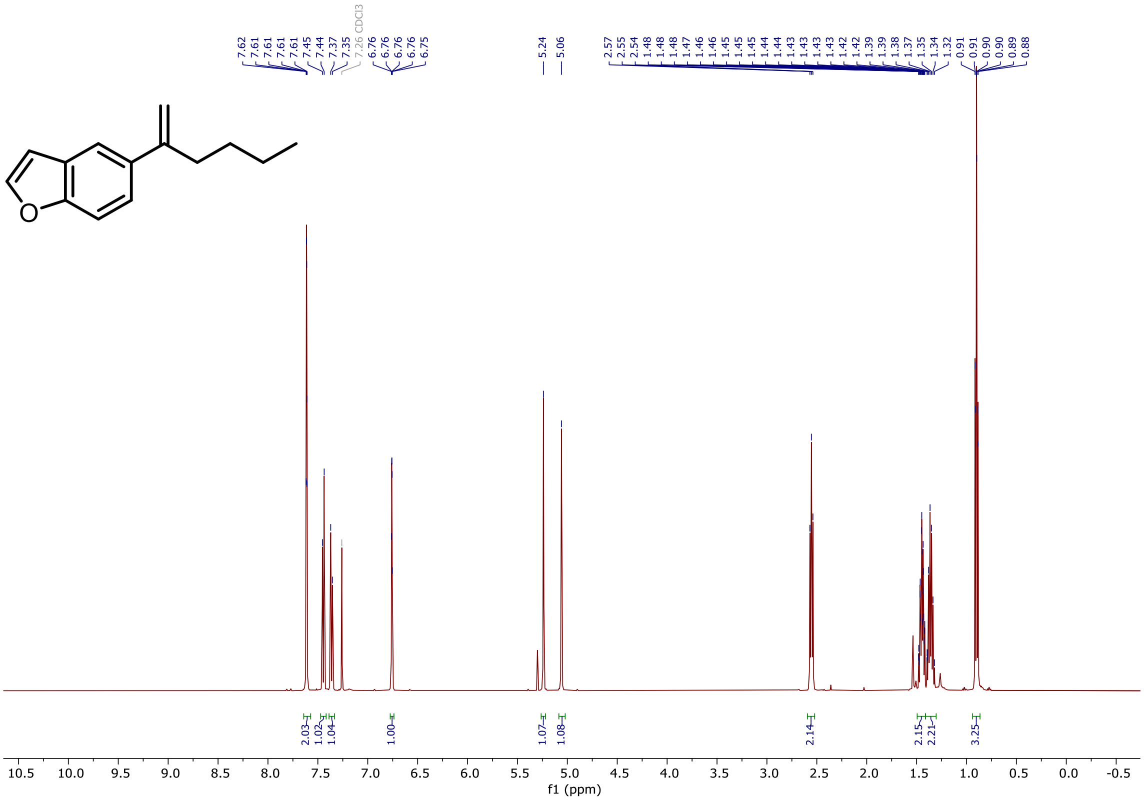

^{13}C NMR spectrum of **1k**

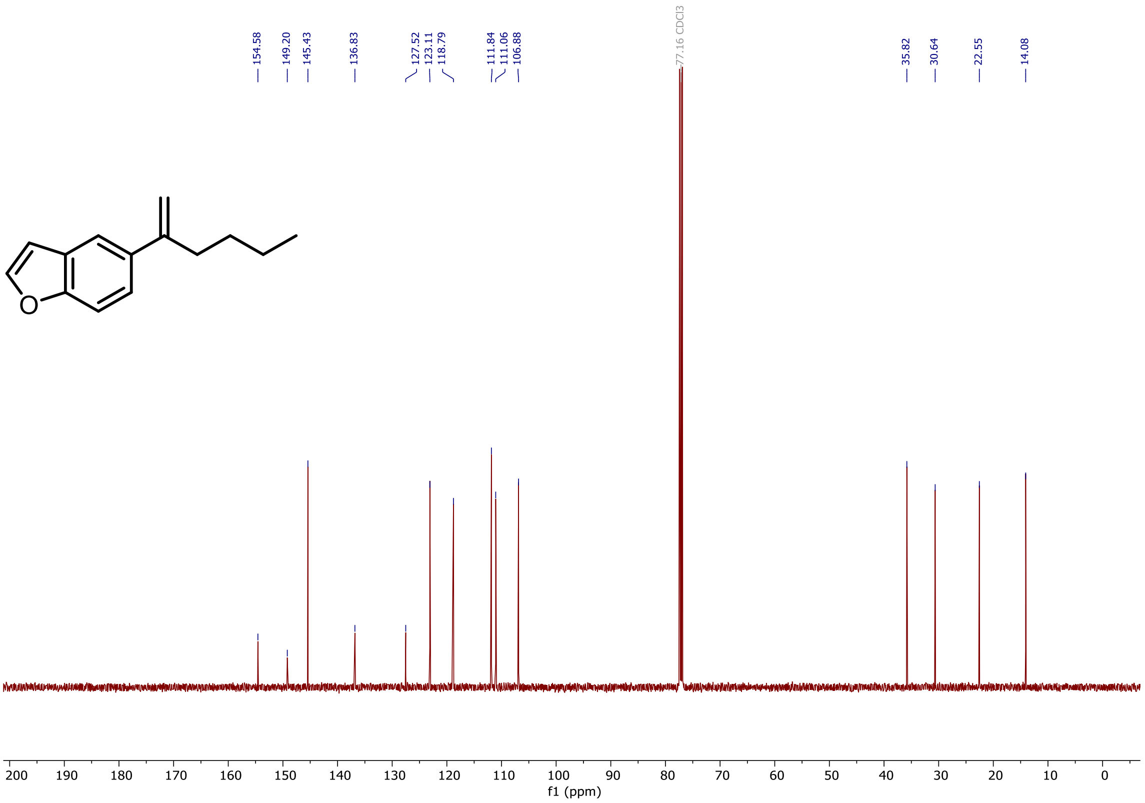

^{1}H NMR spectrum of **1l**

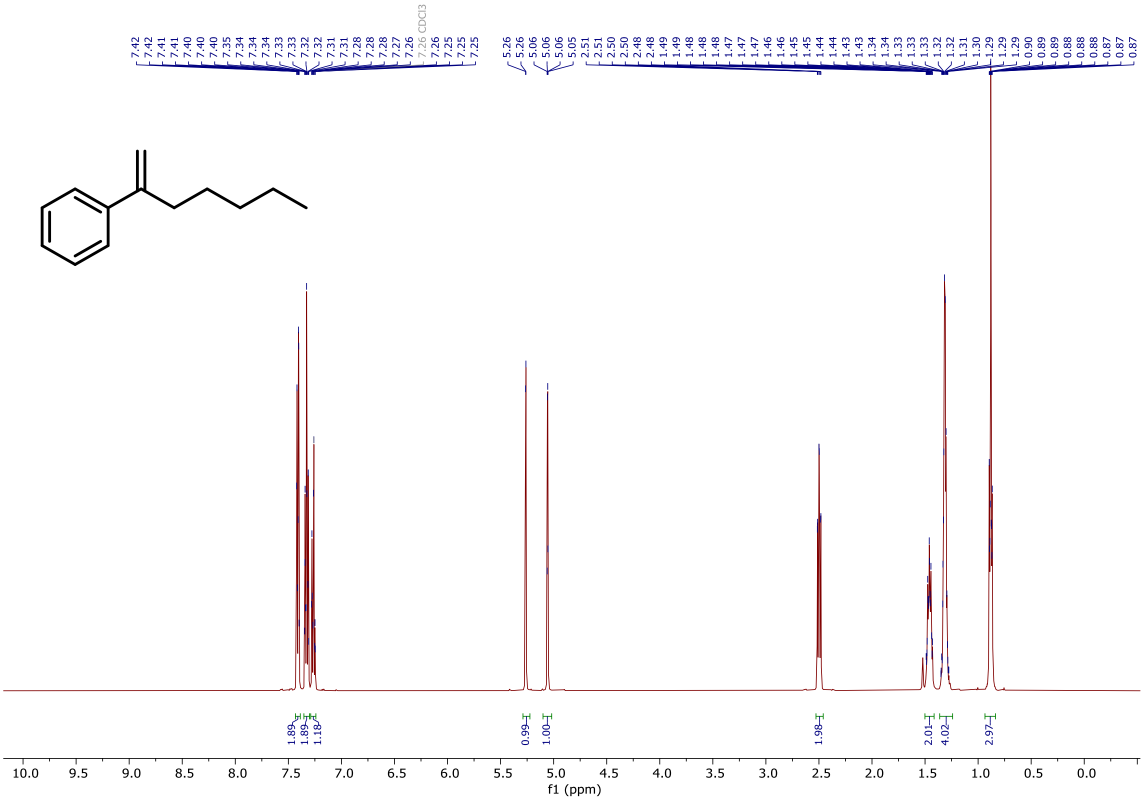

^{13}C NMR spectrum of **1l**

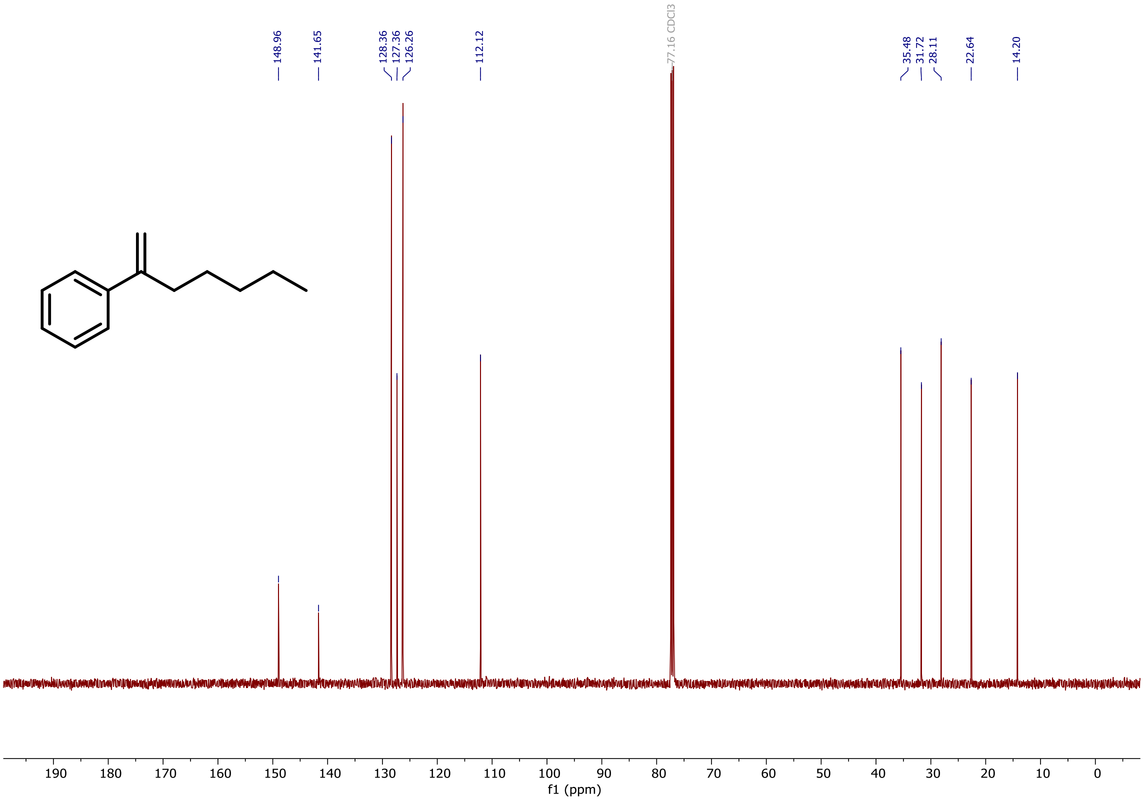

^{1}H NMR spectrum of **1m**

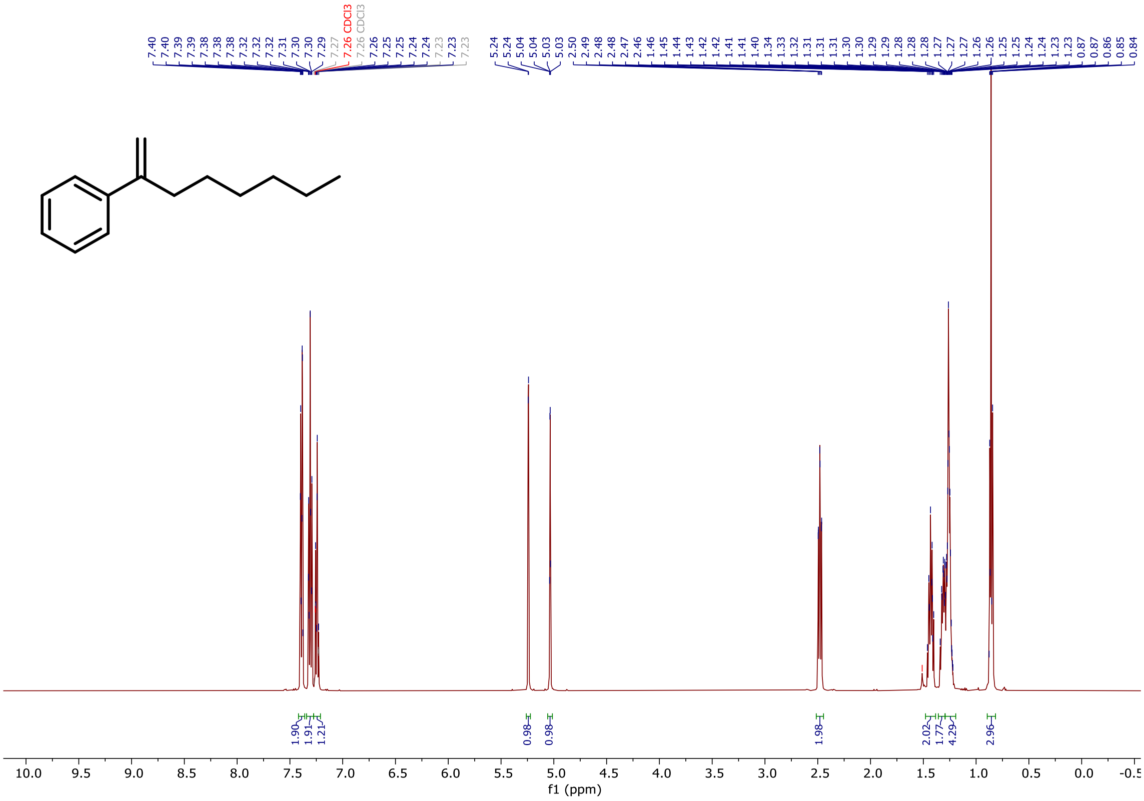

^{13}C NMR spectrum of **1m**

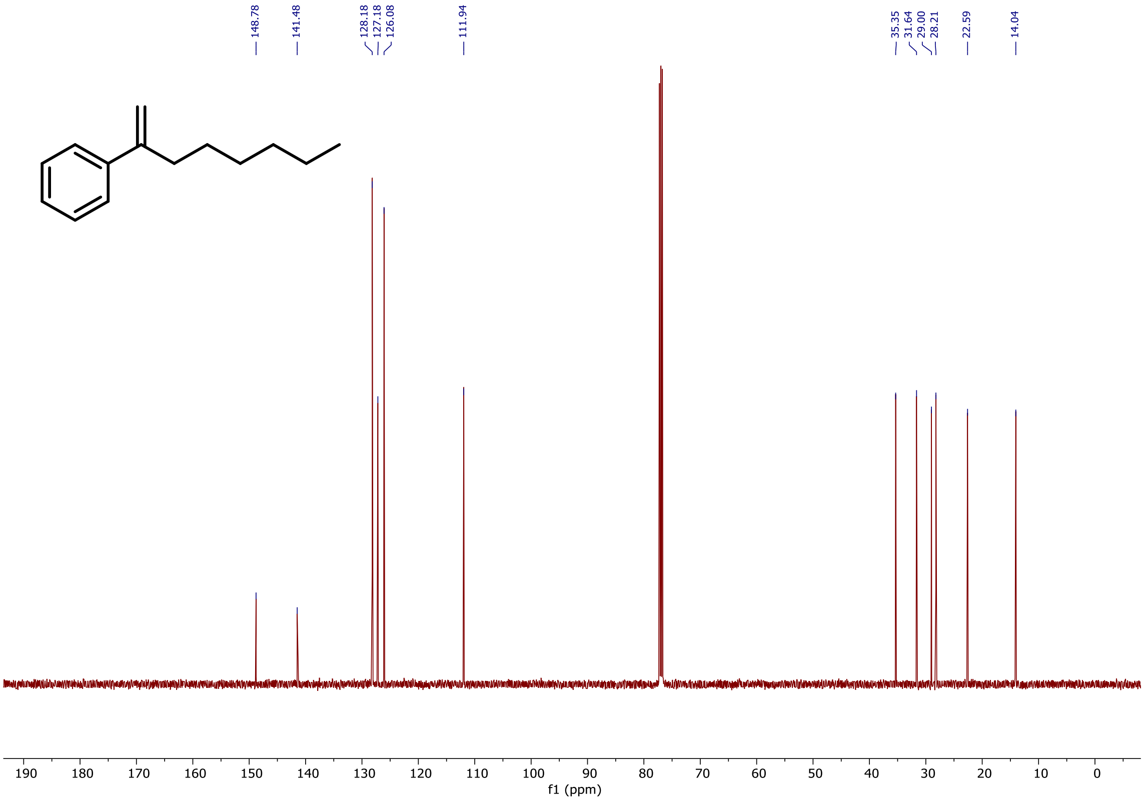

^{1}H NMR spectrum of **1n**

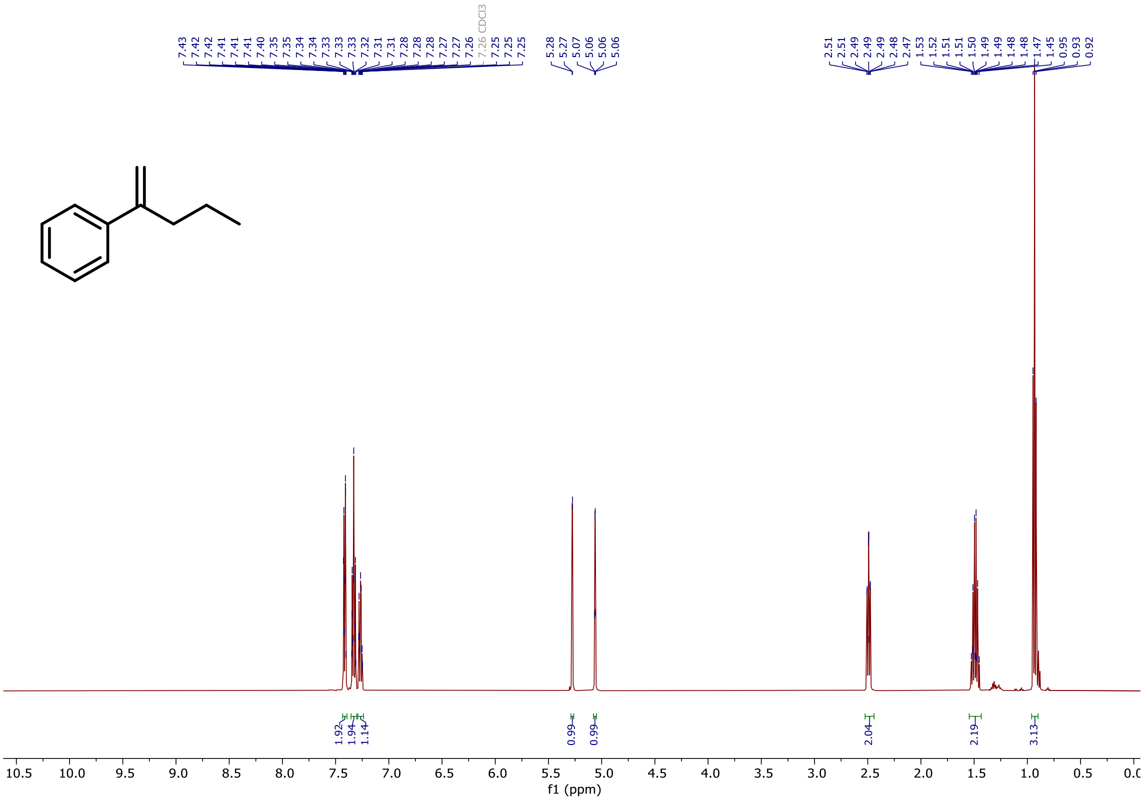

^{13}C NMR spectrum of **1n**

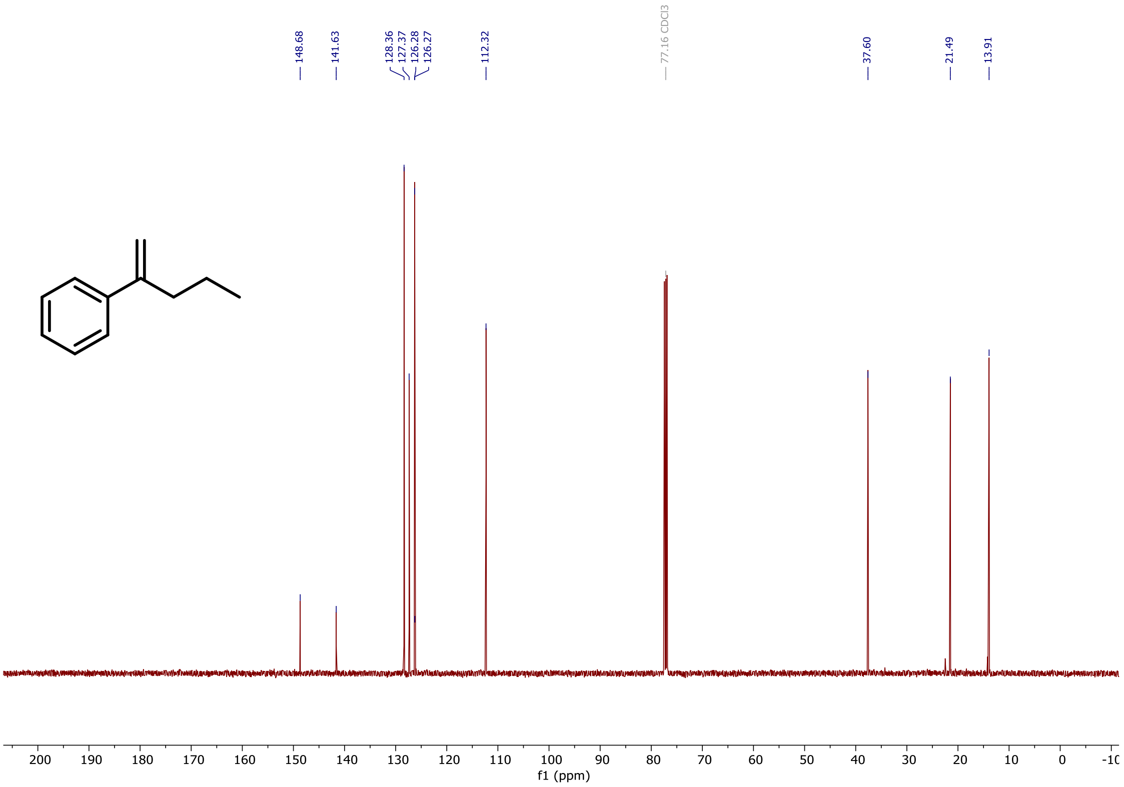

^{1}H NMR spectrum of **1o**

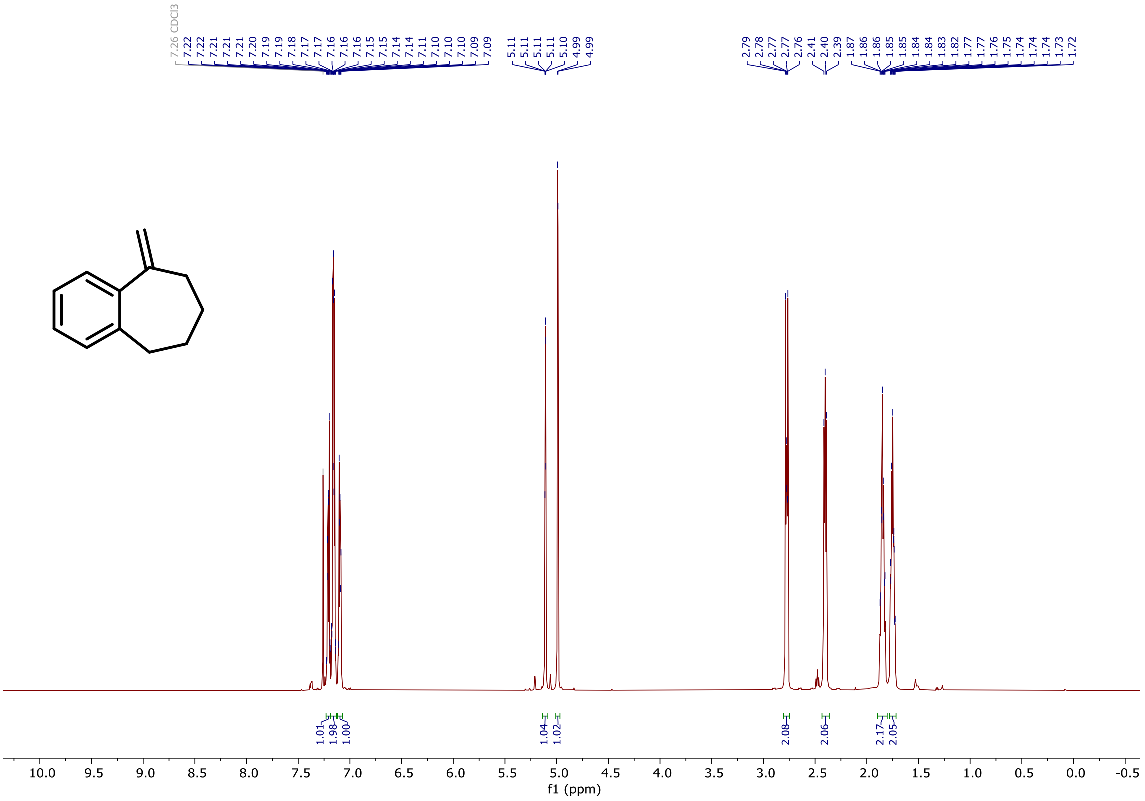

^{13}C NMR spectrum of **1o**

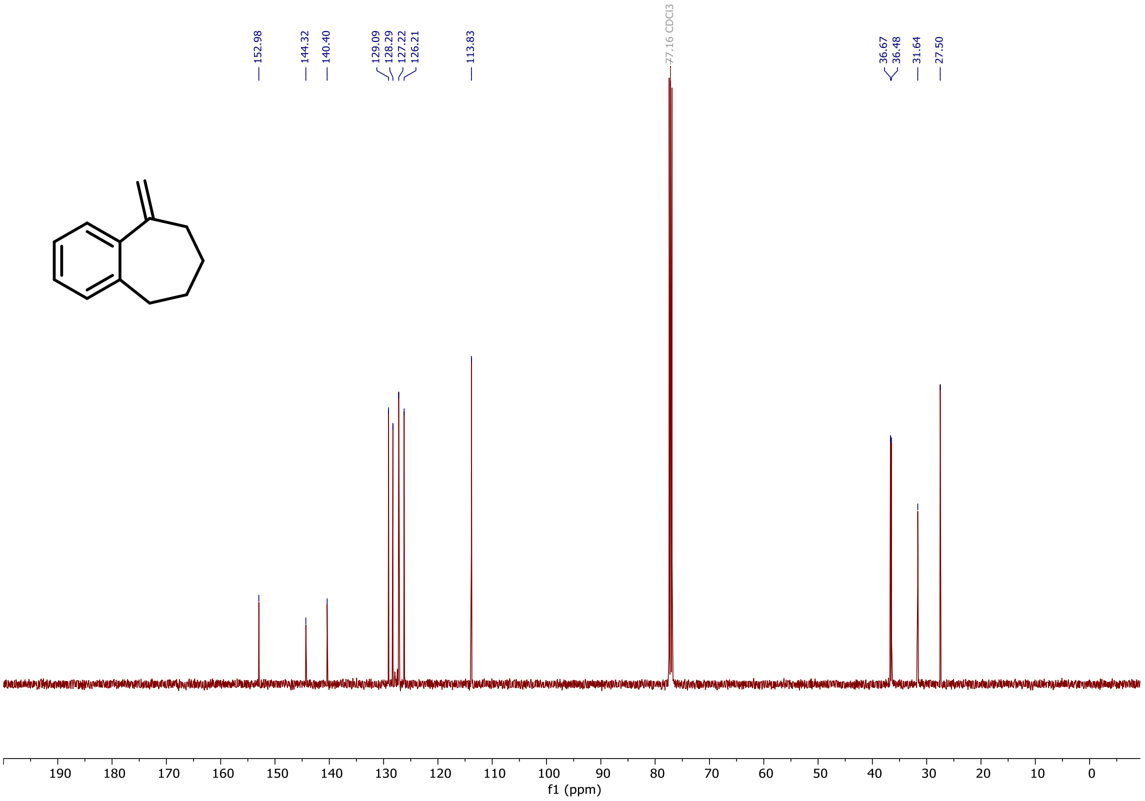

^{1}H NMR spectrum of **S5**

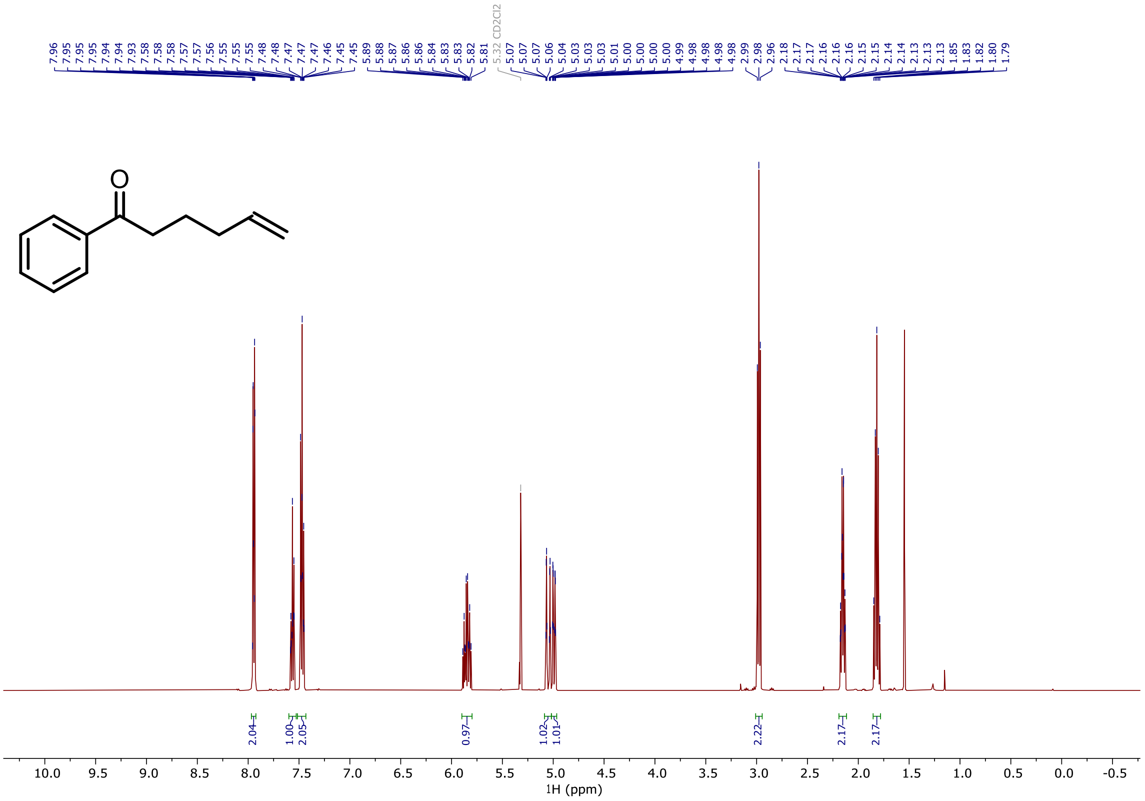

^{13}C NMR spectrum of **S5**

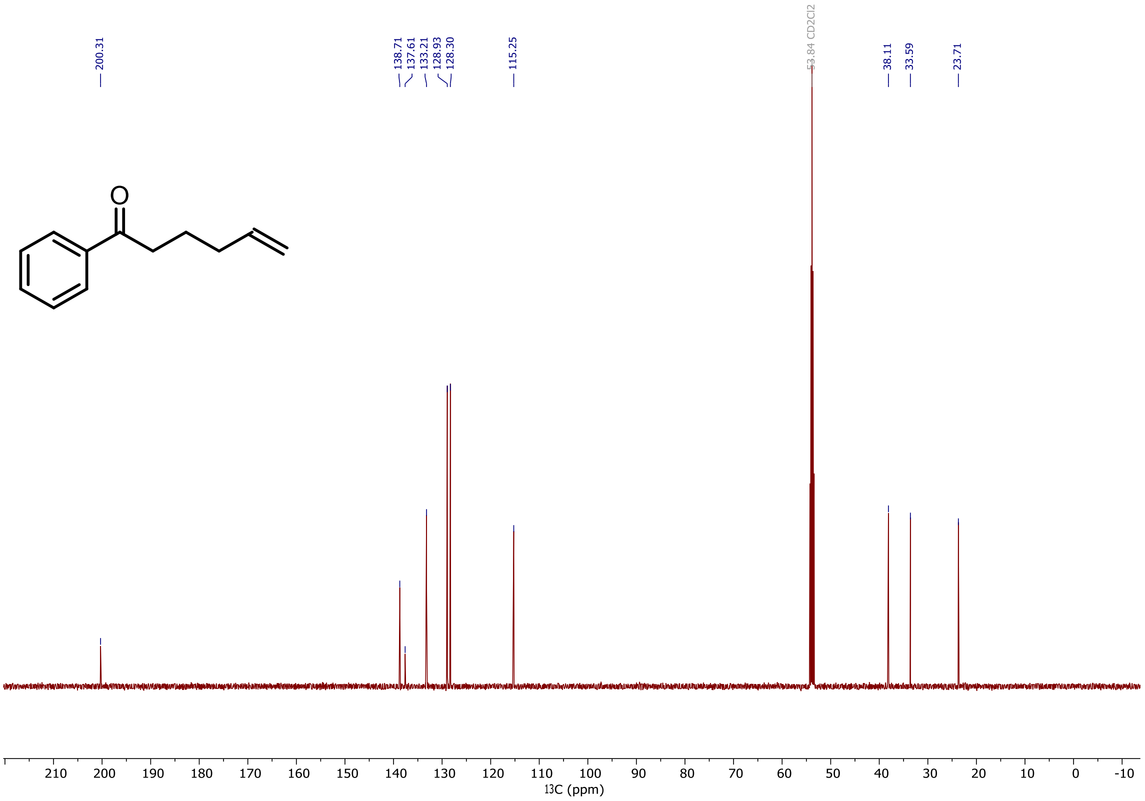

^{1}H NMR spectrum of **1p**

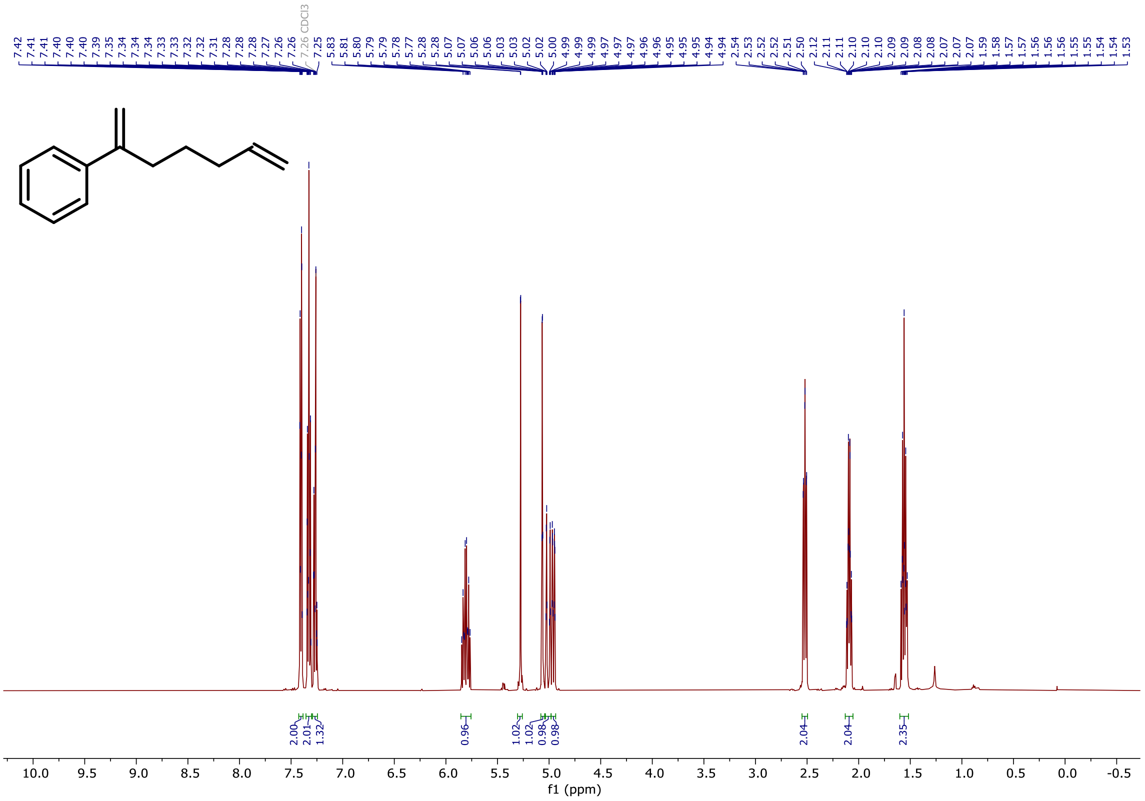

^{13}C NMR spectrum of **1p**

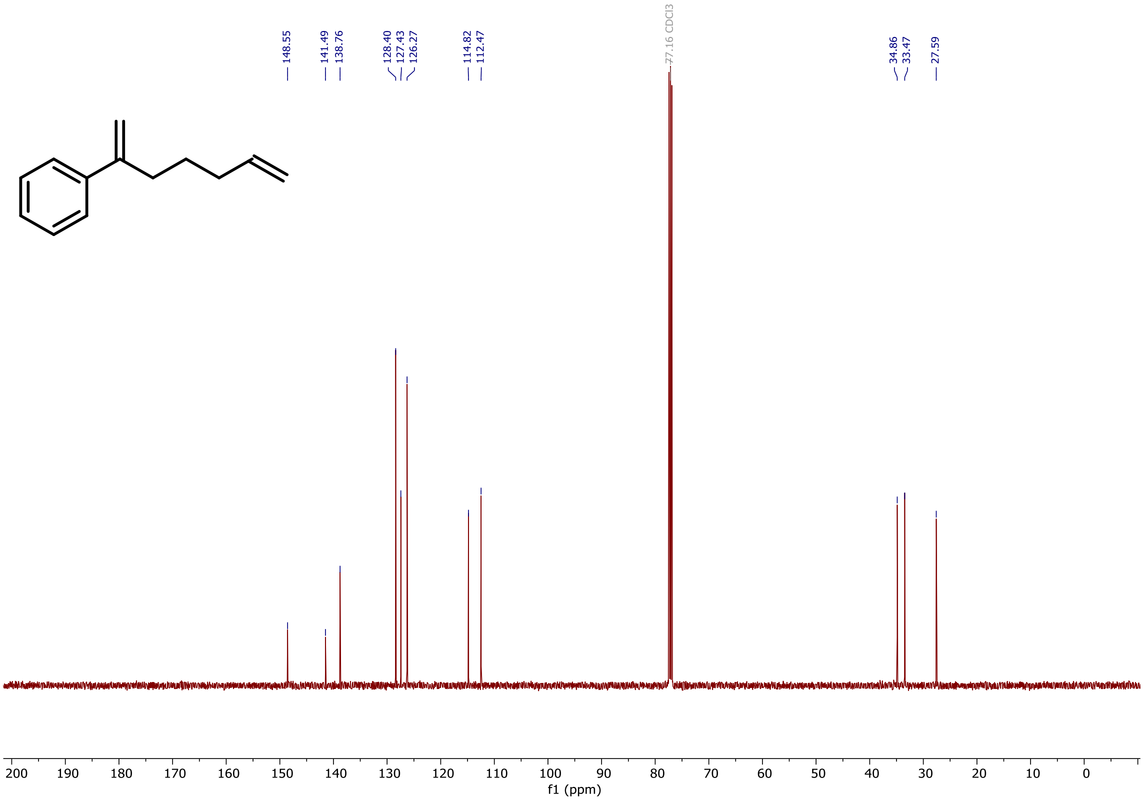

^{1}H NMR spectrum of **1q**

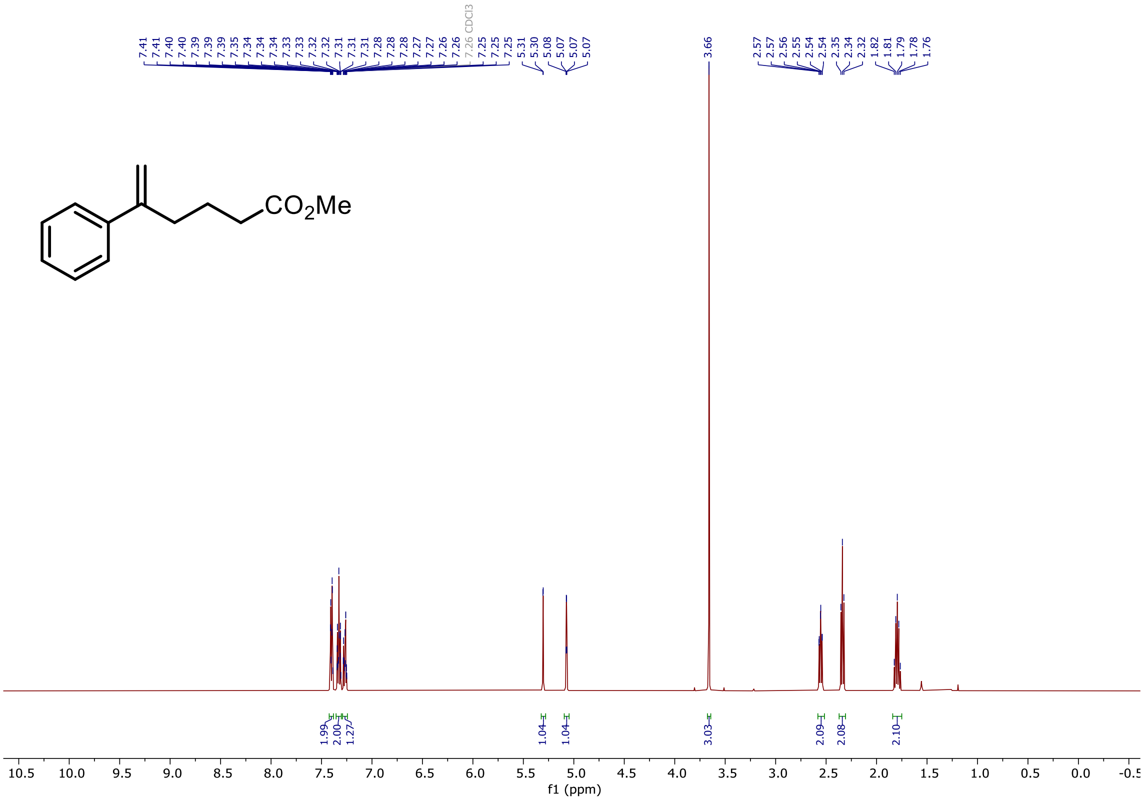

^{13}C NMR spectrum of **1q**

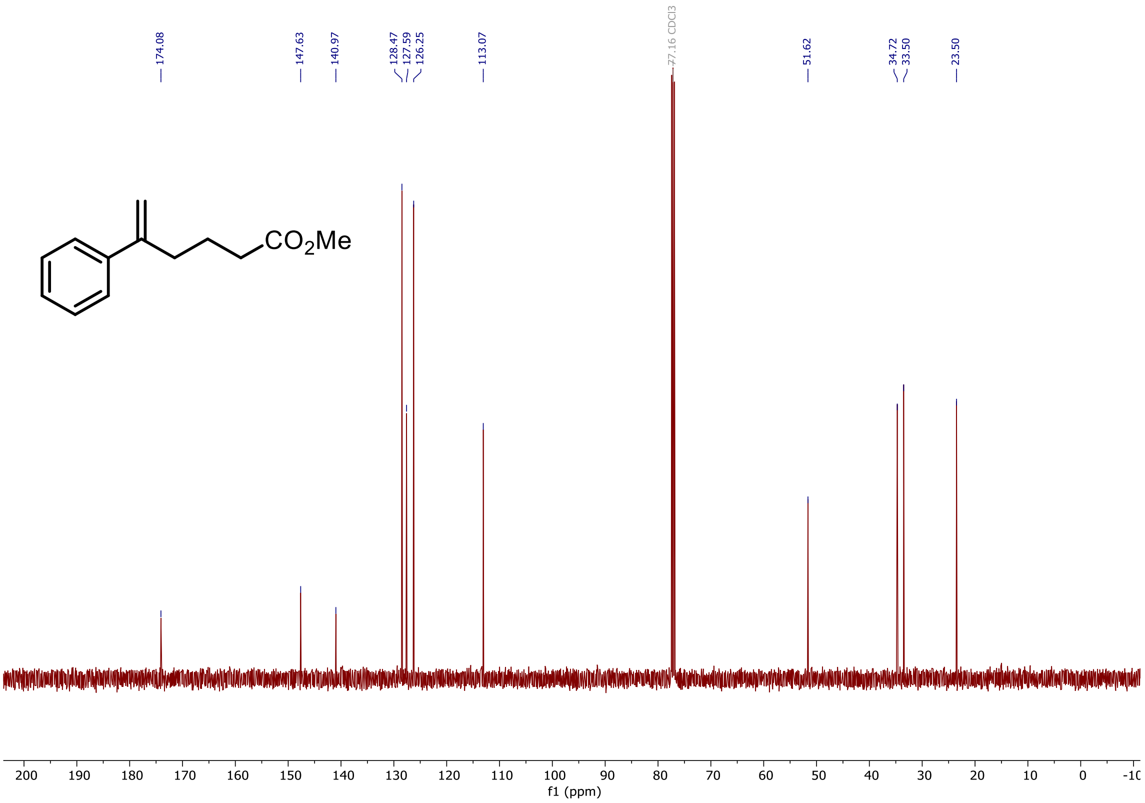

^{1}H NMR spectrum of **S6**

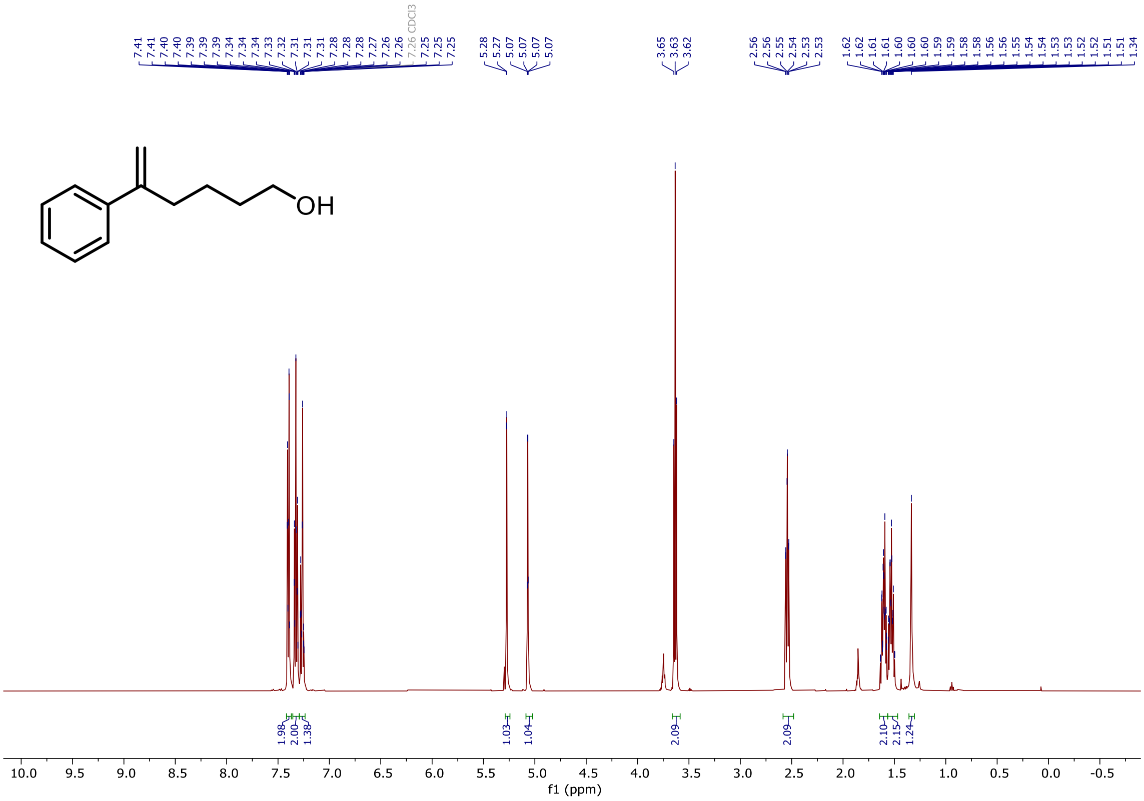

^{13}C NMR spectrum of **S6**

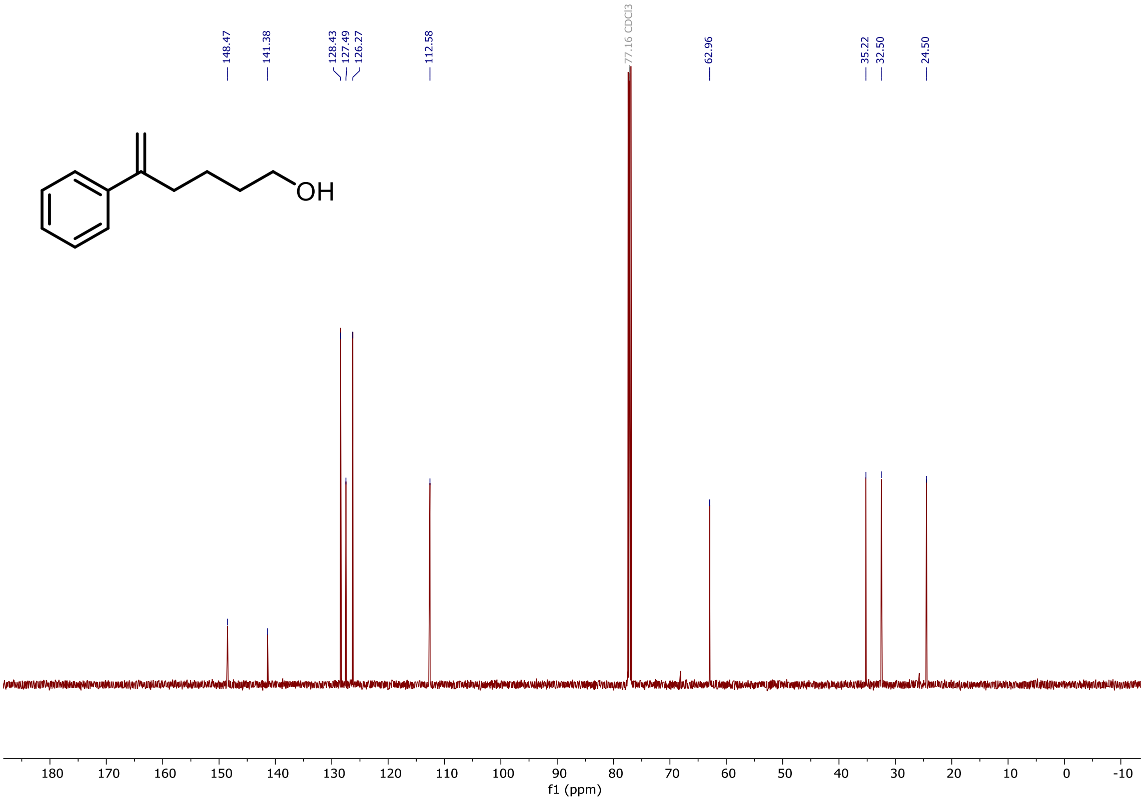

^{1}H NMR spectrum of **1r**

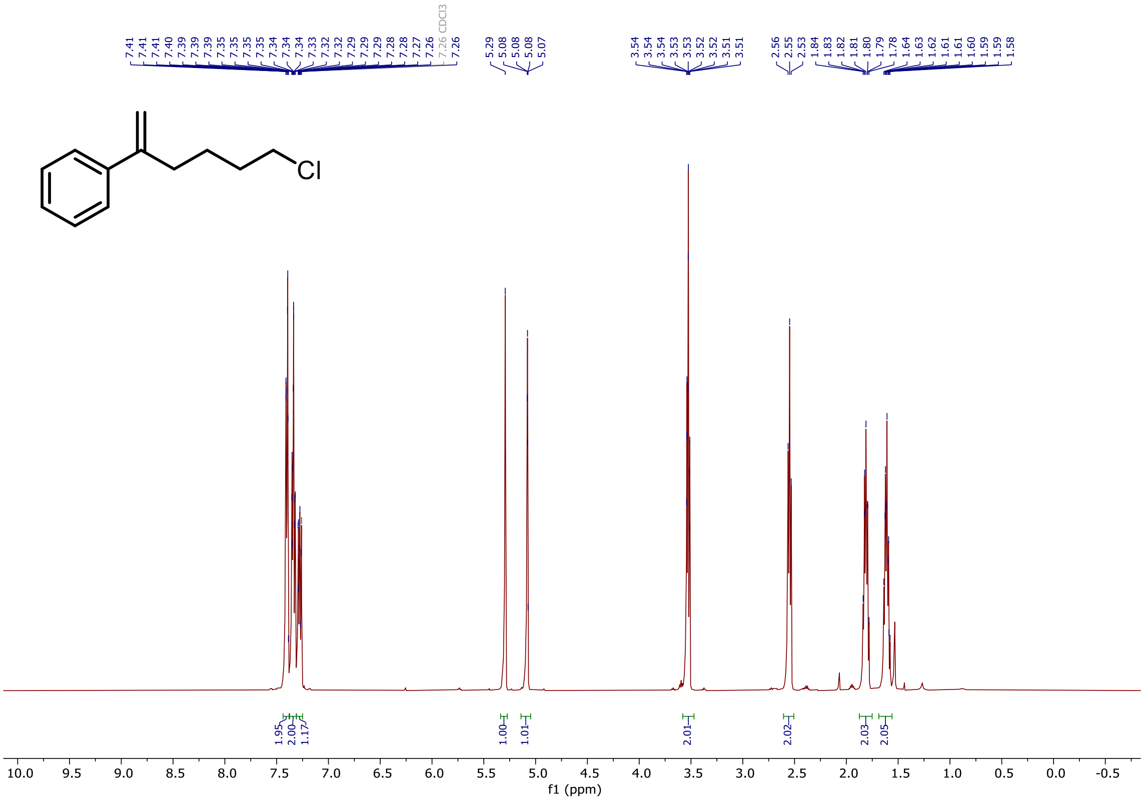

^{13}C NMR spectrum of **1r**

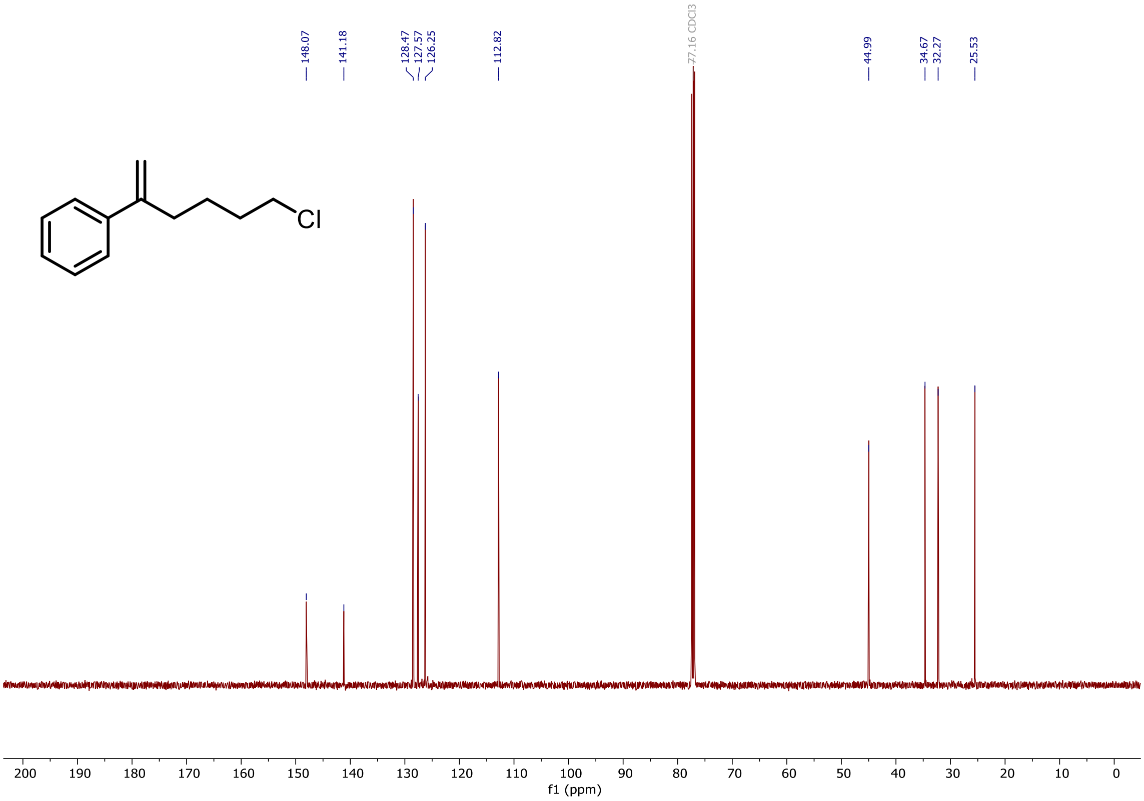

^{1}H NMR spectrum of (*E*)-**3j**

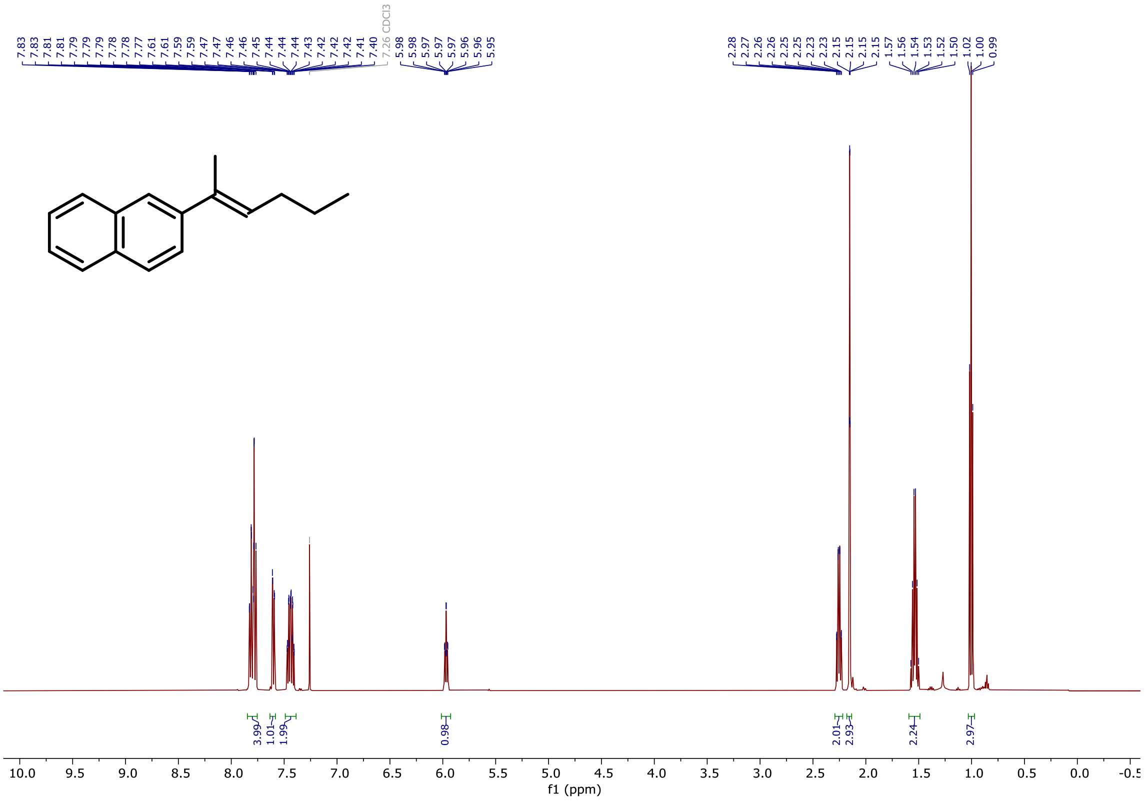

^{13}C NMR spectrum of (*E*)-**3j**

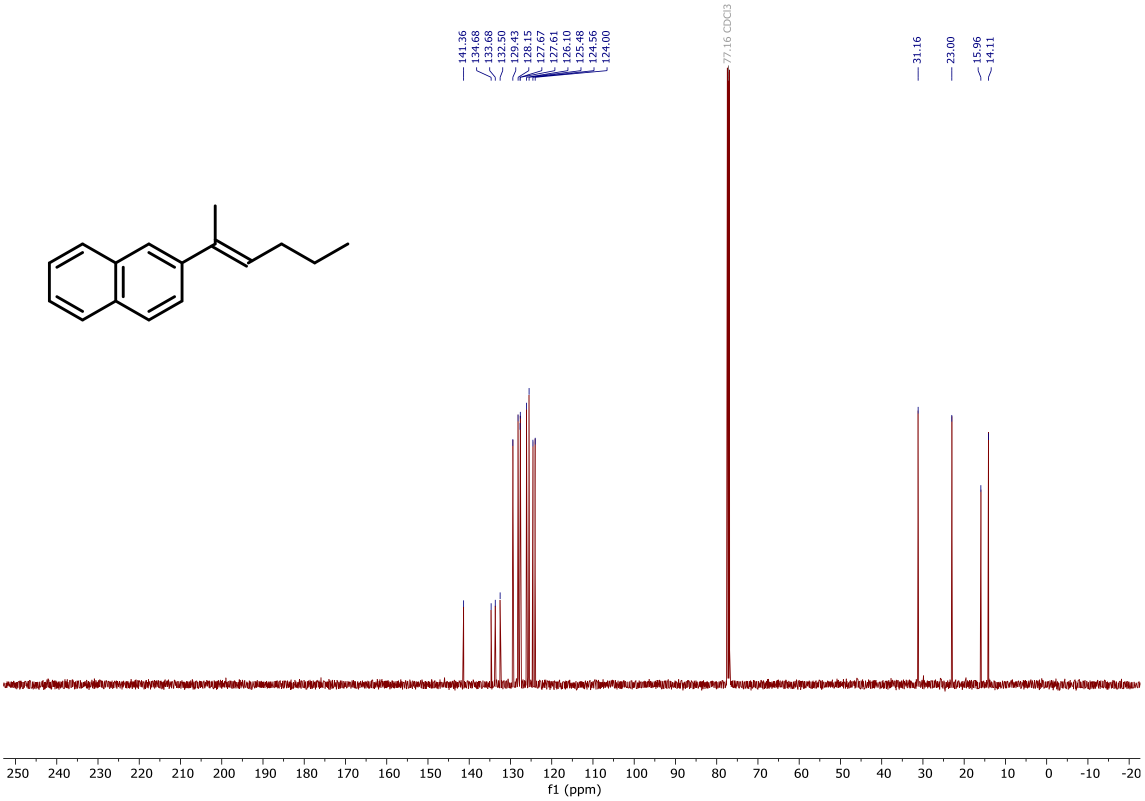

^{1}H NMR spectrum of (*Z*)-**3j**

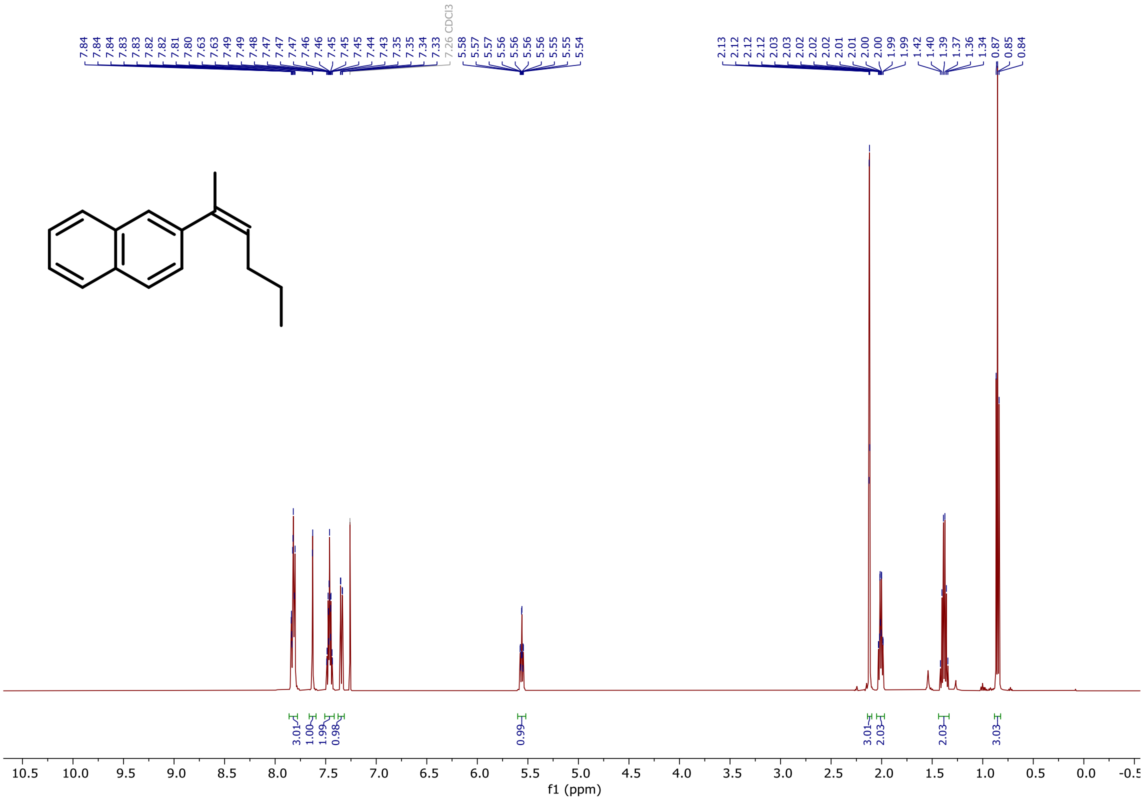

^{13}C NMR spectrum of (*Z*)-**3j**

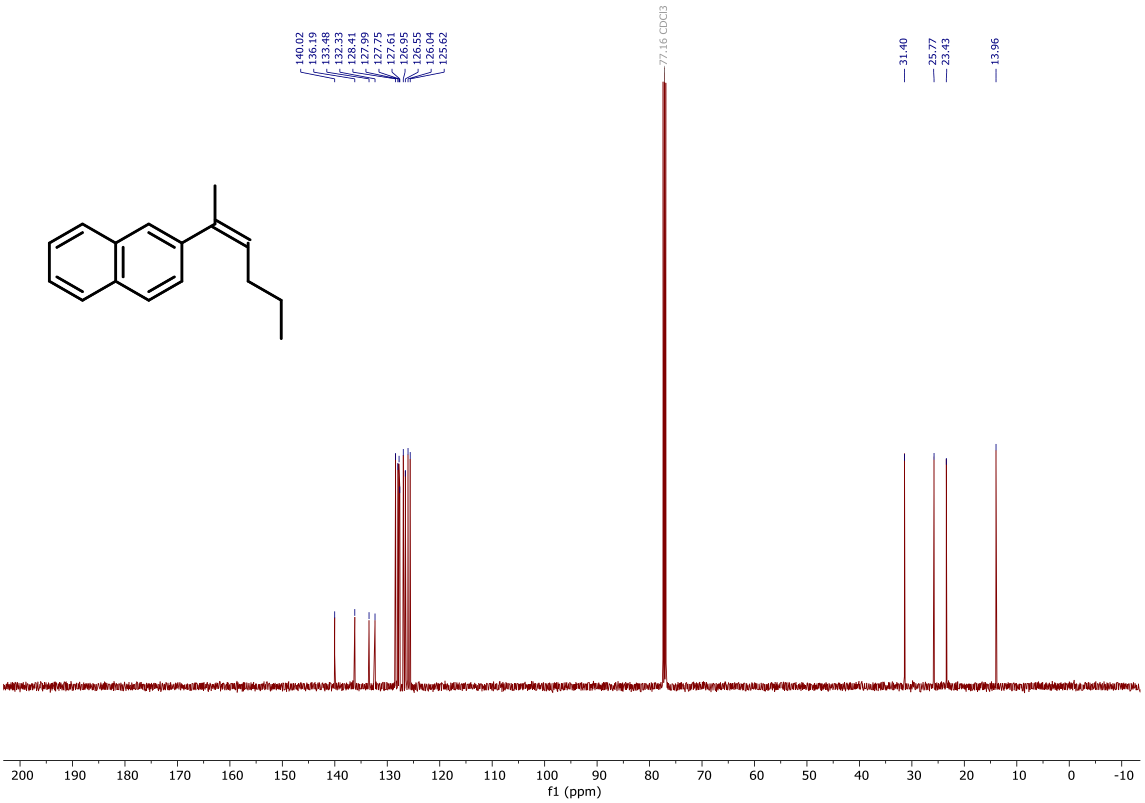

^{1}H NMR spectrum of **2a**

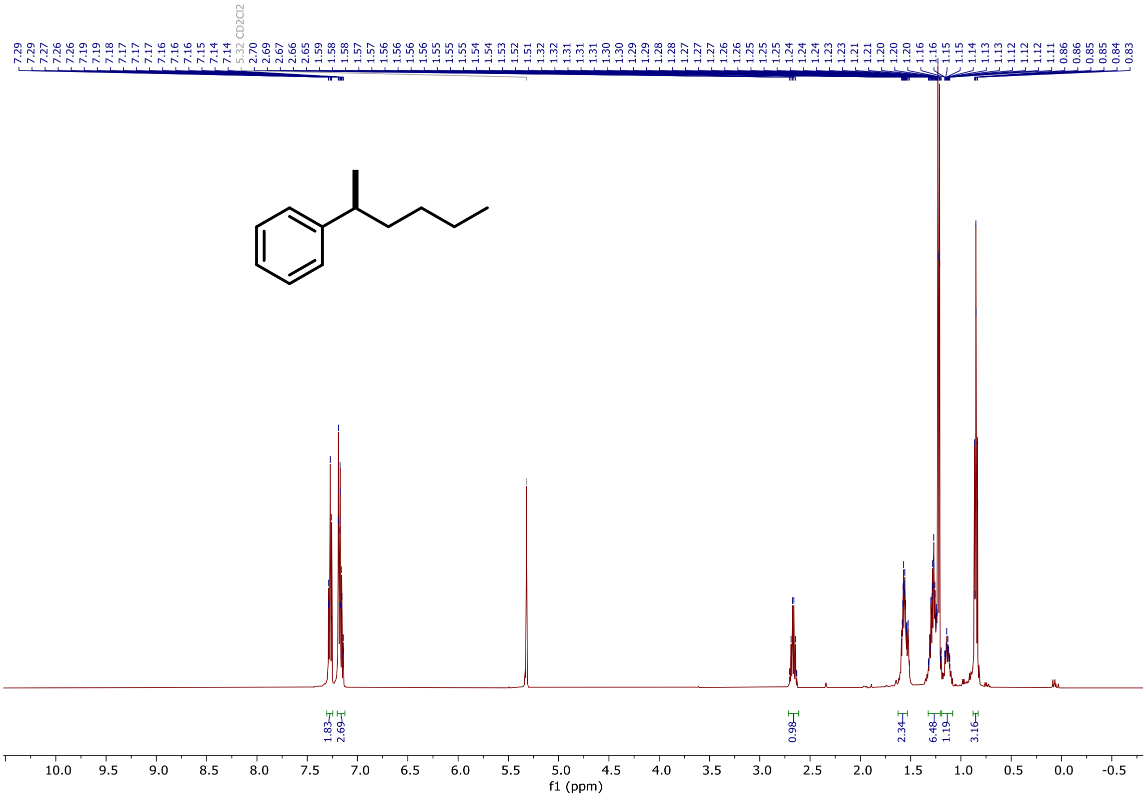

^{13}C NMR spectrum of **2a**

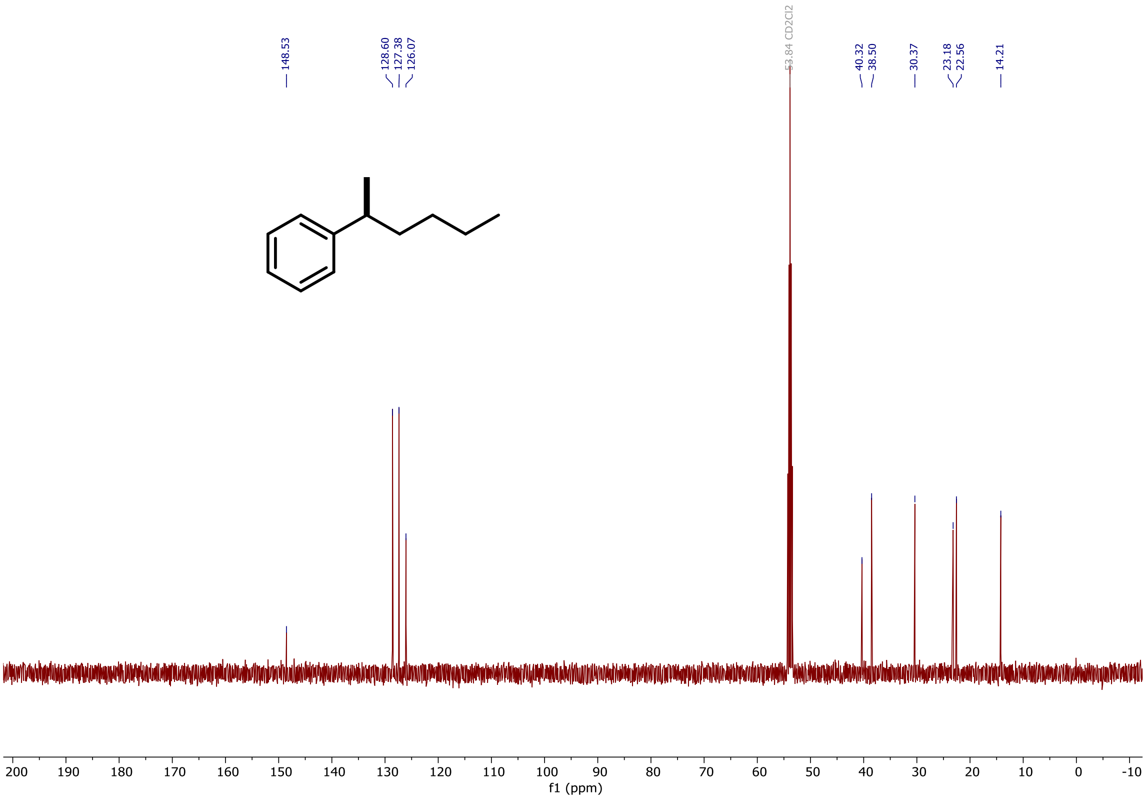

^{1}H NMR spectrum of (*E*)-**3a**

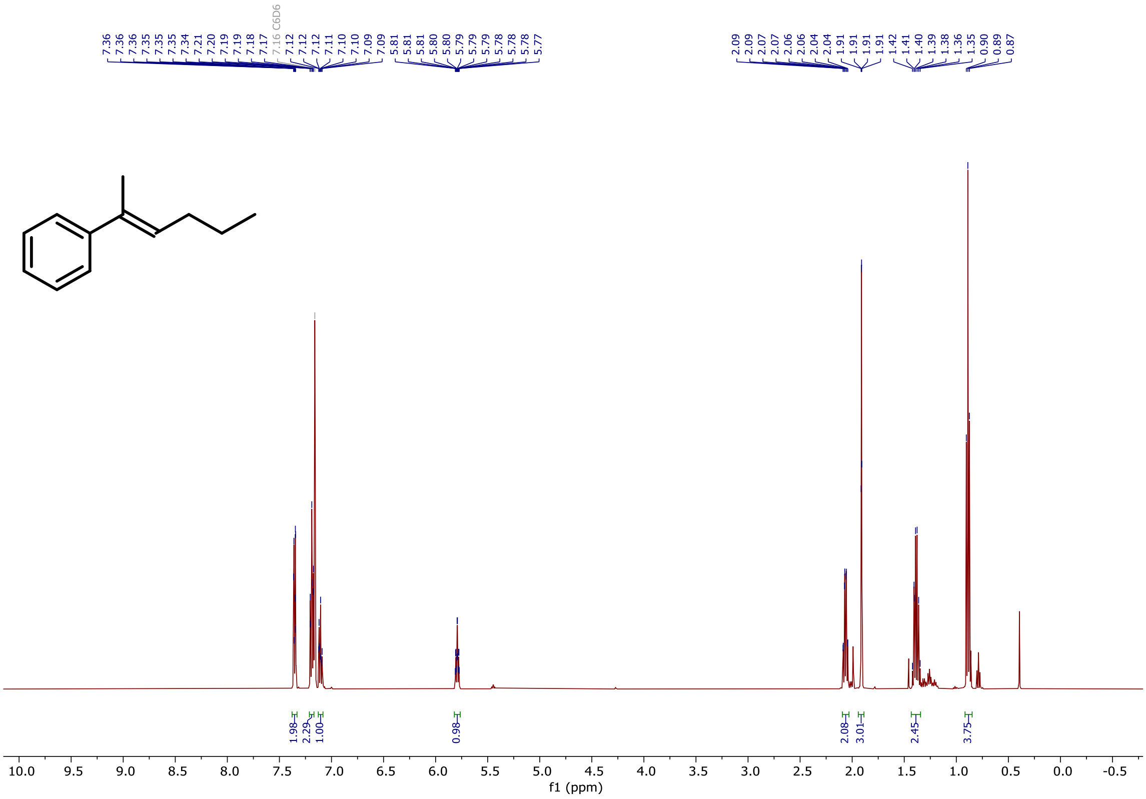

^{13}C NMR spectrum of (*E*)-**3a**

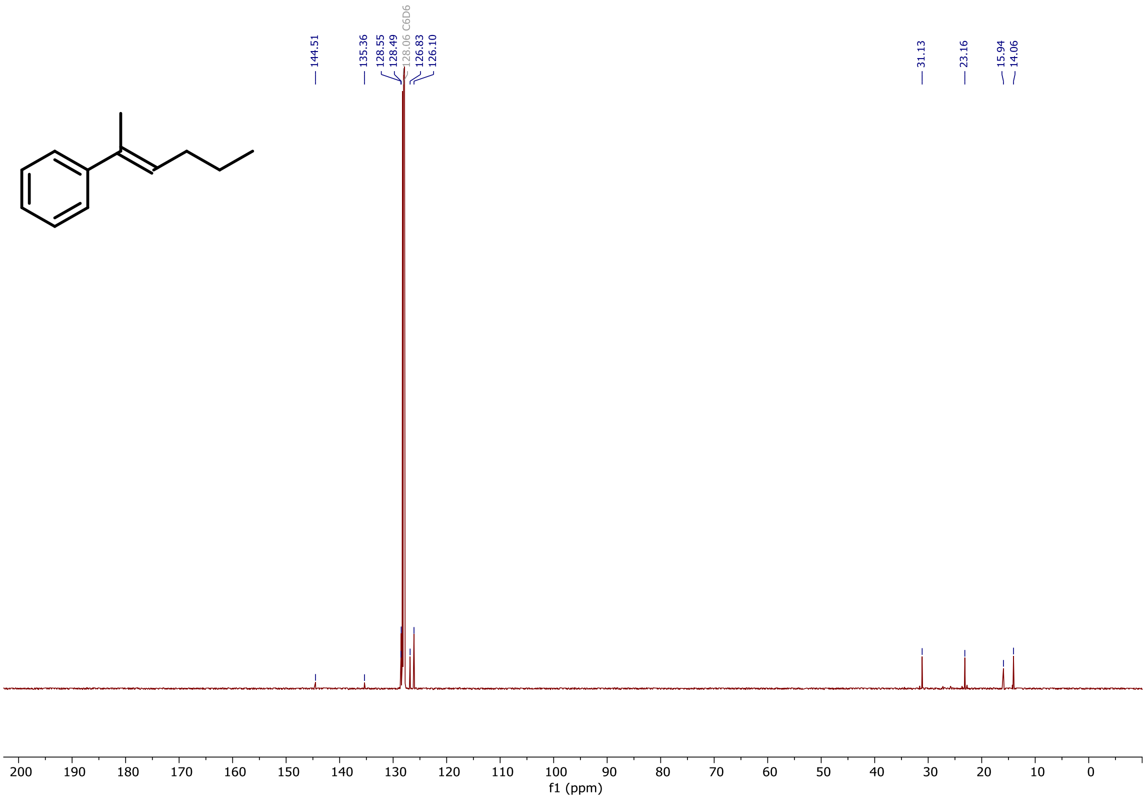

^{1}H NMR spectrum of (*Z*)-**3a**

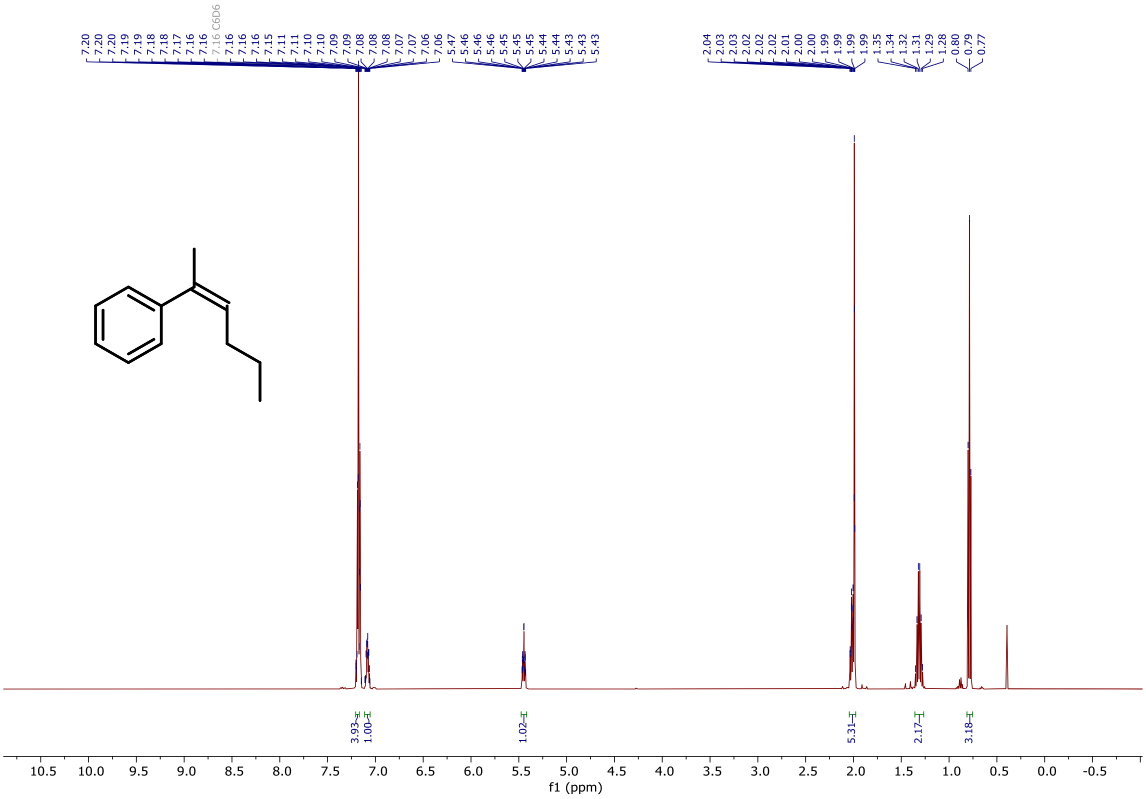

^{13}C NMR spectrum of (*Z*)-**3a**

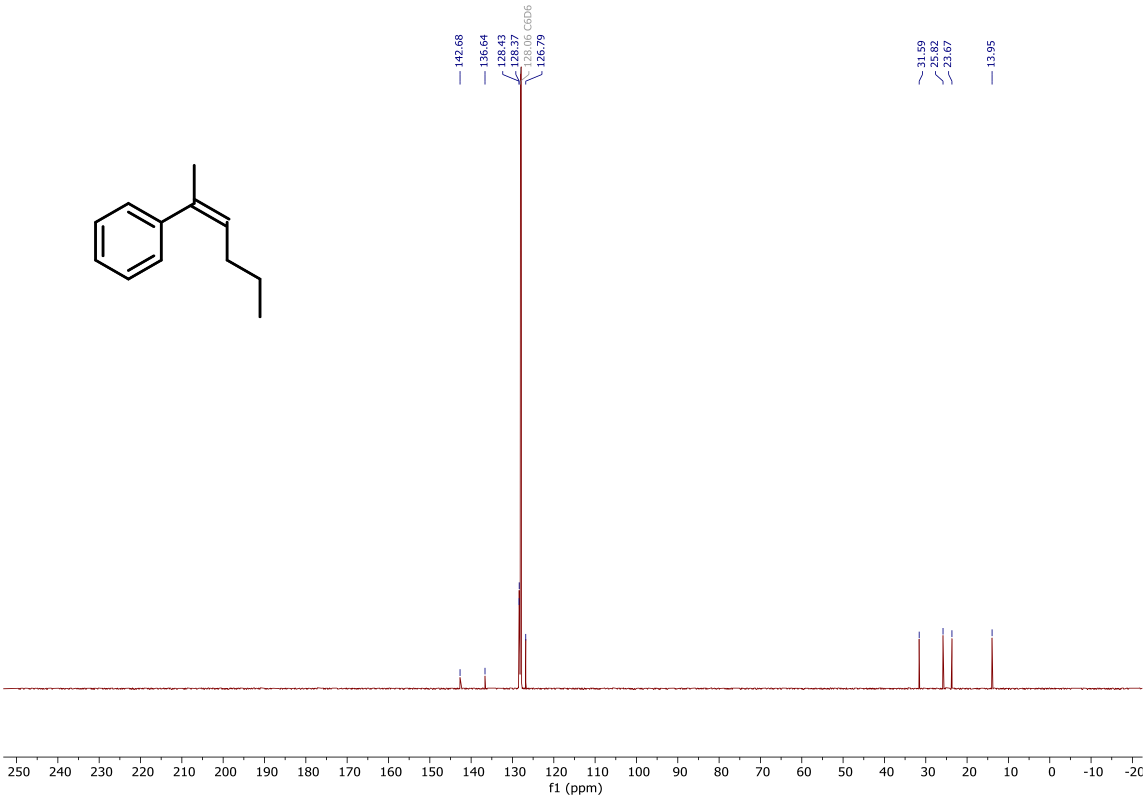

^{1}H NMR spectrum of **4a**

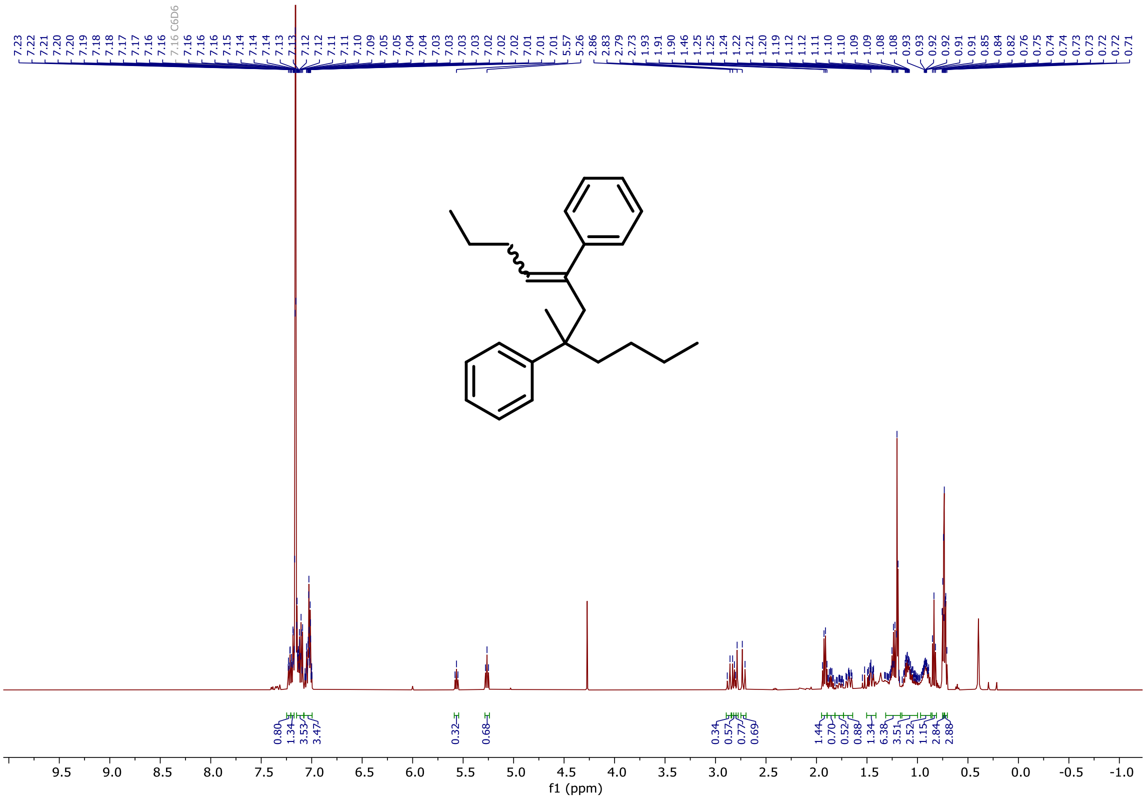

^{1}H NMR spectrum of **2b**

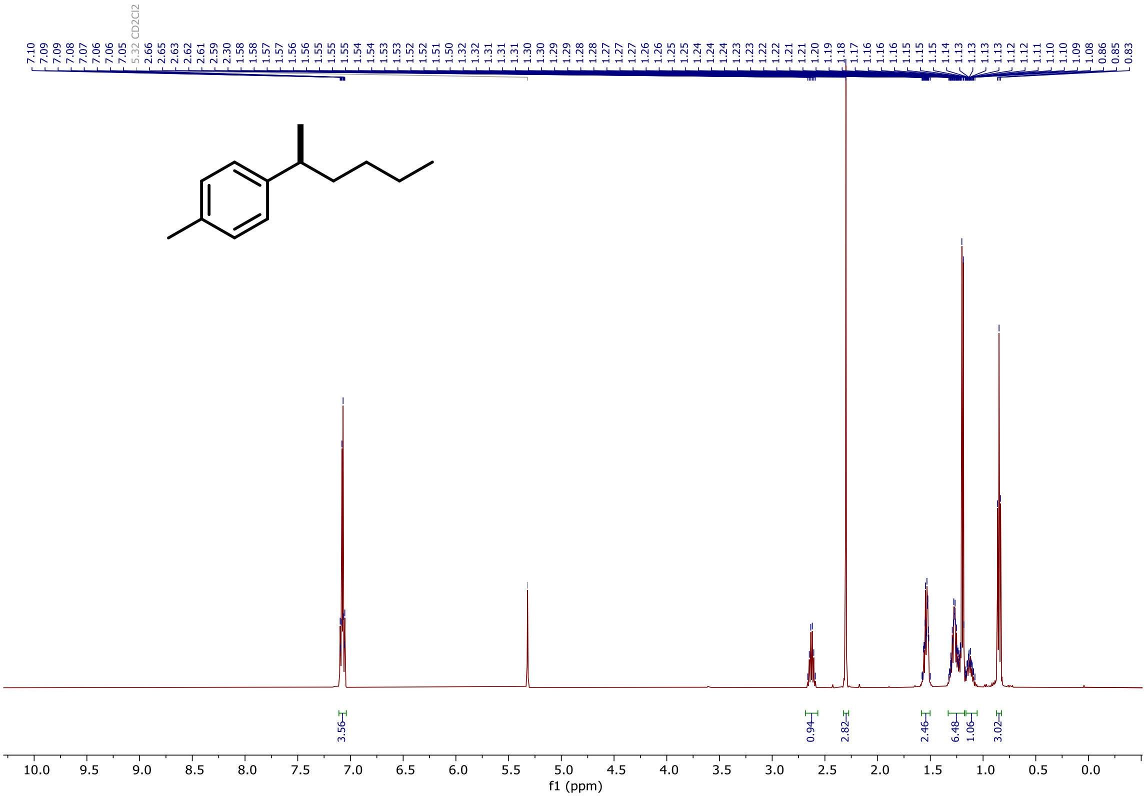

^{13}C NMR spectrum of **2b**

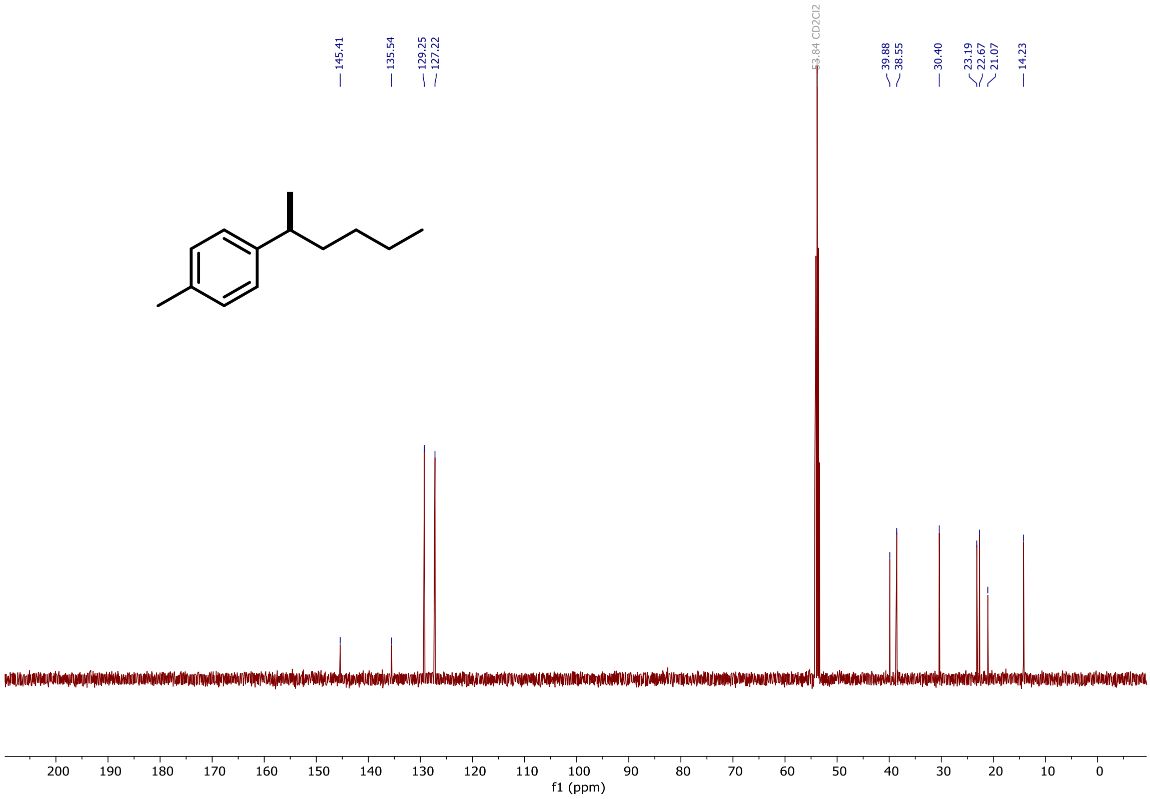

^{1}H NMR spectrum of **2c**

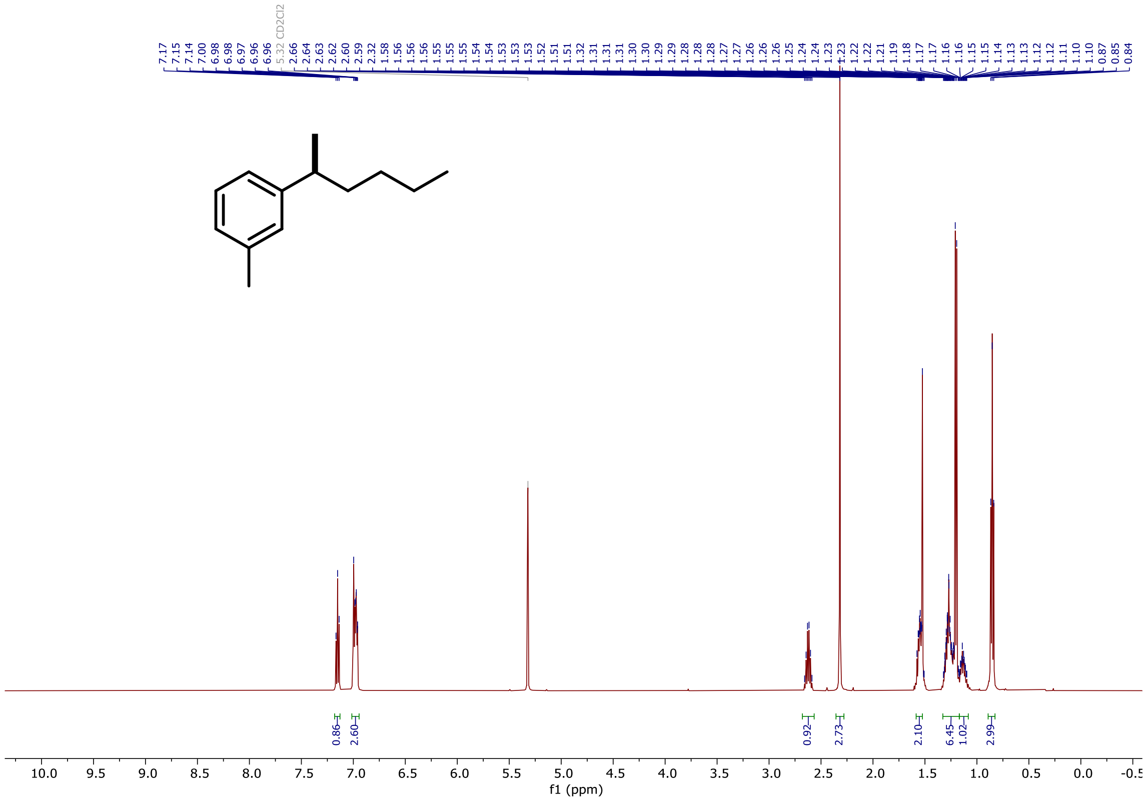

^{13}C NMR spectrum of **2c**

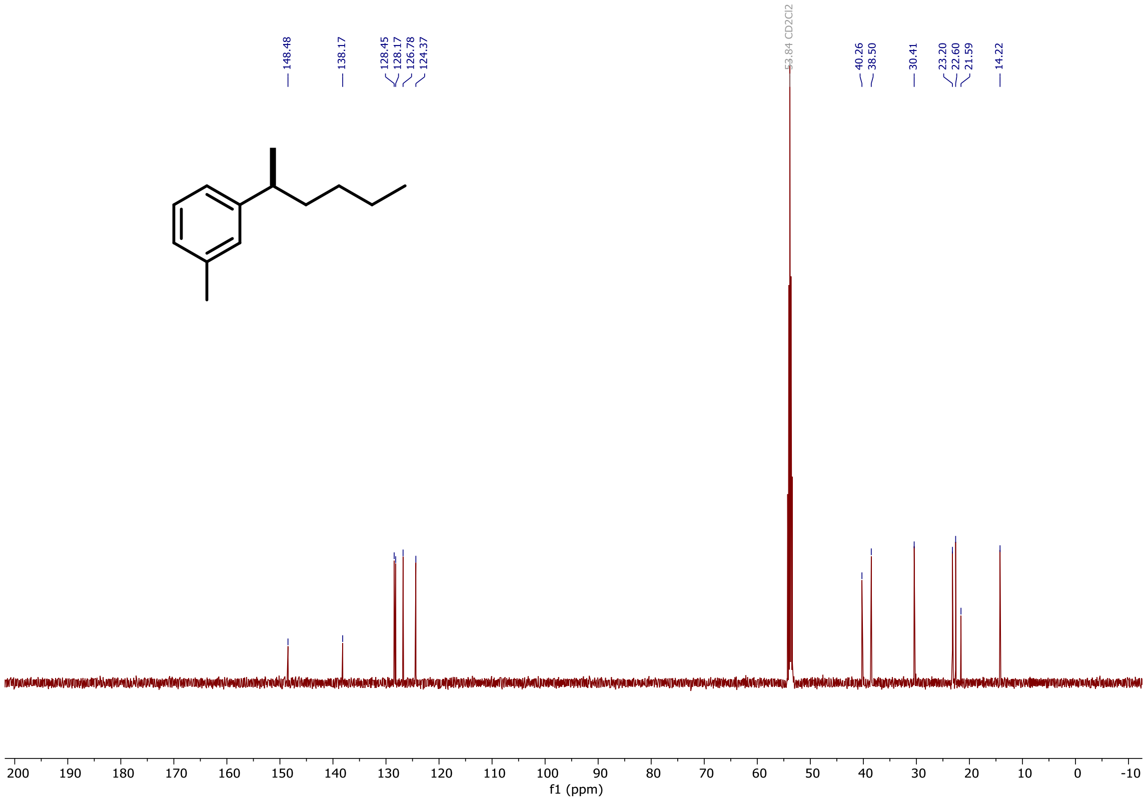

^{1}H NMR spectrum of **2d**

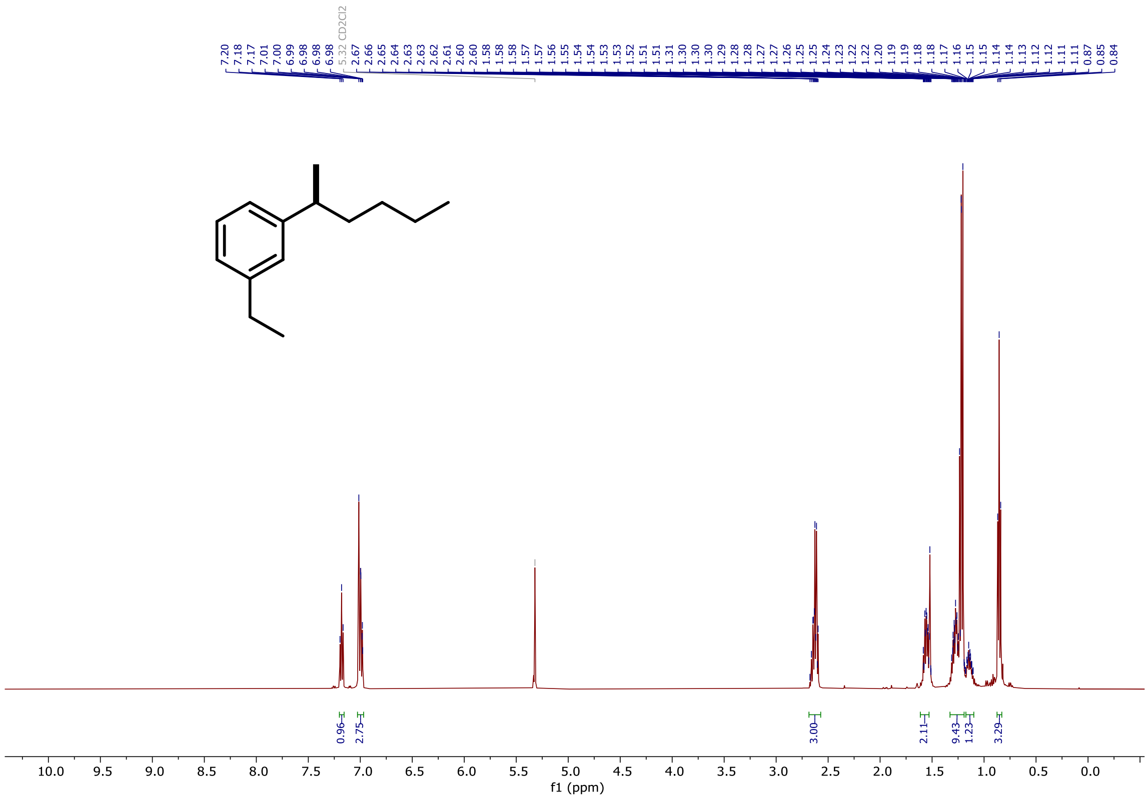

^{13}C NMR spectrum of **2d**

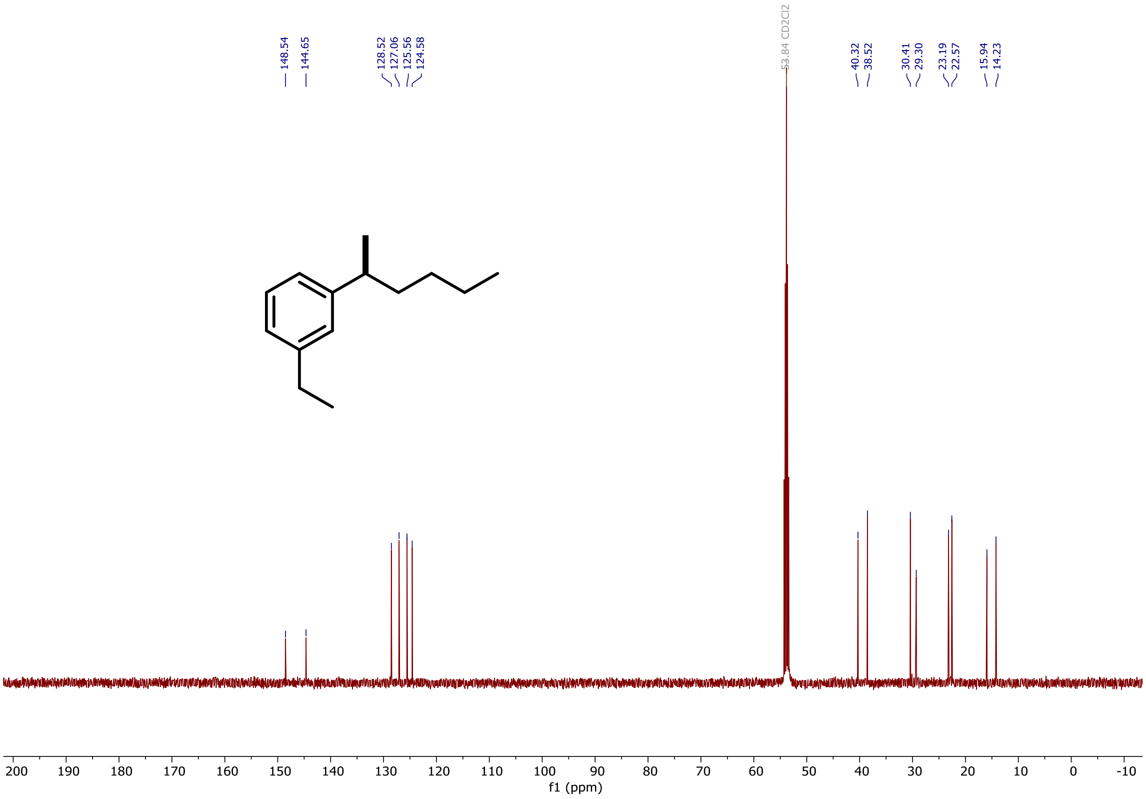

^{1}H NMR spectrum of **2e**

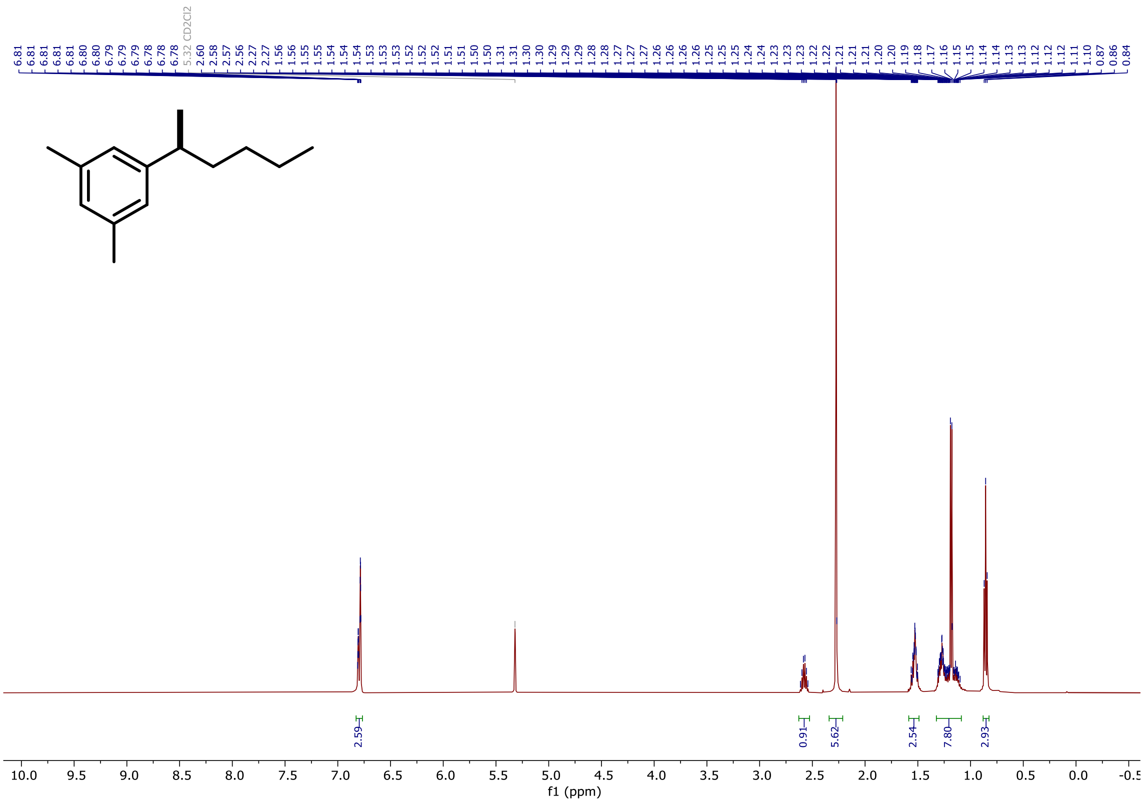

^{13}C NMR spectrum of **2e**

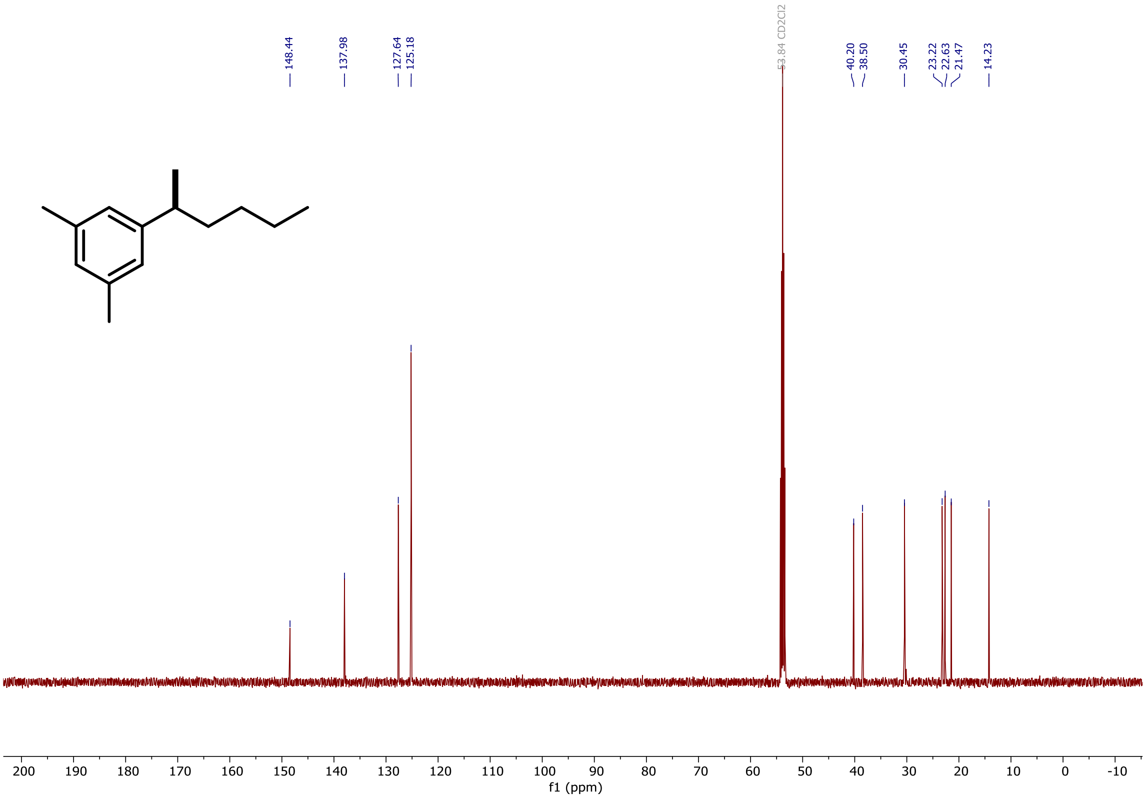

^{1}H NMR spectrum of **2f**

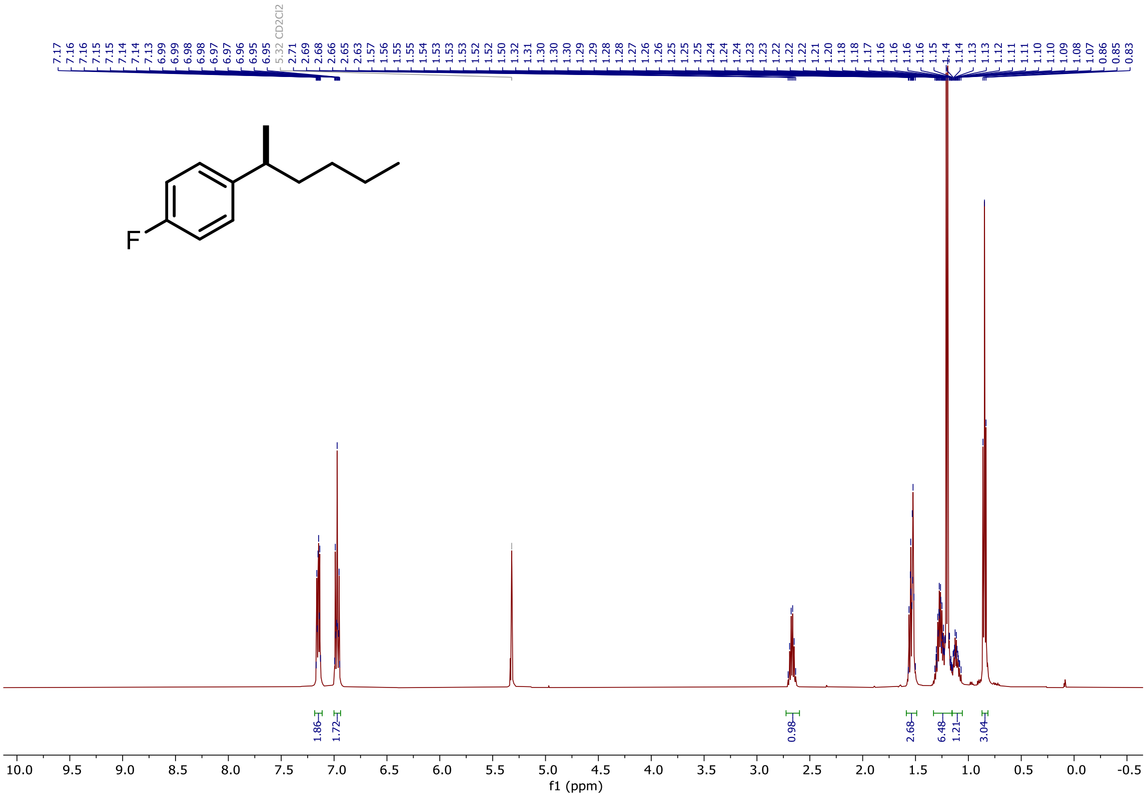

$^{19}F\{H\}$ NMR spectrum of **2f**

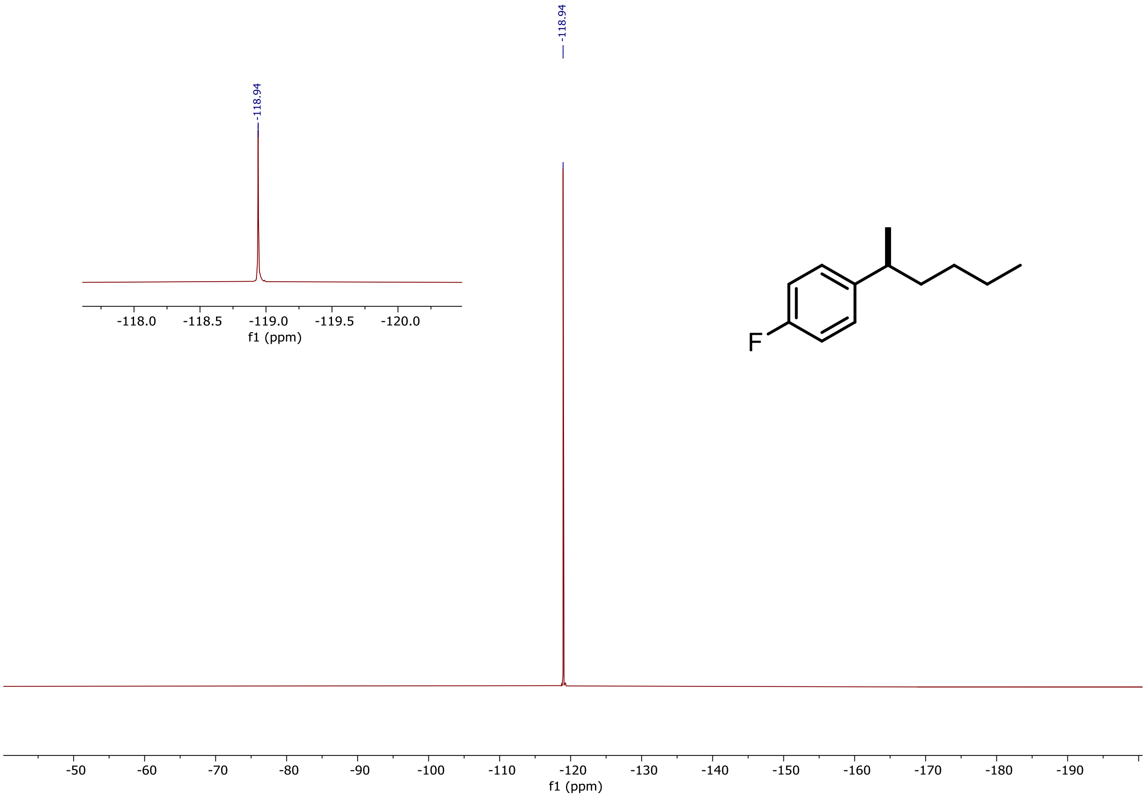

^{13}C NMR spectrum of **2f**

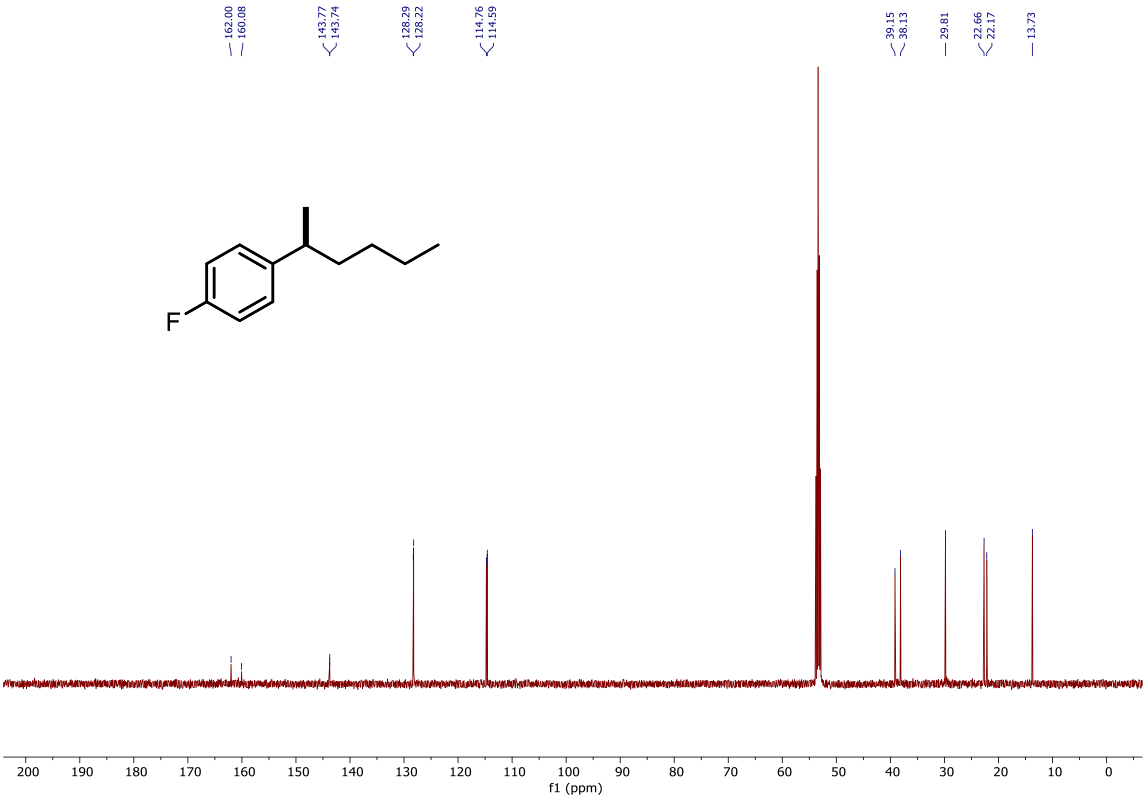

^{1}H NMR spectrum of **2g**

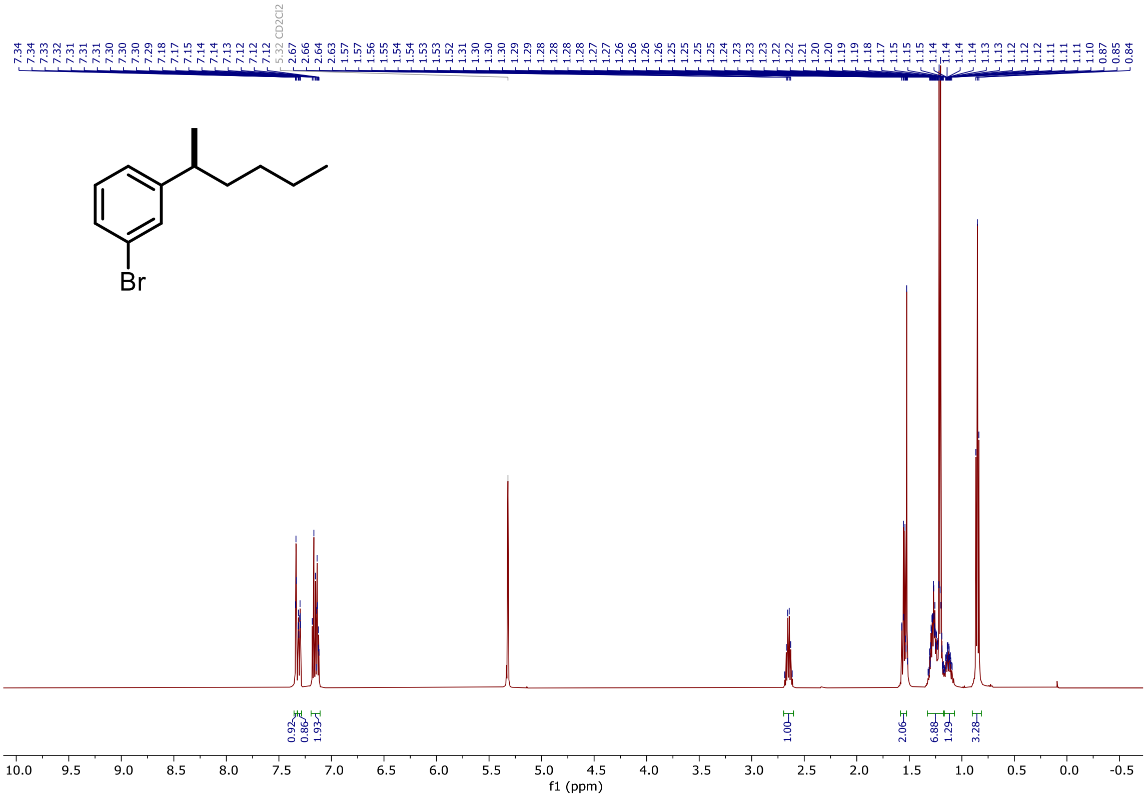

^{13}C NMR spectrum of **2g**

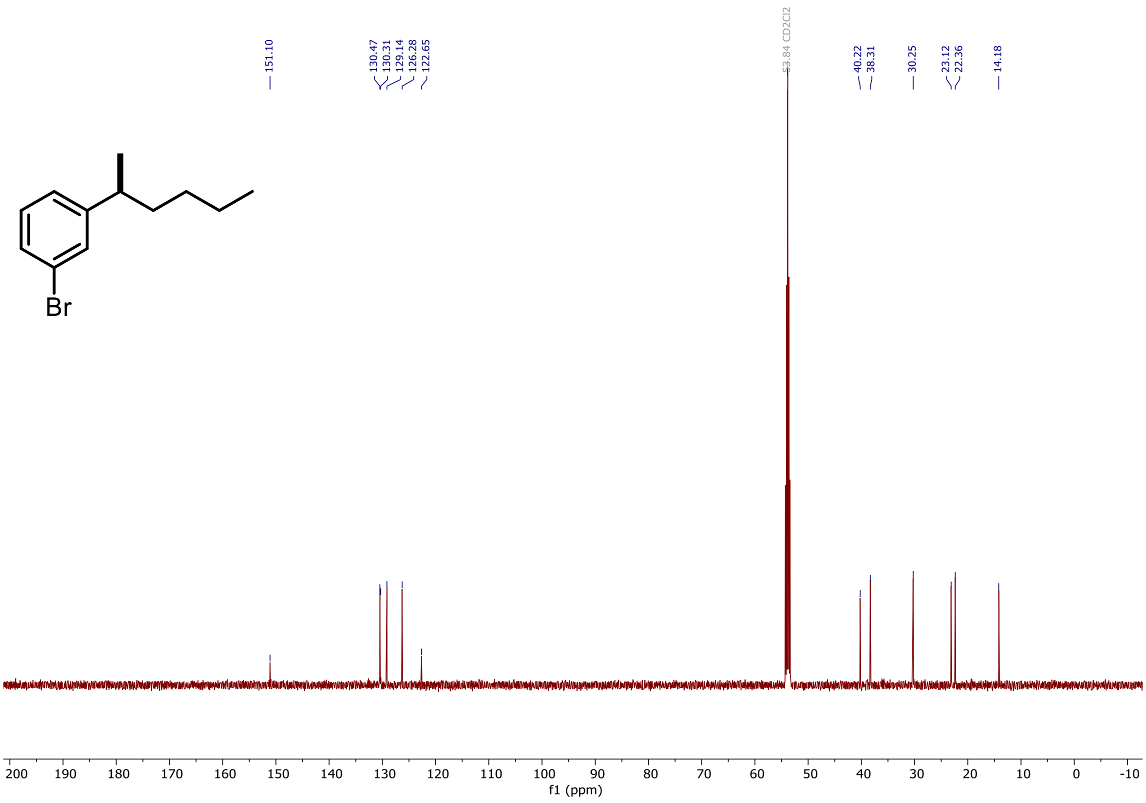

^{1}H NMR spectrum of **2h**

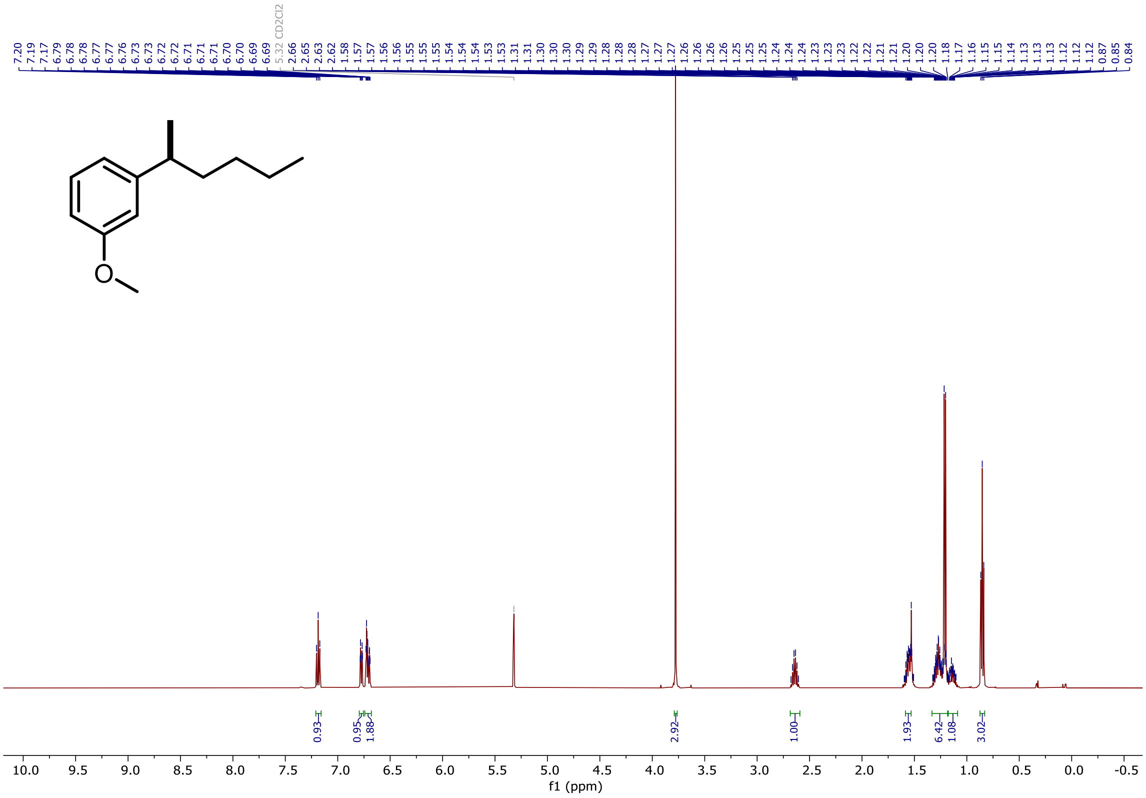

^{13}C NMR spectrum of **2h**

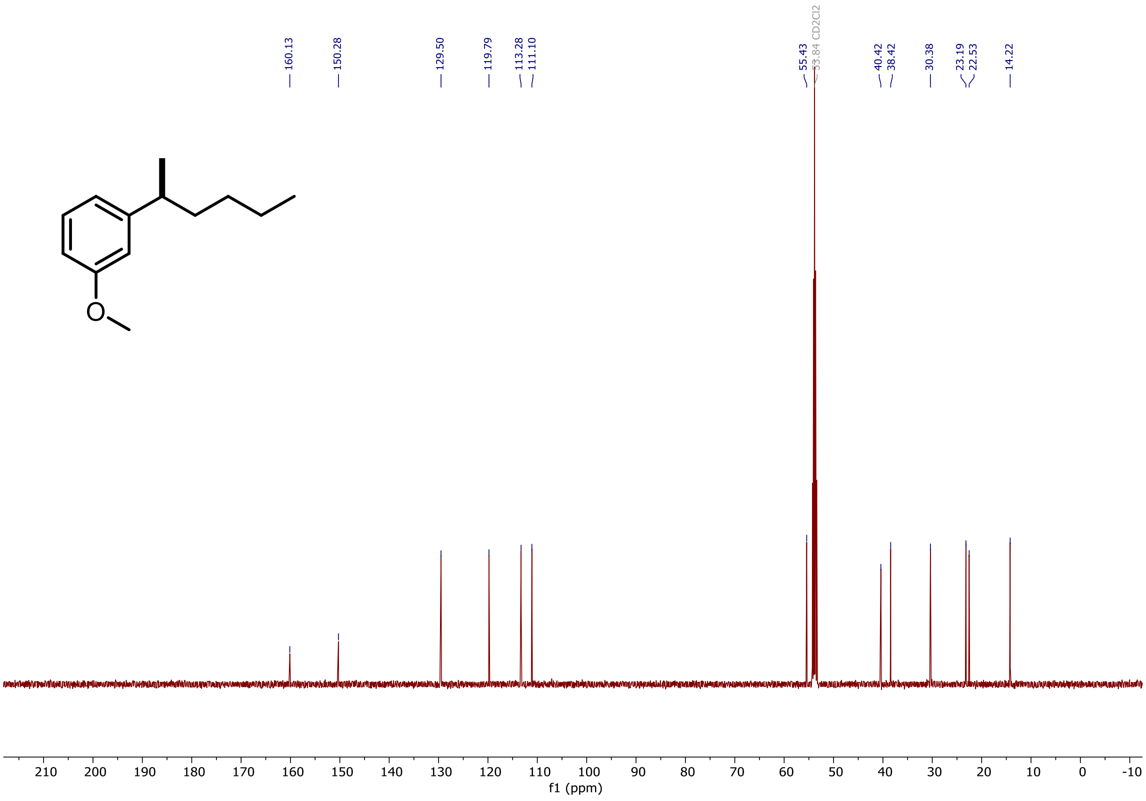

^{1}H NMR spectrum of **2j**

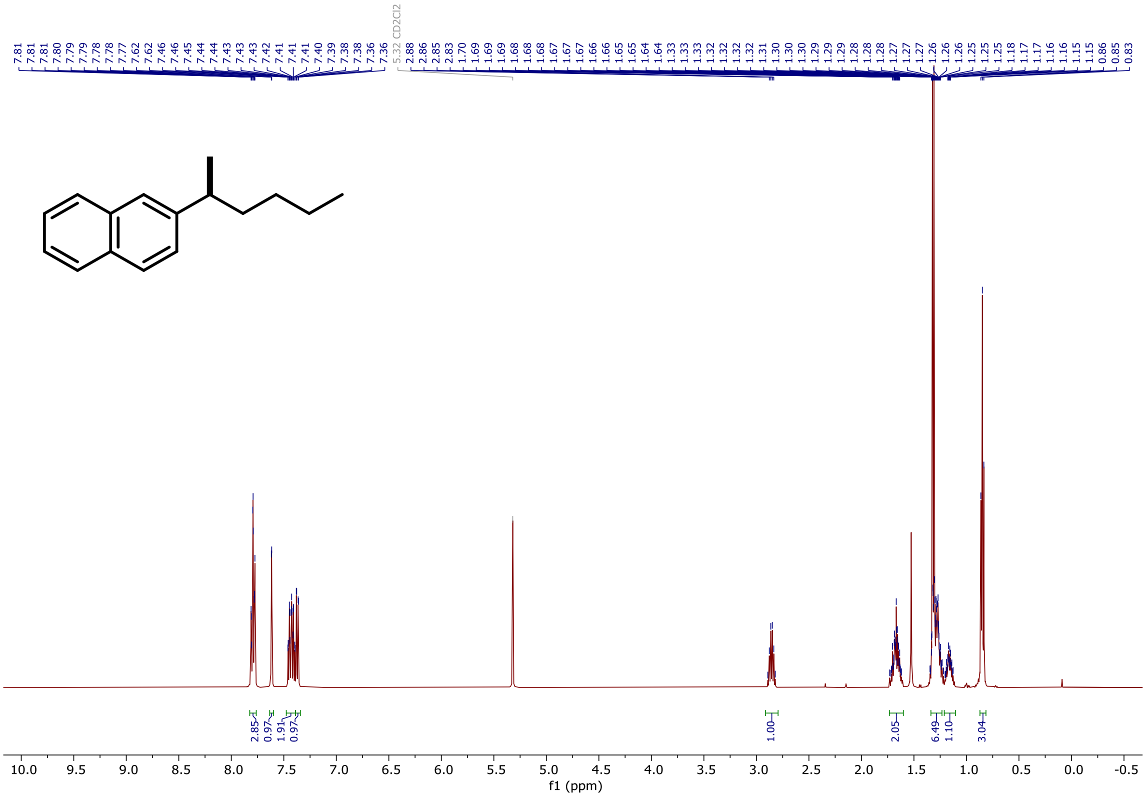

^{13}C NMR spectrum of **2j**

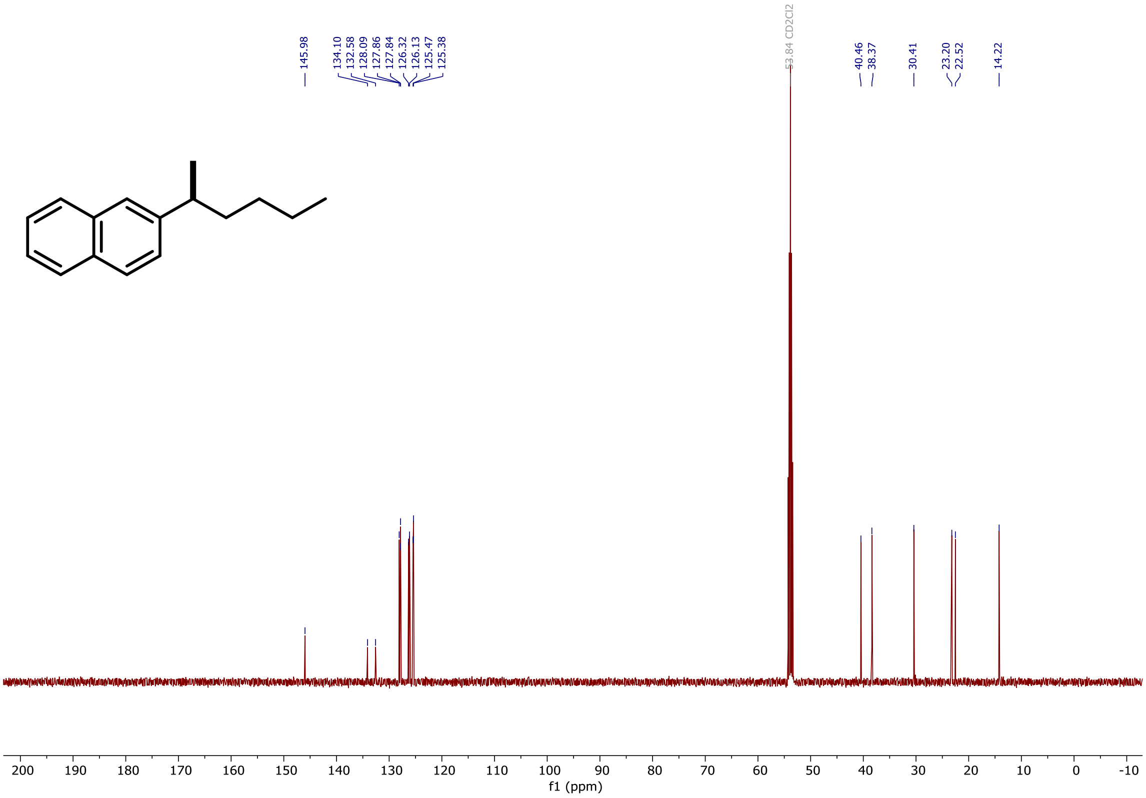

^{1}H NMR spectrum of **2k**

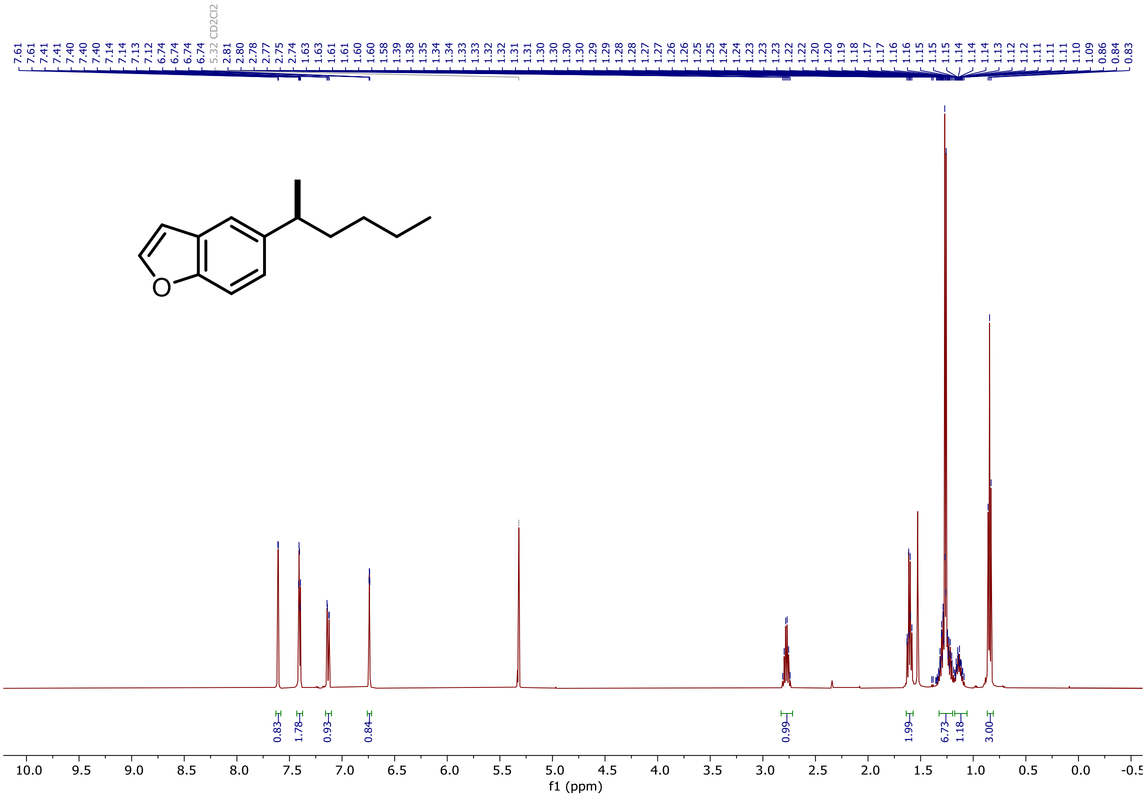

^{13}C NMR spectrum of **2k**

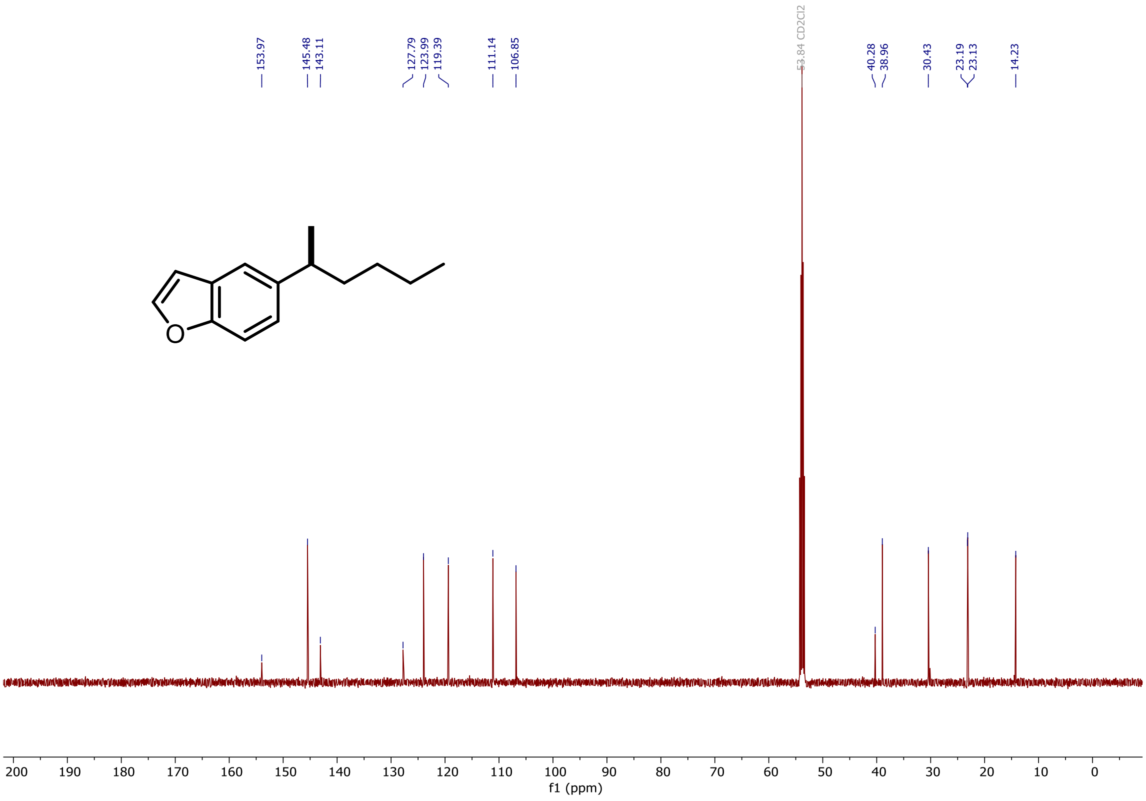

^{1}H NMR spectrum of **2l**

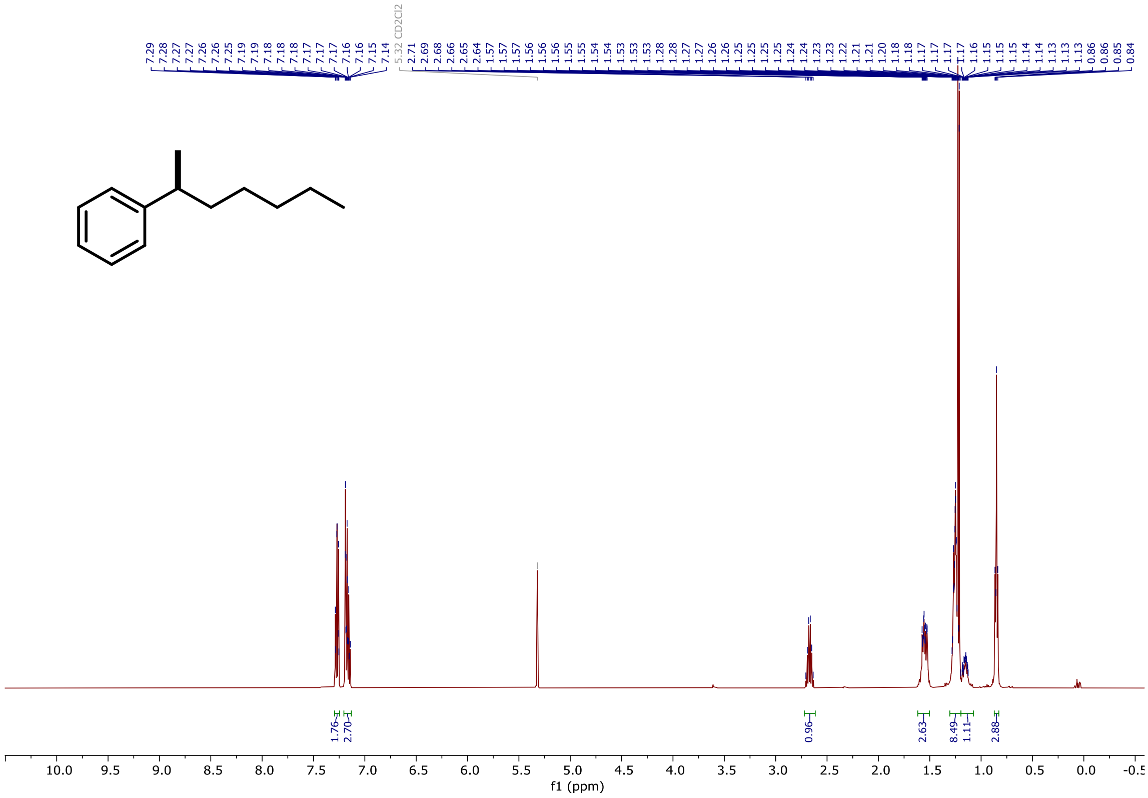

^{13}C NMR spectrum of **2l**

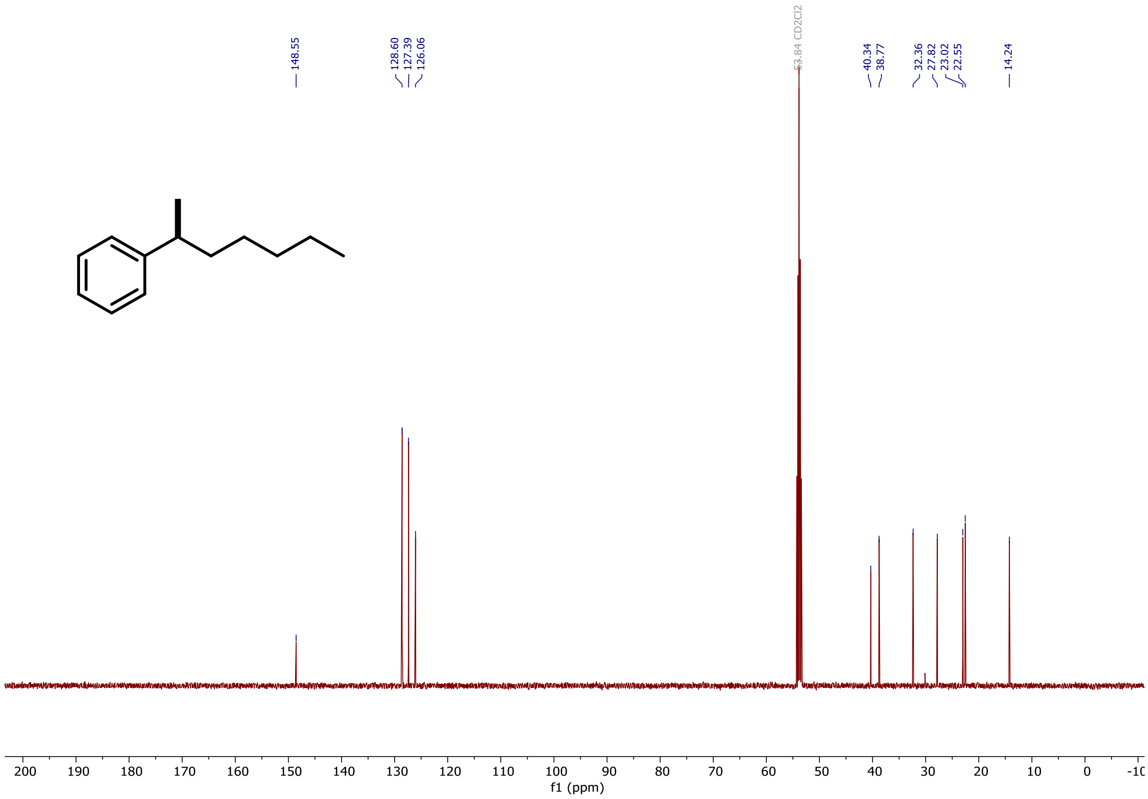

^{1}H NMR spectrum of **2m**

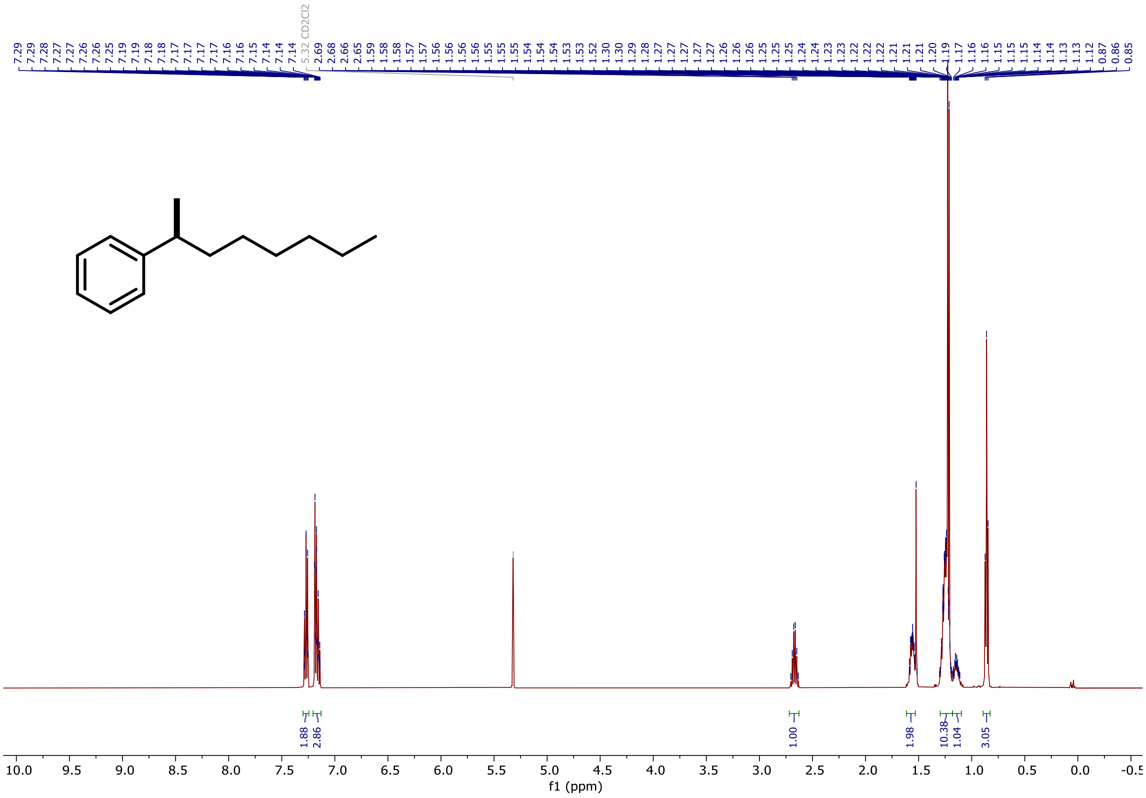

^{13}C NMR spectrum of **2m**

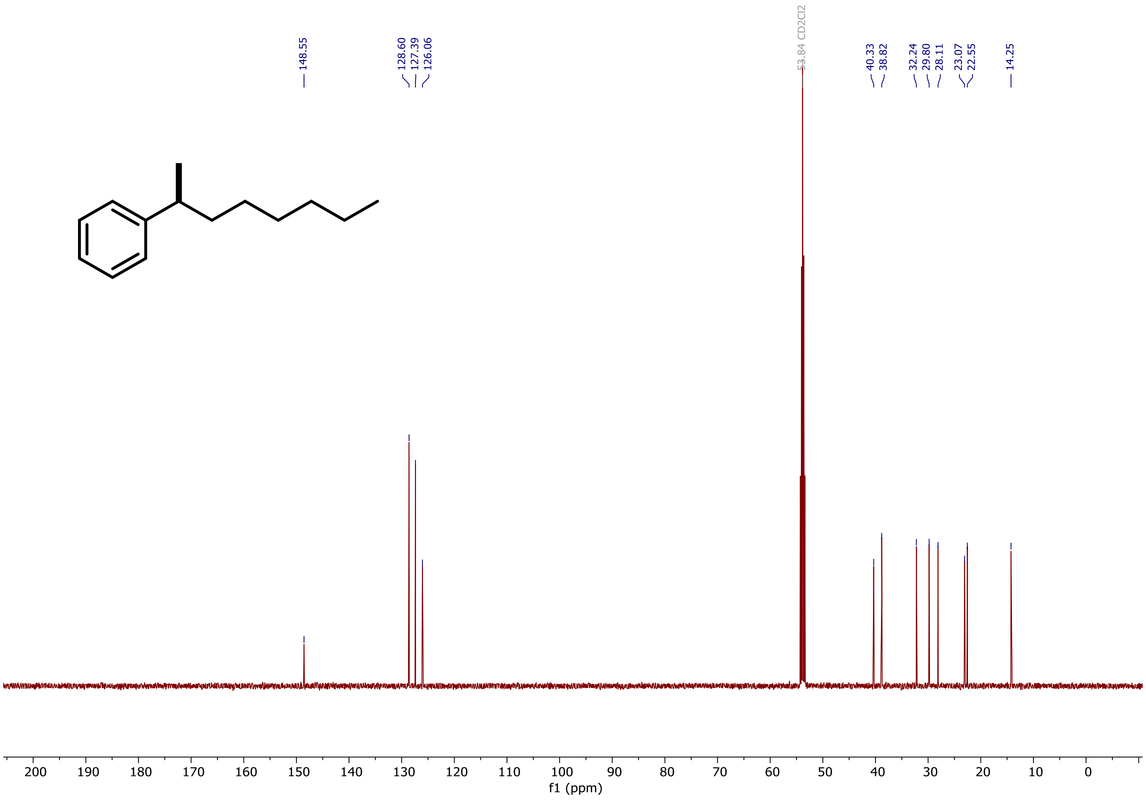

^{1}H NMR spectrum of **2n**

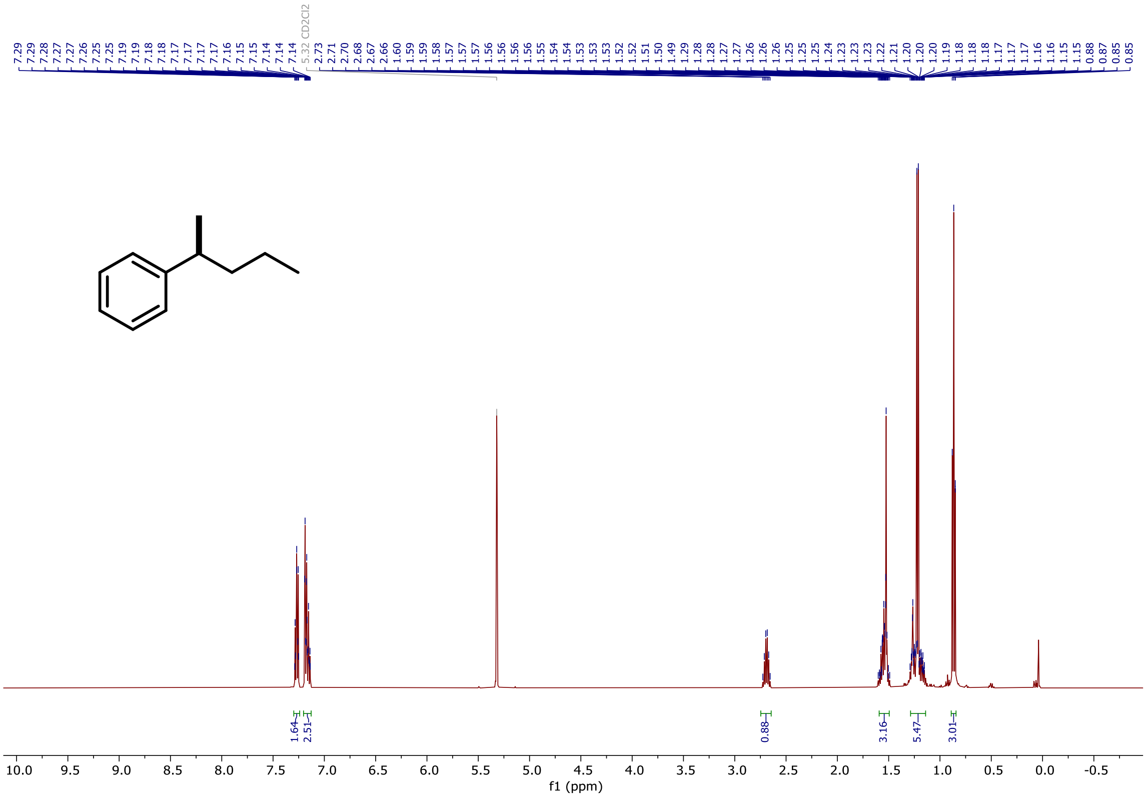

^{13}C NMR spectrum of **2n**

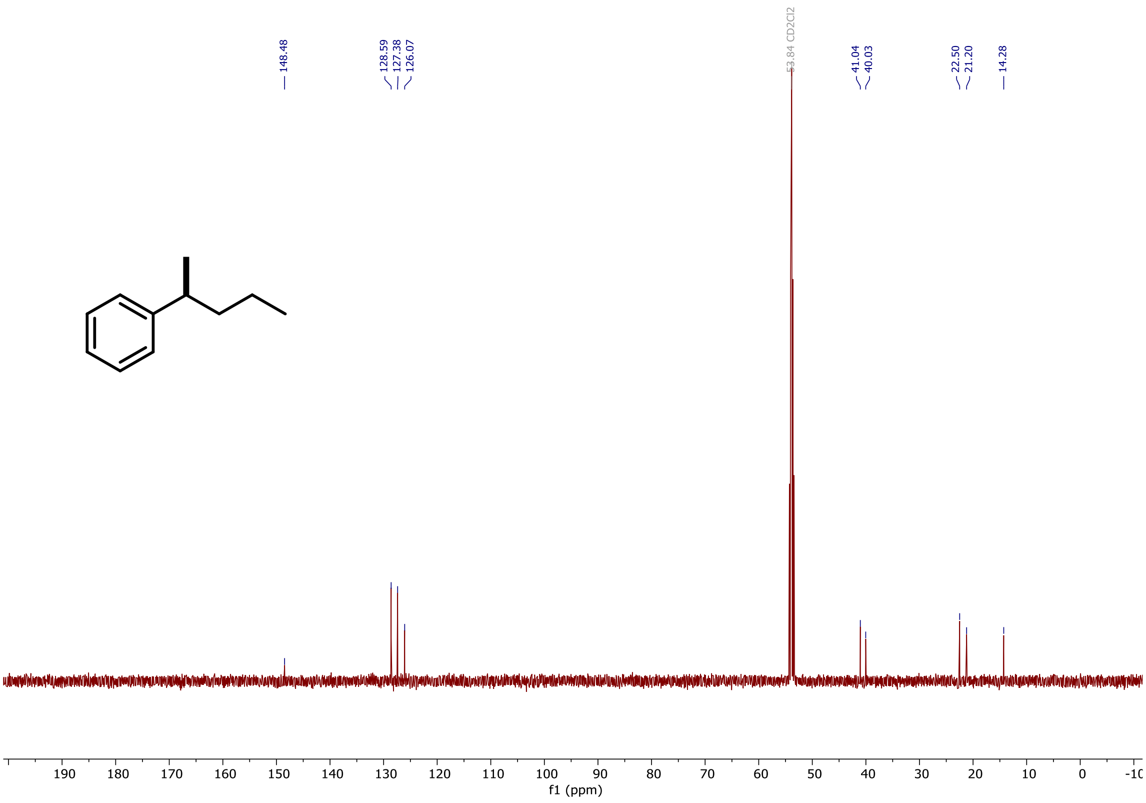

^{1}H NMR spectrum of **2o**

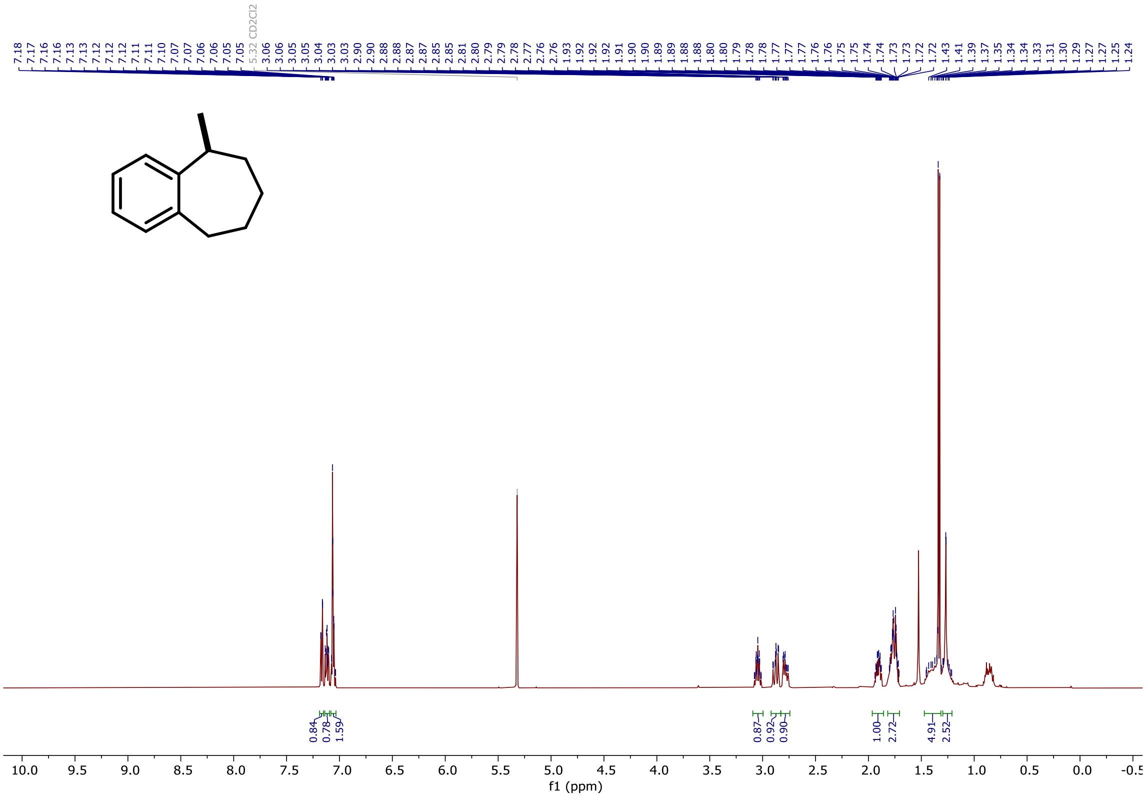

^{13}C NMR spectrum of **2o**

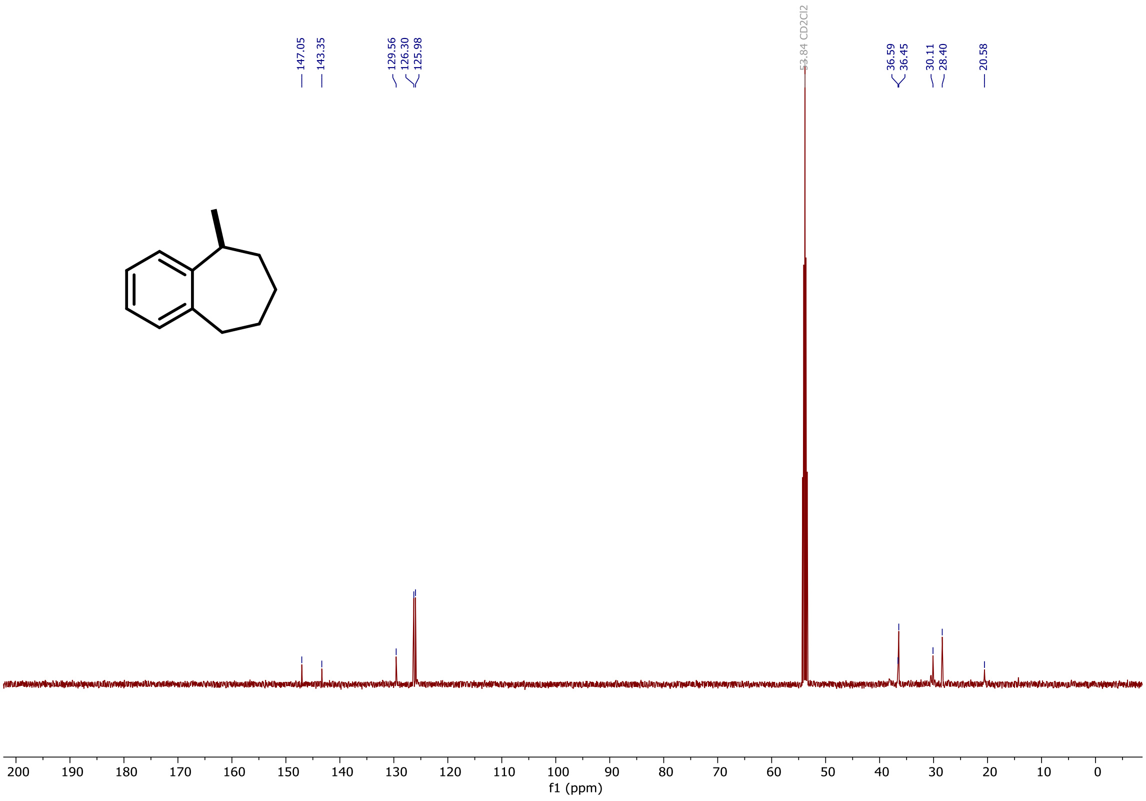

^{1}H NMR spectrum of rac-**2p**

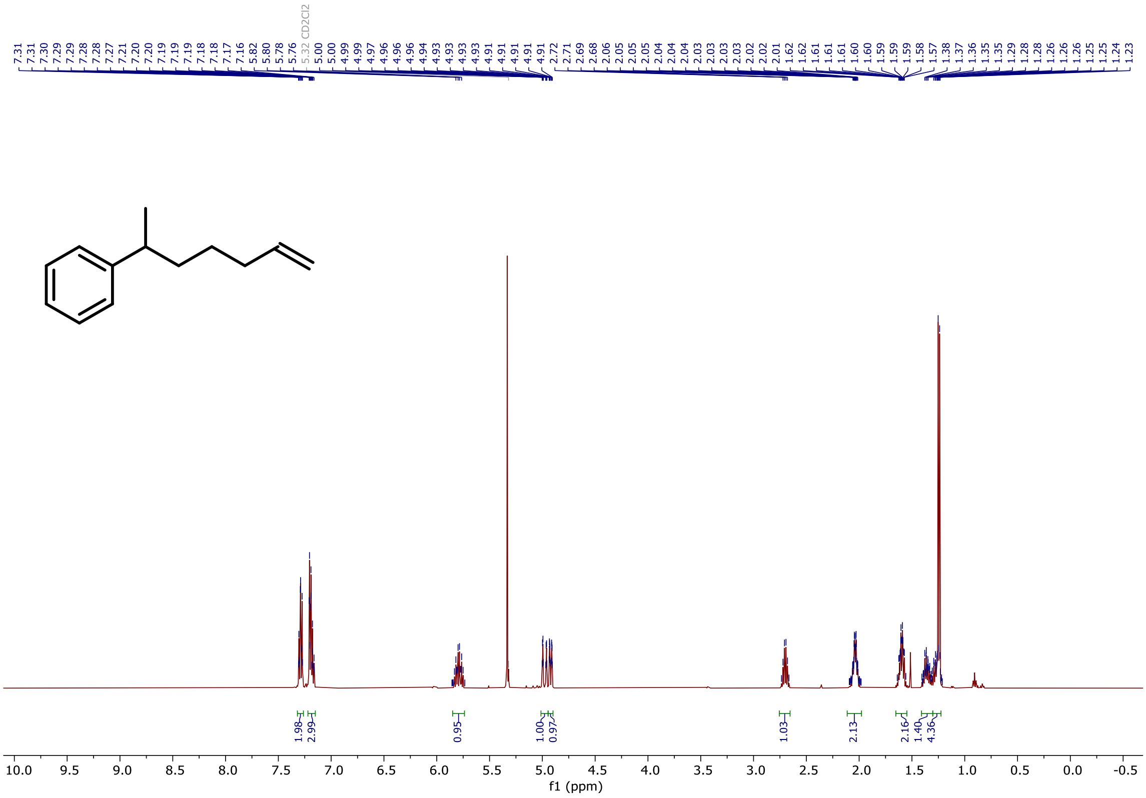

^{13}C NMR spectrum of rac-**2p**

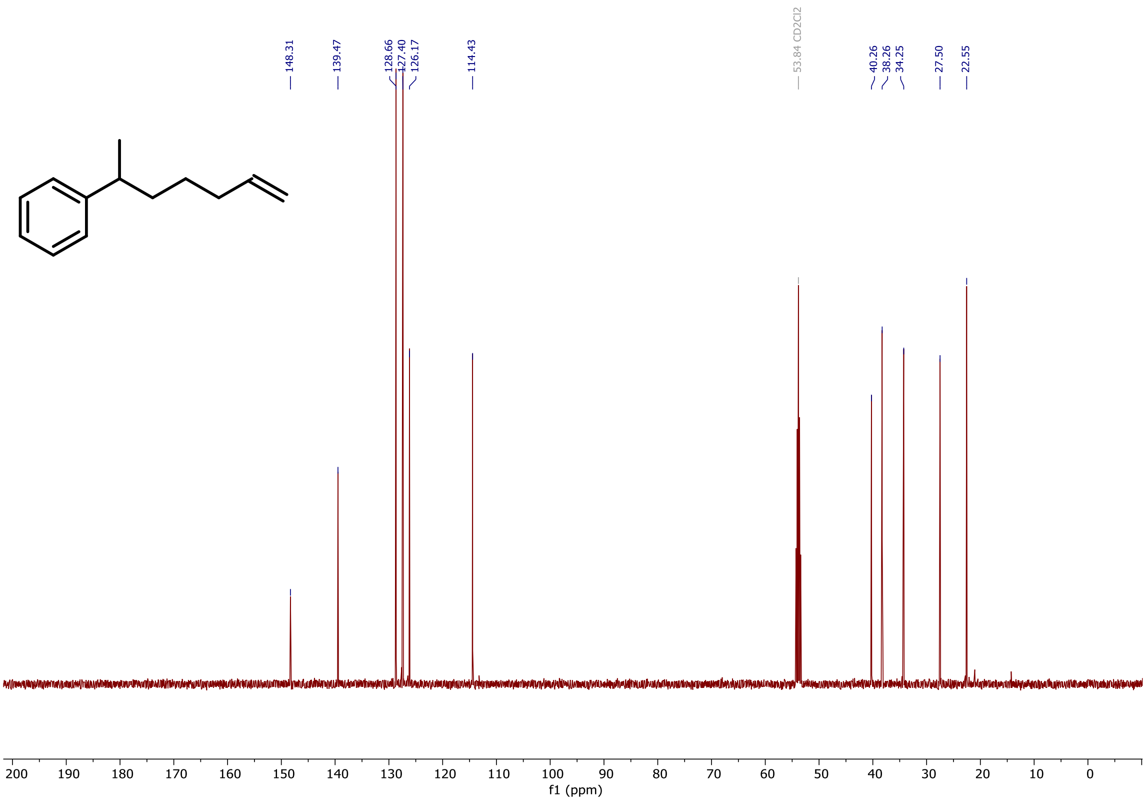

^{1}H NMR spectrum of **2q**

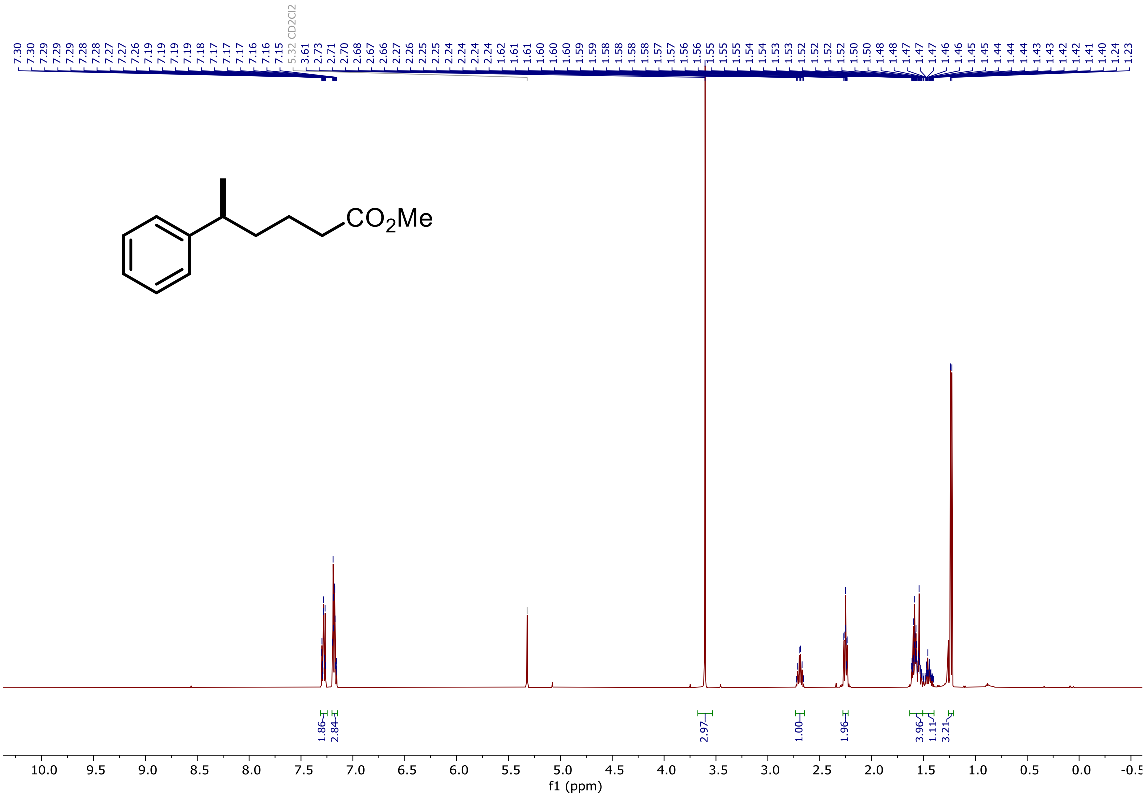

^{13}C NMR spectrum of **2q**

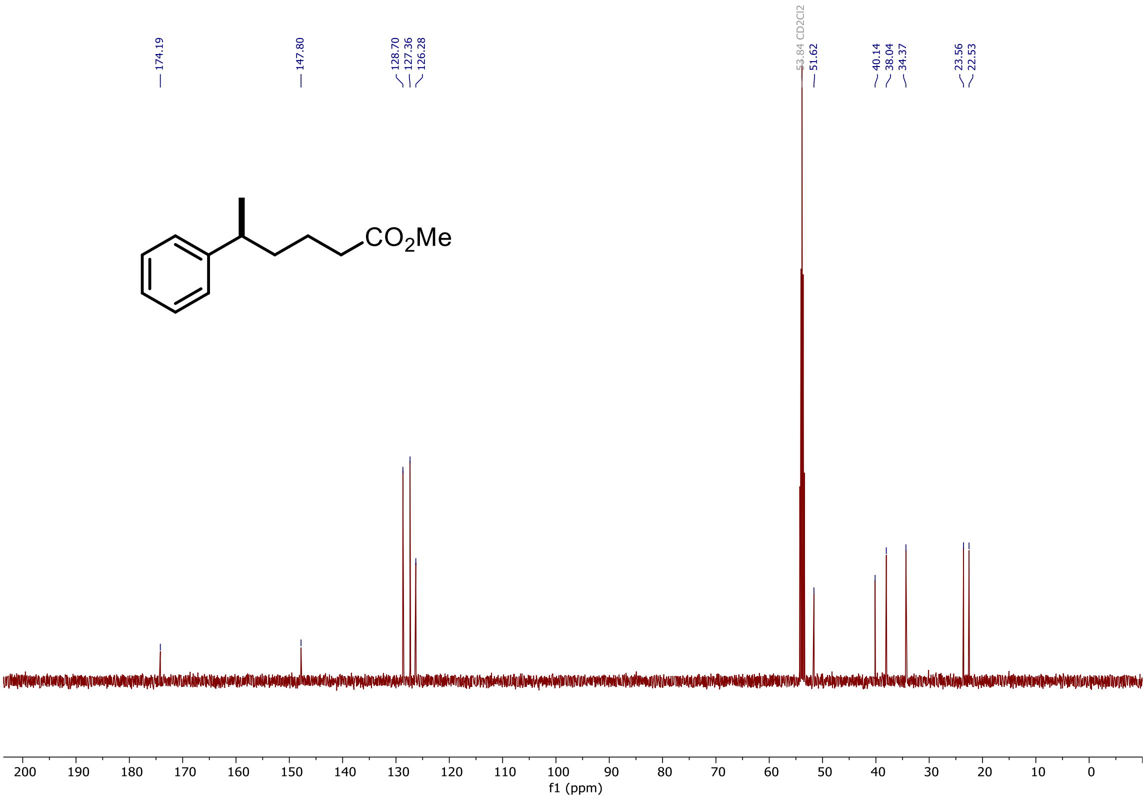

^{1}H NMR spectrum of **2r**

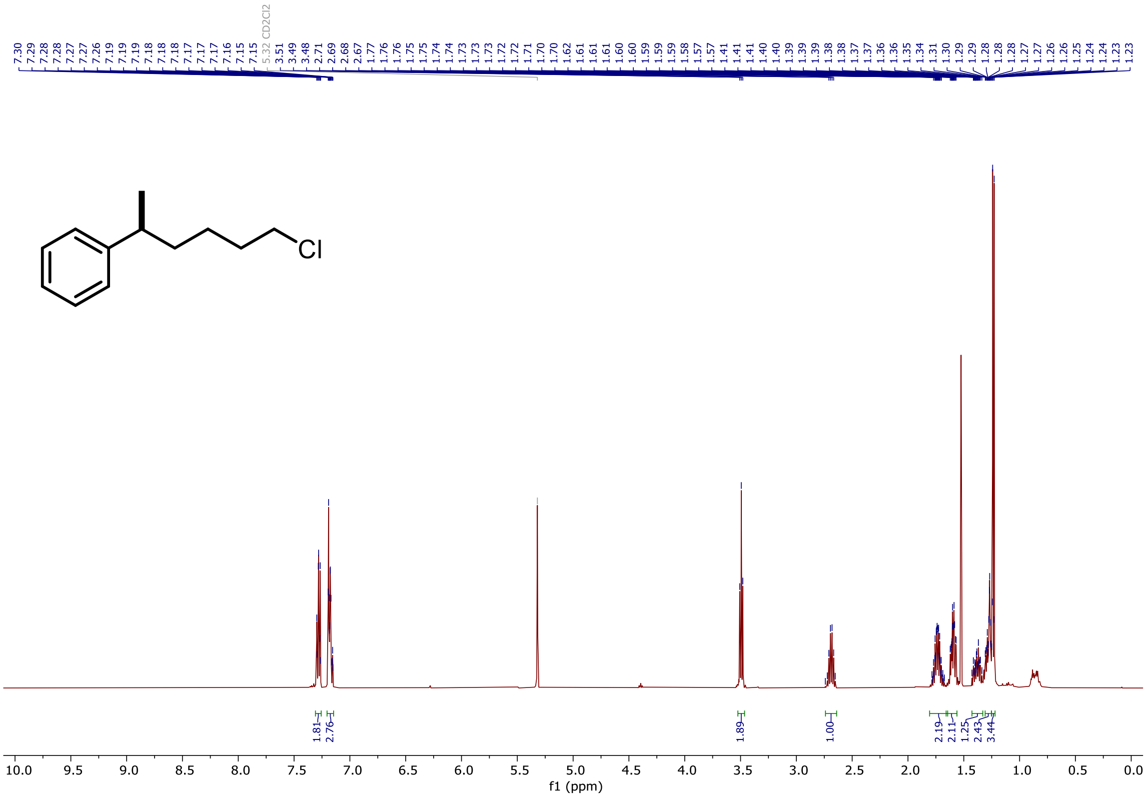

^{13}C NMR spectrum of **2r**

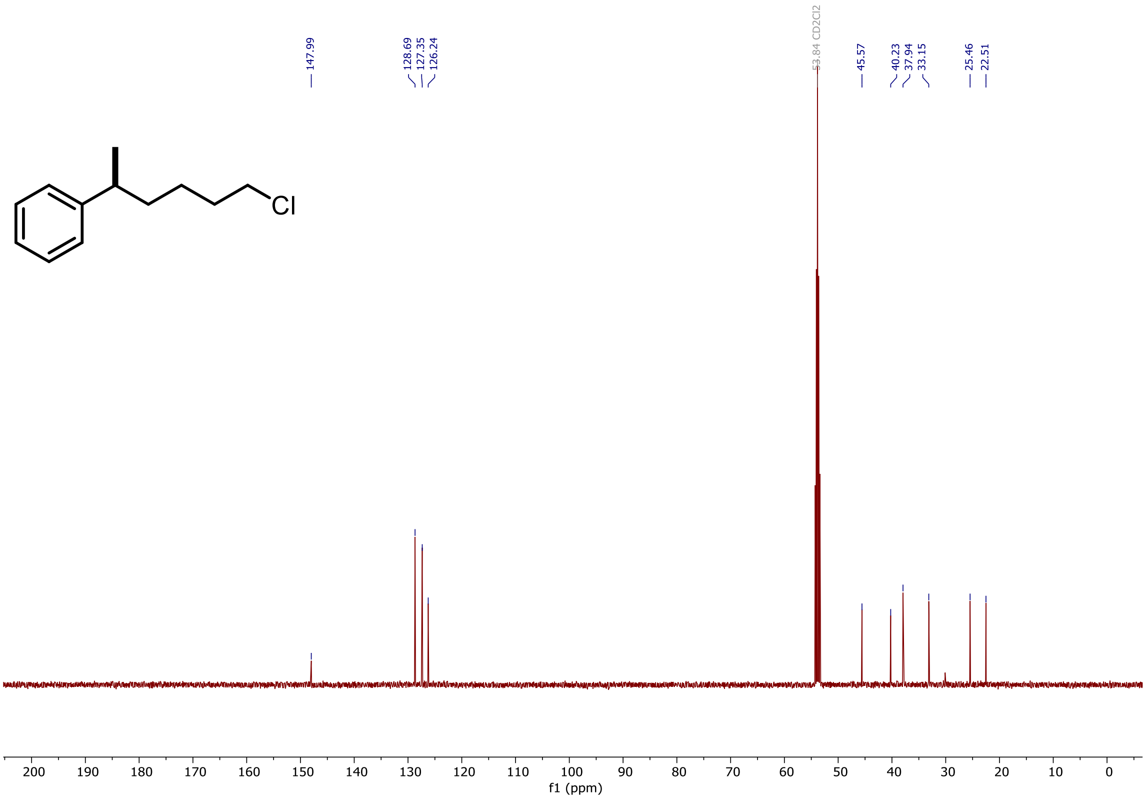

^{1}H NMR spectrum of **S7**

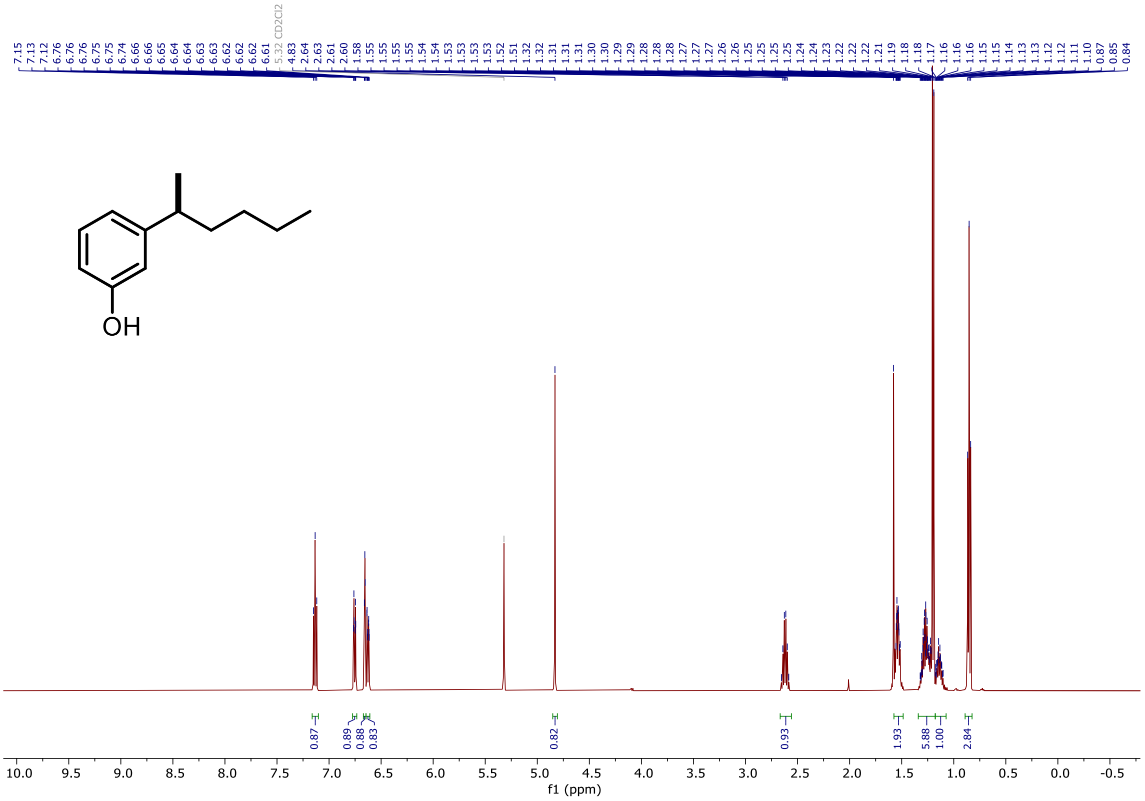

^{13}C NMR spectrum of **S7**

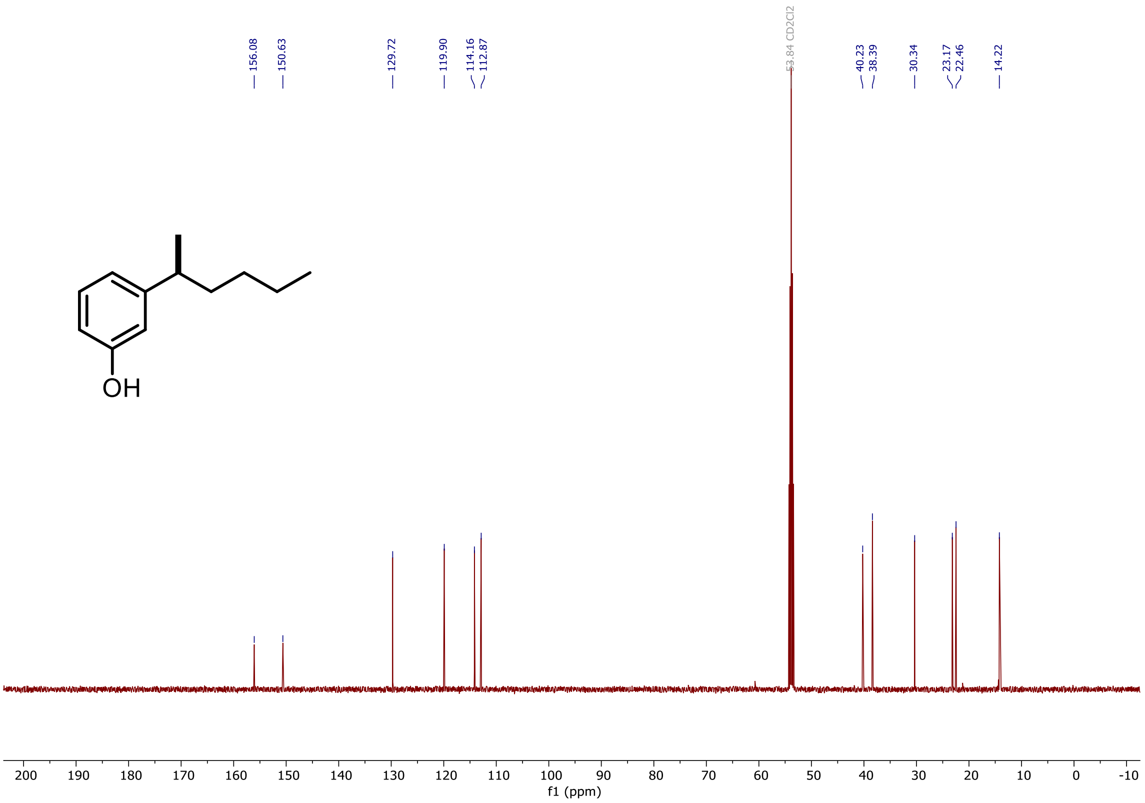

^{1}H NMR spectrum of **2i**

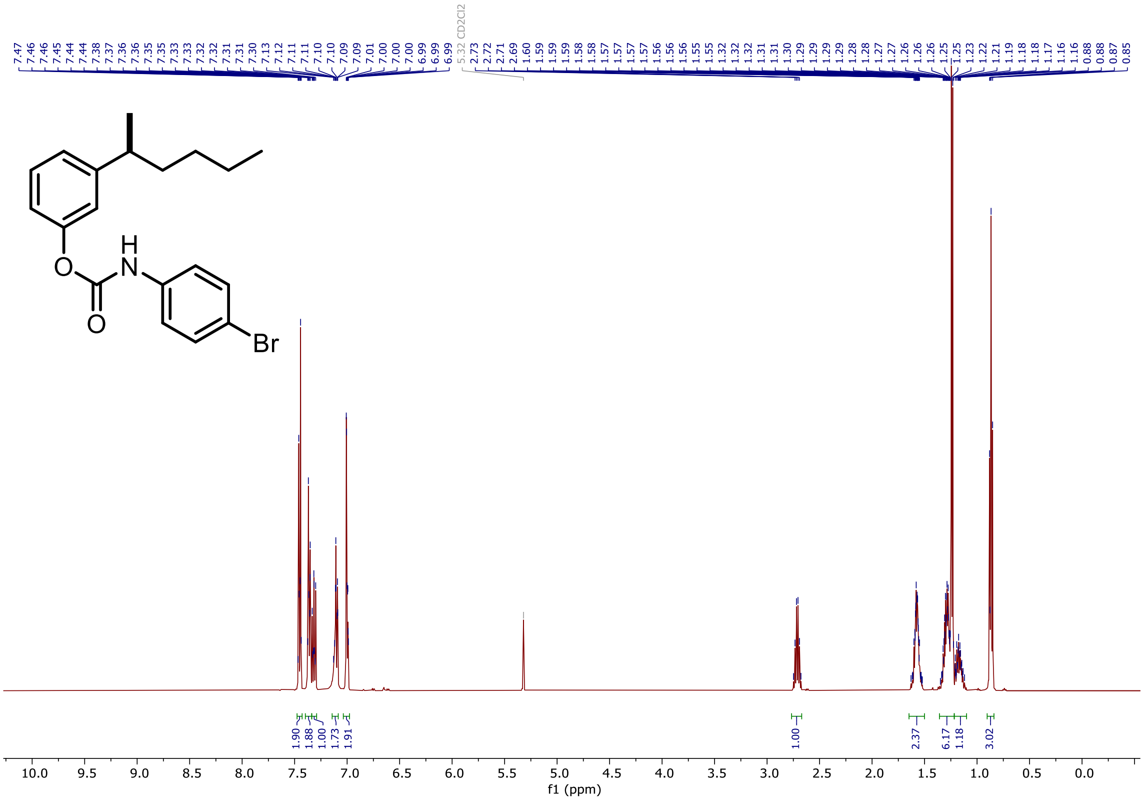

^{13}C NMR spectrum of **2i**

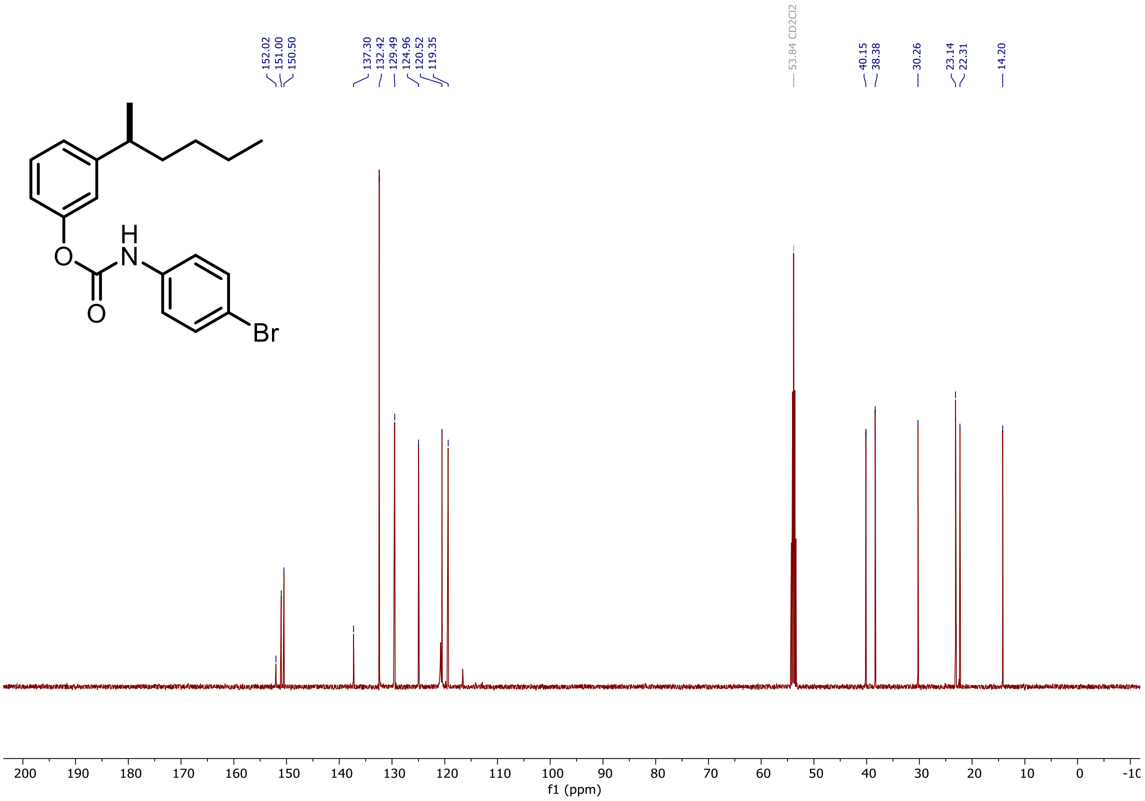

^{1}H NMR spectrum of **S8**

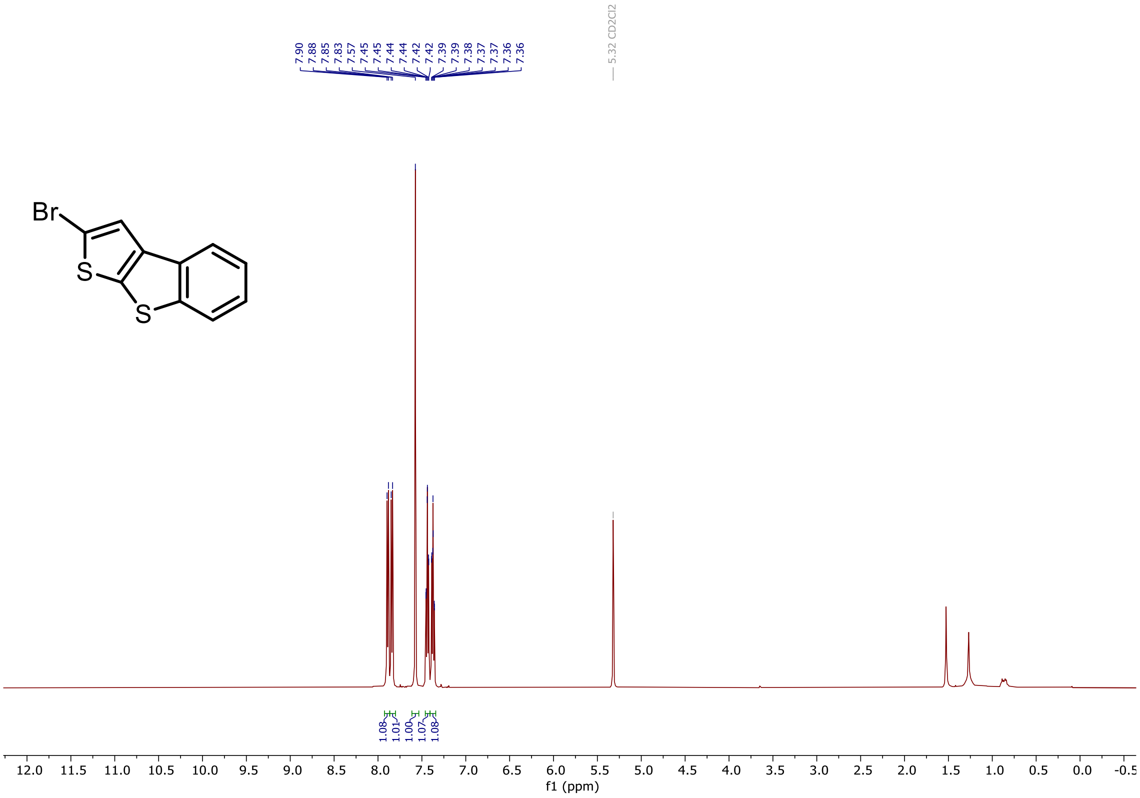

^{13}C NMR spectrum of **S8**

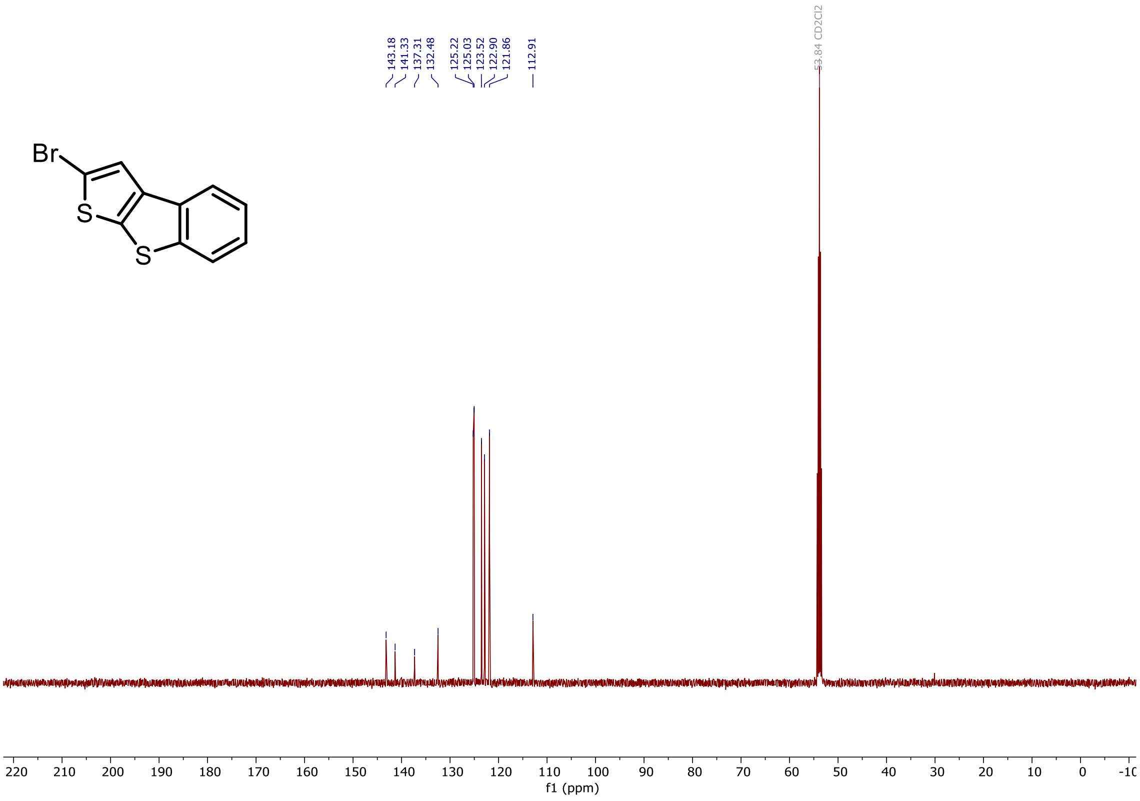

^{1}H NMR spectrum of **S10**

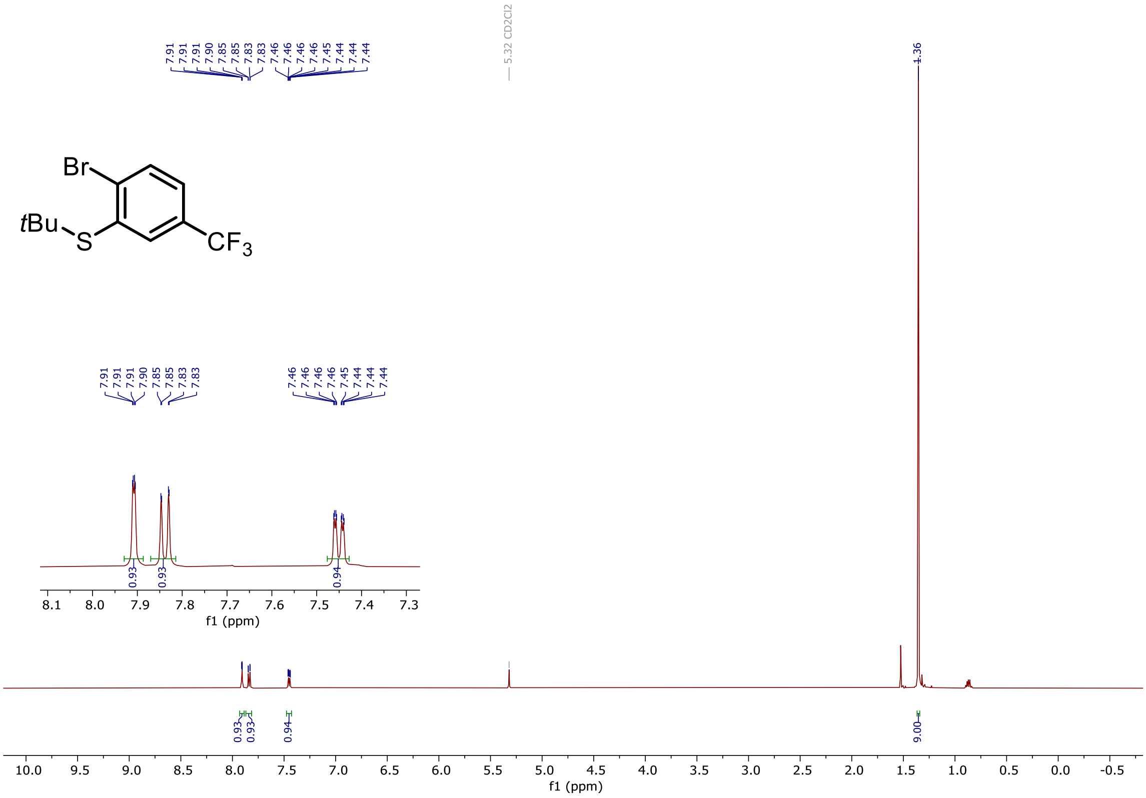

^{19}F NMR spectrum of **S10**

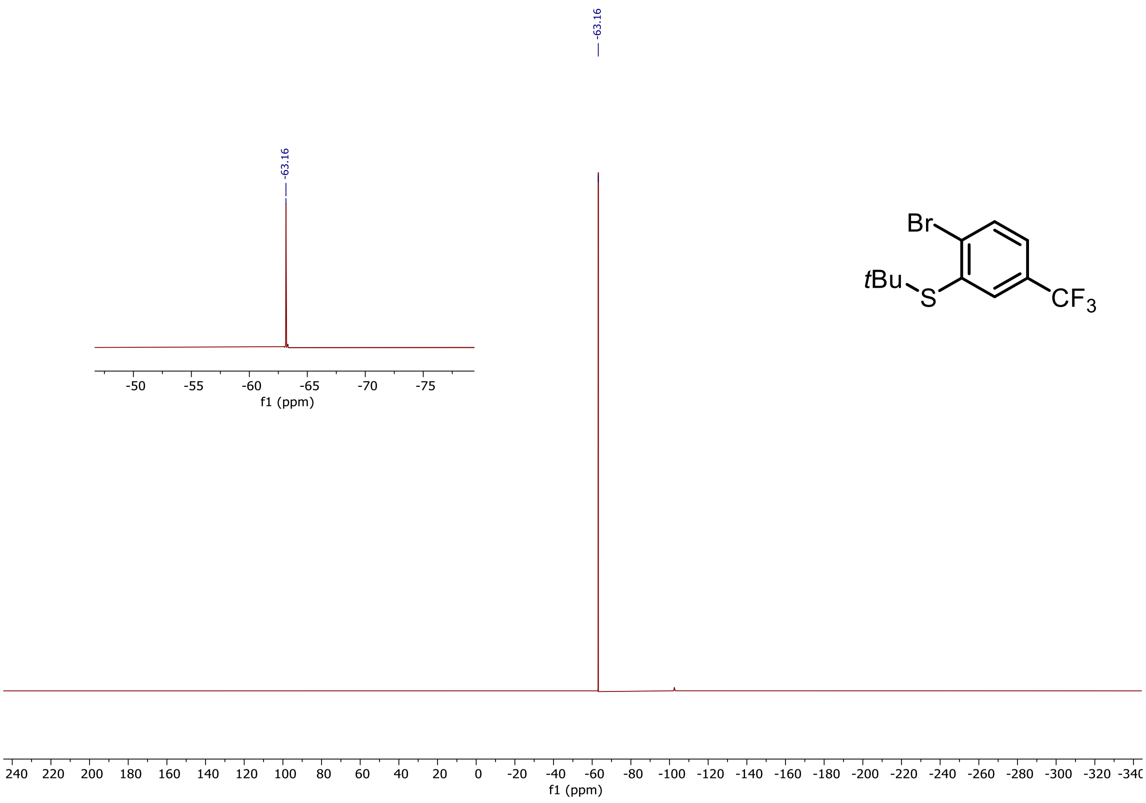

^{13}C NMR spectrum of **S10**

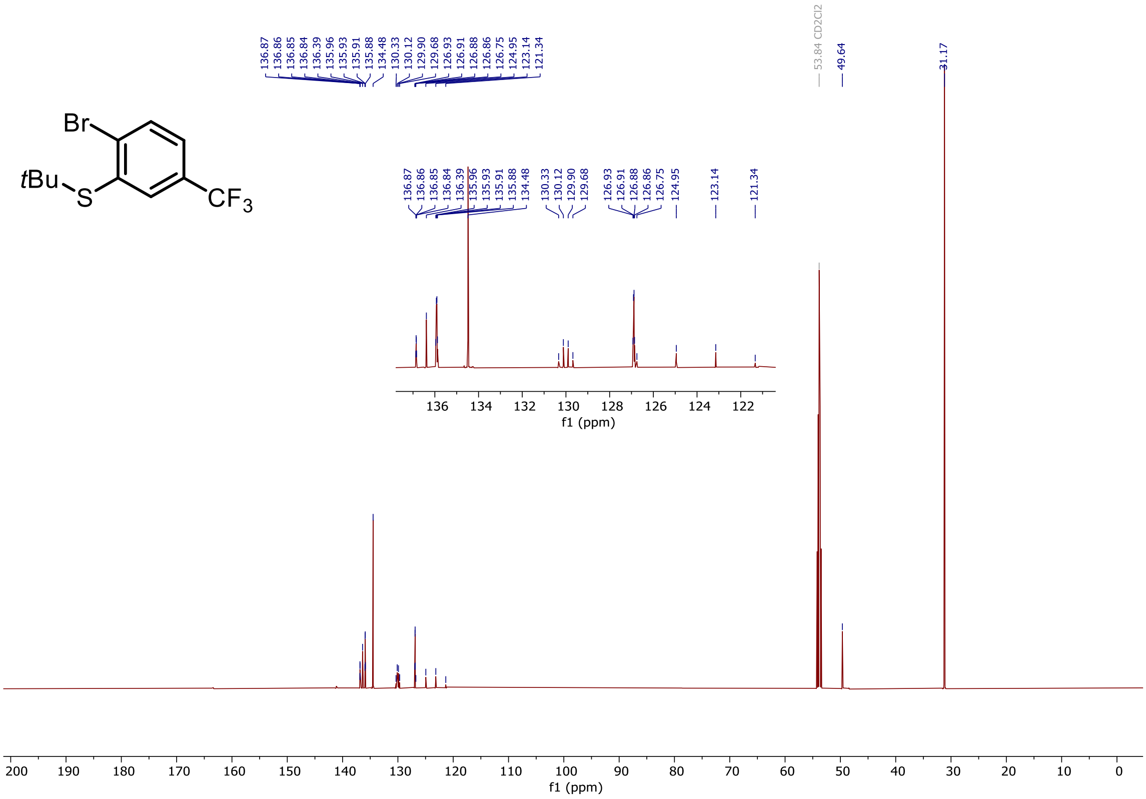

^{1}H NMR spectrum of **S11**

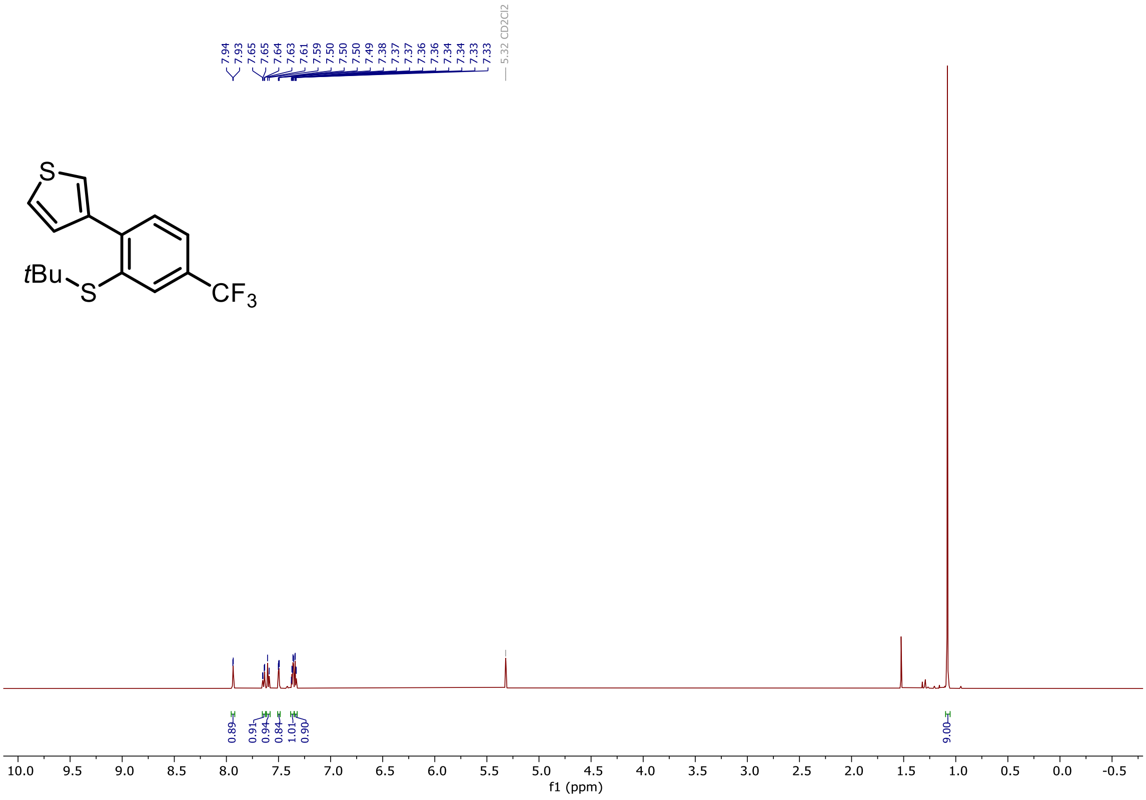

^{19}F NMR spectrum of **S11**

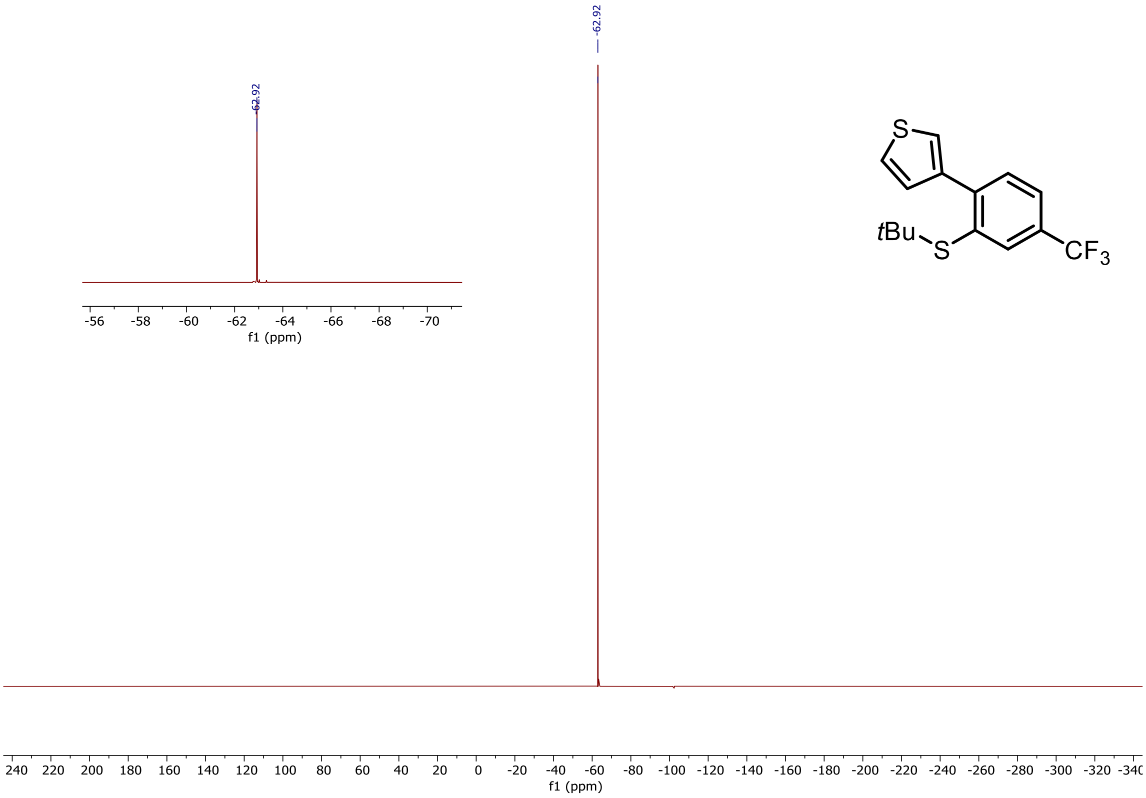

^{13}C NMR spectrum of **S11**

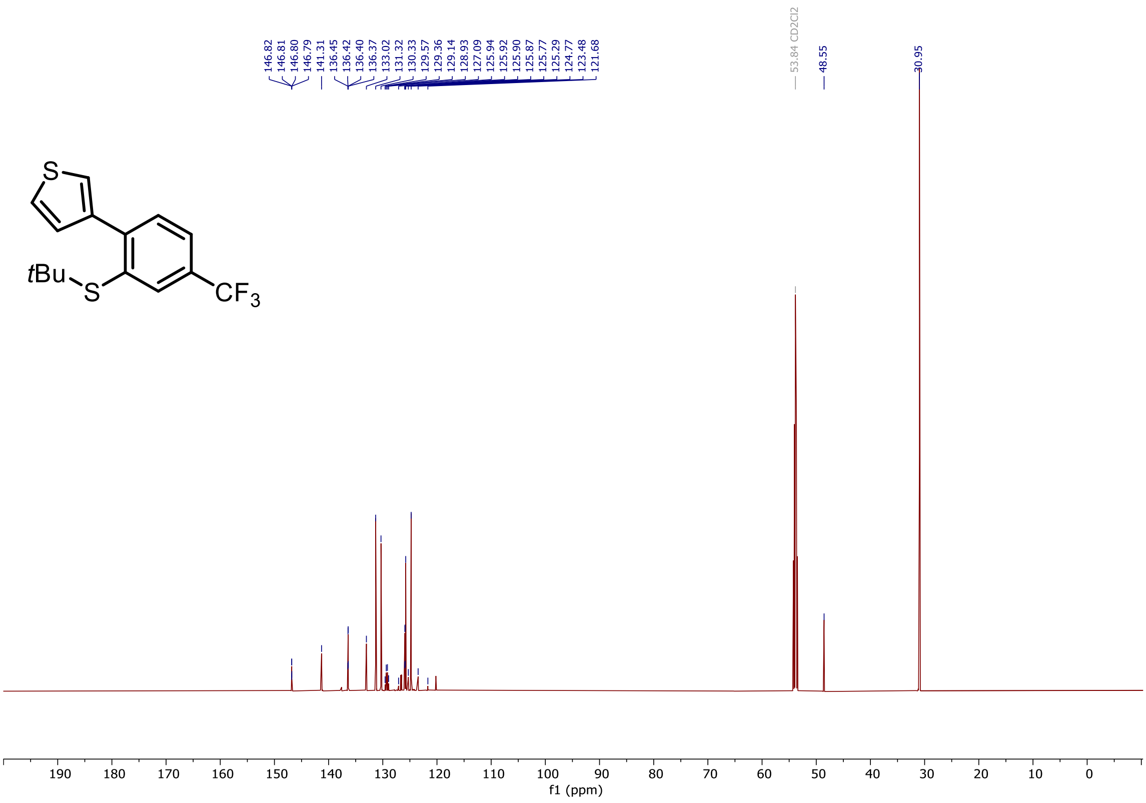

^{1}H NMR spectrum of **S9**

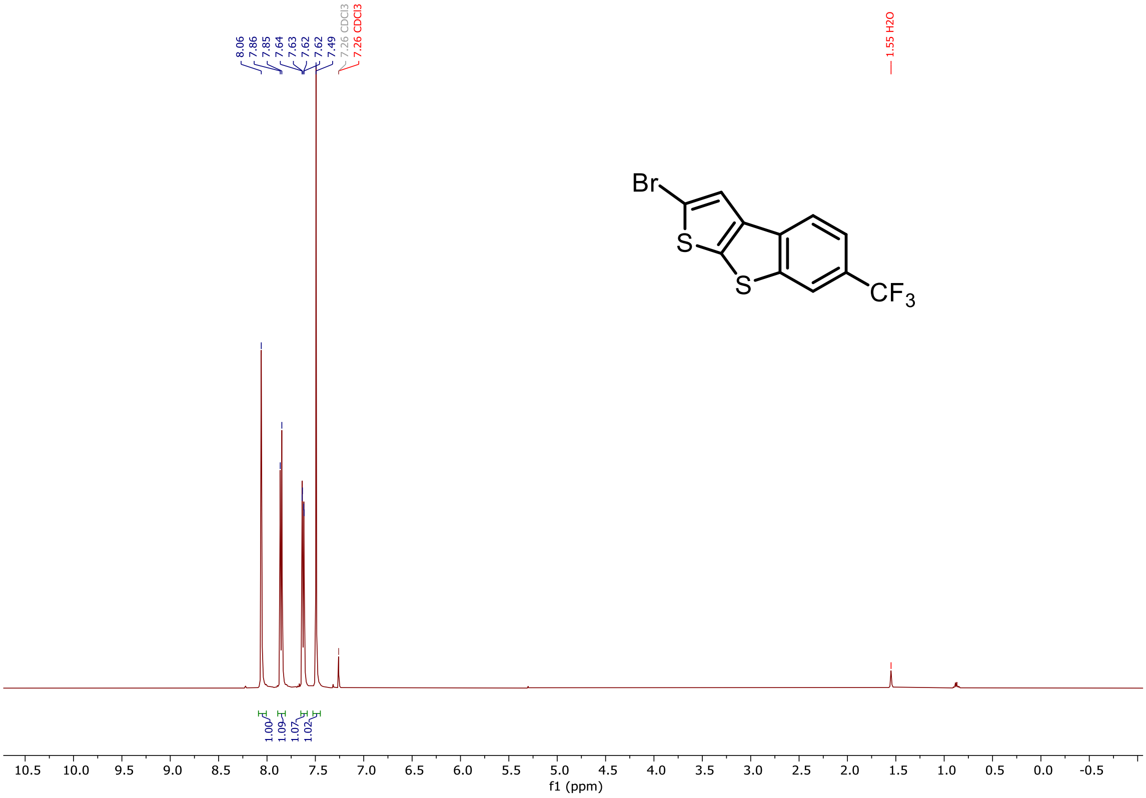

^{19}F NMR spectrum of **S9**

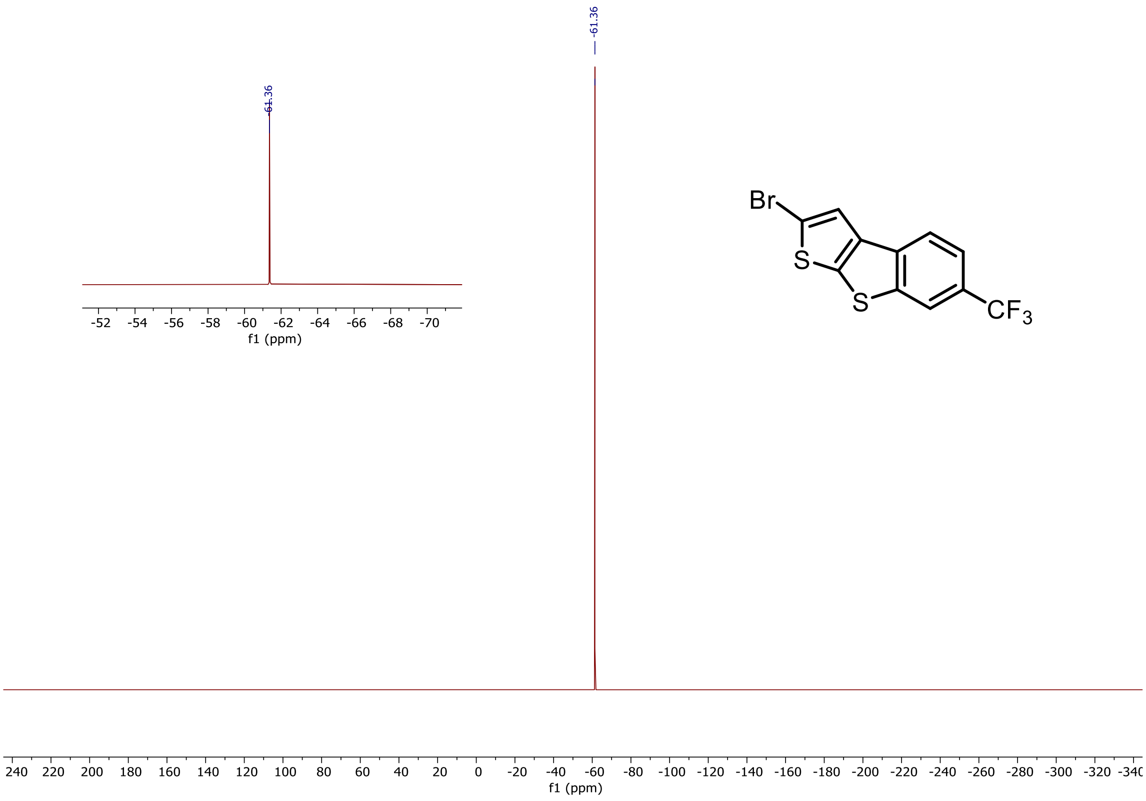

^{13}C NMR spectrum of **S9**

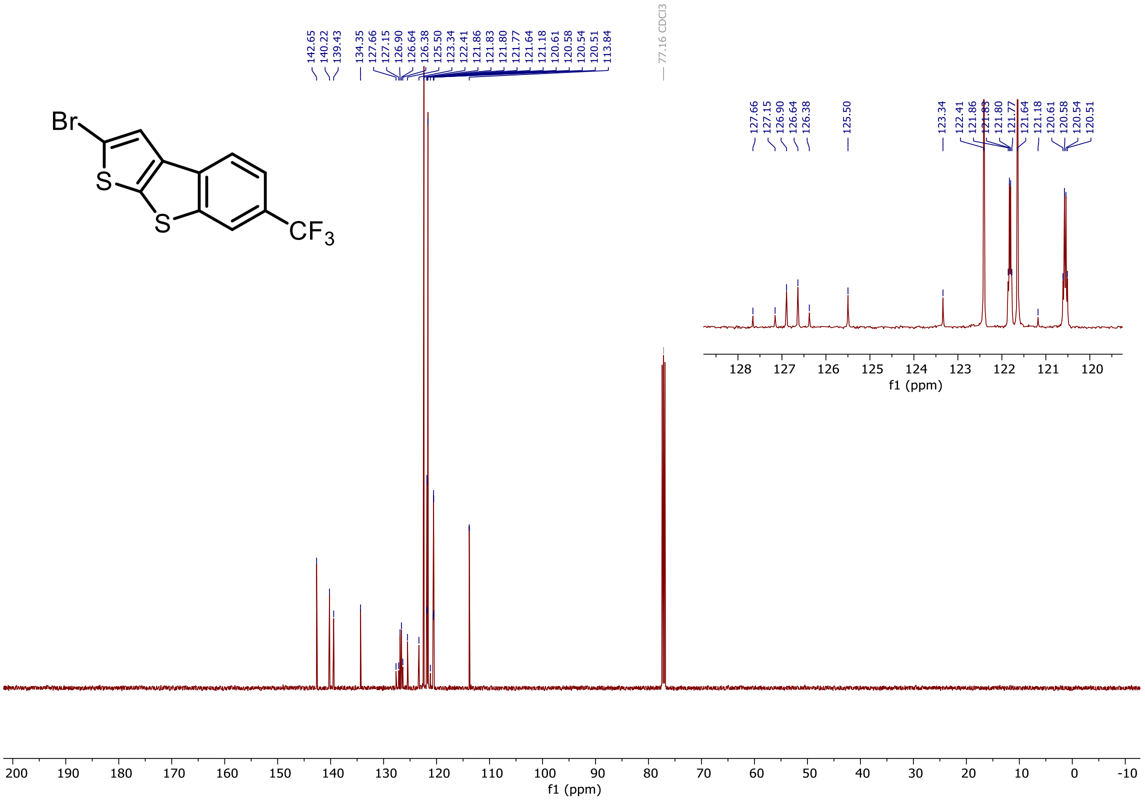

^{1}H NMR spectrum of **S12**

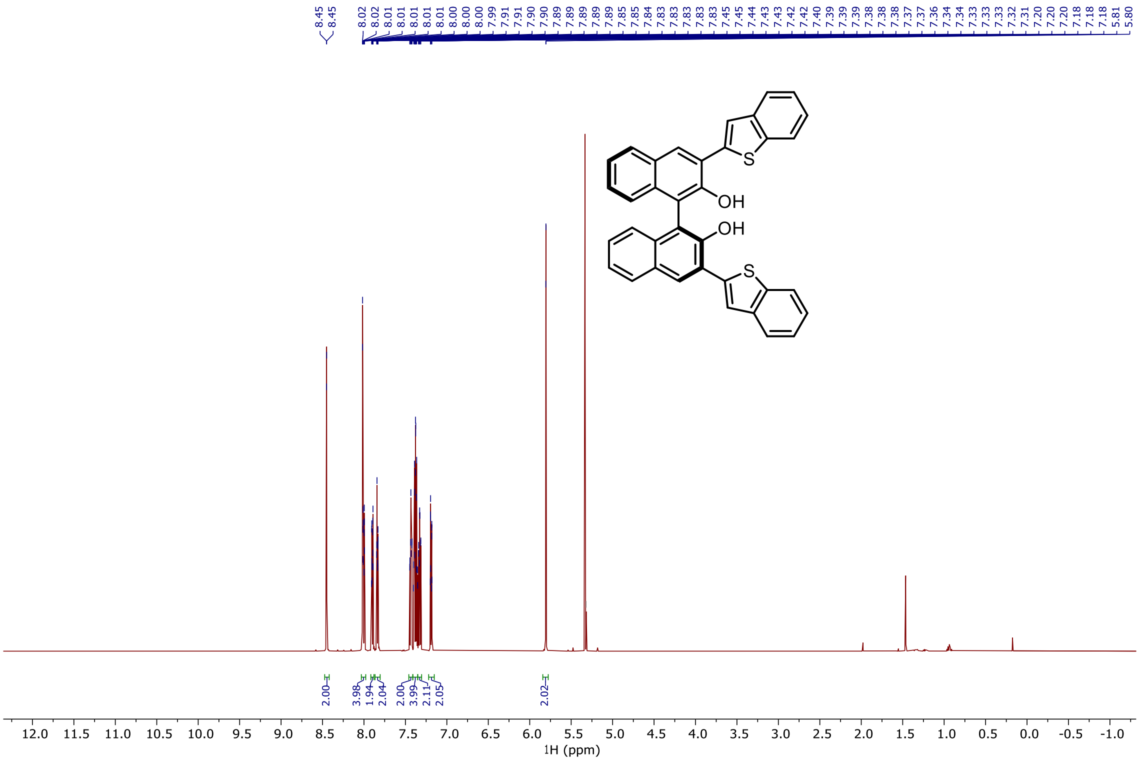

^{13}C NMR spectrum of **S12**

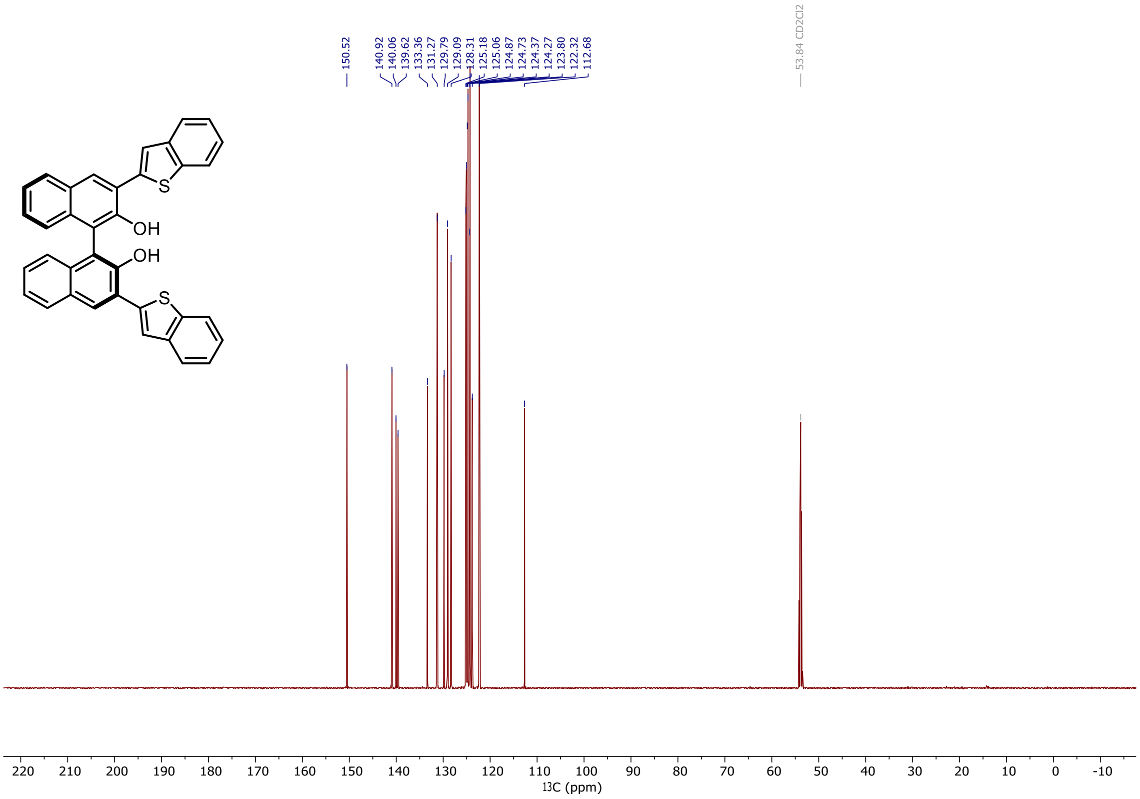

^{1}H NMR spectrum of **S13**

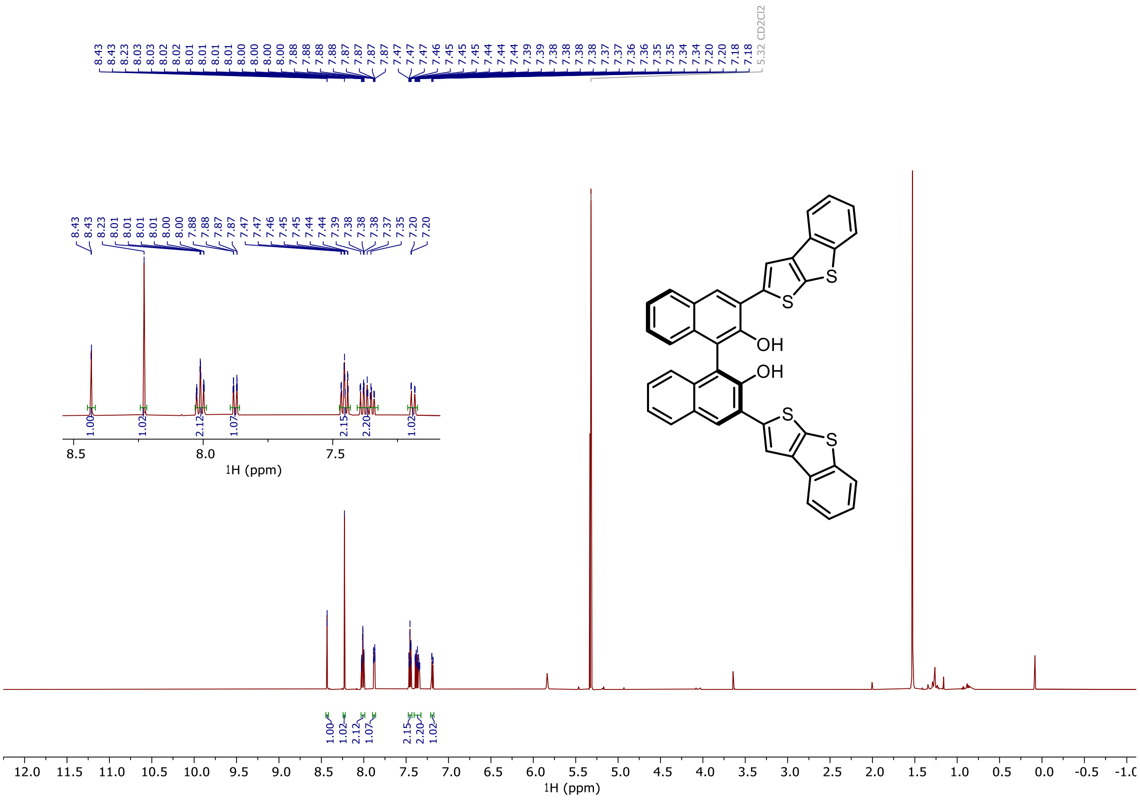

^{13}C NMR spectrum of **S13**

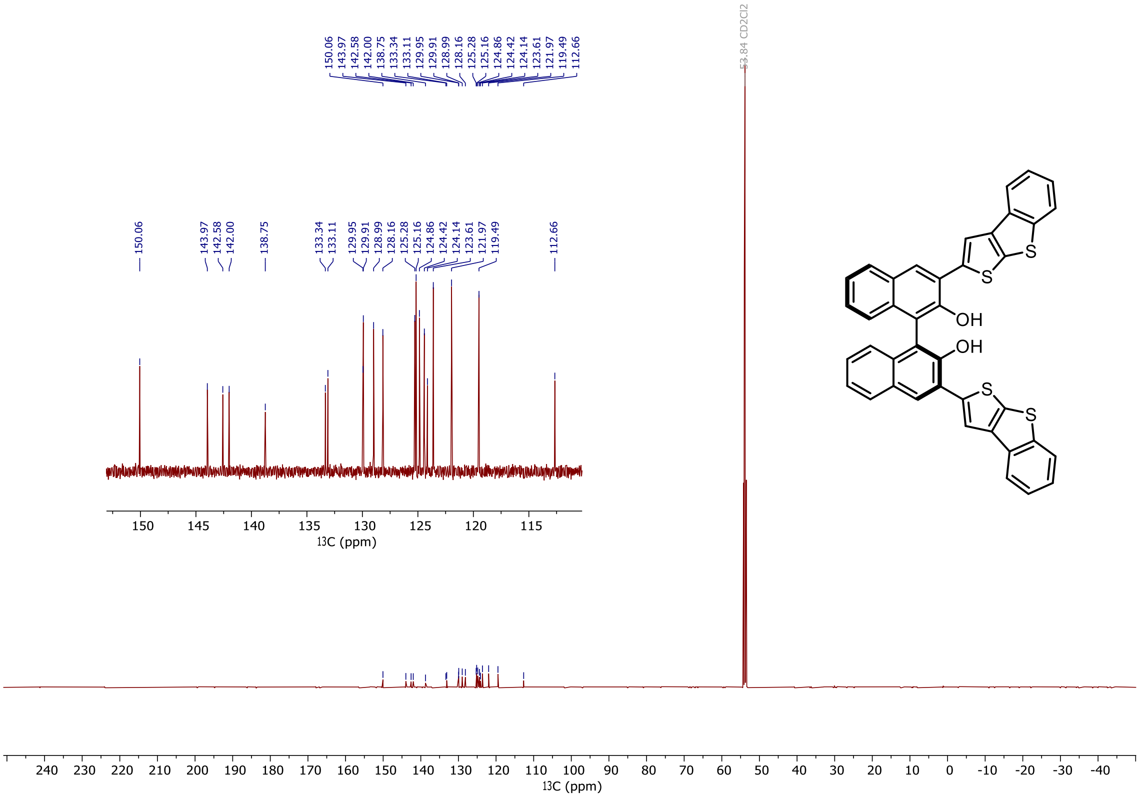

^{1}H NMR spectrum of **S14**

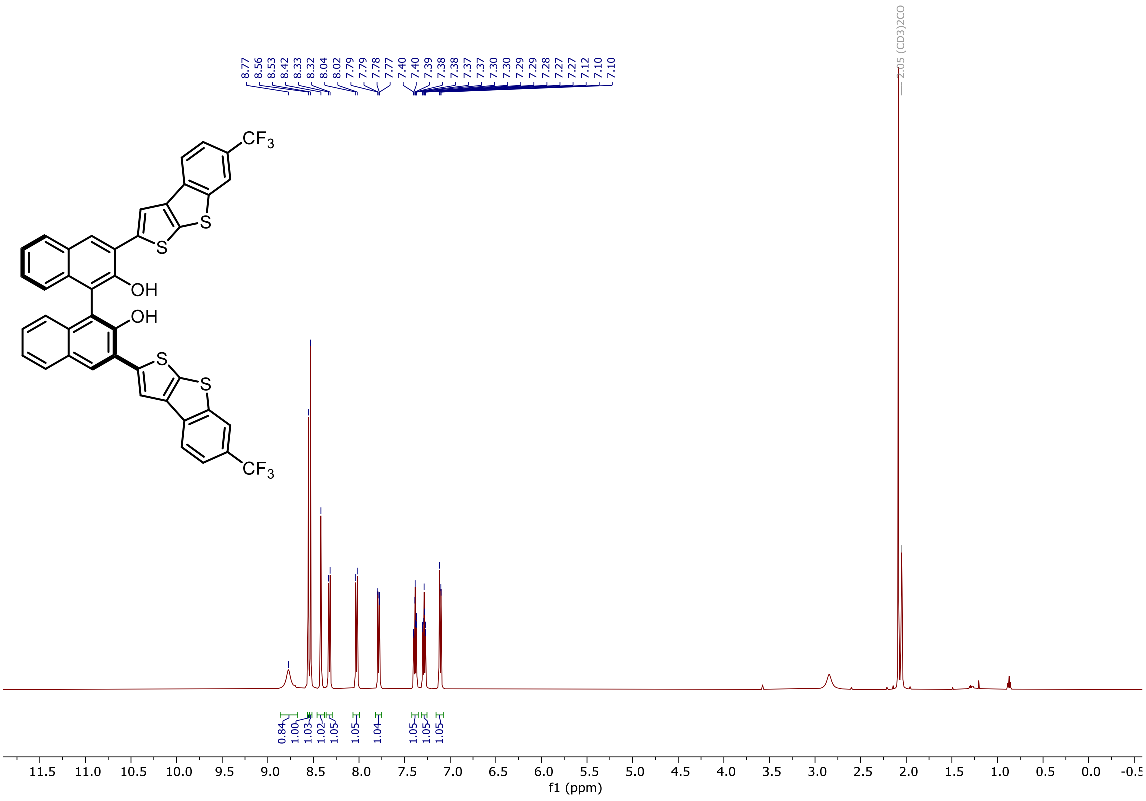

^{19}F NMR spectrum of **S14**

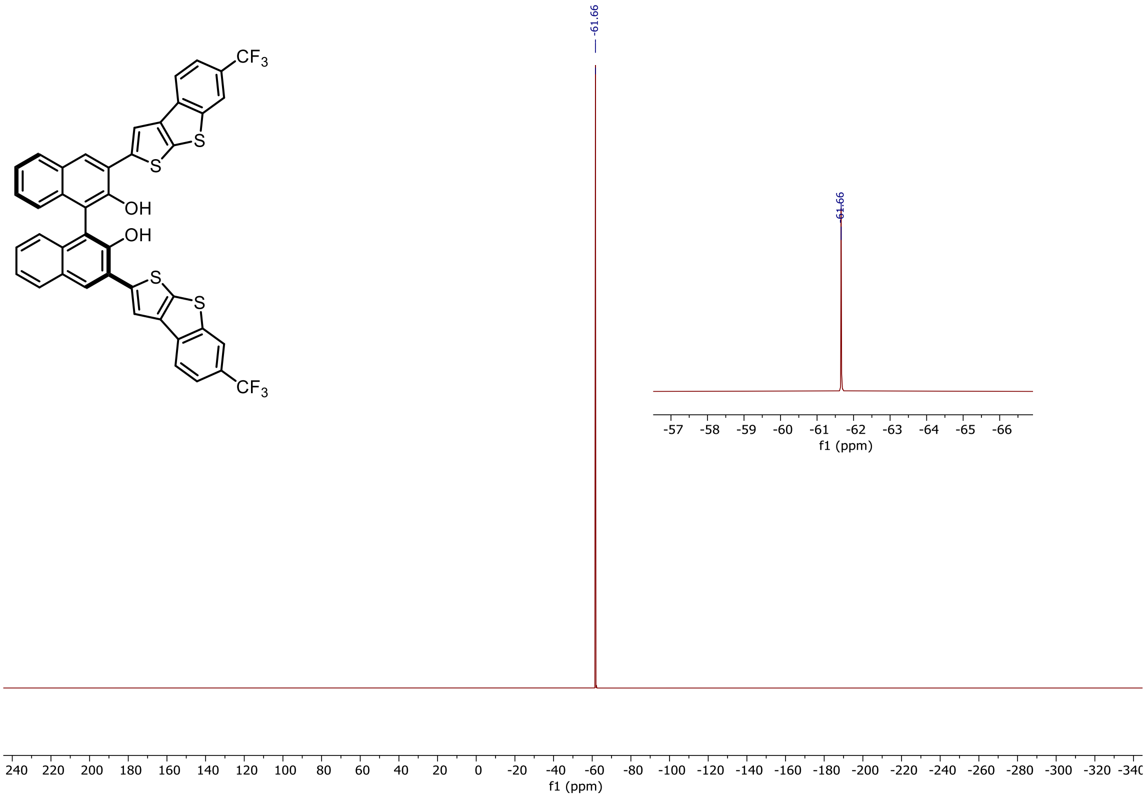

^{13}C NMR spectrum of **S14**

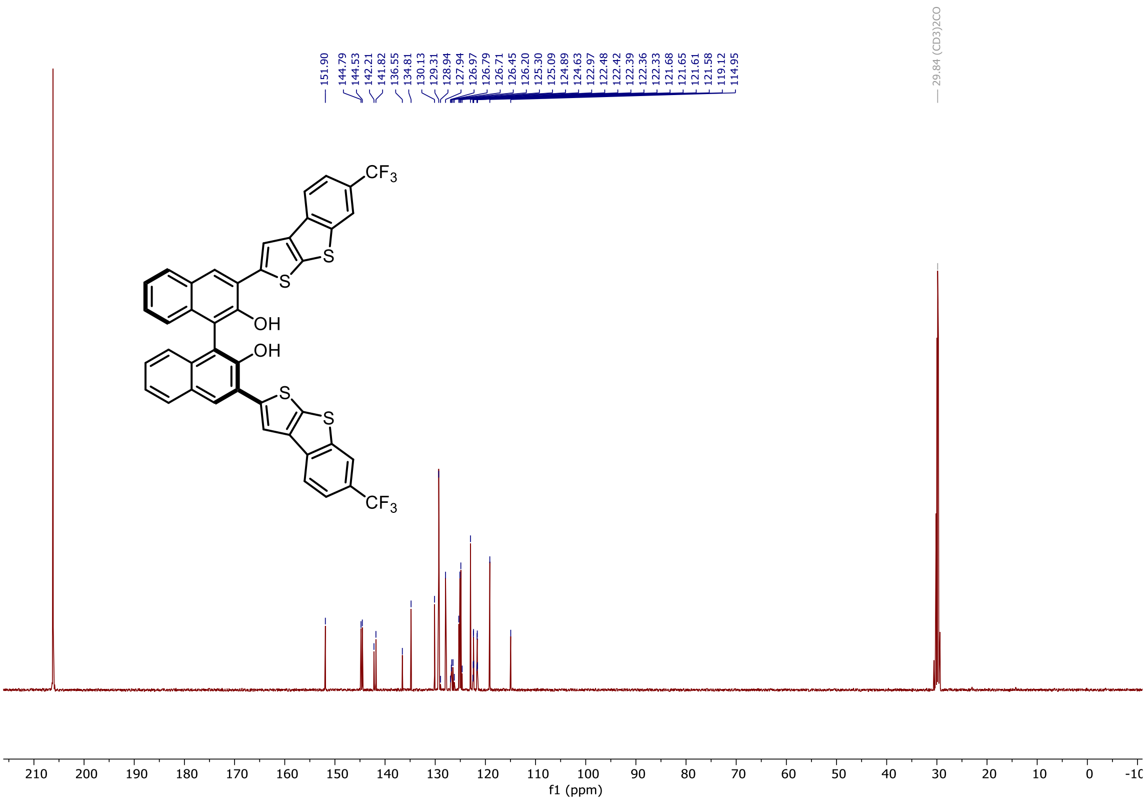

^{1}H NMR spectrum of **5a**

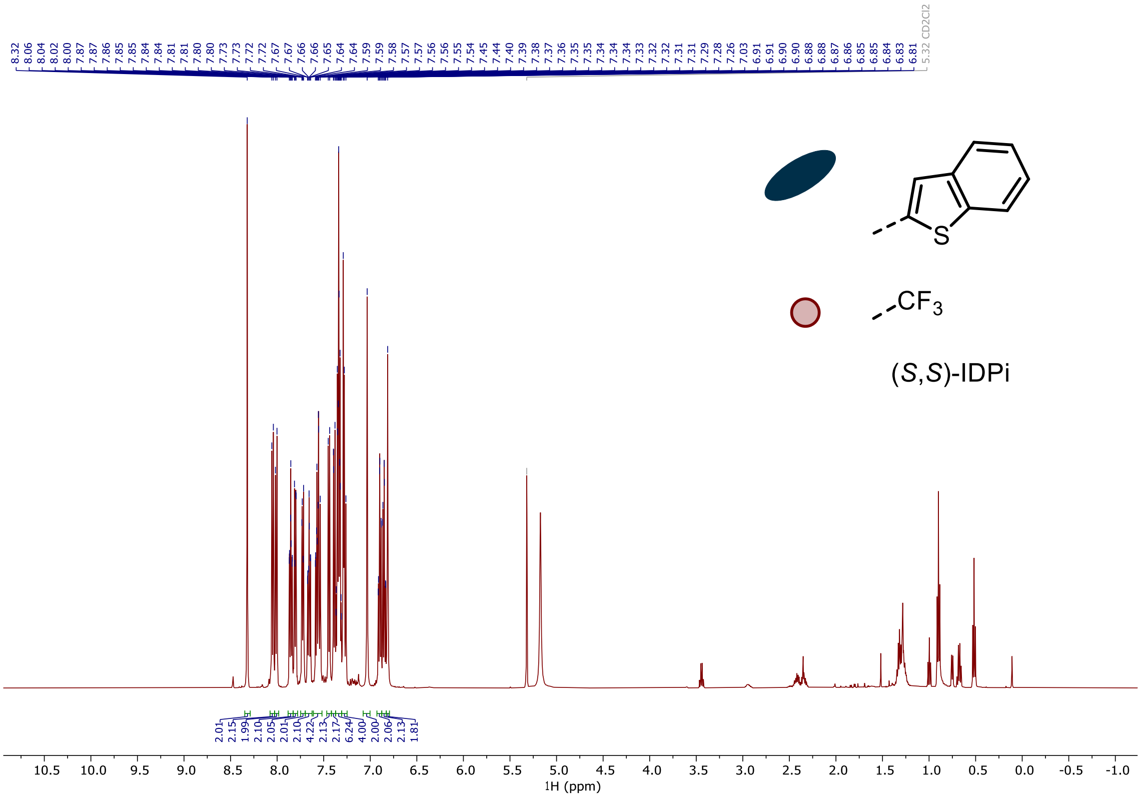

^{19}F NMR spectrum of **5a**

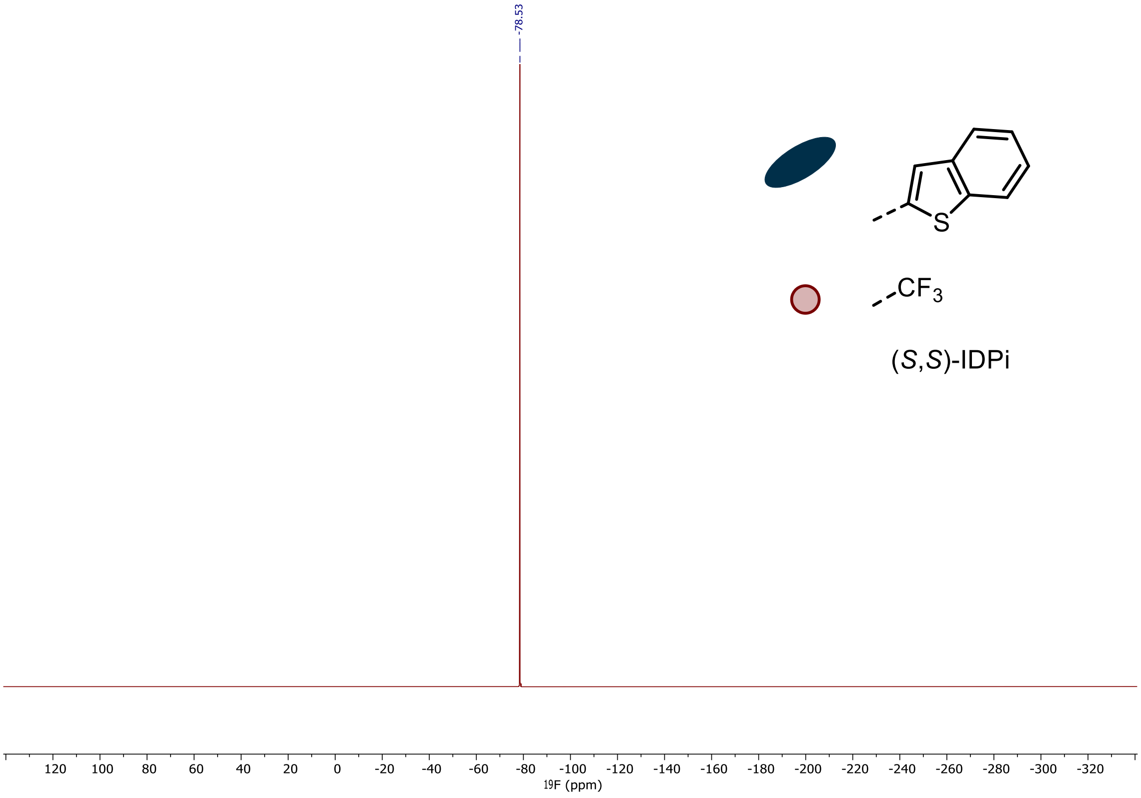

^{31}P NMR spectrum of **5a**

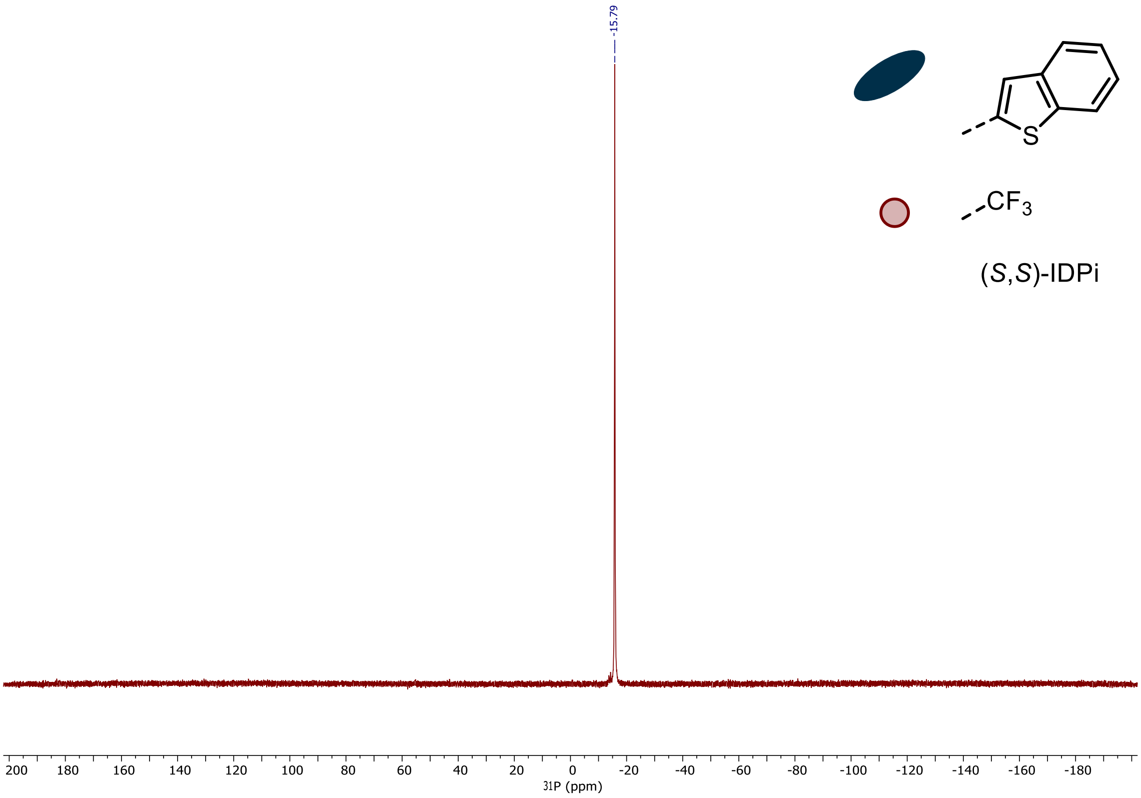

^{13}C NMR spectrum of **5a**

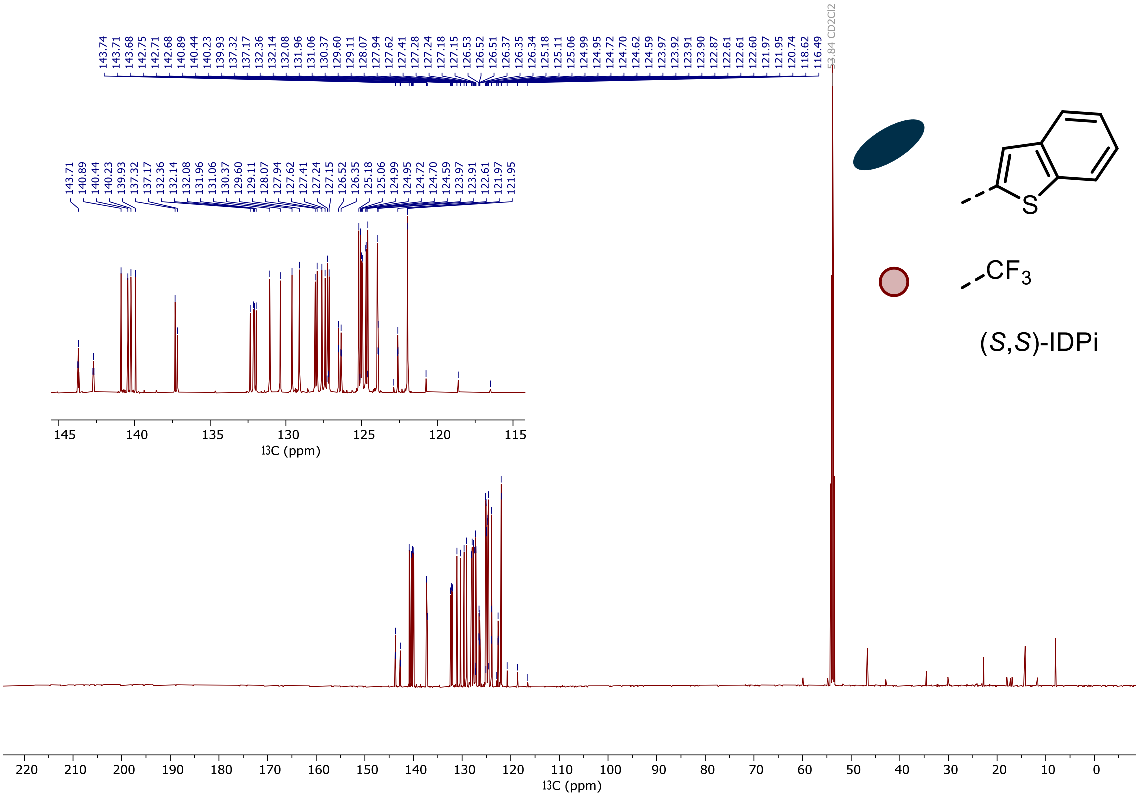

^{1}H NMR spectrum of **5b**

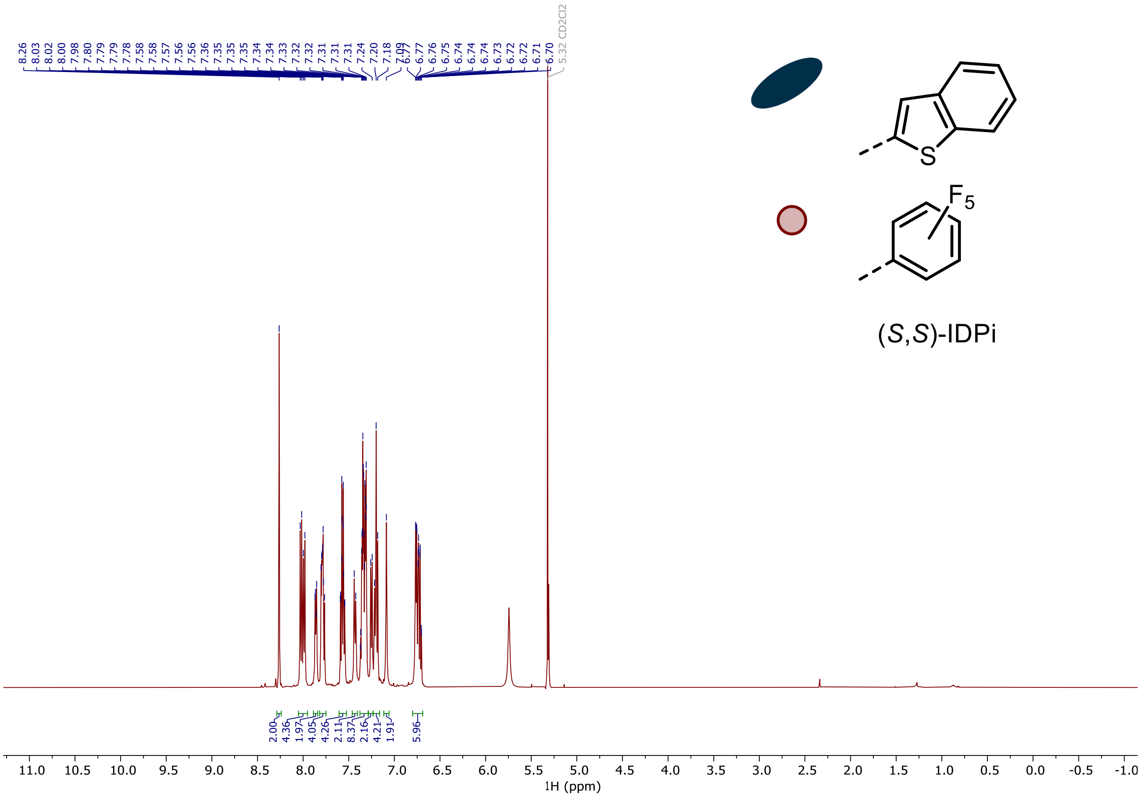

^{19}F NMR spectrum of **5b**

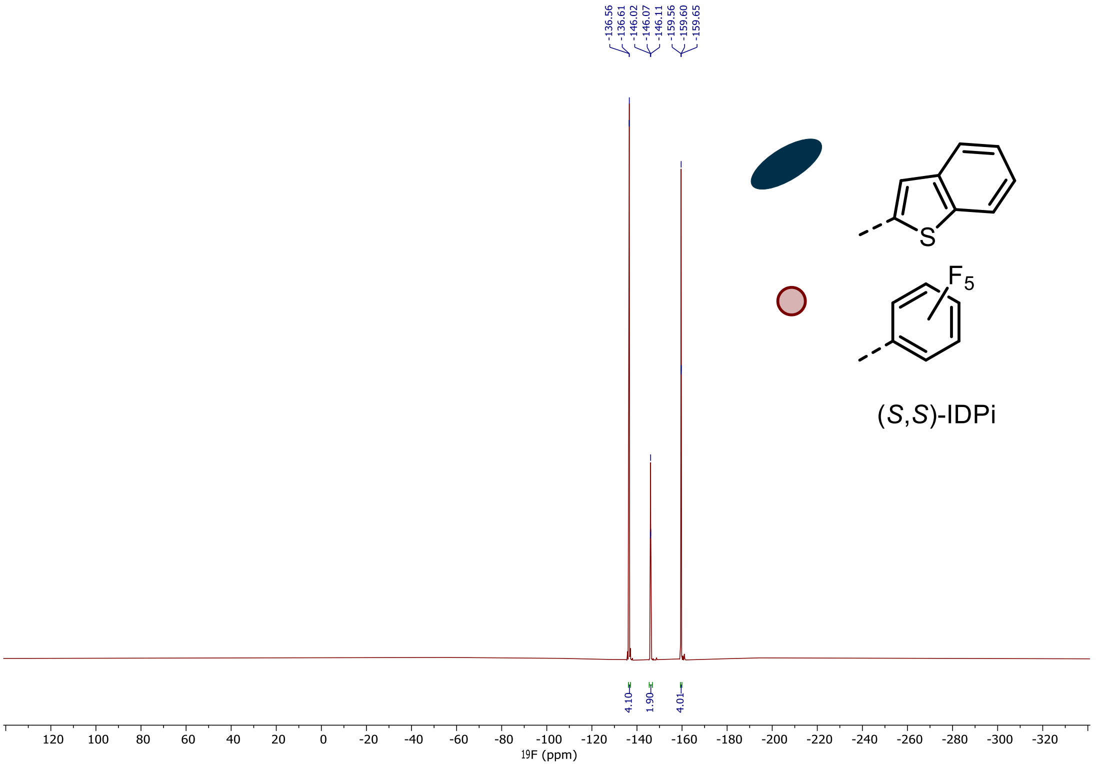

^{31}P NMR spectrum of **5b**

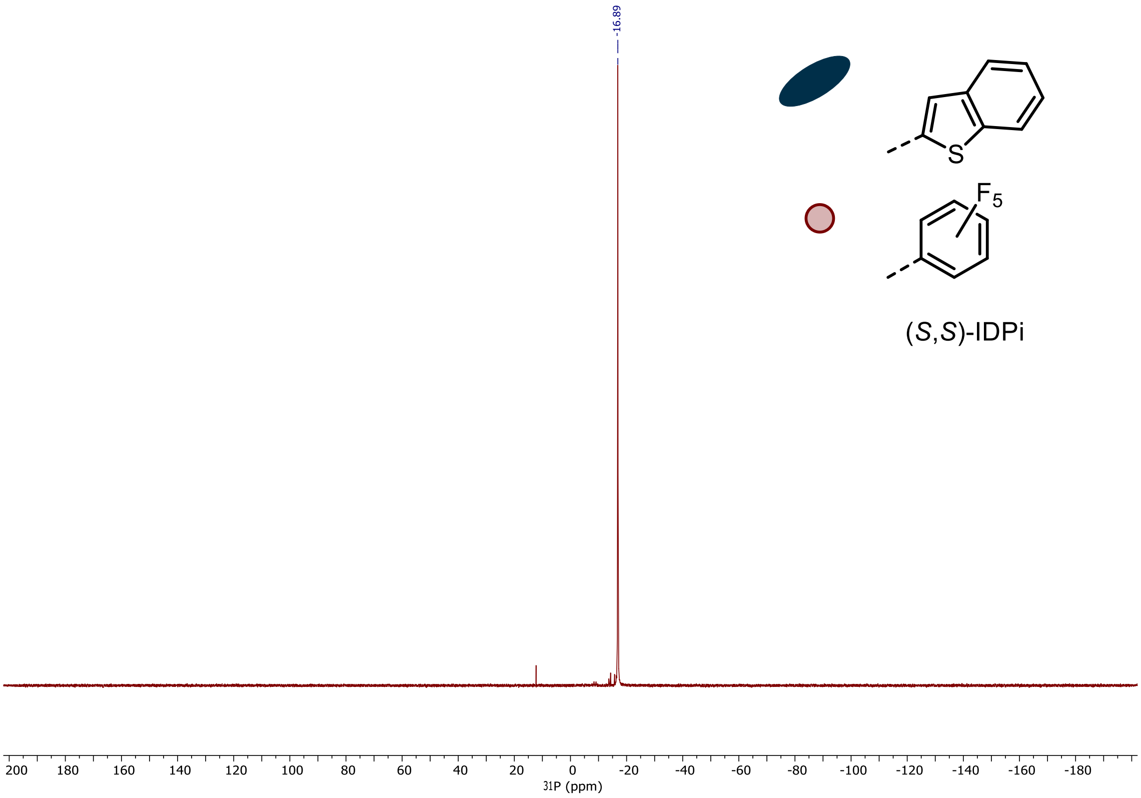

^{13}C NMR spectrum of **5b**

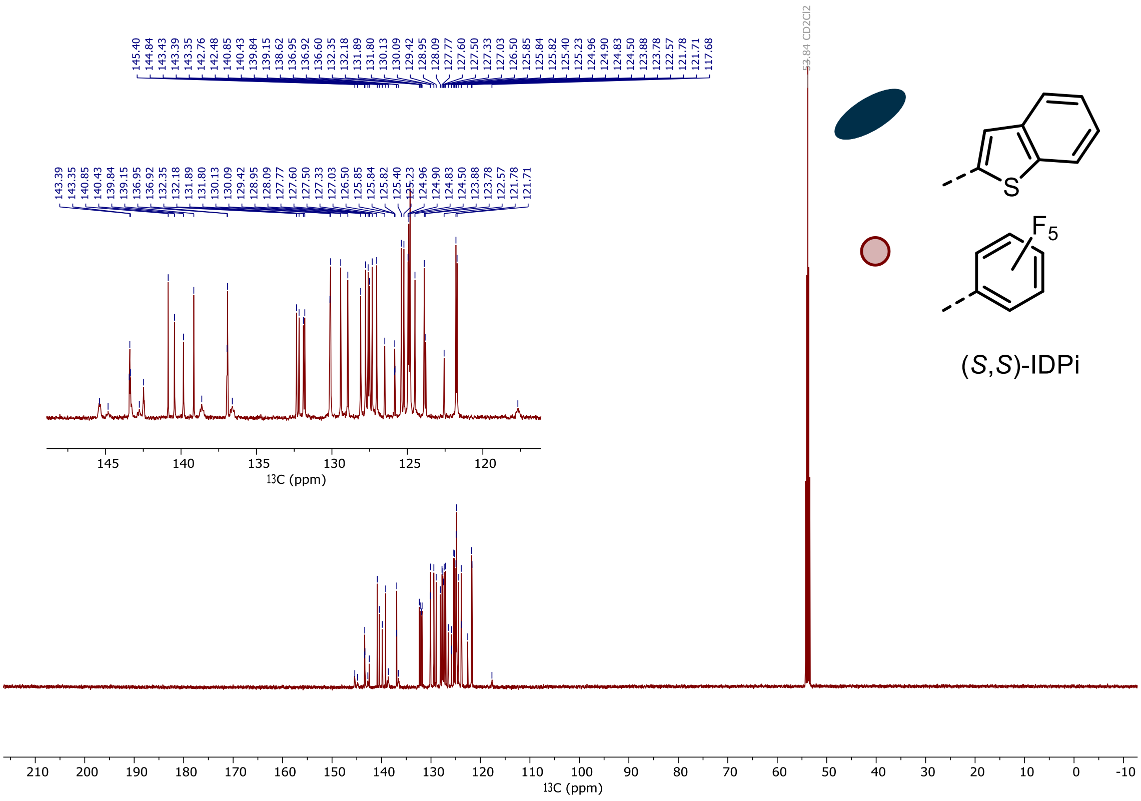

^{1}H NMR spectrum of **6b**

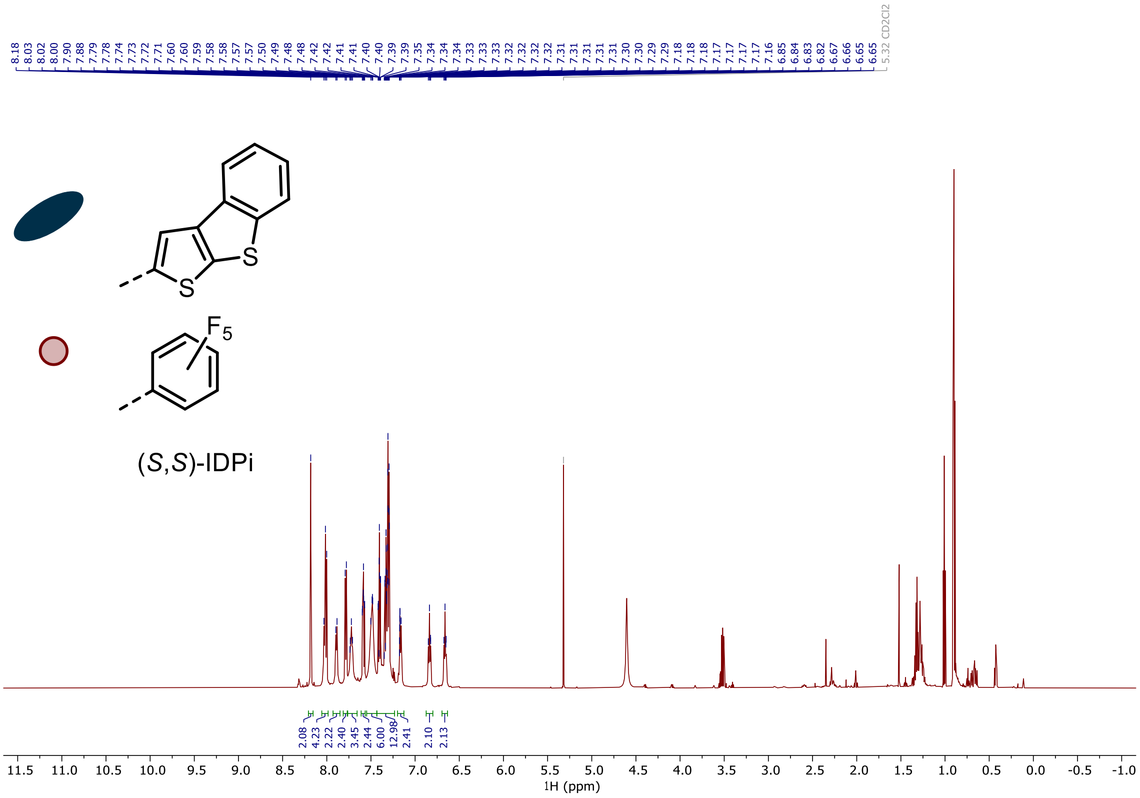

^{19}F NMR spectrum of **6b**

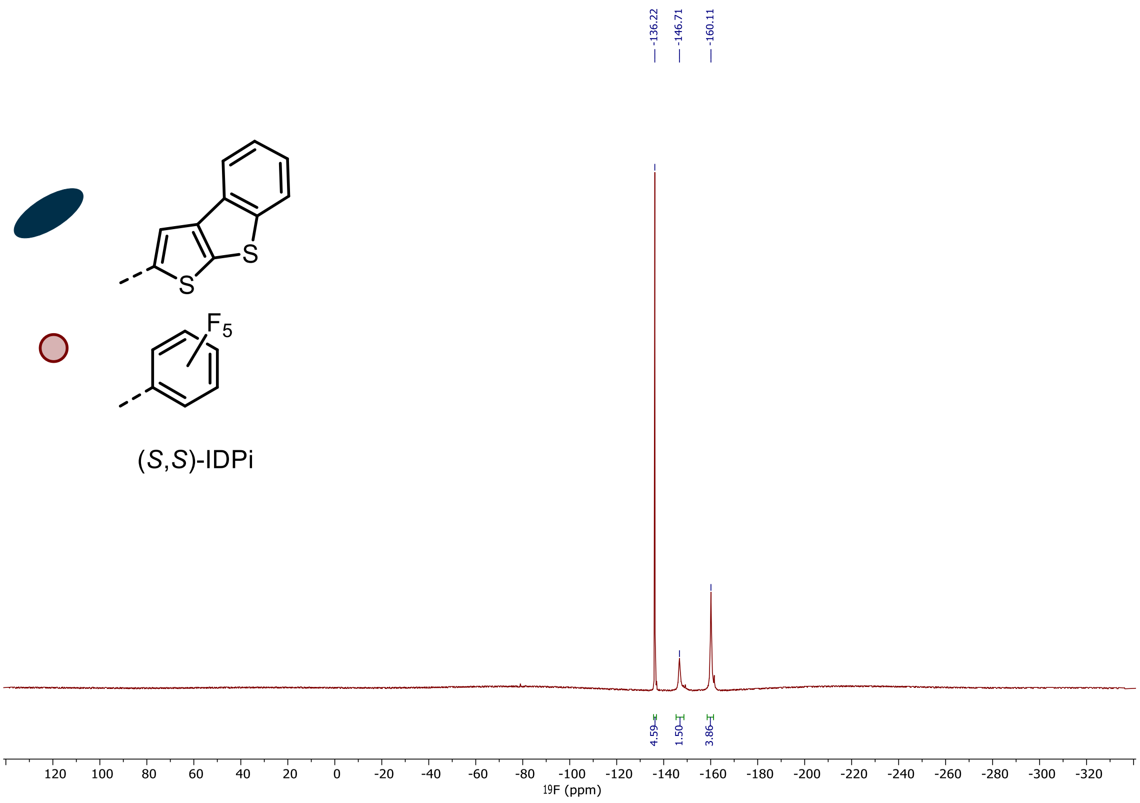

^{31}P NMR spectrum of **6b**

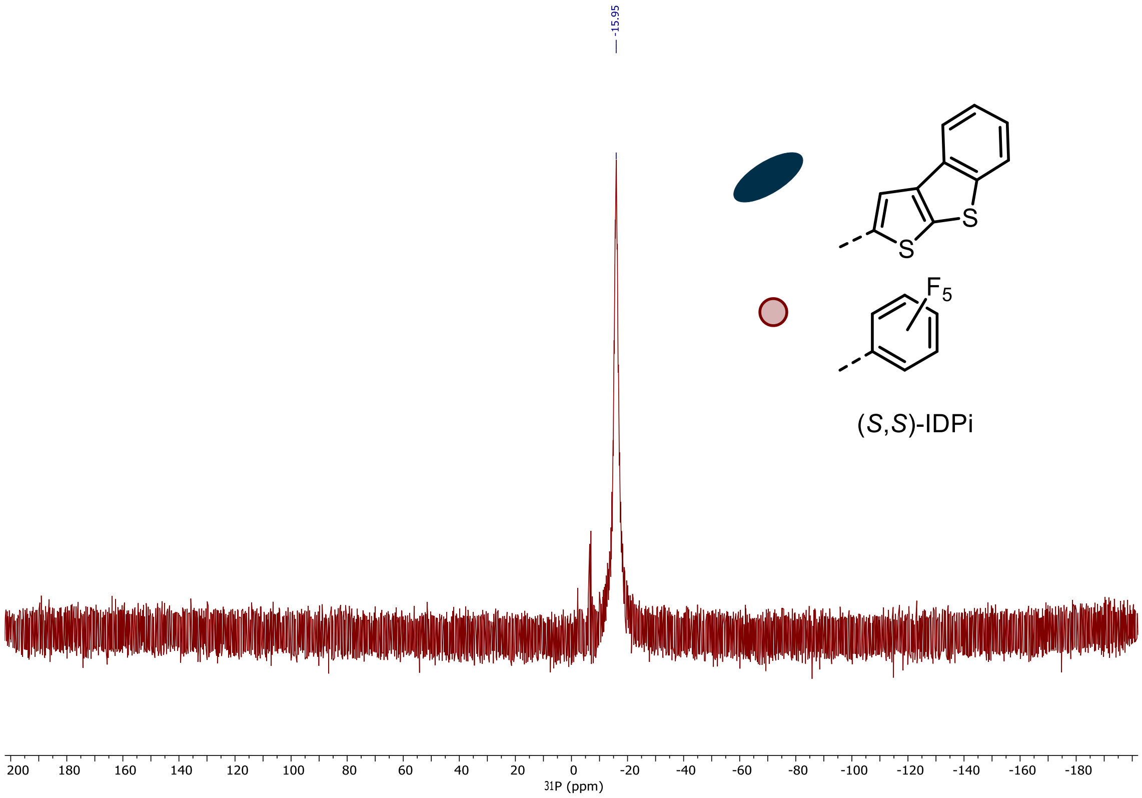

^{13}C NMR spectrum of **6b**

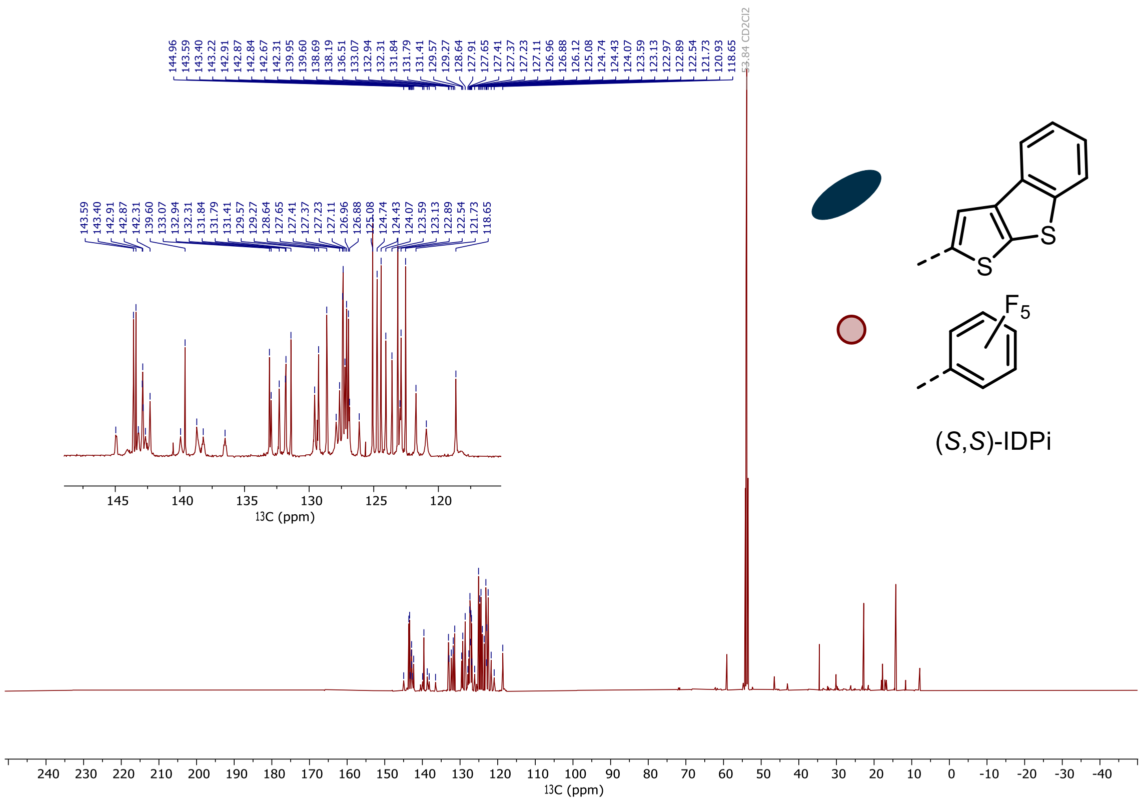

^{1}H NMR spectrum of **7b**

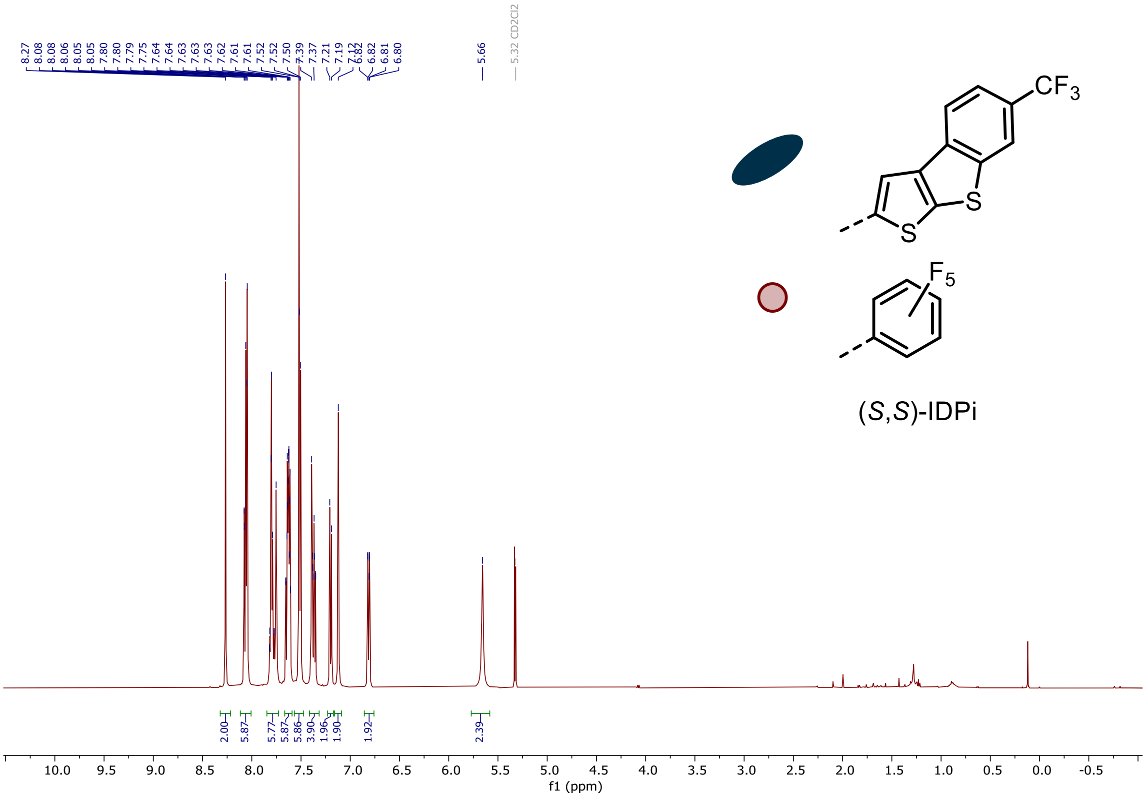

^{19}F NMR spectrum of **7b**

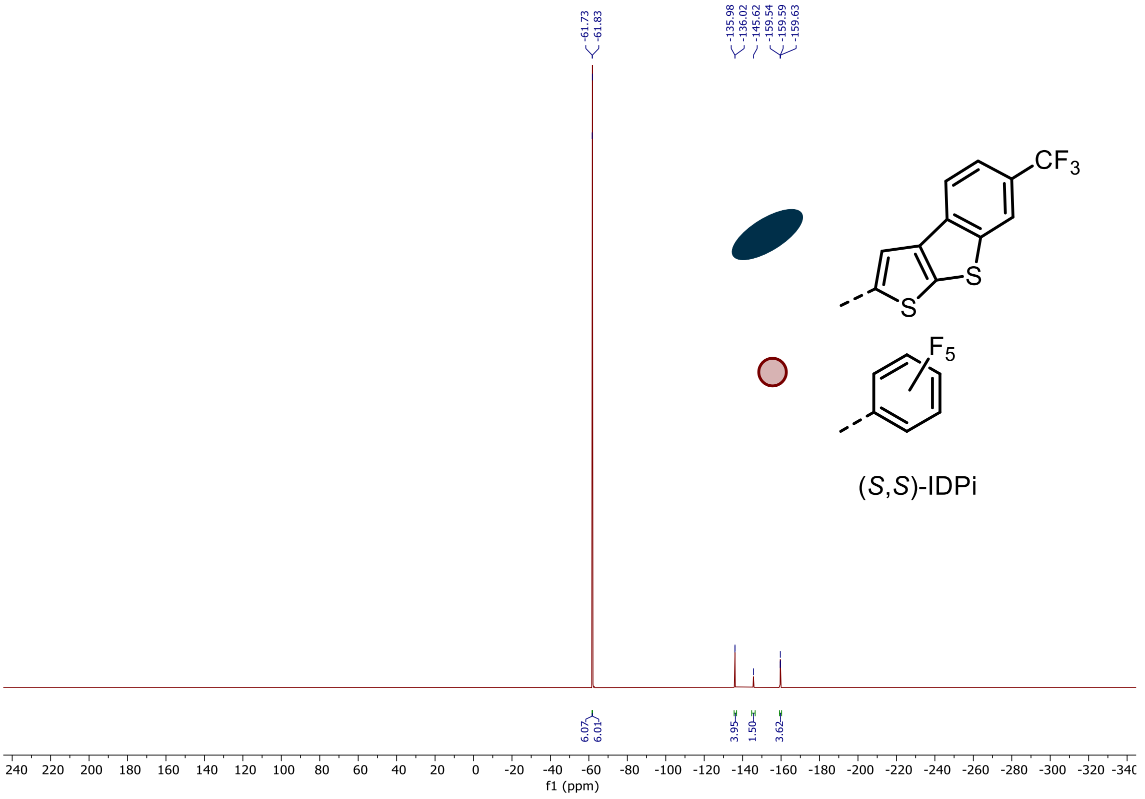

^{31}P NMR spectrum of **7b**

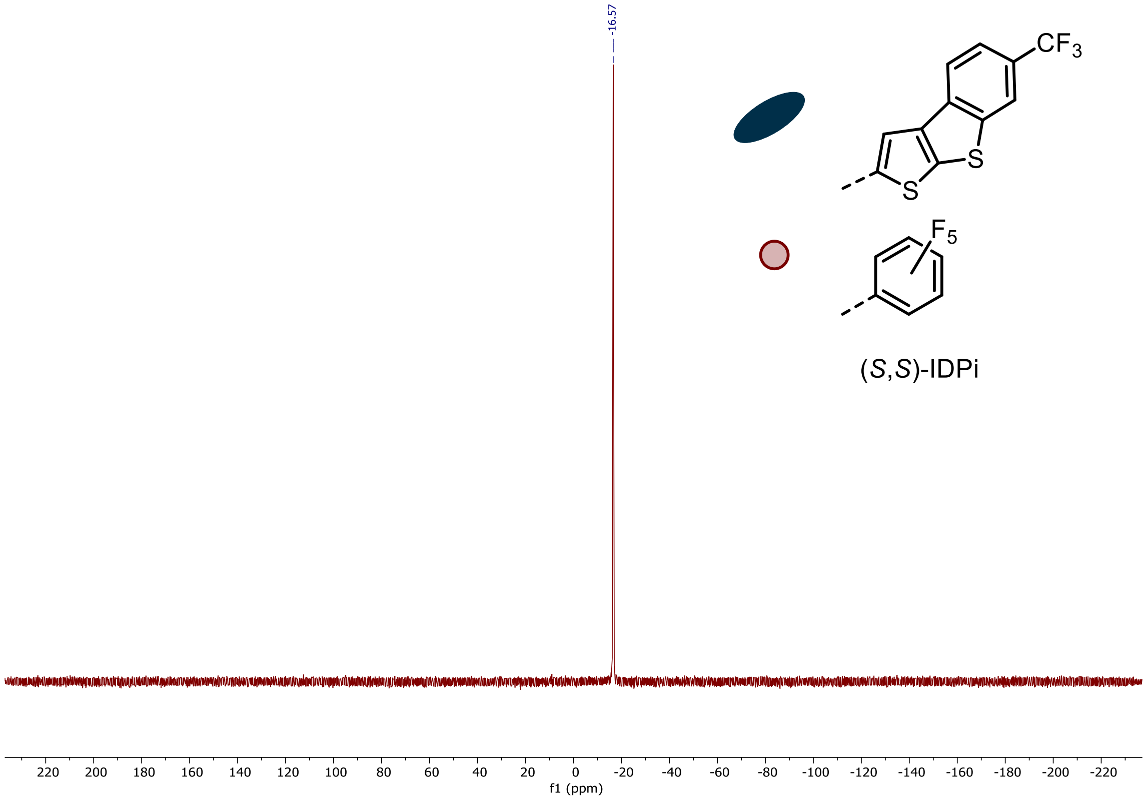

^{13}C NMR spectrum of **7b**

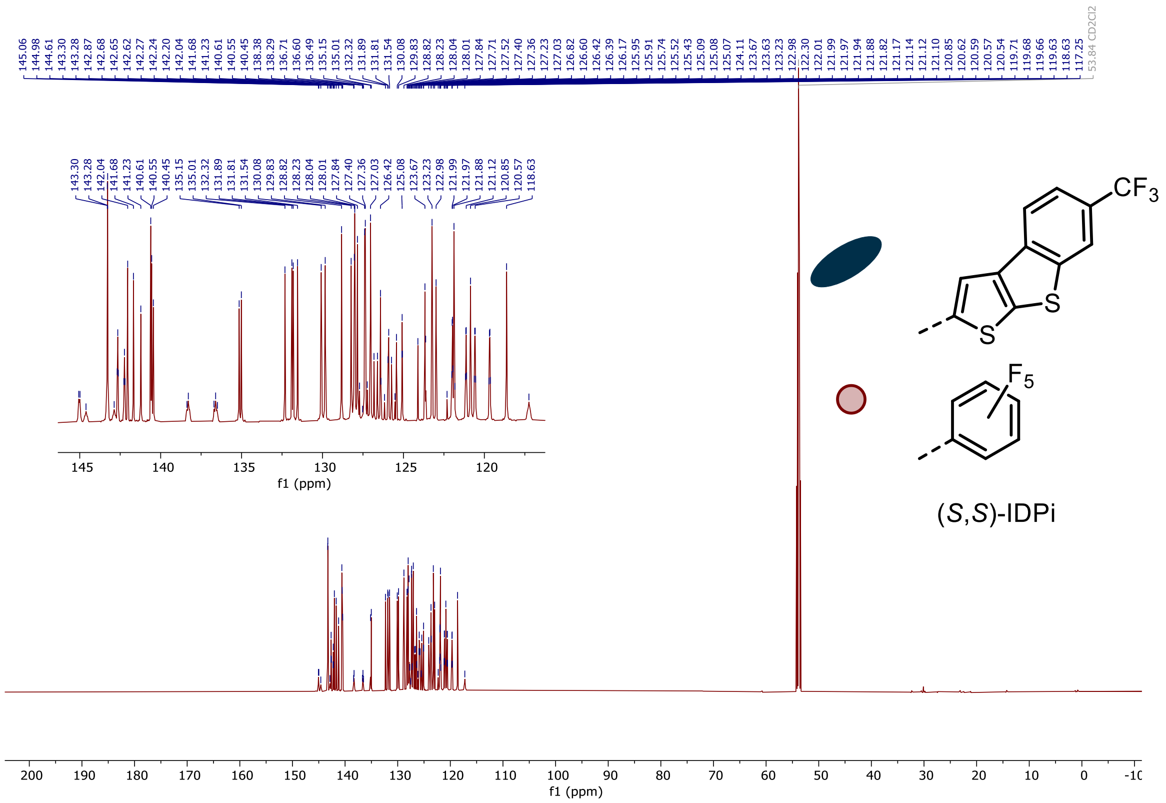

10. GC Traces

GC (30 m Cyclosil B, injection temperature: 220 °C, 90 °C iso 30 min, 1 °C/min, 100 °C iso 5 min, 8 °C/min, 220 °C iso 5 min, 0.5 bar H_2)

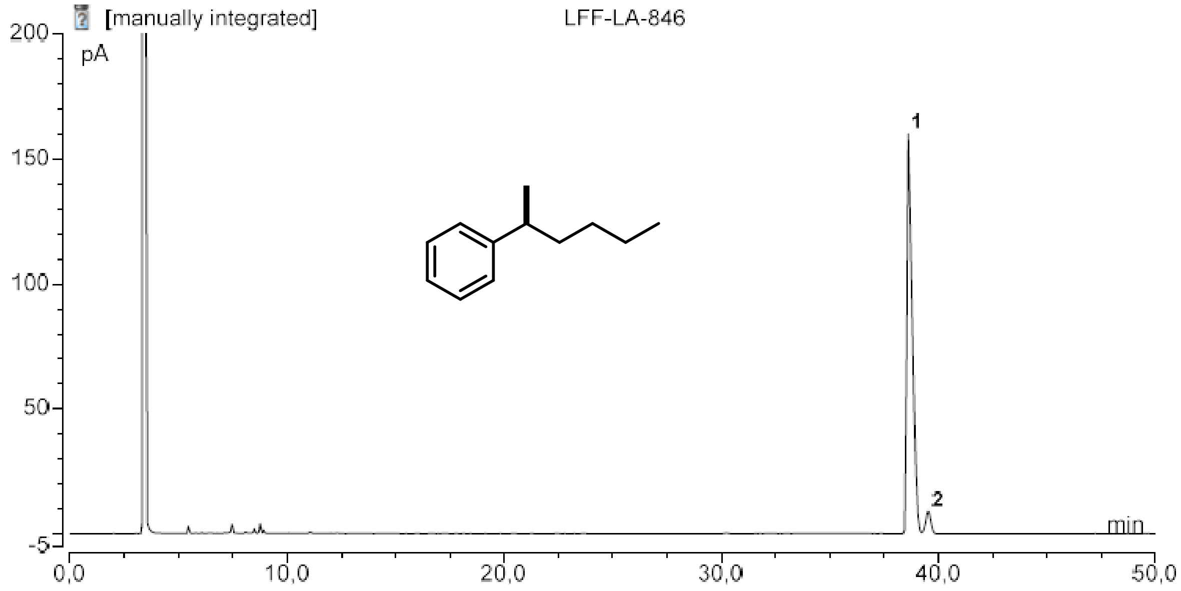

No.	Ret.Time min	Rel.Area %	Peak Name
1	38,61	95,03	Component 15
2	39,54	4,97	Component 16

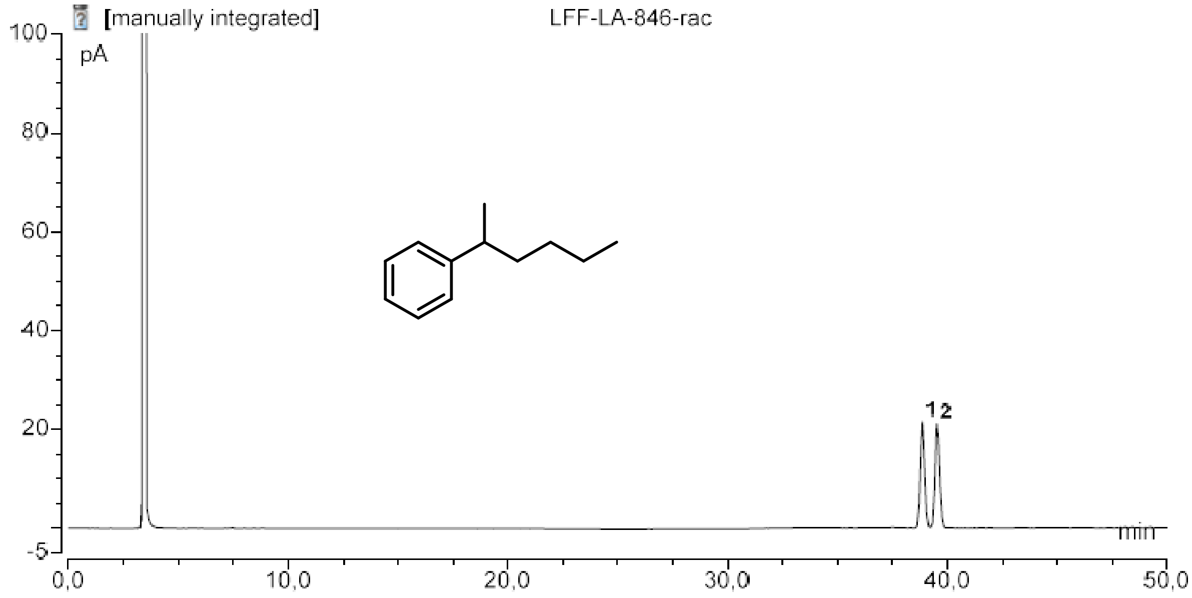

No.	Ret.Time min	Rel.Area %	Peak Name
1	38,86	49,99	Component 15
2	39,52	50,01	Component 16

GC (30 m Cyclosil B, injection temperature: 220 °C, 90 °C iso 30 min, 1 °C/min, 100 °C iso 5 min, 8 °C/min, 220 °C iso 5 min, 0.5 bar H_2)

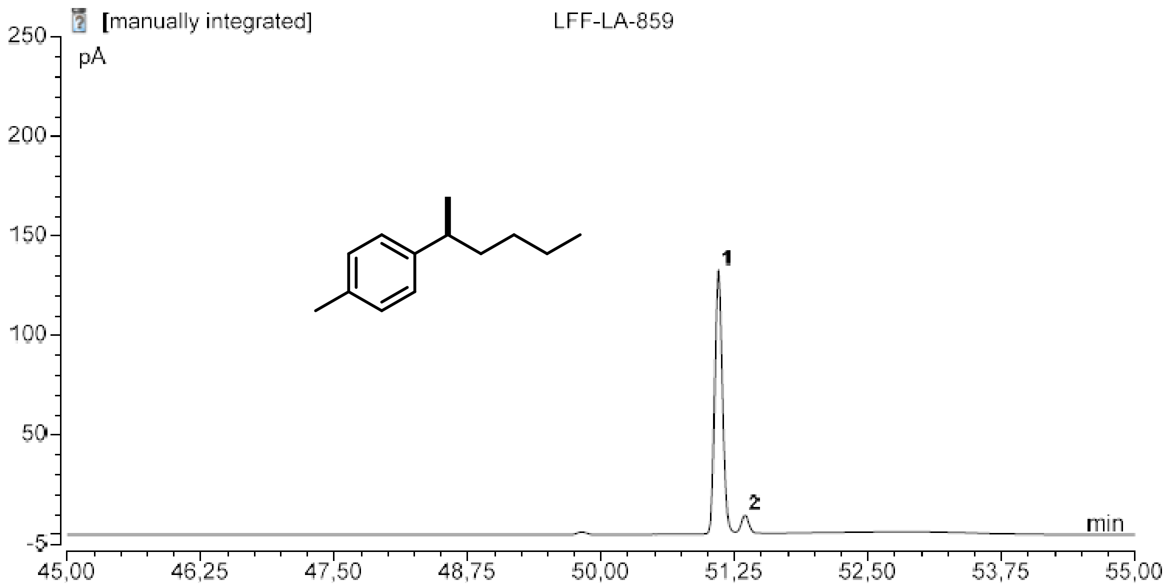

No.	Ret.Time min	Rel.Area %	Peak Name
1	51,10	93,74	Component 21
2	51,35	6,26	Component 22

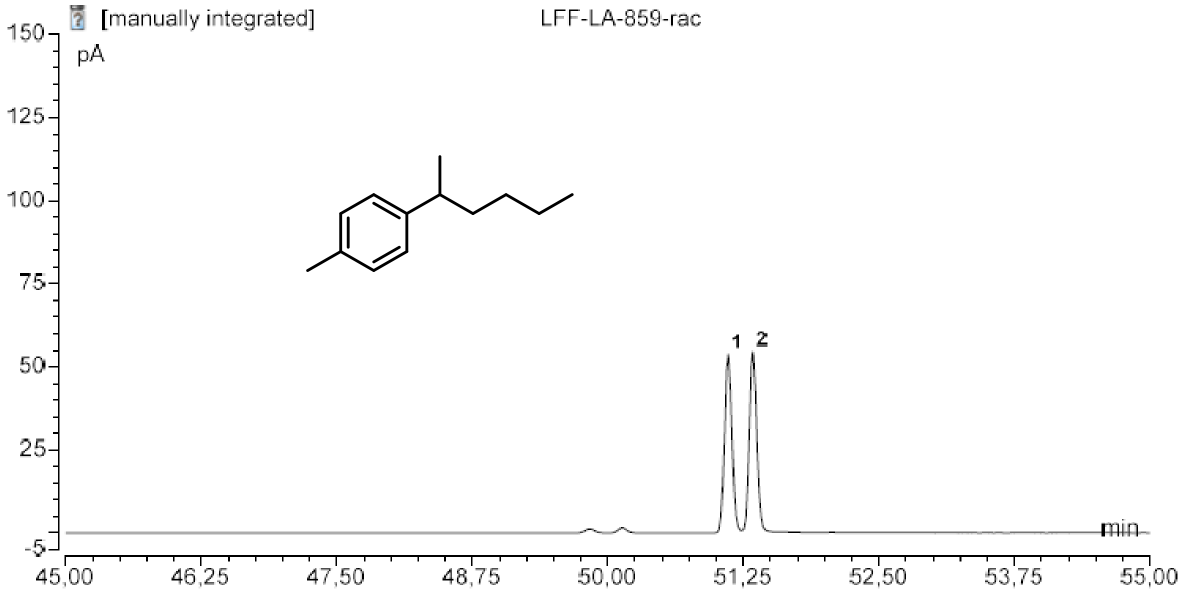

No.	Ret.Time min	Rel.Area %	Peak Name
1	51,11	49,94	Component 21
2	51,34	50,06	Component 22

GC (25 m Lipodex-G, injection temperature: 220 °C, 80 °C iso 20 min, 8 °C/min, 220 °C iso 3 min, 0.6 bar H_2)

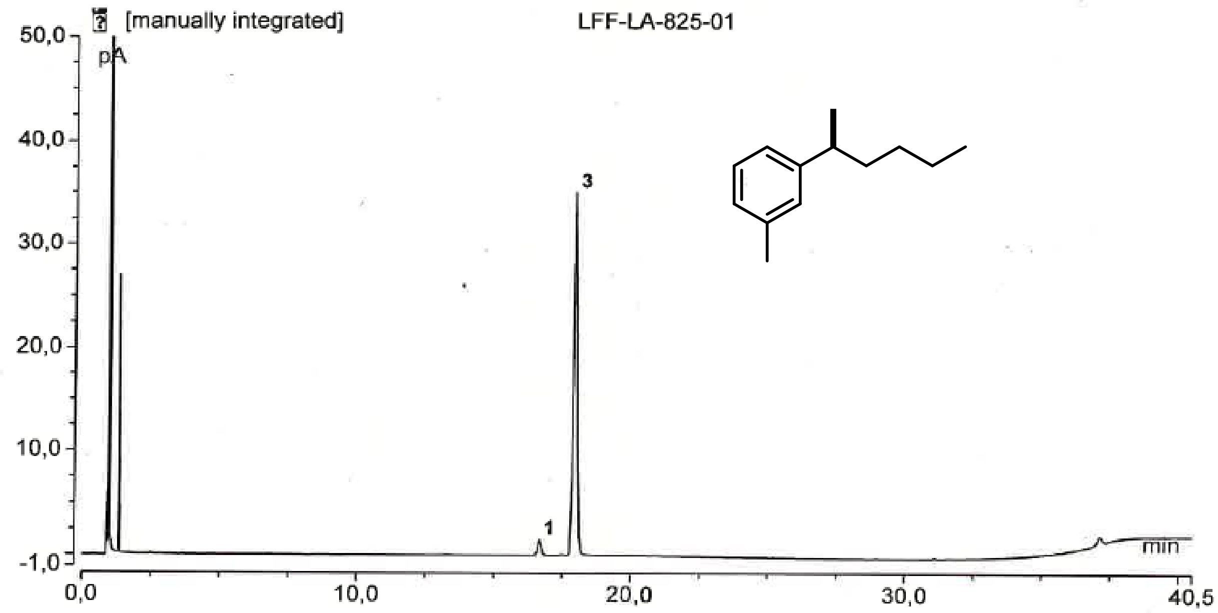

No.	Ret.Time min	Rel.Area %	Peak Name
1	16,67	3,75 .	
3	17,92	96,25 .	

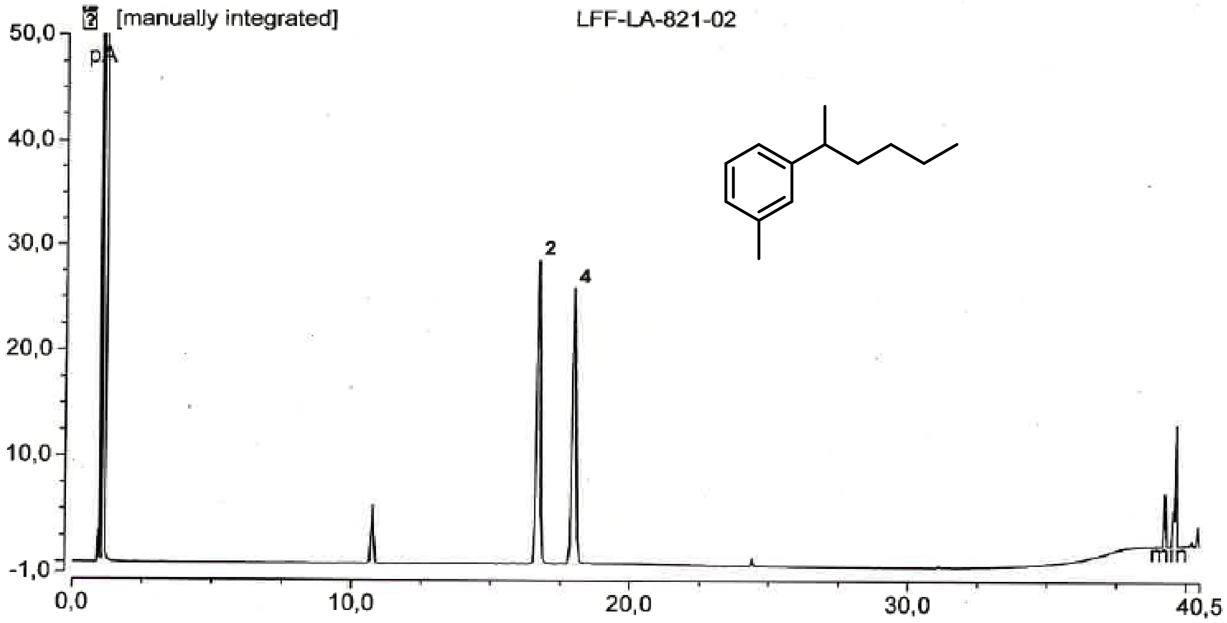

No.	Ret.Time min	Rel.Area %	Peak Name
2	16,67	50,06 .	
4	17,94	49,94 .	

GC (25 m Lipodex-G, injection temperature: 220 °C, 75 °C iso 45 min, 8 °C/min, 220 °C iso 3 min, 0.6 bar H_2)

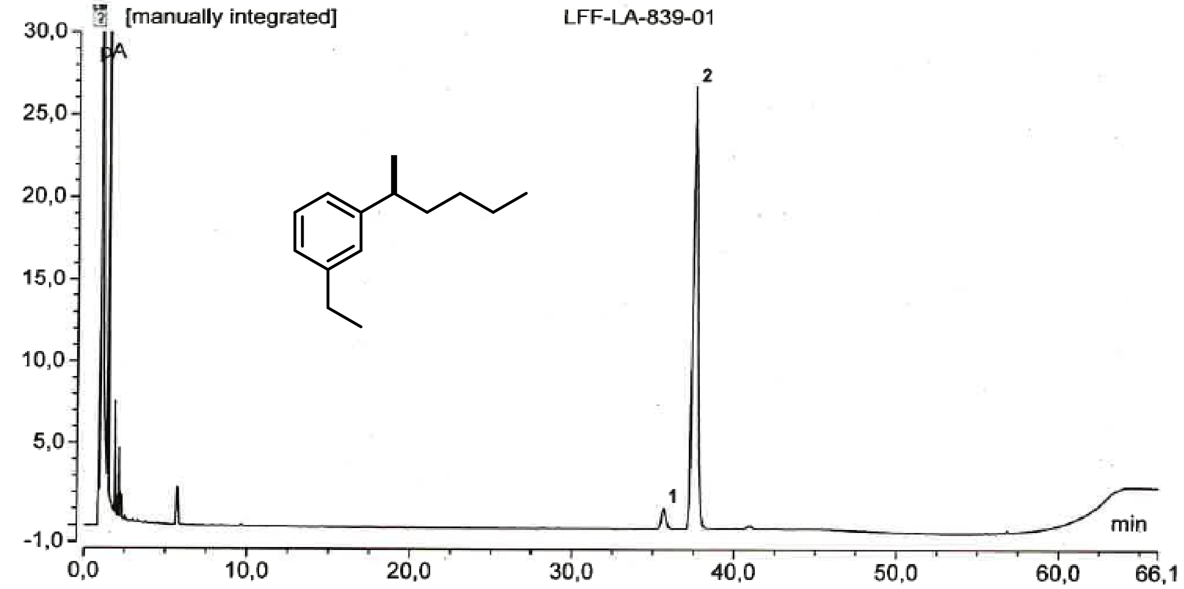

No.	Ret.Time min	Rel.Area %	Peak Name
1	35,64	4,22	.
2	37,47	95,78	.

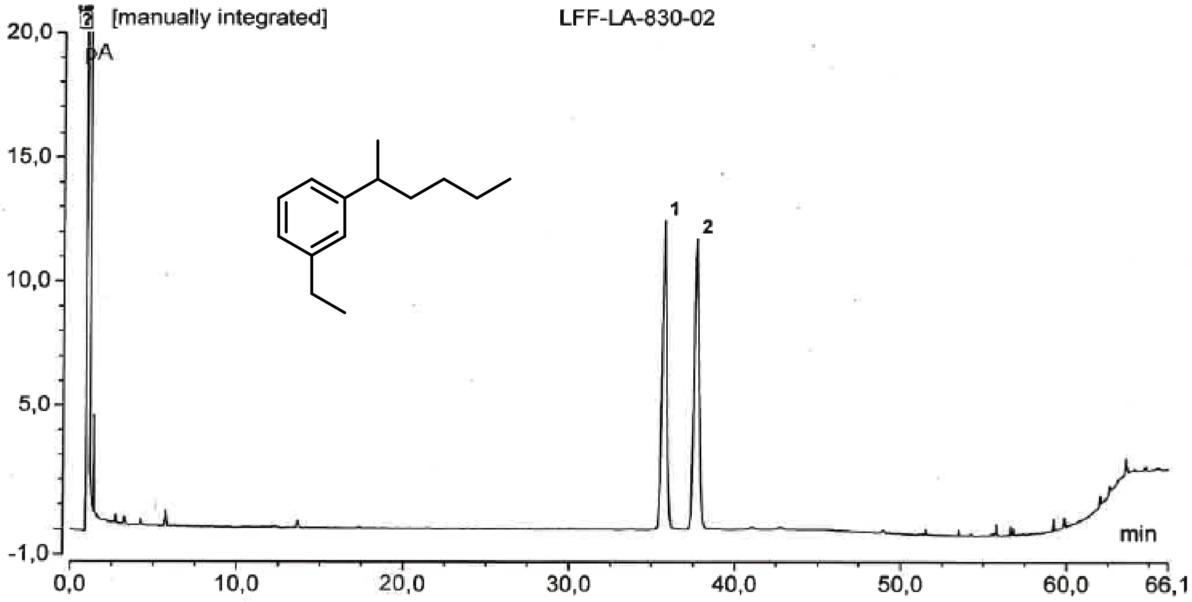

No.	Ret.Time min	Rel.Area %	Peak Name
1	35,69	49,97	.
2	37,63	50,03	.

GC (25 m Lipodex-G, injection temperature: 220 °C, 70 °C iso 53 min, 8 °C/min, 220 °C iso 3 min, 0.6 bar H_2)

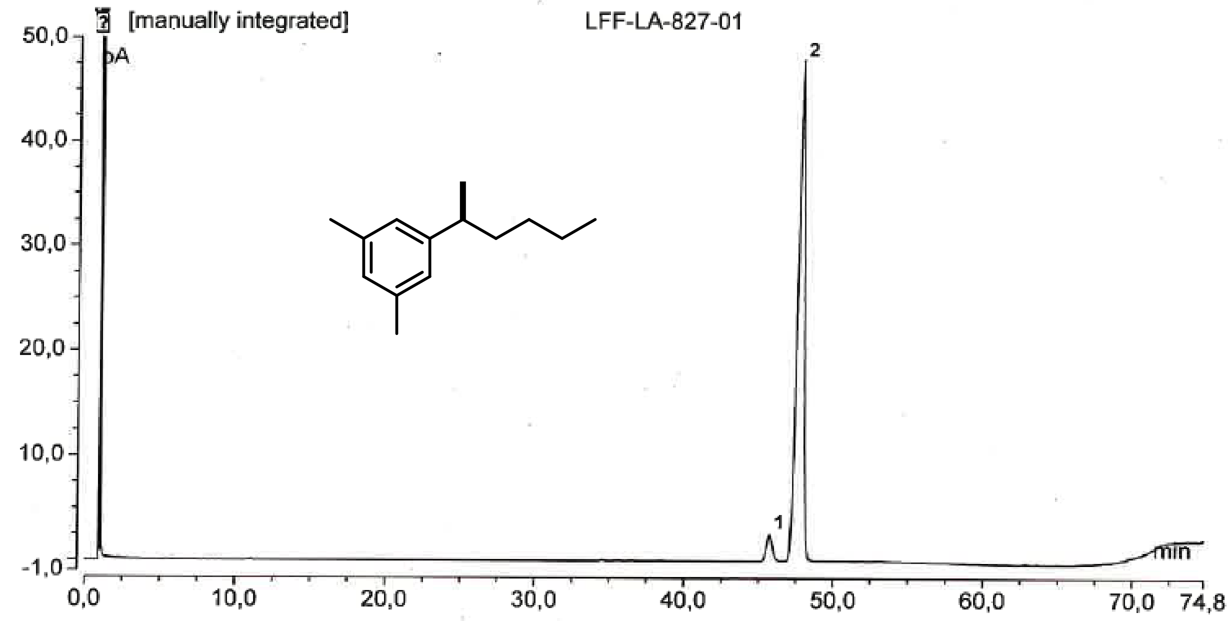

No.	Ret.Time min	Rel.Area %	Peak Name
1	45,71	4,53	.
2	47,80	95,47	.

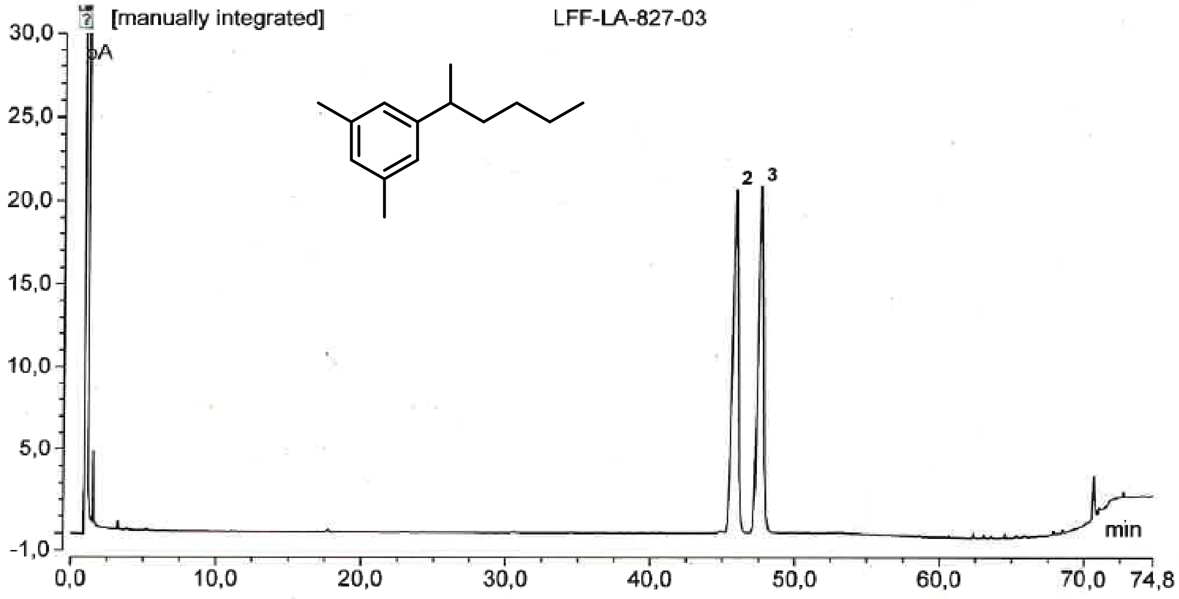

No.	Ret.Time min	Rel.Area %	Peak Name
2	45,85	49,94	.
3	47,58	50,06	.

GC (30 m Cyclosil B, injection temperature: 220 °C, 90 °C iso 30 min, 1 °C/min, 100 °C iso 5 min, 8 °C/min, 2 20 °C iso 5 min, 0.5 bar H_2)

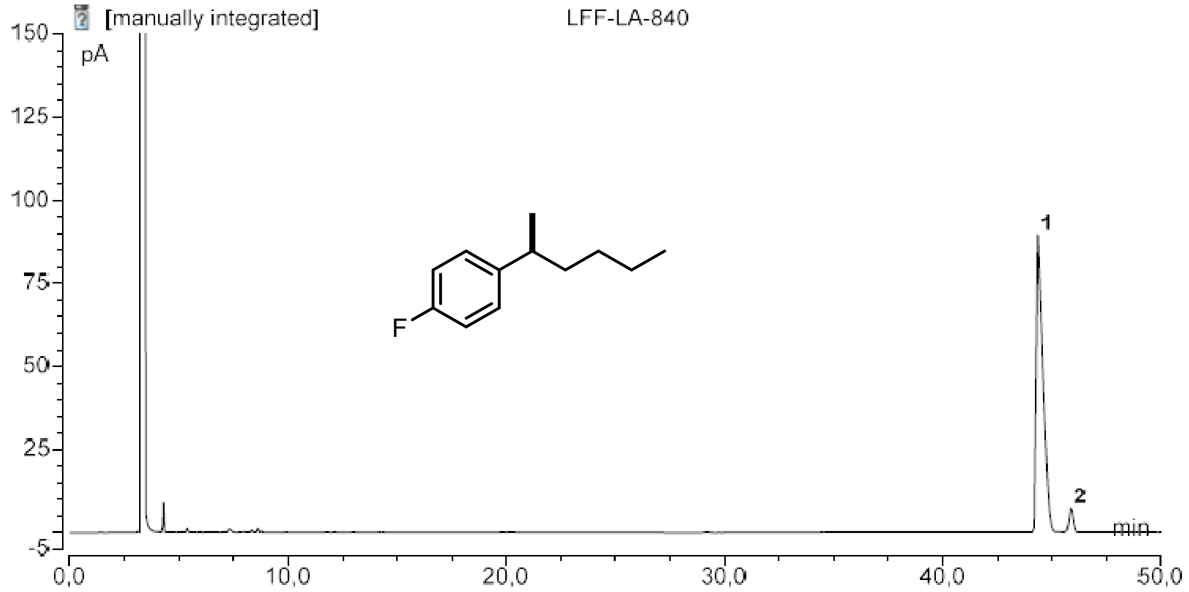

No.	Ret.Time min	Rel.Area %	Peak Name
1	44,36	95,33	Component 19
2	45,88	4,67	Component 20

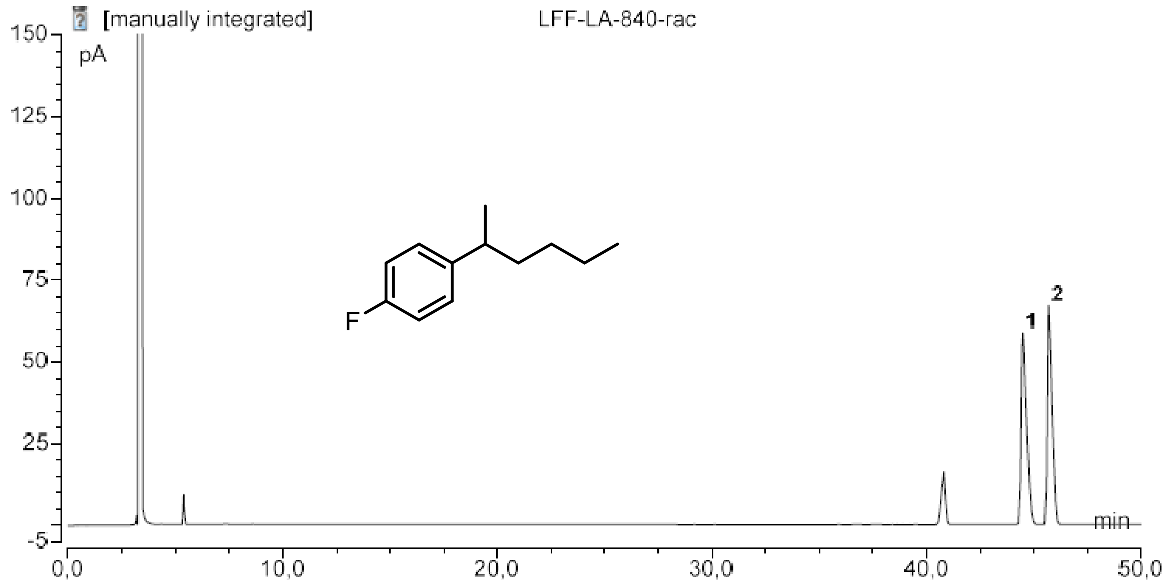

No.	Ret.Time min	Rel.Area %	Peak Name
1	44,49	50,00	Component 19
2	45,69	50,00	Component 20

GC (25 m Lipodex-G, injection temperature: 220 °C, 85 °C iso 55 min, 8 °C/min, 220 °C iso 3 min, 0.6 bar H_2)

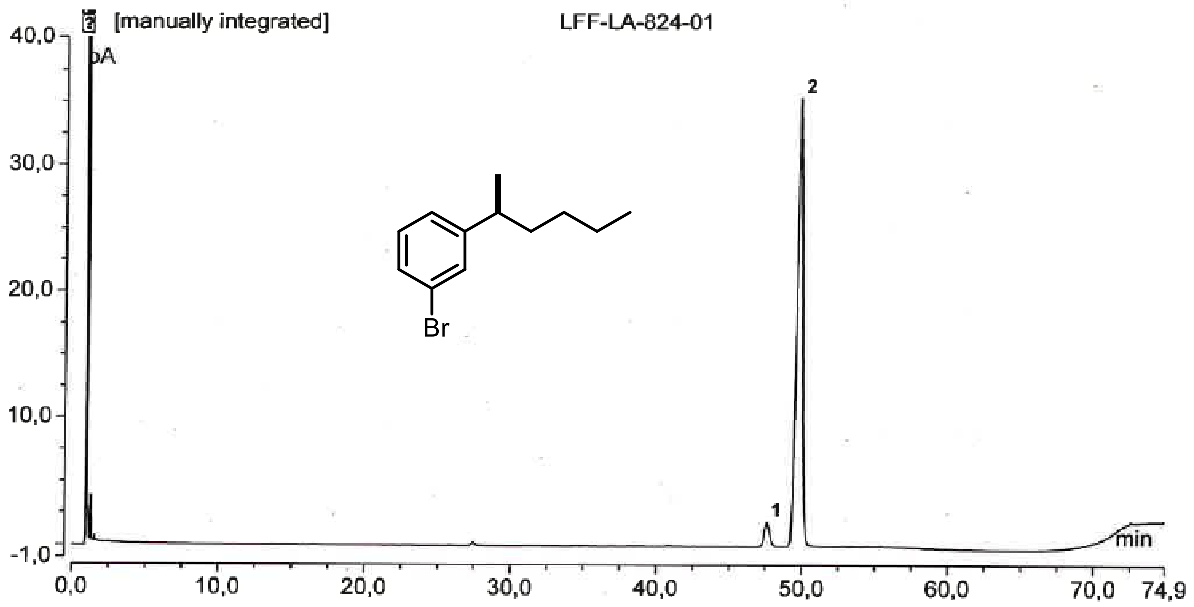

No.	Ret.Time min	Rel.Area %	Peak Name
1	47,59	4,97	.
2	49,76	95,03	.

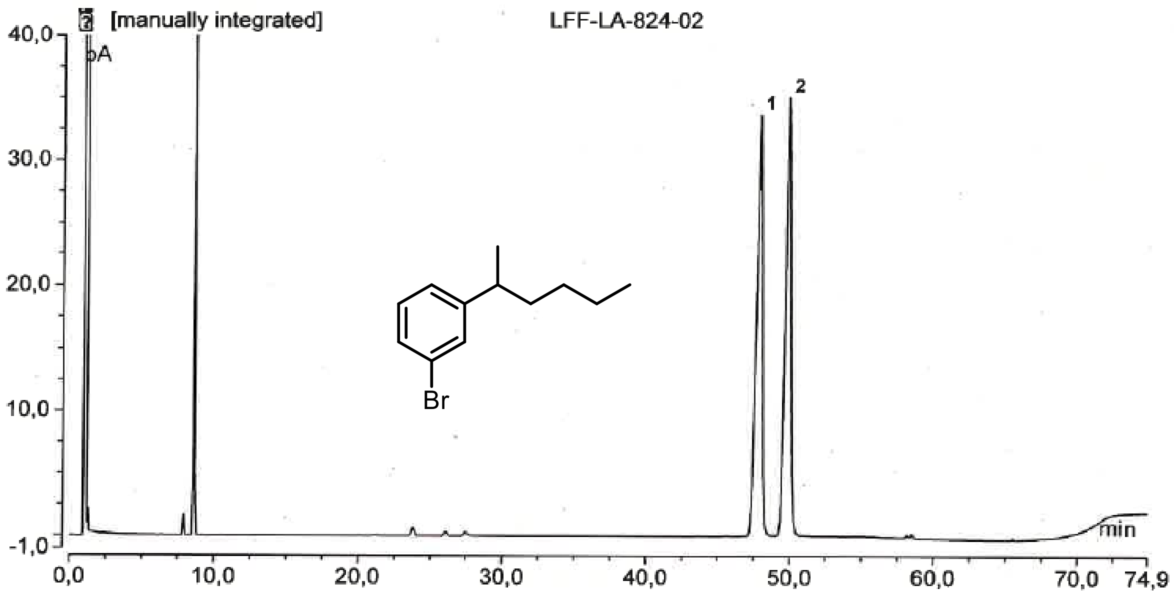

No.	Ret.Time min	Rel.Area %	Peak Name
1	47,85	49,91	.
2	49,82	50,09	.

GC (25 m Lipodex-G, injection temperature: 220 °C, 90 °C iso 35 min, 8 °C/min, 220 °C iso 3 min, 0.6 bar H_2)

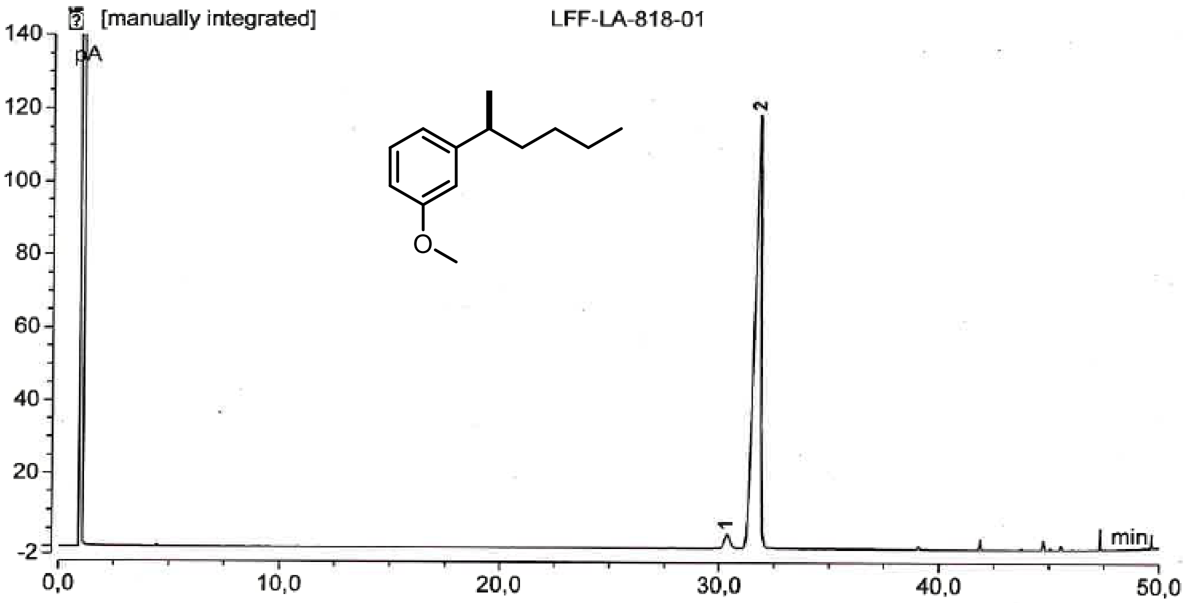

No.	Ret.Time min	Rel.Area %	Peak Name
1	30,35	3,00	.
2	31,76	97,00	.

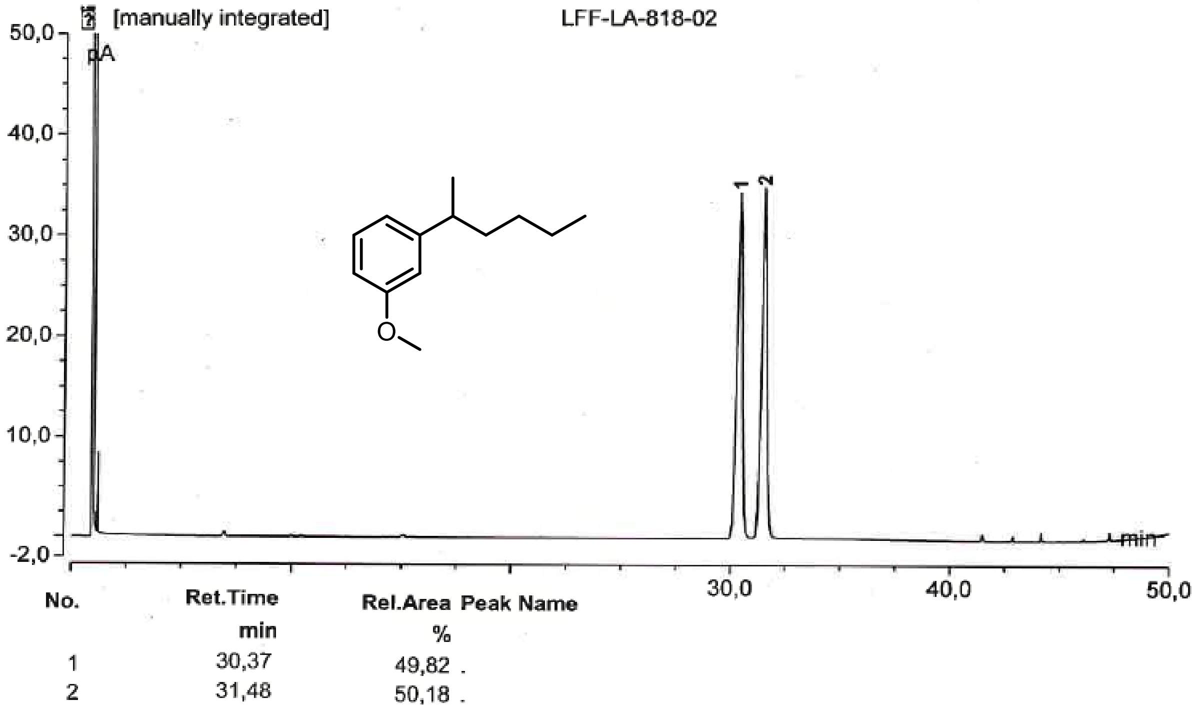

No.	Ret.Time min	Rel.Area %	Peak Name
1	30,37	49,82	.
2	31,48	50,18	.

GC (24 m Cyclodextrin-H, injection temperature: 220 °C, 100 °C iso 112 min, 8 °C/min, 180 °C iso 3 min, 0.5 bar H_2)

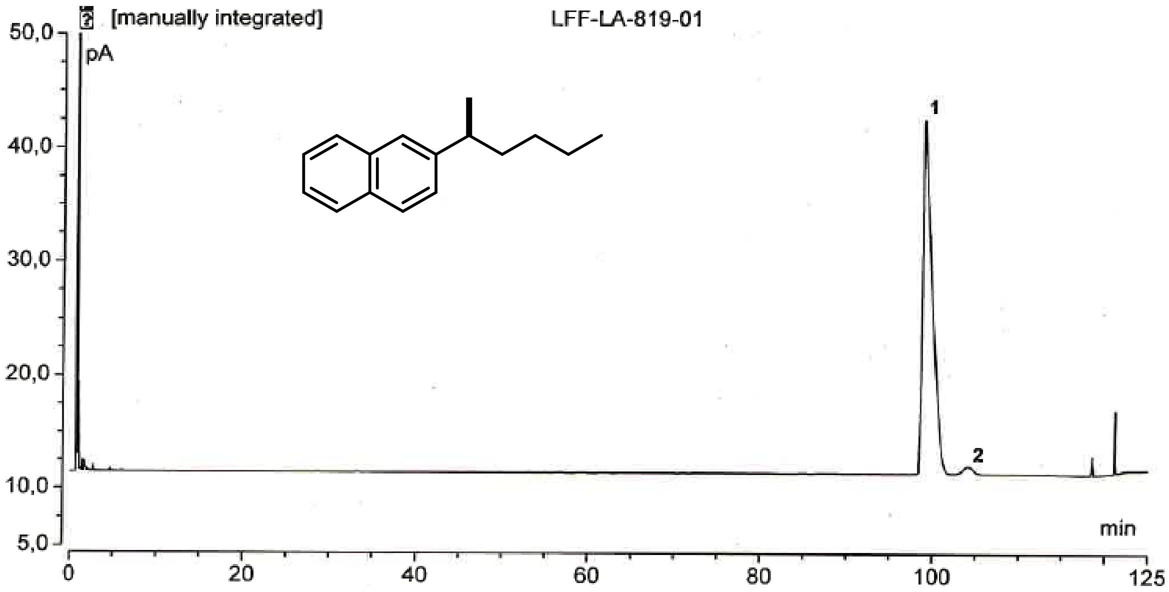

No.	Ret.Time min	Rel.Area %	Peak Name
1	98,83	97,82	.
2	104,14	2,18	.

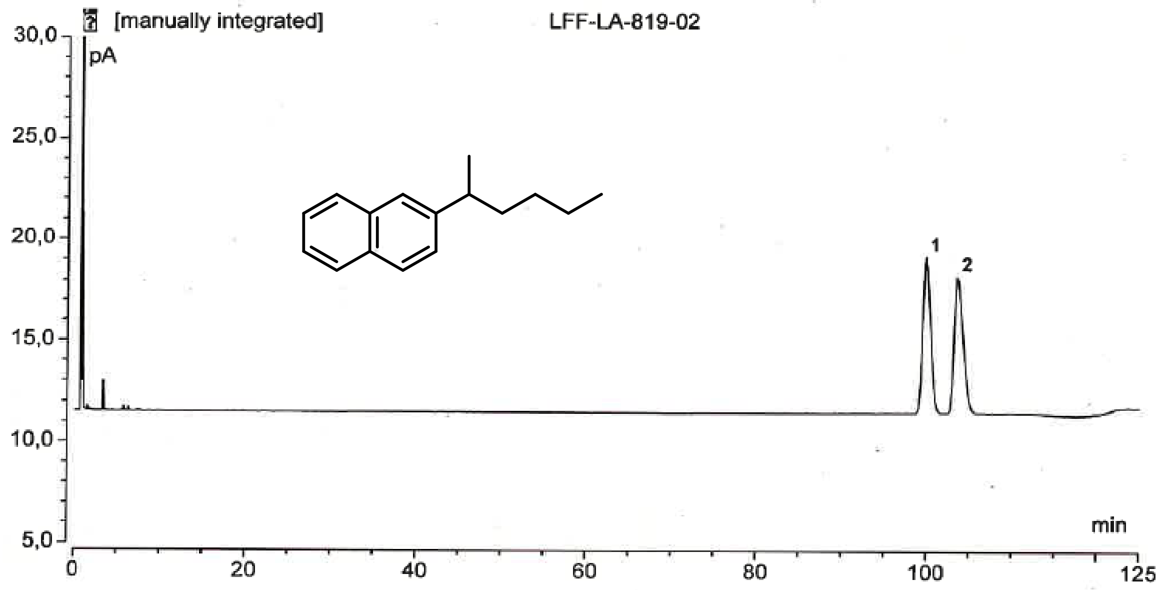

No.	Ret.Time min	Rel.Area %	Peak Name
1	99,76	49,98	.
2	103,45	50,02	.

GC (24 m Cyclodextrin-H, injection temperature: 220 °C, 100 °C iso 45 min, 8 °C/min, 180 °C iso 3 min, 0.5 bar H_2)

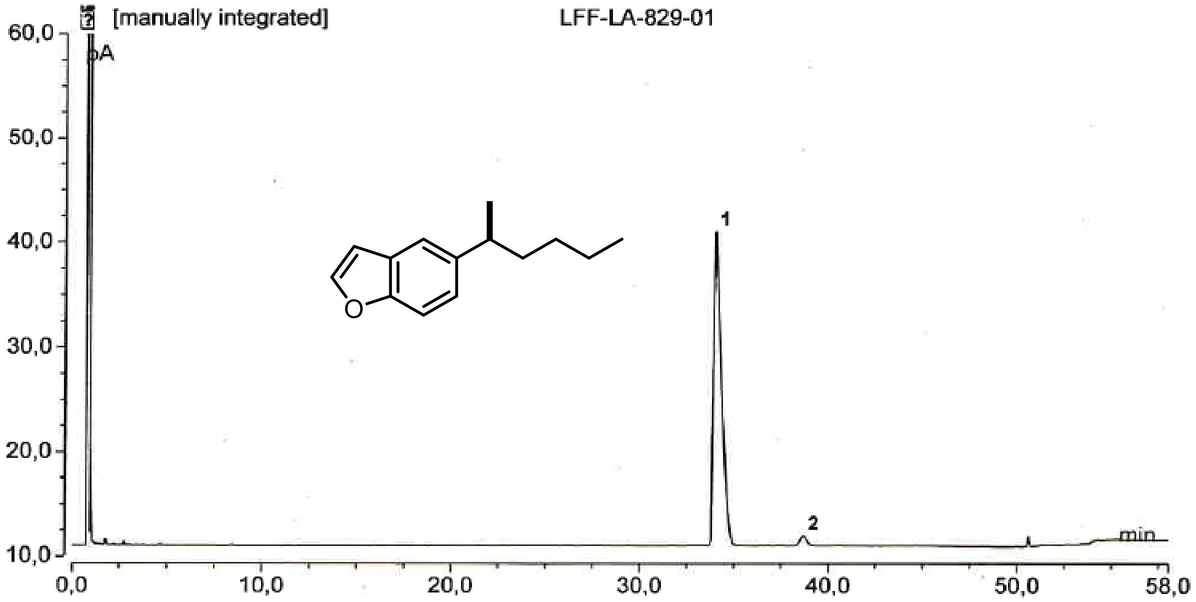

No.	Ret.Time min	Rel.Area %	Peak Name
1	33,97	97,13	.
2	38,68	2,87	.

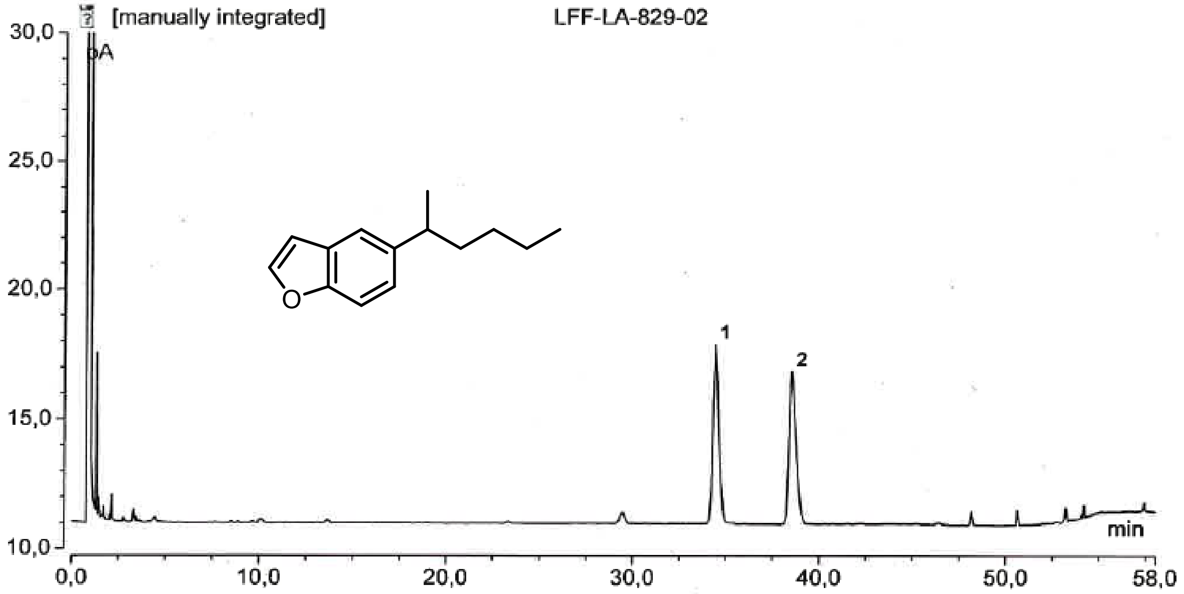

No.	Ret.Time min	Rel.Area %	Peak Name
1	34,42	49,95	.
2	38,49	50,05	.

GC (30 m Cyclosil B, injection temperature: 220 °C, 80 °C iso 30 min, 1 °C/min, 95 °C iso 5 min, 8 °C/min, 220 °C iso 5 min, 0.5 bar H_2)

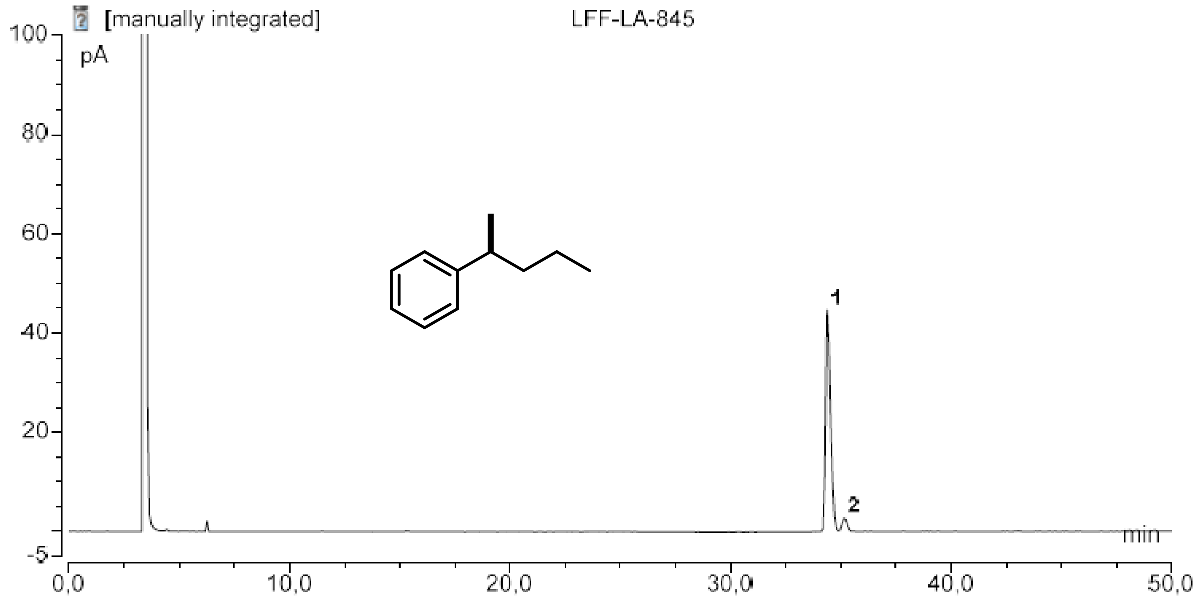

No.	Ret.Time min	Rel.Area %	Peak Name
1	34,39	94,54	Component 11
2	35,18	5,46	Component 12

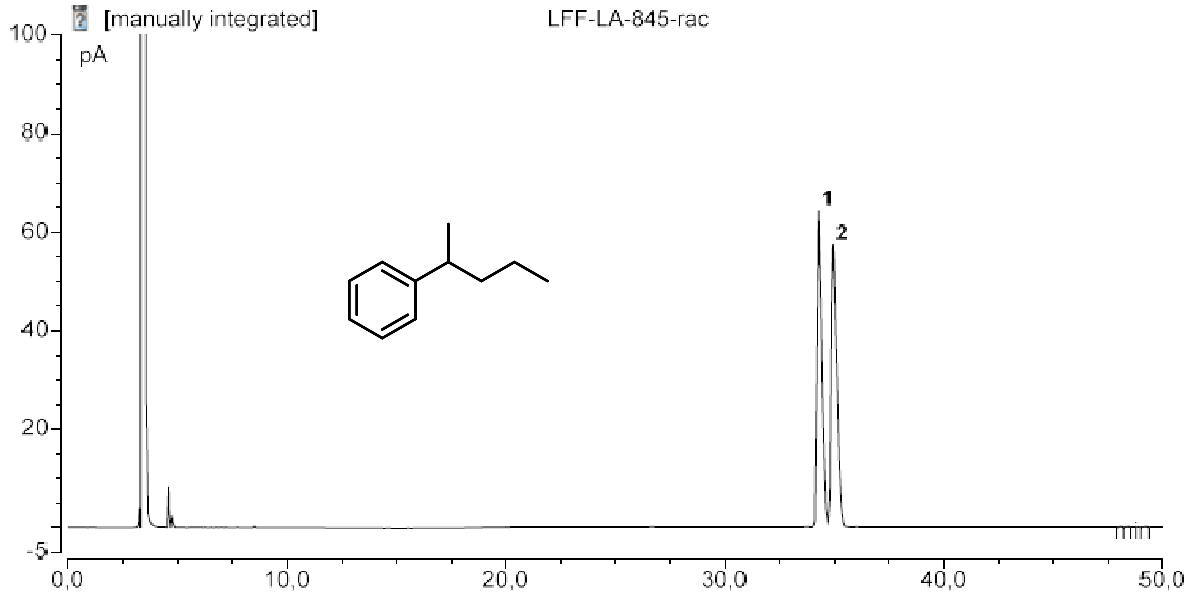

No.	Ret.Time min	Rel.Area %	Peak Name
1	34,27	49,94	Component 11
2	34,94	50,06	Component 12

GC (29.5 m BGB-178/BGB-15, injection temperature: 220 °C, 65 °C iso 142 min, 8 °C/min, 230 °C iso 3 min, 0.6 bar H_2)

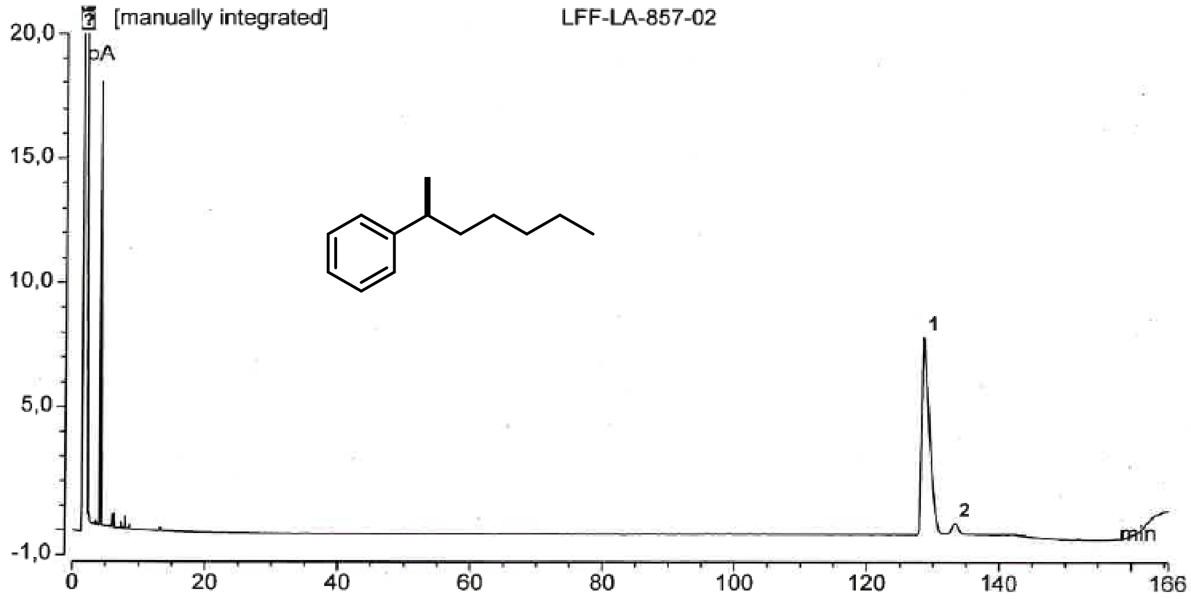

No.	Ret.Time min	Rel.Area %	Peak Name
1	128,39	94,88	.
2	133,25	5,12	.

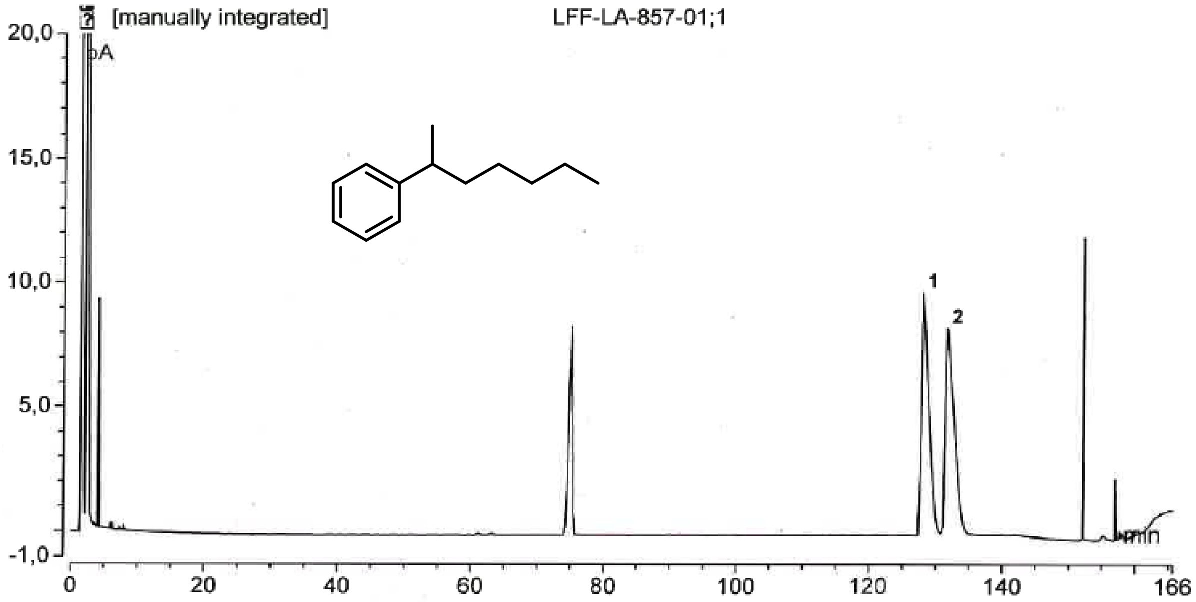

No.	Ret.Time min	Rel.Area %	Peak Name
1	127,90	49,81	.
2	131,56	50,19	.

GC (24 m Cyclodextrin-H, injection temperature: 220 °C, 90 °C iso 25 min, 8 °C/min, 180 °C, 0.5 bar H_2)

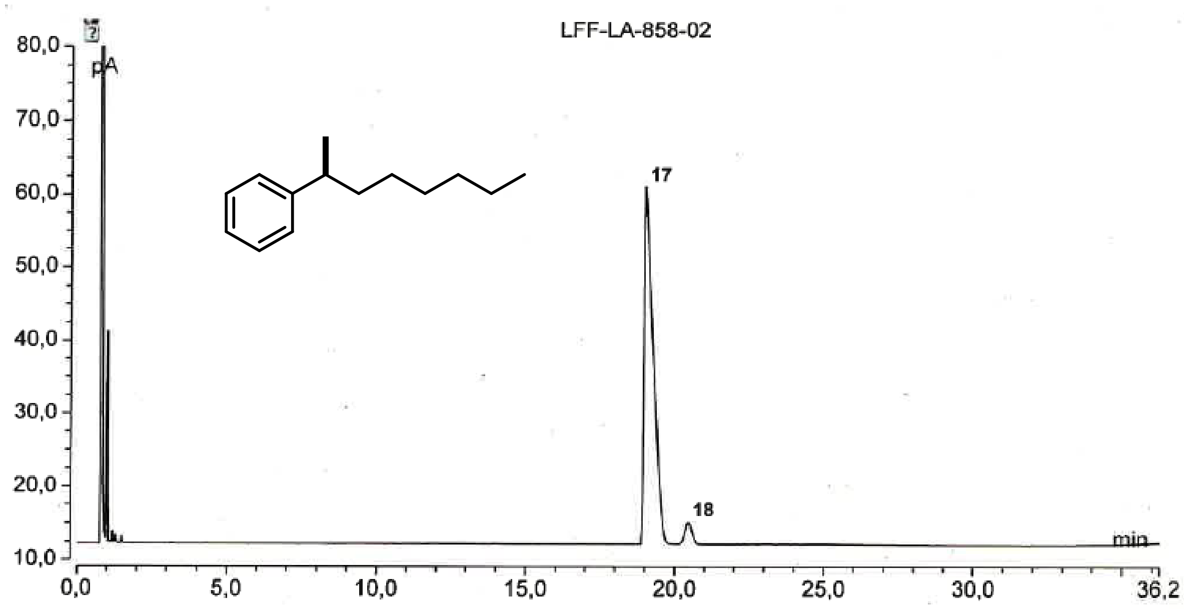

No.	Ret.Time min	Rel.Area %	Peak Name
17	18,97	95,37	.
18	20,46	4,63	.

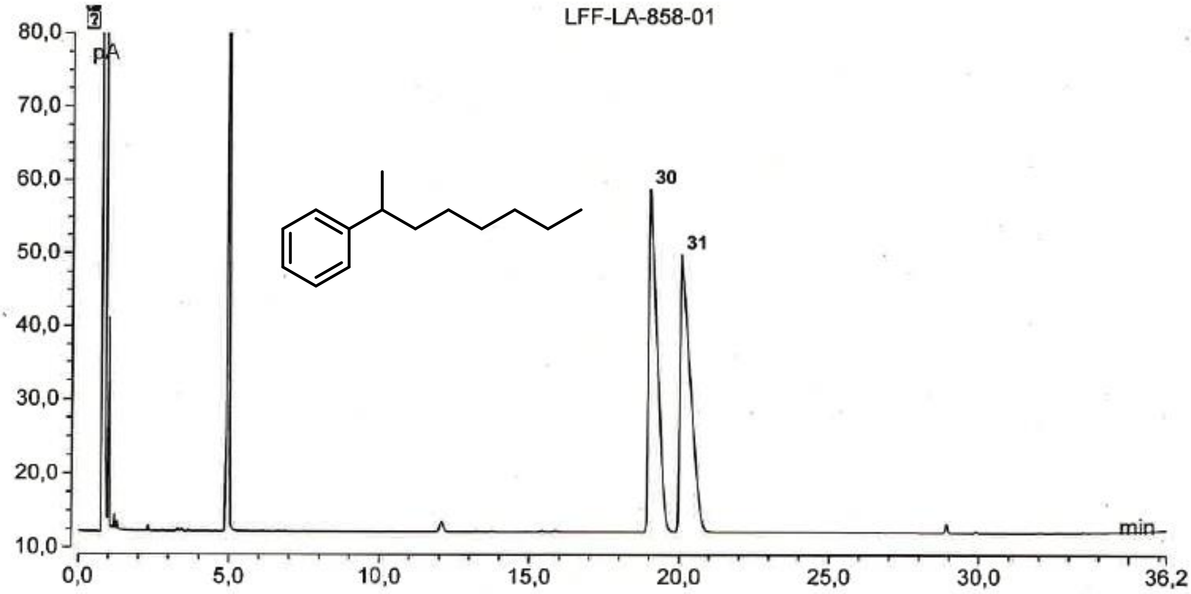

No.	Ret.Time min	Rel.Area %	Peak Name
30	18,98	49,99	.
31	20,03	50,01	.

GC (30 m Cyclosil B, injection temperature: 220 °C, 90 °C iso 30 min, 1 °C/min, 100 °C iso 5 min, 8 °C/min, 220 °C iso 5 min, 0.5 bar H_2)

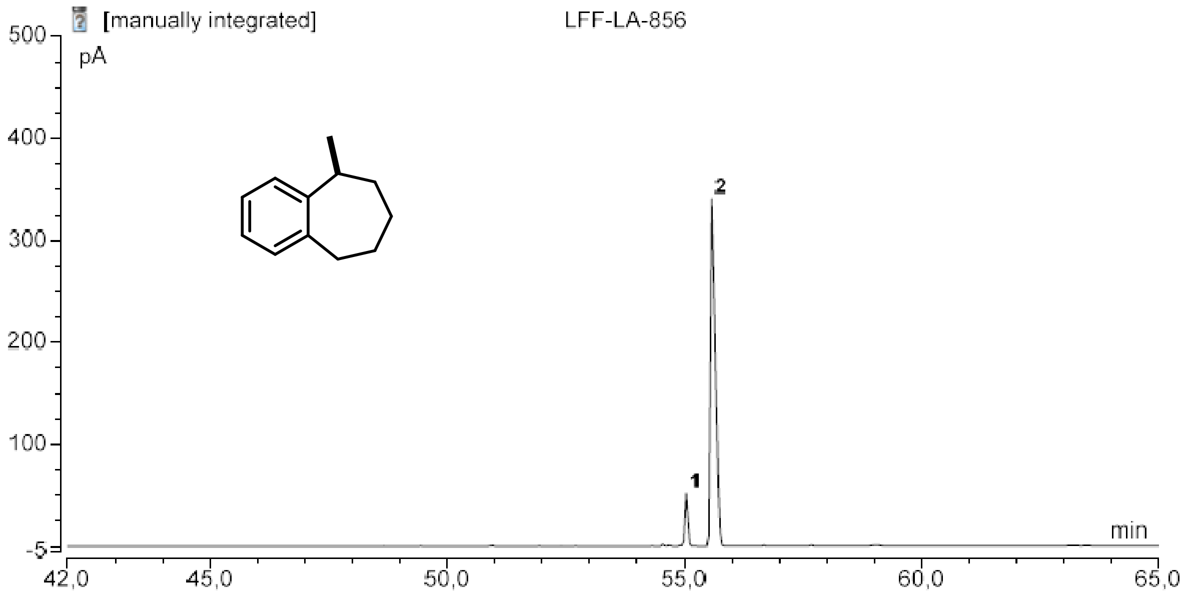

No.	Ret.Time min	Rel.Area %	Peak Name
1	55,04	7,58	Component 23
2	55,57	92,42	Component 24

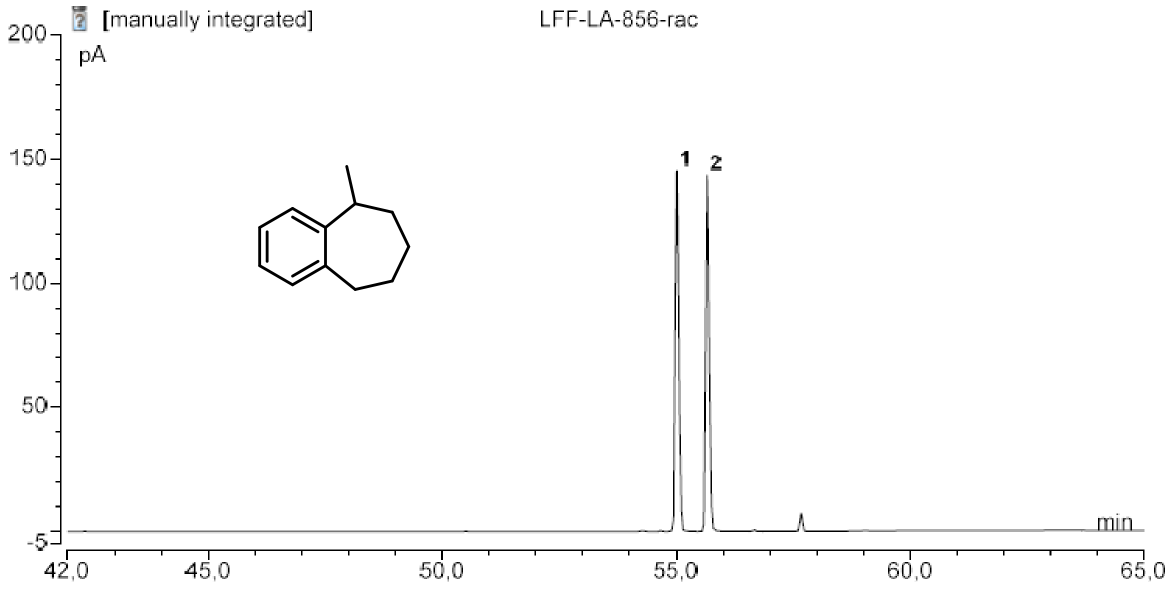

No.	Ret.Time min	Rel.Area %	Peak Name
1	55,01	49,96	Component 23
2	55,66	50,04	Component 24

GC (25 m Ivadex-7/PS086, injection temperature: 220 °C, 100 °C iso 62 min, 8 °C/min, 220 °C, 0.6 bar H_2)

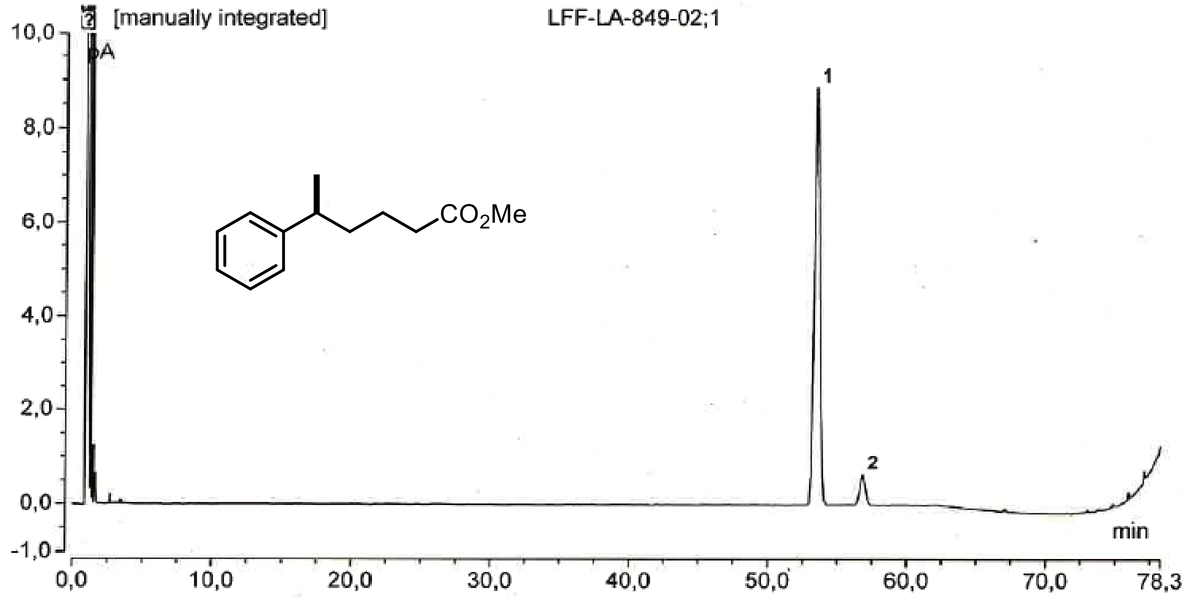

No.	Ret.Time min	Rel.Area %	Peak Name
1	53,39	92,89	.
2	56,83	7,11	.

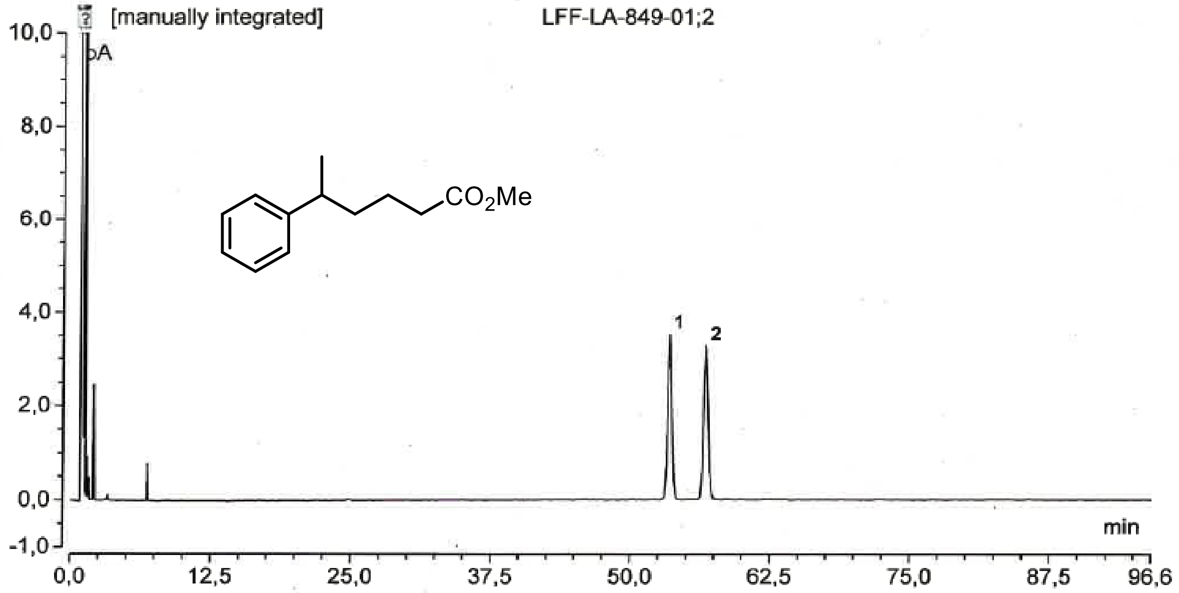

No.	Ret.Time min	Rel.Area %	Peak Name
1	53,51	49,96	.
2	56,73	50,04	.

GC (30 m Cyclosil B, injection temperature: 220 °C, 110 °C iso 60 min, 8 °C/min, 220 °C iso 5 min, 0.5 bar H_2)

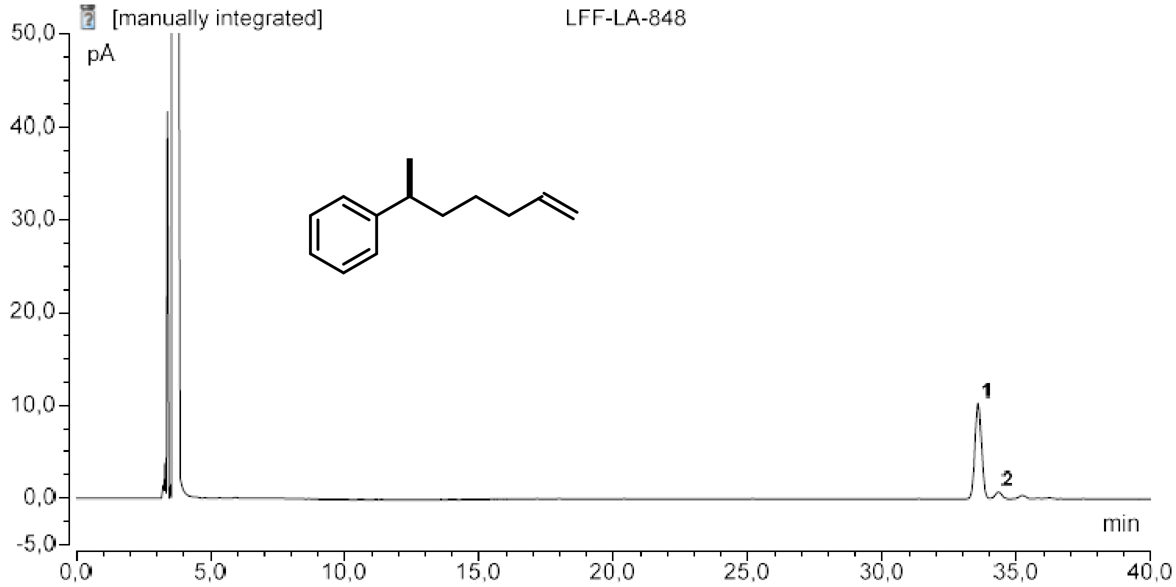

No.	Ret.Time min	Rel.Area %	Peak Name
1	33,57	92,91	Component 11
2	34,33	7,09	Component 12

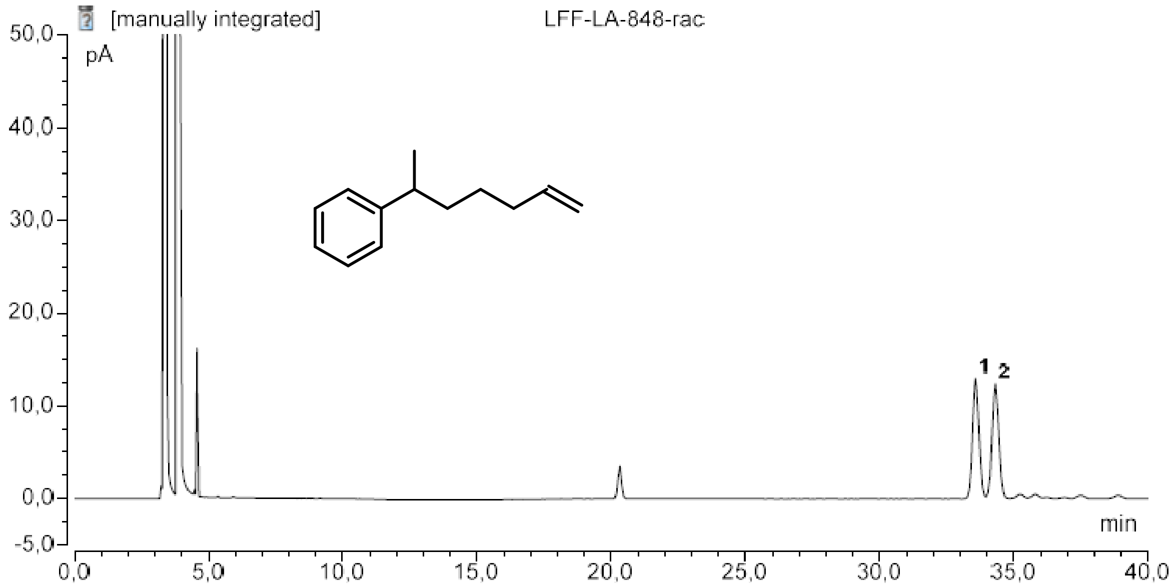

No.	Ret.Time min	Rel.Area %	Peak Name
1	33,57	49,95	Component 11
2	34,31	50,05	Component 12

GC (25 m Hydrodex-beta-TBDAc-CD, injection temperature: 220 °C, 90 °C iso 220 min, 8 °C/min, 220 °C iso 3 min, 0.6 bar H_2)

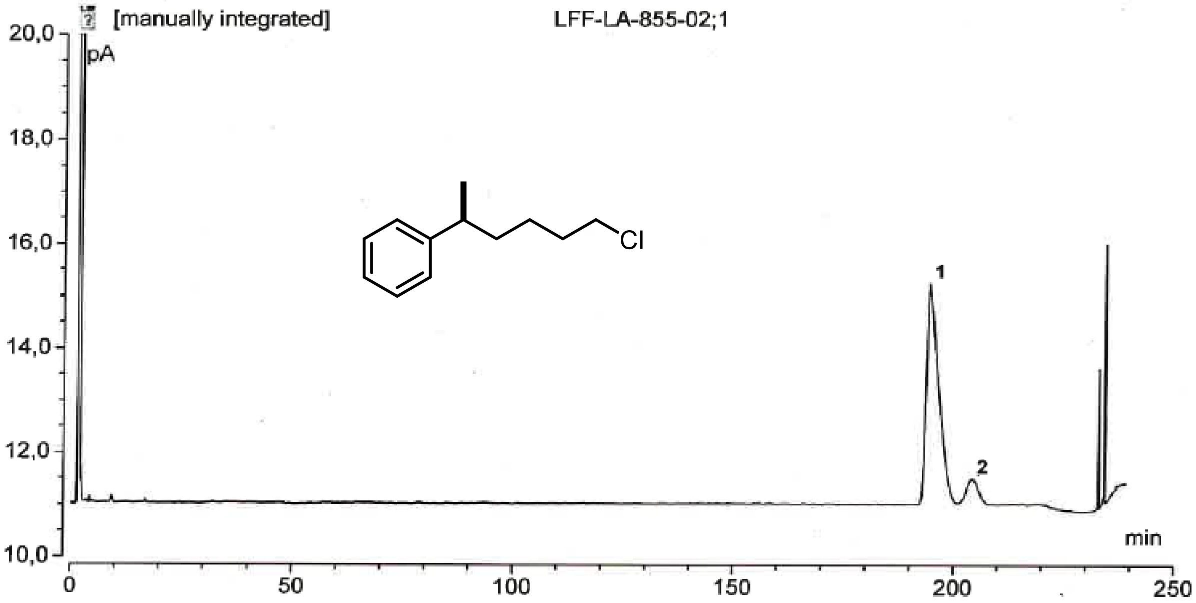

No.	Ret.Time min	Rel.Area %	Peak Name
1	194,24	89,94	.
2	204,23	10,06	.

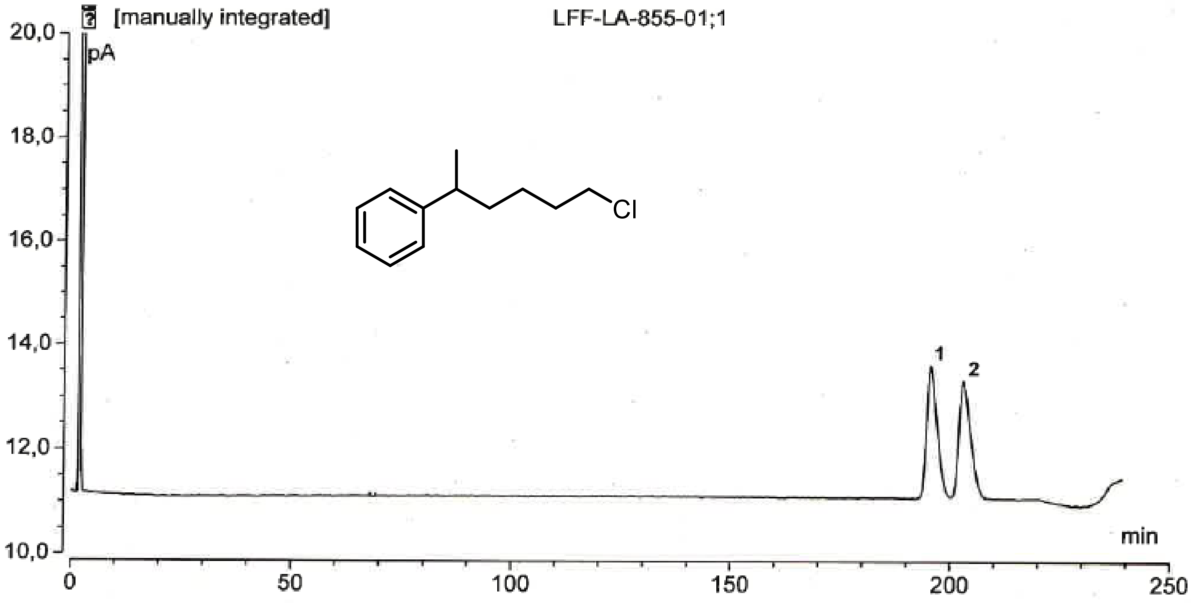

No.	Ret.Time min	Rel.Area %	Peak Name
1	195,18	50,02	.
2	202,73	49,98	.

11. Crystallographic Data

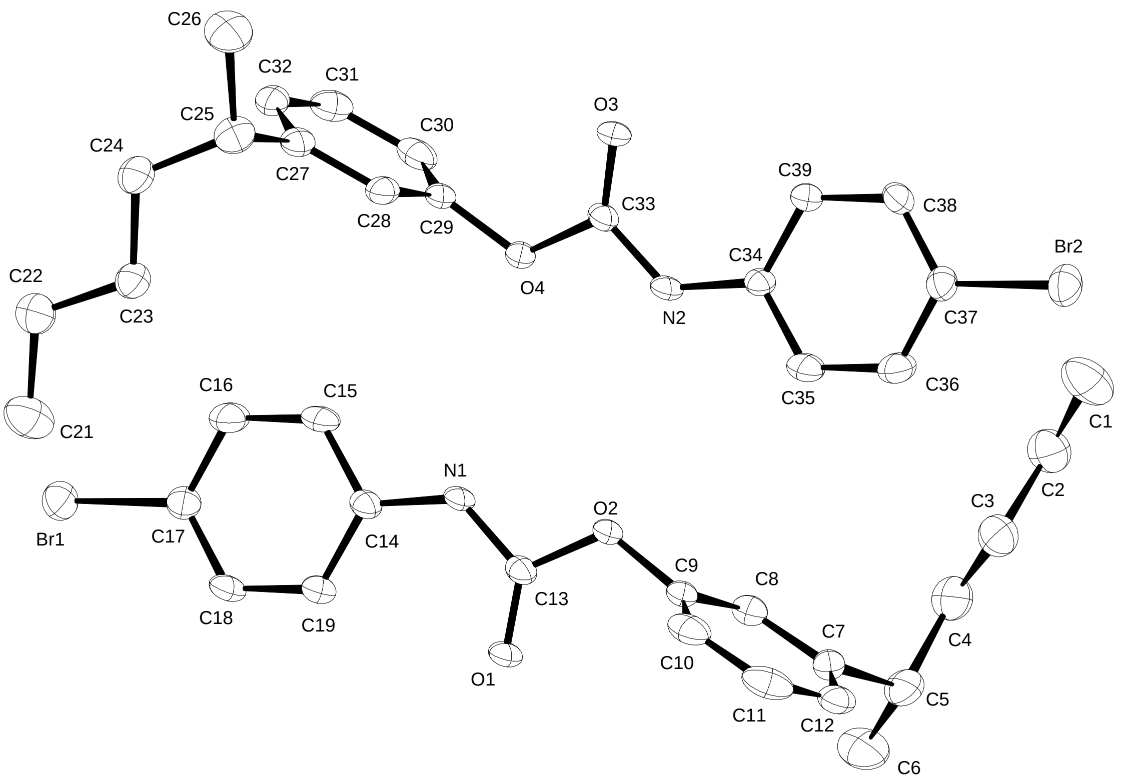

Table S23: *Crystal data and structure refinement.*

Identification code	16205	
Empirical formula	$C_{19} H_{22} Br N O_2$	
Color	colorless	
Formula weight	376.28 g · mol^{-1}	
Temperature	100(2) K	
Wavelength	0.71073 Å	
Crystal system	MONOCLINIC	
Space group	**$P2_1$, (no. 4)**	
Unit cell dimensions	a = 9.5575(3) Å	α= 90°.

	b = 9.6647(2) Å	β= 99.7280(10)°.
	c = 19.7404(5) Å	γ = 90°.
Volume	1797.21(8) $Å^3$	
Z	4	
Density (calculated)	1.391 $Mg \cdot m^{-3}$	
Absorption coefficient	2.296 mm^{-1}	
F(000)	776 e	
Crystal size	0.220 x 0.053 x 0.046 mm^3	
θ range for data collection	2.093 to 31.548°.	
Index ranges	$-14 \le h \le 14$, $-14 \le k \le 14$, $-29 \le l \le 29$	
Reflections collected	252398	
Independent reflections	11968 [R_{int} = 0.0936]	
Reflections with I>2σ(I)	9970	
Completeness to θ = 25.242°	99.9 %	
Absorption correction	Gaussian	
Max. and min. transmission	0.94 and 0.76	
Refinement method	Full-matrix least-squares on F^2	
Data / restraints / parameters	11968 / 1 / 435	
Goodness-of-fit on F^2	1.015	
Final R indices [I>2σ(I)]	R_1 = 0.0328	wR^2 = 0.0722
R indices (all data)	R_1 = 0.0475	wR^2 = 0.0785
Absolute structure parameter	0.006(4)	
Largest diff. peak and hole	0.6 and -0.3 $e \cdot Å^{-3}$	

Table S24: *Bond lengths [Å] and angles [°].*

Br(1)-C(17)	1.899(3)	O(1)-C(13)
1.210(4)	O(2)-C(9)	1.404(4)
O(2)-C(13)	1.360(4)	N(1)-H(1)
0.81(4)	N(1)-C(13)	1.349(4)
N(1)-C(14)	1.406(4)	C(1)-C(2)
1.519(6)	C(2)-C(3)	1.518(5)
C(3)-C(4)	1.509(5)	C(4)-C(5)
1.534(5)	C(5)-H(5)	1.02(4)
C(5)-C(6)	1.531(6)	C(5)-C(7)
1.520(4)	C(7)-C(8)	1.392(4)
C(7)-C(12)	1.386(4)	C(8)-C(9)
1.379(4)	C(9)-C(10)	1.373(5)
C(10)-C(11)	1.376(5)	C(11)-C(12)
1.383(5)	C(14)-C(15)	1.388(5)
C(14)-C(19)	1.397(5)	C(15)-C(16)
1.386(5)	C(16)-C(17)	1.387(5)
C(17)-C(18)	1.379(4)	C(18)-C(19)
1.385(5)	Br(2)-C(37)	1.901(3)
O(3)-C(33)	1.207(4)	O(4)-C(29)
1.405(4)	O(4)-C(33)	1.367(4)
N(2)-H(2)	0.77(4)	N(2)-C(33)
1.348(4)	N(2)-C(34)	1.411(4)
C(21)-C(22)	1.523(7)	C(22)-C(23)
1.500(5)	C(23)-C(24)	1.521(5)
C(24)-C(25)	1.523(4)	C(25)-H(25)
0.93(3)	C(25)-C(26)	1.537(5)
C(25)-C(27)	1.518(4)	C(27)-C(28)
1.382(4)	C(27)-C(32)	1.393(4)
C(28)-C(29)	1.380(4)	C(29)-C(30)
1.379(5)	C(30)-C(31)	1.388(5)
C(31)-C(32)	1.384(4)	C(34)-C(35)
1.389(5)	C(34)-C(39)	1.392(5)
C(35)-C(36)	1.386(5)	C(36)-C(37)
1.378(5)	C(37)-C(38)	1.378(4)

C(38)-C(39)	1.391(4)	

C(13)-O(2)-C(9)	117.6(3)	C(13)-N(1)-H(1)
113(3)	C(13)-N(1)-C(14)	127.8(3)
C(14)-N(1)-H(1)	119(3)	C(3)-C(2)-C(1)
114.9(3)	C(4)-C(3)-C(2)	113.7(3)
C(3)-C(4)-C(5)	115.3(3)	C(4)-C(5)-H(5)
105(2)	C(6)-C(5)-C(4)	111.5(3)
C(6)-C(5)-H(5)	110(2)	C(7)-C(5)-C(4)
112.2(3)	C(7)-C(5)-H(5)	107(2)
C(7)-C(5)-C(6)	111.1(3)	C(8)-C(7)-C(5)
121.8(3)	C(12)-C(7)-C(5)	120.4(3)
C(12)-C(7)-C(8)	117.8(3)	C(9)-C(8)-C(7)
119.7(3)	C(8)-C(9)-O(2)	117.5(3)
C(10)-C(9)-O(2)	120.0(3)	C(10)-C(9)-C(8)
122.3(3)	C(9)-C(10)-C(11)	118.3(3)
C(10)-C(11)-C(12)	120.1(3)	C(11)-C(12)-C(7)
121.8(3)	O(1)-C(13)-O(2)	124.0(3)
O(1)-C(13)-N(1)	128.0(3)	N(1)-C(13)-O(2)
108.0(3)	C(15)-C(14)-N(1)	116.4(3)
C(15)-C(14)-C(19)	119.9(3)	C(19)-C(14)-N(1)
123.6(3)	C(16)-C(15)-C(14)	120.5(3)
C(15)-C(16)-C(17)	119.0(3)	C(16)-C(17)-Br(1)
118.9(3)	C(18)-C(17)-Br(1)	120.2(2)
C(18)-C(17)-C(16)	120.9(3)	C(17)-C(18)-C(19)
120.2(3)	C(18)-C(19)-C(14)	119.4(3)
C(33)-O(4)-C(29)	118.0(3)	C(33)-N(2)-H(2)
113(3)	C(33)-N(2)-C(34)	127.3(3)
C(34)-N(2)-H(2)	120(3)	C(23)-C(22)-C(21)
114.9(3)	C(22)-C(23)-C(24)	112.6(3)
C(23)-C(24)-C(25)	115.3(3)	C(24)-C(25)-H(25)
107(2)	C(24)-C(25)-C(26)	110.8(3)
C(26)-C(25)-H(25)	107(2)	C(27)-C(25)-C(24)
111.8(3)	C(27)-C(25)-H(25)	110(2)
C(27)-C(25)-C(26)	110.3(3)	C(28)-C(27)-C(25)

120.3(3)	C(28)-C(27)-C(32)	118.1(3)
C(32)-C(27)-C(25)	121.7(3)	C(29)-C(28)-C(27)
120.2(3)	C(28)-C(29)-O(4)	118.4(3)
C(30)-C(29)-O(4)	118.8(3)	C(30)-C(29)-C(28)
122.4(3)	C(29)-C(30)-C(31)	117.6(3)
C(32)-C(31)-C(30)	120.7(3)	C(31)-C(32)-C(27)
121.2(3)	O(3)-C(33)-O(4)	123.5(3)
O(3)-C(33)-N(2)	128.7(3)	N(2)-C(33)-O(4)
107.8(3)	C(35)-C(34)-N(2)	116.6(3)
C(35)-C(34)-C(39)	119.6(3)	C(39)-C(34)-N(2)
123.8(3)	C(36)-C(35)-C(34)	120.2(3)
C(37)-C(36)-C(35)	119.6(3)	C(36)-C(37)-Br(2)
119.3(3)	C(36)-C(37)-C(38)	120.9(3)
C(38)-C(37)-Br(2)	119.8(2)	C(37)-C(38)-C(39)
119.7(3)	C(38)-C(39)-C(34)	119.9(3)

Note: Two additional crystals from the same batch were measured to confirm the correct assignment of the absolute configuration. The analysis is consistent with the data presented.

12. Computational Details

Possible TS conformations were explored by the artificial force induced reaction (AFIR) method[16] implemented in the global route reaction mapping (GRRM) program.[17] An extensive conformational search has been performed on possible catalyst substrate orientations at GFN2-xTB level of theory[18] implemented in Orca 4.2.1[19], using SC-AFIR with constraint. Molecular geometries were optimized at r^2SCAN-3c[20] implemented in Orca 5.0.3 program.[21] In the case of transition state optimization, they were combined with GRRM program. Thermal free energy corrections have been performed at the same level of theory using Orca 6.0.1 program[22], and the temperature was set at 253.15K. Transition state structures were verified by the presence of a single imaginary vibrational frequency and the corresponding intrinsic reaction coordinates (IRC). Solvation effect has been accounted by using SMD (cyclohexane) solvation model as implemented in Orca 6.0.1 program. All single point energy is calculated at SMD(methylcyclohexane)-ωB97X-V/def2-TZVPP level of theory.[23,24] RI approximation was used with AutoAux option implemented in Orca 6.0.1 program. IGMH analysis was performed using Multiwfn[25,26] using the default parameters with isovalue of 0.004 a.u. The wavefunction file was generated at ωB97XD/def2-TZVPP level of theory using Gaussian 16.[27] The visualizations of the molecular geometries were generated using the ChimeraX[28] version 1.9 followed by rendering with Blender version 4.0.[29] Conversion of enantiomeric ratio and ΔΔG was performed based on the Boltzmann distribution as follows: $\Delta\Delta G = RT\ln(pdt(R)/pdt(S))$.[30]

Results and Discussion

The DFT calculation is performed using substrate **1a** and catalyst **7b**, and the temperature is set at 253.15 K. The calculated energy diagram and the proposed mechanism are depicted in Figure 4 in the main text. Intermediates **I–IV** correspond to the reactants and products connected by the IRC pathway from each transition state. **TS1** was calculated in the absence of silane; therefore, the energy of silane is added to **I**, **TS1**, and **II** in the energy diagram for better comparison. For all **TS1**, **TS2**, and **TS2'**, a few negative vibrational modes were initially observed; however, each transition state was confirmed by a single distinct imaginary frequency corresponding to the reaction coordinate, as well as by intrinsic reaction coordinate (IRC) analysis. The additional negative frequencies, all greater than −30 cm^{-1}, are likely numerical artifacts and do not correspond to meaningful reaction coordinates. The calculated energy difference between **TS2** and **TS2'** agreed well with the experimental one (1.7 kcal/mol (calc) vs. 1.6 kcal/mol (exp)). The anion of catalyst **7b** appears to possess a relatively rigid structure, as indicated by the nearly identical geometries observed in **TS2** and **TS2'** (Figure S22A). This rigidity is presumably due

to multiple π–π interactions between the wings or between a wing and the backbone. Additionally, the anion is further stabilized by noncovalent interactions between aromatic C–H bonds and the oxygen atoms of the sulfonyl groups at the catalytic active site, further rigidifying the structure of the counterion. These interactions in **TS2** is visualized by IGMH analysis (Figure S22B).

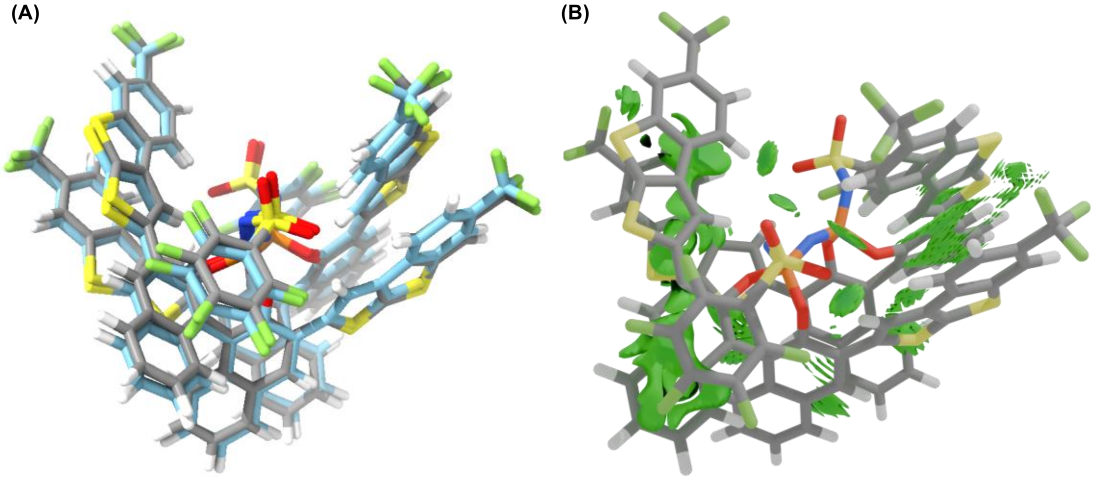

***Figure S22**: (**A**) Overlaid image of the counteranion structures of **TS2** and **TS2'**, visualized using ChimeraX. Gray: **TS2**, light blue: **TS2'**. (**B**) Visualization of multiple intramolecular noncovalent interactions within the catalyst anion, contributing to the stabilization of **TS2**.*

For a better understanding of the origin of enantioselectivity, distortion-interaction analysis was conducted following the Houk-Bickelhaupt protocol[31] (Table S25). Gas phase single point energies of the optimized **TS2** and **TS2'**, leading to the major enantiomer and minor enantiomer, respectively, were calculated at ωB97X-V/def2-TZVPP level of theory. Then they were decomposed into the catalyst fragment and the substrate fragment. The result suggests that the interactions between the catalyst and the substrates would be the major factor in controlling the enantioselectivity.

Table S25**: Summary of the distortion-interaction analysis of **TS2** and **TS2'

TS	ωB97X-V/def2-TZVPP (in hartree)	Relative energy ΔΔE (kcal/mol)
**TS2**	-12224.30919	4.22
**TS2'**	-12224.30247	

	Substrate fragment	
subst-**TS2**	-916.451405	1.19
subst-**TS2’**	-916.4495155	
	Catalyst fragment	
cat-**TS2**	-11307.69917	-0.18
cat-**TS2’**	-11307.69945	
Total distortion		1.01
Total interaction		3.21 (major factor)

Table S26*: Energy table of the optimized structures. Energies are given in Hartree. Computed single point energies (E), Gibbs free energy corrections (G_{corr}), Gibbs free energies (G), and the most negative imaginary frequencies for transition states are provided.*

Structures	E (solv)	G_{corr}	G (solv)	Imaginary Frequency
**1a**	-466.9527635	0.21588128	-466.7368823	-
**7b (H-X*)**	-11308.24367	1.02415917	-11307.21951	-
**I**	-11775.21621	1.26204838	-11773.95416	-
**TS1**	-11775.19437	1.25792388	-11773.93645	-87.16552907
**II**	-11775.21131	1.26150441	-11773.94981	-
**$EtMe_2SiH$**	-449.155673	0.12116426	-449.0345087	-
**III**	-12224.37949	1.40123965	-12222.97825	-
**III’**	-12224.37563	1.40053054	-12222.9751	-
**TS2**	-12224.37715	1.40256706	-12222.97458	-973.85598462
**TS2’**	-12224.37255	1.40061657	-12222.97193	-863.08787083
**IV**	-12224.38988	1.4048345	-12222.98504	-
**IV’**	-12224.38319	1.40486367	-12222.97833	-
**2a’**	-468.185491	0.23836337	-467.9471276	-
**[Si]-X***	-11756.24477	1.14717926	-11755.09759	-

Cartesian coordinates of the optimized structures

1a

C	-1.184631	-1.194955	-0.941685
C	-0.213718	-1.249602	0.050952
C	-0.224518	-0.344780	1.120668
C	-1.227436	0.633496	1.148497
C	-2.201092	0.685422	0.158253
C	-2.185090	-0.228701	-0.891287
H	-1.152616	-1.902987	-1.765053
H	0.582340	-1.986635	-0.007307
H	-1.258776	1.350001	1.964122
H	-2.977124	1.444154	0.206229
H	-2.942138	-0.183079	-1.668654
C	0.804311	-0.420529	2.185522
C	1.225924	-1.601109	2.652666
H	0.796638	-2.536869	2.307152
H	1.997821	-1.657703	3.414551
C	1.367867	0.880406	2.705533
H	2.108694	0.656010	3.482382
H	0.572327	1.458769	3.195458
C	2.011210	1.744406	1.609685
H	1.249595	2.023955	0.869164
H	2.365163	2.680854	2.062117
C	3.171341	1.049315	0.900958
H	2.810823	0.117861	0.445177
H	3.924734	0.752571	1.644469
C	3.812806	1.930313	-0.168258
H	4.642293	1.418322	-0.666413
H	3.080768	2.205995	-0.935772
H	4.203946	2.858068	0.265037

7b

O	2.500882	2.670018	-1.606054
O	0.491420	2.356010	-0.087538
O	2.312999	-1.609496	1.045387
O	3.758733	-1.243170	-0.985016
S	-0.045904	-2.415116	-1.833877
S	0.159124	2.672258	-3.841201
O	-1.053751	-3.003880	-0.943177
O	-0.414043	-1.097019	-2.422888
O	0.552706	3.951283	-3.271258
O	0.689029	2.183382	-5.110140
N	1.419193	-2.415294	-1.203938
N	0.518839	1.474822	-2.670455
P	2.206932	-1.230057	-0.523644
N	1.748982	0.268755	-0.657464
P	1.343491	1.628726	-1.237880
H	0.211911	0.506989	-2.870921
C	0.315813	3.740512	-0.054772
C	3.306678	3.075356	-0.529419
C	3.239179	-0.879636	1.790950

C	4.569577	-2.298785	-0.570545	C	5.395079	2.963099	0.610867
C	-1.015069	4.232583	-0.167208	C	2.763817	3.951969	0.380932
C	4.986203	-3.252123	-1.543711	H	2.003314	5.409070	2.561018
C	4.631829	2.562988	-0.472562	H	3.411096	5.975860	4.490334
C	2.755221	0.152435	2.644183	C	7.825754	0.071230	3.004832
C	2.135194	8.218750	0.050839	C	6.934448	-0.648329	2.245318
C	2.347954	6.862359	0.107493	C	5.539241	-0.471967	2.402053
C	1.259391	5.958651	0.098710	C	5.077258	0.519361	3.321882
C	-0.062200	6.485762	-0.015332	C	6.021188	1.219153	4.113512
C	-0.248038	7.890503	-0.055583	C	7.366776	0.996383	3.965111
C	0.826879	8.740741	-0.015877	H	8.892497	-0.078791	2.867578
H	2.984561	8.895222	0.048121	H	7.299568	-1.361017	1.514633
H	3.360856	6.477293	0.144540	C	4.569737	-1.219317	1.663036
C	1.421941	4.539678	0.150091	C	3.701650	0.803589	3.418308
C	-1.156233	5.610346	-0.122050	H	5.656437	1.951439	4.828445
H	-1.259725	8.279633	-0.134646	H	3.388215	1.571602	4.121638
H	-2.141225	6.056821	-0.225183	C	7.325360	-5.612838	2.020148
C	5.162874	4.951343	3.736368	C	7.021094	-5.454851	0.692251
C	5.677939	4.172909	2.730914	C	6.215204	-4.372704	0.256992
C	4.880587	3.800501	1.620620	C	5.750928	-3.420506	1.215176
C	3.541638	4.291606	1.532284	C	6.056588	-3.630219	2.580503
C	3.031963	5.067694	2.598945	C	6.824073	-4.700641	2.972005
C	3.821954	5.383882	3.677962	H	6.216981	-4.992741	-1.786769
H	6.426370	2.628959	0.697620	H	7.377771	-6.168495	-0.045850
H	6.701405	3.812433	2.777128	H	5.669401	-2.941191	3.323221

H	7.039922	-4.850154	4.025638
C	5.841521	-4.245329	-1.092923
C	4.967954	-2.319968	0.752019
H	0.675379	9.815175	-0.055022
H	5.782710	5.227634	4.584460
H	8.081377	1.540084	4.576449
H	7.935595	-6.450395	2.344360
C	-2.177987	3.369960	-0.351367
C	-2.298413	2.039147	-0.682623
S	-3.789575	4.129831	-0.240234
C	-3.637169	1.627044	-0.901040
H	-1.465027	1.362392	-0.786131
C	-4.545957	2.652171	-0.697987
C	-4.280127	0.421677	-1.343282
C	-5.677957	0.593124	-1.452585
S	-6.202321	2.225426	-1.003488
C	-3.720381	-0.820976	-1.662257
H	-2.650316	-0.975386	-1.582955
C	-4.543254	-1.852115	-2.070239
H	-4.112860	-2.818636	-2.313885
C	-6.514782	-0.437733	-1.861495
H	-7.587803	-0.301067	-1.933121
C	-5.930511	-1.659243	-2.168280
C	-6.775382	-2.818519	-2.609123
F	-6.376901	-3.294708	-3.814430
F	-8.083130	-2.498156	-2.717102
F	-6.688448	-3.856163	-1.738367
C	4.520720	-3.226434	-2.924955
C	3.675405	-2.365904	-3.583381
S	5.001528	-4.568593	-3.999742
C	3.369214	-2.770997	-4.903665
H	3.277293	-1.466102	-3.144847
C	4.024000	-3.934816	-5.269001
C	2.476685	-2.310551	-5.924762
S	3.637745	-4.506985	-6.864760
C	2.513042	-3.152365	-7.059113
C	1.611004	-1.209896	-5.923008
C	1.719651	-2.914138	-8.174630
H	1.569152	-0.547167	-5.063389
C	0.812474	-0.967141	-7.023419
H	1.753346	-3.564065	-9.041805
C	0.868979	-1.816628	-8.140384
H	0.139233	-0.115710	-7.024184
C	-0.027863	-1.511434	-9.304275
F	0.236137	-0.286909	-9.828409
F	-1.332761	-1.496887	-8.936654
F	0.097212	-2.408844	-10.305014
C	1.347926	0.533947	2.683154
C	0.207470	-0.154446	2.342076
S	0.957954	2.180348	3.232453

C	-0.978088	0.606482	2.512896	C	6.553281	-0.983299	-4.981346
H	0.216347	-1.180247	1.999426	C	4.765291	0.335370	-5.947667
C	-0.719636	1.883401	2.989790	C	6.730603	-1.680363	-6.167378
C	-2.387272	0.375513	2.344450	H	3.995574	1.097752	-5.865901
S	-2.132517	2.857880	3.263702	C	4.935678	-0.352691	-7.135908
C	-3.145073	1.507701	2.730275	H	7.477308	-2.462030	-6.257805
C	-3.063437	-0.759547	1.880616	C	5.908249	-1.355782	-7.241553
C	-4.531921	1.512005	2.697070	H	4.298360	-0.131008	-7.986089
H	-2.505508	-1.628336	1.542248	C	6.107673	-2.042294	-8.561270
C	-4.446719	-0.764869	1.844443	F	4.938008	-2.237193	-9.215131
H	-5.102274	2.380769	3.007606	F	6.703179	-3.249455	-8.427756
C	-5.174534	0.358618	2.261174	F	6.894807	-1.307534	-9.389991
H	-4.975280	-1.641635	1.483100	C	0.144938	-3.548954	-3.241602
C	-6.671462	0.273824	2.320051	C	1.029794	-4.630615	-3.233496
F	-7.192864	-0.367271	1.246880	C	-0.666949	-3.381787	-4.367670
F	-7.257125	1.491323	2.387146	C	1.148392	-5.469153	-4.335502
F	-7.081908	-0.418182	3.415298	C	-0.548782	-4.209458	-5.475836
C	5.155103	1.679038	-1.506372	C	0.366065	-5.252803	-5.461809
C	4.788156	1.536875	-2.824277	F	1.780741	-4.920847	-2.174051
S	6.501627	0.596188	-1.097415	F	2.000236	-6.494941	-4.308574
C	5.559370	0.571482	-3.517624	F	0.472658	-6.056880	-6.515014
H	4.008626	2.125726	-3.288361	F	-1.317165	-4.012838	-6.545868
C	6.515824	-0.021202	-2.706036	F	-1.599485	-2.432446	-4.423986
C	5.571910	0.028023	-4.847093	C	-1.638100	2.588178	-3.956423
S	7.466730	-1.249038	-3.488190	C	-2.466598	3.680461	-3.685633

C	-2.224128	1.403387	-4.406765
C	-3.842420	3.577995	-3.863266
F	-1.988252	4.848696	-3.273215
C	-3.594932	1.287089	-4.569192
F	-1.464015	0.345798	-4.687960
C	-4.404674	2.386771	-4.305756
F	-4.625978	4.626418	-3.620008
F	-4.132885	0.147106	-4.985141
F	-5.715253	2.298366	-4.484097

I

O	1.772302	2.599708	-2.197170
O	-0.090575	2.012365	-0.588345
O	1.489000	-1.596097	0.141259
O	2.897144	-1.253357	-1.933446
S	-0.304744	-3.647321	-1.230655
S	0.006250	2.969501	-4.599054
O	-1.061729	-3.007557	-0.137405
O	-1.050299	-4.476628	-2.178973
O	1.367343	2.709331	-5.054368
O	-1.118204	2.807443	-5.514510
N	0.586384	-2.593006	-2.079900
N	-0.400192	1.973712	-3.268178
P	1.357501	-1.361529	-1.453711
N	0.777098	0.040095	-1.904101
P	0.577055	1.555535	-1.975475
H	-1.055587	1.224720	-3.572661
C	-0.399112	3.360235	-0.416707
C	2.583638	2.948771	-1.105033
C	2.516538	-1.033746	0.884805
C	3.675848	-2.369966	-1.641644
C	-1.776995	3.715105	-0.396642
C	3.998492	-3.249830	-2.712810
C	3.924777	2.479567	-1.117899
C	2.144585	-0.087507	1.880999
C	1.000207	7.965538	-0.027752
C	1.341056	6.638065	-0.132601
C	0.344169	5.634061	-0.133766
C	-1.029061	6.027251	-0.073223
C	-1.343616	7.403929	0.060492
C	-0.351750	8.351027	0.089578
H	1.777217	8.723694	-0.042531
H	2.383453	6.350630	-0.224589
C	0.645523	4.242067	-0.229729
C	-2.045321	5.059334	-0.178684
H	-2.388670	7.696619	0.117047
H	-3.074148	5.402750	-0.112767
C	4.518371	4.587411	3.228512
C	5.018819	3.888378	2.159946
C	4.198855	3.578214	1.046900

C	2.846687	4.041920	1.025137
C	2.355569	4.736938	2.155612
C	3.169753	4.998743	3.231034
H	5.744435	2.505803	0.006512
H	6.049279	3.544687	2.153905
C	4.704680	2.822118	-0.027634
C	2.039062	3.743149	-0.119100
H	1.319617	5.055241	2.176206
H	2.769380	5.527256	4.090931
C	7.207308	-0.502632	1.936612
C	6.231023	-1.120447	1.192636
C	4.860674	-0.846507	1.417466
C	4.517420	0.121870	2.412179
C	5.547099	0.720943	3.179228
C	6.864687	0.415416	2.950516
H	8.252116	-0.725678	1.742176
H	6.509999	-1.823111	0.415577
C	3.809713	-1.466228	0.667839
C	3.168442	0.464642	2.628507
H	5.268433	1.438874	3.945734
H	2.935964	1.175192	3.418552
C	6.212325	-6.082758	0.578125
C	5.895226	-5.786951	-0.722865
C	5.168468	-4.612524	-1.045183
C	4.804605	-3.714632	0.006931
C	5.119762	-4.064947	1.340888
C	5.804941	-5.223171	1.619607
H	5.081680	-5.055742	-3.135964
H	6.174829	-6.463120	-1.526291
H	4.803149	-3.413411	2.148874
H	6.023513	-5.484746	2.650479
C	4.779107	-4.344497	-2.371878
C	4.096019	-2.524287	-0.336465
H	-0.606019	9.402906	0.177313
H	5.154788	4.816755	4.078179
H	7.645706	0.881232	3.544476
H	6.753233	-6.994402	0.812781
C	-2.843210	2.745800	-0.615355
C	-2.832753	1.369959	-0.714203
S	-4.497487	3.372435	-0.820643
C	-4.110383	0.811985	-0.967754
H	-1.950831	0.761642	-0.575704
C	-5.103303	1.776769	-1.033209
C	-4.628898	-0.507167	-1.204470
C	-6.024106	-0.479940	-1.412599
S	-6.695621	1.157465	-1.330341
C	-3.961618	-1.736018	-1.254330
H	-2.891117	-1.791143	-1.087947
C	-4.676604	-2.893181	-1.493847
H	-4.154545	-3.844399	-1.540674

C	-6.757879	-1.636703	-1.642441
H	-7.832230	-1.606732	-1.784346
C	-6.067169	-2.840875	-1.680648
C	-6.806814	-4.129358	-1.895186
F	-6.246449	-4.872081	-2.877243
F	-8.102787	-3.933517	-2.227275
F	-6.797291	-4.898180	-0.774829
C	3.539754	-3.016689	-4.077304
C	2.795711	-1.997507	-4.628821
S	3.961378	-4.218825	-5.325164
C	2.547010	-2.160106	-6.010418
H	2.445625	-1.137455	-4.082028
C	3.128557	-3.305540	-6.524455
C	1.868700	-1.400157	-7.017908
S	2.912017	-3.514154	-8.235685
C	2.000776	-1.997277	-8.290275
C	1.166679	-0.198033	-6.901994
C	1.475071	-1.410670	-9.432393
H	1.051936	0.269917	-5.929904
C	0.636674	0.396942	-8.030132
H	1.591319	-1.868087	-10.408653
C	0.798133	-0.205171	-9.287267
H	0.091842	1.332210	-7.942801
C	0.156323	0.442625	-10.475605
F	0.609027	-0.052965	-11.648080
F	0.365232	1.781913	-10.496833
F	-1.194582	0.267210	-10.470311
C	0.747341	0.267052	2.103889
C	-0.394837	-0.479004	1.943545
S	0.376088	1.897278	2.703318
C	-1.572622	0.232891	2.288808
H	-0.394215	-1.501255	1.586970
C	-1.306552	1.533161	2.692338
C	-2.982757	-0.046148	2.284424
S	-2.716024	2.485336	3.051618
C	-3.736083	1.093649	2.655166
C	-3.659580	-1.226741	1.962774
C	-5.122303	1.083642	2.674717
H	-3.100115	-2.108558	1.663874
C	-5.043326	-1.250882	1.992107
H	-5.691799	1.968230	2.939225
C	-5.767194	-0.100715	2.331830
H	-5.572984	-2.158017	1.718924
C	-7.265694	-0.162399	2.387693
F	-7.778026	-0.984767	1.442928
F	-7.834597	1.054243	2.215095
F	-7.704177	-0.629506	3.585903
C	4.449486	1.679896	-2.217209
C	4.149566	1.710281	-3.557224
S	5.702203	0.477431	-1.860232

C	4.905500	0.779453	-4.314145
H	3.420225	2.383465	-3.987112
C	5.767454	0.034376	-3.521137
C	4.986439	0.393524	-5.696281
S	6.676228	-1.175559	-4.373036
C	5.898162	-0.673287	-5.880814
C	4.314750	0.904704	-6.811157
C	6.126066	-1.238848	-7.126085
H	3.601983	1.715297	-6.692218
C	4.542724	0.354879	-8.060429
H	6.815016	-2.066798	-7.254416
C	5.435218	-0.714274	-8.213110
H	4.007867	0.735939	-8.924595
C	5.704615	-1.256103	-9.586469
F	4.597401	-1.246762	-10.365883
F	6.166680	-2.527141	-9.556649
F	6.640670	-0.517238	-10.238997
C	0.926379	-4.750763	-0.456590
C	1.145702	-4.822169	0.918901
C	1.644989	-5.621630	-1.278584
C	2.014481	-5.765372	1.457615
C	2.507460	-6.570645	-0.750186
C	2.678556	-6.654973	0.625704
F	0.541685	-4.005151	1.783156
F	2.209053	-5.822728	2.777292
F	3.485332	-7.576914	1.144700
F	3.176699	-7.398259	-1.555857
F	1.525770	-5.567012	-2.604324
C	-0.052407	4.628875	-3.908718
C	1.075193	5.454014	-3.839255
C	-1.300775	5.180372	-3.601917
C	0.944511	6.803269	-3.532891
F	2.303889	5.001214	-4.063193
C	-1.434436	6.524710	-3.293233
F	-2.396594	4.427427	-3.604248
C	-0.310888	7.343438	-3.285695
F	2.021895	7.583250	-3.483764
F	-2.630746	7.032948	-3.007576
F	-0.434812	8.638921	-3.033839
C	-0.669704	-3.844946	-6.058320
C	-0.782034	-3.809160	-7.445833
C	-1.427607	-2.735467	-8.049580
C	-1.951513	-1.705961	-7.276966
C	-1.852065	-1.728765	-5.877354
C	-1.205240	-2.825467	-5.285394
H	-0.178483	-4.678903	-5.564709
H	-0.373872	-4.614315	-8.050175
H	-1.519736	-2.689670	-9.131078
H	-2.432739	-0.875302	-7.782197
H	-1.137870	-2.914897	-4.207945

C	-2.450307	-0.654227	-5.048233
C	-2.177822	-0.538650	-3.735313
H	-1.489349	-1.206442	-3.228200
H	-2.736307	0.157244	-3.118689
C	-3.405555	0.297171	-5.727863
H	-4.142473	-0.295277	-6.288789
H	-2.842835	0.861031	-6.487309
C	-4.140348	1.292904	-4.835308
H	-4.768033	0.754647	-4.109620
H	-3.423103	1.887605	-4.252676
C	-5.005742	2.255142	-5.648613
H	-5.716213	1.681790	-6.259877
H	-4.363016	2.802508	-6.351862
C	-5.766169	3.244621	-4.769254
H	-6.364530	3.937669	-5.369514
H	-6.443709	2.718948	-4.086647
H	-5.074896	3.837334	-4.159114

II

O	1.836714	2.715115	-2.040433
O	-0.031234	2.119303	-0.441101
O	1.505710	-1.503835	0.163396
O	2.936493	-1.095206	-1.880850
S	-0.253479	-3.503061	-1.314003
S	-0.083529	2.989023	-4.390182
O	-1.051288	-2.924012	-0.216214
O	-0.977132	-4.292646	-2.321653
O	1.252596	2.657447	-4.922379
O	-1.210855	2.882653	-5.332336
N	0.656743	-2.431393	-2.097245
N	-0.479436	2.185859	-3.055761
P	1.381698	-1.175466	-1.422667
N	0.788412	0.203794	-1.839680
P	0.544415	1.741230	-1.907720
H	-1.367552	0.284264	-3.868600
C	-0.329297	3.460561	-0.245149
C	2.636393	3.015960	-0.948506
C	2.519425	-0.963392	0.939694
C	3.693490	-2.224553	-1.608139
C	-1.700326	3.843426	-0.252723
C	4.002675	-3.096914	-2.690449
C	3.981561	2.550417	-0.980957
C	2.137401	-0.050278	1.963316
C	1.137324	8.040399	0.198726
C	1.458203	6.707888	0.095384
C	0.445944	5.719689	0.072536
C	-0.921651	6.136705	0.108860
C	-1.215466	7.517543	0.242444
C	-0.209409	8.448524	0.292637
H	1.927033	8.785588	0.198102

H	2.496844	6.403666	0.017238
C	0.726005	4.323888	-0.034420
C	-1.953398	5.187680	-0.027748
H	-2.256577	7.827254	0.276573
H	-2.977528	5.550118	0.009665
C	4.623889	4.552741	3.406795
C	5.111761	3.874662	2.318501
C	4.281696	3.590989	1.206346
C	2.930727	4.059749	1.206160
C	2.453032	4.733372	2.355487
C	3.277162	4.969462	3.429644
H	5.815952	2.543820	0.123486
H	6.141302	3.528411	2.296037
C	4.775555	2.860700	0.107558
C	2.111517	3.795646	0.063121
H	1.419242	5.058624	2.387511
H	2.886790	5.483421	4.303007
C	7.201840	-0.444653	2.042062
C	6.233518	-1.041118	1.270436
C	4.860512	-0.783466	1.497611
C	4.505623	0.148460	2.522178
C	5.526944	0.725027	3.316696
C	6.847567	0.433916	3.086391
H	8.249222	-0.653480	1.845340
H	6.521182	-1.712617	0.469332
C	3.817371	-1.383322	0.722842
C	3.153551	0.479502	2.737333
H	5.239873	1.417304	4.103232
H	2.913310	1.167418	3.544925
C	6.235346	-5.988361	0.529511
C	5.921212	-5.663301	-0.765292
C	5.189678	-4.485314	-1.061466
C	4.820177	-3.611263	0.008686
C	5.131626	-3.992689	1.335098
C	5.819622	-5.155040	1.588780
H	5.092106	-4.887532	-3.161495
H	6.206033	-6.319258	-1.583609
H	4.811316	-3.360635	2.156827
H	6.035332	-5.439690	2.614159
C	4.791189	-4.193523	-2.380587
C	4.113327	-2.412050	-0.307471
H	-0.447964	9.504431	0.376070
H	5.269277	4.762380	4.254927
H	7.622189	0.884147	3.700443
H	6.779965	-6.902541	0.744915
C	-2.769823	2.889387	-0.523501
C	-2.754342	1.522413	-0.681567
S	-4.414906	3.528207	-0.777877
C	-4.015868	0.979379	-1.026317
H	-1.877164	0.910882	-0.537322

C	-5.006643	1.945127	-1.099717
C	-4.515237	-0.325544	-1.349143
C	-5.896453	-0.291357	-1.645274
S	-6.579790	1.335168	-1.512862
C	-3.843163	-1.553768	-1.397241
H	-2.793428	-1.620144	-1.127773
C	-4.532157	-2.700024	-1.748306
H	-4.004909	-3.649076	-1.789129
C	-6.602905	-1.438144	-1.984491
H	-7.665717	-1.404433	-2.196579
C	-5.904120	-2.638498	-2.040947
C	-6.613005	-3.912595	-2.393443
F	-5.999499	-4.558319	-3.417182
F	-7.896802	-3.707964	-2.763361
F	-6.632554	-4.779138	-1.349489
C	3.482429	-2.871087	-4.034125
C	2.764573	-1.824878	-4.564537
S	3.701334	-4.158150	-5.250942
C	2.360969	-2.039411	-5.902167
H	2.539705	-0.915042	-4.031590
C	2.811673	-3.247453	-6.409999
C	1.590637	-1.305545	-6.859969
S	2.372779	-3.535434	-8.065696
C	1.508700	-1.990720	-8.092106
C	0.964415	-0.062019	-6.727094
C	0.817458	-1.466604	-9.174984
H	1.046075	0.498260	-5.800698
C	0.278978	0.473919	-7.801257
H	0.759851	-1.995205	-10.120158
C	0.199638	-0.230548	-9.012776
H	-0.188092	1.450470	-7.705036
C	-0.623043	0.351257	-10.121502
F	-0.458526	-0.299746	-11.292717
F	-0.342236	1.654713	-10.336826
F	-1.957260	0.291266	-9.825558
C	0.738238	0.298346	2.181022
C	-0.401649	-0.438016	1.969930
S	0.358674	1.907799	2.828056
C	-1.584770	0.266660	2.309794
H	-0.395265	-1.446764	1.577628
C	-1.323935	1.550846	2.765505
C	-2.994847	0.000434	2.243311
S	-2.739591	2.500834	3.107250
C	-3.753698	1.133879	2.623700
C	-3.667658	-1.160630	1.850045
C	-5.139499	1.138062	2.579150
H	-3.102743	-2.037900	1.547817
C	-5.051288	-1.170198	1.813491
H	-5.711858	2.019483	2.848169
C	-5.779427	-0.024665	2.160368

H	-5.577086	-2.061354	1.485529	C	0.944156	-4.650718	-0.550026
C	-7.278467	-0.072193	2.139298	C	1.147344	-4.754381	0.826065
F	-7.749571	-0.855972	1.139818	C	1.671946	-5.503972	-1.381027
F	-7.829006	1.154633	1.982124	C	2.011937	-5.708628	1.353095
F	-7.782522	-0.578419	3.295604	C	2.529733	-6.463625	-0.865951
C	4.487125	1.786576	-2.116080	C	2.687944	-6.577645	0.509249
C	4.129469	1.829503	-3.440912	F	0.530199	-3.961298	1.701670
S	5.768085	0.590196	-1.829733	F	2.189719	-5.797181	2.672995
C	4.855857	0.908542	-4.238788	F	3.491588	-7.509272	1.015701
H	3.364923	2.487746	-3.832380	F	3.209927	-7.271138	-1.684208
C	5.759207	0.161974	-3.496521	F	1.569087	-5.421307	-2.709629
C	4.845159	0.514540	-5.619853	C	-0.030266	4.734357	-3.865637
S	6.611439	-1.054033	-4.402611	C	1.140719	5.491101	-3.819249
C	5.732755	-0.563208	-5.857454	C	-1.225937	5.358537	-3.504945
C	4.095328	1.019335	-6.687259	C	1.108798	6.837522	-3.470838
C	5.850483	-1.155140	-7.105781	F	2.336898	4.977105	-4.105432
H	3.405337	1.841924	-6.521869	C	-1.268595	6.701037	-3.161451
C	4.215625	0.443382	-7.939916	F	-2.372076	4.677126	-3.477490
H	6.516269	-1.995096	-7.273337	C	-0.097655	7.448380	-3.161363
C	5.074788	-0.644915	-8.142639	F	2.236775	7.550440	-3.442655
H	3.623270	0.820514	-8.767648	F	-2.426396	7.277203	-2.829866
C	5.211099	-1.226836	-9.517869	F	-0.130651	8.744228	-2.862702
F	4.041405	-1.198774	-10.202939	C	-0.616884	-4.331488	-6.105708
F	5.629605	-2.514366	-9.492246	C	-0.981313	-4.505154	-7.440377
F	6.111679	-0.540749	-10.269073	C	-1.720949	-3.522727	-8.105118

C	-2.095472	-2.372218	-7.444423
C	-1.732342	-2.166843	-6.086119
C	-0.975133	-3.180183	-5.435439
H	-0.048642	-5.094240	-5.582718
H	-0.695861	-5.412379	-7.965257
H	-1.999603	-3.662013	-9.145078
H	-2.655763	-1.618821	-7.983878
H	-0.682441	-3.086695	-4.397395
C	-2.104443	-0.981104	-5.386186
C	-1.709263	-0.753135	-4.000322
H	-0.975053	-1.439001	-3.588600
H	-2.626226	-0.802789	-3.385839
C	-2.891671	0.106904	-6.009638
H	-3.436448	-0.224415	-6.896816
H	-2.120893	0.817795	-6.365691
C	-3.818545	0.911344	-5.082020
H	-4.553295	0.237719	-4.615961
H	-3.238294	1.373971	-4.276712
C	-4.530349	2.015021	-5.861237
H	-5.131418	1.572675	-6.668061
H	-3.772023	2.649285	-6.338015
C	-5.417471	2.867077	-4.958736
H	-5.902752	3.669080	-5.523620
H	-6.200256	2.262389	-4.487659
H	-4.824943	3.327357	-4.159983

$EtMe_2SiH$

Si	1.112291	-2.612087	-0.193748
H	0.771049	-1.787443	-1.390494
C	-0.480126	-3.096946	0.684814
H	-1.020678	-2.215125	1.045675
H	-1.148644	-3.645432	0.012402
H	-0.276861	-3.741920	1.546984
C	2.025922	-4.156003	-0.763317
H	1.420182	-4.732606	-1.470714
H	2.966811	-3.899238	-1.262202
H	2.262874	-4.812683	0.081160
C	2.206679	-1.579353	0.947346
H	3.097164	-1.265246	0.385637
H	1.670582	-0.653759	1.198424
C	2.627078	-2.306742	2.232970
H	3.257588	-1.674155	2.867412
H	1.756153	-2.601663	2.828617
H	3.196063	-3.216337	2.010841

III

O	1.768152	2.424278	-2.363061
O	-0.151187	1.896314	-0.804228
O	1.488029	-1.616994	0.054884
O	2.939996	-1.253242	-1.990056

S	-0.142342	-3.784714	-1.337421	C	-0.484065	8.249686	-0.401468
S	-0.052428	2.649727	-4.750389	H	1.645565	8.632597	-0.471949
O	-0.974770	-3.181797	-0.278812	H	2.273253	6.259310	-0.521819
O	-0.826654	-4.651519	-2.309964	C	0.552423	4.136967	-0.495594
O	1.314989	2.362778	-5.222136	C	-2.145616	4.935296	-0.577620
O	-1.126828	2.530680	-5.752528	H	-2.515695	7.579989	-0.425287
N	0.737557	-2.723038	-2.165492	H	-3.178378	5.273864	-0.565799
N	-0.492804	1.802748	-3.455525	C	4.338154	4.693057	3.030803
P	1.384125	-1.397332	-1.551589	C	4.876493	3.963642	2.001157
N	0.709638	-0.086606	-2.068045	C	4.088058	3.588100	0.885891
P	0.477024	1.449842	-2.231197	C	2.725219	4.014730	0.817761
H	-1.101312	-0.976451	-3.465000	C	2.196241	4.745460	1.908274
C	-0.478031	3.238298	-0.680761	C	2.980861	5.071985	2.988198
C	2.521576	2.823524	-1.270739	H	5.687911	2.538663	-0.096383
C	2.480642	-1.016272	0.813423	H	5.915071	3.645941	2.028833
C	3.745548	-2.336291	-1.666087	C	4.636999	2.813064	-0.154429
C	-1.857067	3.586911	-0.719080	C	1.945979	3.651753	-0.327826
C	4.129913	-3.218044	-2.715442	H	1.153460	5.041557	1.892467
C	3.885029	2.413610	-1.242973	H	2.550228	5.627511	3.815984
C	2.062809	-0.068087	1.788633	C	7.132219	-0.310404	1.948713
C	0.873253	7.869776	-0.448609	C	6.191799	-0.970427	1.194681
C	1.226512	6.541868	-0.478919	C	4.808669	-0.742795	1.392440
C	0.237768	5.529819	-0.470927	C	4.415055	0.225918	2.367242
C	-1.139014	5.915394	-0.481683	C	5.408357	0.869190	3.145698
C	-1.467341	7.293594	-0.424656	C	6.739674	0.606062	2.945578

H	8.187576	-0.497702	1.774215
H	6.510353	-1.668832	0.429366
C	3.793143	-1.407621	0.632710
C	3.051575	0.527736	2.548851
H	5.091701	1.589239	3.895211
H	2.777788	1.244592	3.319527
C	6.374618	-5.926493	0.655156
C	6.079841	-5.656189	-0.656664
C	5.312923	-4.516654	-1.009010
C	4.883090	-3.625053	0.022734
C	5.177567	-3.949523	1.368080
C	5.903517	-5.074936	1.676292
H	5.299268	-4.978880	-3.097772
H	6.408590	-6.326988	-1.445912
H	4.813807	-3.302836	2.159912
H	6.106574	-5.316677	2.715199
C	4.946589	-4.275901	-2.347490
C	4.139481	-2.465519	-0.350398
H	-0.748478	9.302391	-0.375743
H	4.951676	4.973362	3.882128
H	7.493010	1.106873	3.546797
H	6.948035	-6.811450	0.913581
C	-2.904701	2.587232	-0.894108
C	-2.865340	1.210787	-0.890553
S	-4.572080	3.165257	-1.138107
C	-4.135959	0.611144	-1.076855
H	-1.967657	0.635667	-0.720584
C	-5.149026	1.546425	-1.213510
C	-4.640082	-0.732124	-1.111813
C	-6.047323	-0.751969	-1.241867
S	-6.742347	0.870528	-1.365876
C	-3.953686	-1.946709	-0.996272
H	-2.874489	-1.964537	-0.878734
C	-4.661628	-3.133406	-0.984487
H	-4.128652	-4.073894	-0.876353
C	-6.769949	-1.937176	-1.219883
H	-7.851880	-1.940688	-1.290591
C	-6.061996	-3.125092	-1.084350
C	-6.788814	-4.434567	-1.013670
F	-6.291606	-5.333426	-1.902099
F	-8.109522	-4.312294	-1.268705
F	-6.669740	-5.004435	0.213190
C	3.678307	-3.031719	-4.090155
C	2.920025	-2.042962	-4.672958
S	4.107852	-4.277305	-5.293504
C	2.656266	-2.267692	-6.044955
H	2.570461	-1.165531	-4.152688
C	3.249621	-3.425051	-6.518489
C	1.907078	-1.591177	-7.062184
S	2.976365	-3.733440	-8.207106

C	1.994026	-2.263218	-8.301616
C	1.158402	-0.413302	-6.978458
C	1.359366	-1.785642	-9.439063
H	1.112650	0.140907	-6.045909
C	0.512135	0.068571	-8.101892
H	1.440770	-2.299925	-10.390679
C	0.612398	-0.618064	-9.322435
H	-0.058812	0.990681	-8.037126
C	-0.172860	-0.121214	-10.497498
F	-1.484810	-0.498509	-10.405936
F	0.283586	-0.607220	-11.672338
F	-0.170084	1.225831	-10.584750
C	0.652426	0.252566	1.981705
C	-0.462918	-0.541912	1.880421
S	0.232136	1.906681	2.463687
C	-1.662847	0.151942	2.192139
H	-0.423674	-1.584174	1.588023
C	-1.435381	1.488322	2.491420
C	-3.061797	-0.173480	2.257305
S	-2.869718	2.422699	2.792320
C	-3.846492	0.969939	2.544156
C	-3.706078	-1.404823	2.096026
C	-5.230311	0.913766	2.617191
H	-3.123402	-2.295564	1.880287
C	-5.084937	-1.476805	2.189856
H	-5.822598	1.800955	2.813724
C	-5.841057	-0.321531	2.429483
H	-5.588023	-2.428217	2.047634
C	-7.332143	-0.432947	2.548517
F	-7.849783	-1.313444	1.657056
F	-7.953374	0.752175	2.346795
F	-7.709221	-0.867280	3.779160
C	4.455791	1.627766	-2.330310
C	4.149762	1.627415	-3.668116
S	5.768671	0.490579	-1.964097
C	4.945300	0.722010	-4.414991
H	3.376742	2.247257	-4.103494
C	5.845939	0.027954	-3.620274
C	5.019395	0.312347	-5.789094
S	6.791468	-1.161375	-4.466107
C	5.967560	-0.723709	-5.969549
C	4.304147	0.774346	-6.898707
C	6.184450	-1.312500	-7.206126
H	3.566114	1.562299	-6.778076
C	4.522671	0.201378	-8.139237
H	6.899603	-2.118547	-7.331433
C	5.446034	-0.842497	-8.287787
H	3.959561	0.546192	-9.000774
C	5.690165	-1.417304	-9.651152
F	4.558181	-1.462463	-10.396566

F	6.186034	-2.675934	-9.598115
F	6.583947	-0.677300	-10.358127
C	1.088578	-4.849184	-0.509939
C	1.270270	-4.891691	0.872083
C	1.866055	-5.701960	-1.295700
C	2.161429	-5.789856	1.450440
C	2.749897	-6.607316	-0.728929
C	2.885350	-6.663617	0.652396
F	0.603695	-4.091562	1.704734
F	2.318155	-5.821094	2.775680
F	3.712355	-7.545471	1.208009
F	3.472832	-7.420668	-1.503107
F	1.784503	-5.673478	-2.627633
C	-0.080332	4.400966	-4.238330
C	1.058362	5.204588	-4.179908
C	-1.315175	4.999339	-3.982119
C	0.958600	6.569882	-3.932726
F	2.286122	4.718715	-4.362955
C	-1.425227	6.359486	-3.737567
F	-2.436827	4.276611	-3.970051
C	-0.284604	7.152120	-3.734047
F	2.057274	7.326628	-3.893113
F	-2.619075	6.909637	-3.503384
F	-0.381630	8.463687	-3.533268
C	-1.167702	-3.592800	-8.134204
C	-0.368119	-4.544034	-7.493876
C	-0.120069	-4.448118	-6.126287
C	-0.692665	-3.428298	-5.389432
C	-1.516581	-2.460669	-6.015645
C	-1.727328	-2.559152	-7.412912
H	-1.355671	-3.666285	-9.200847
H	0.060396	-5.361648	-8.066023
H	0.515901	-5.171917	-5.626054
H	-0.480284	-3.378032	-4.328309
H	-2.359648	-1.847038	-7.929071
C	-2.119315	-1.420857	-5.231981
C	-2.045662	-1.469027	-3.765018
H	-2.026461	-2.482763	-3.363572
H	-2.845455	-0.889949	-3.304421
C	-2.675651	-0.204383	-5.846617
H	-3.040272	-0.396976	-6.860309
H	-1.786812	0.447338	-5.996397
C	-3.693069	0.609896	-5.039274
H	-4.437017	-0.066861	-4.592918
H	-3.183161	1.130124	-4.218886
C	-4.408816	1.634316	-5.916219
H	-4.931256	1.112530	-6.731423
H	-3.664257	2.292181	-6.380833
C	-5.406744	2.464220	-5.113662
H	-5.927231	3.185859	-5.750989

H	-6.161660	1.824549	-4.640212
H	-4.897131	3.021508	-4.320345
Si	-5.022407	-3.554038	-5.441934
H	-4.262115	-2.267351	-5.302377
C	-5.069747	-3.994113	-7.270738
H	-5.719361	-4.862659	-7.432235
H	-5.456767	-3.171704	-7.881709
H	-4.072686	-4.252740	-7.641917
C	-6.751526	-3.197925	-4.791177
H	-6.671278	-3.010533	-3.713667
H	-7.369017	-4.100934	-4.895735
C	-7.425373	-2.001842	-5.478080
H	-8.411923	-1.793978	-5.049480
H	-6.822458	-1.092083	-5.369339
H	-7.565180	-2.176751	-6.550461
C	-4.117114	-4.868570	-4.461143
H	-4.680822	-5.808294	-4.449707
H	-3.129631	-5.075502	-4.889368
H	-3.967354	-4.561112	-3.421256

III’

O	1.627391	2.799077	-2.320630
O	-0.128250	2.325900	-0.541302
O	1.678201	-1.506550	0.525616
O	2.957459	-1.408807	-1.653893
S	-0.489341	-3.195235	-0.947536
S	-0.294525	1.965399	-4.520151
O	-0.915530	-2.882830	0.426039
O	-1.553408	-3.602490	-1.886838
O	1.050537	1.707994	-5.071526
O	-1.439546	1.504273	-5.319753
N	0.358427	-2.037235	-1.664698
N	-0.482652	1.428187	-3.010686
P	1.482523	-1.097998	-1.030342
N	1.362515	0.445124	-1.217752
P	0.584854	1.648048	-1.841852
H	-1.111455	-1.296249	-3.510262
C	-0.503774	3.658185	-0.592816
C	2.460463	3.303085	-1.326626
C	2.741278	-0.883094	1.172582
C	3.662355	-2.525158	-1.242378
C	-1.895351	3.972917	-0.564505
C	3.882704	-3.562803	-2.195797
C	3.801890	2.837007	-1.269423
C	2.454333	0.224081	2.016838
C	0.693014	8.316004	-1.112025
C	1.089264	7.008573	-0.960195
C	0.135493	5.986645	-0.743074
C	-1.249979	6.332964	-0.721203
C	-1.622324	7.693906	-0.856441

C	-0.671698	8.665052	-1.042482
H	1.436598	9.085694	-1.295407
H	2.140767	6.746920	-1.020433
C	0.495397	4.615222	-0.593532
C	-2.219138	5.320739	-0.601547
H	-2.678144	7.951056	-0.834474
H	-3.260442	5.630570	-0.582016
C	4.371033	5.477953	2.779657
C	4.877750	4.645911	1.813196
C	4.070470	4.210080	0.733196
C	2.720263	4.672439	0.641384
C	2.224718	5.515651	1.663566
C	3.029646	5.906934	2.706640
H	5.618977	3.009360	-0.150617
H	5.906410	4.297785	1.862607
C	4.581018	3.322624	-0.235269
C	1.915118	4.207540	-0.444189
H	1.191840	5.844696	1.625997
H	2.627311	6.549049	3.484519
C	7.502776	-0.308037	1.768055
C	6.463505	-1.032379	1.234925
C	5.119896	-0.681049	1.503444
C	4.864951	0.464126	2.321623
C	5.959578	1.179903	2.867045
C	7.251694	0.803482	2.598513
H	8.526592	-0.590971	1.542297
H	6.671033	-1.876940	0.586482
C	4.005183	-1.370619	0.932692
C	3.539615	0.871789	2.576385
H	5.751646	2.040648	3.497767
H	3.379570	1.723046	3.234162
C	6.128938	-6.090664	1.313723
C	5.745660	-5.949342	0.004123
C	5.075693	-4.778702	-0.431480
C	4.833758	-3.726007	0.502810
C	5.221016	-3.913625	1.850468
C	5.851083	-5.069281	2.245104
H	4.846325	-5.468147	-2.438110
H	5.932552	-6.742636	-0.715170
H	5.002208	-3.137878	2.576469
H	6.128402	-5.201319	3.286569
C	4.618728	-4.652481	-1.756194
C	4.173814	-2.546999	0.043651
H	-0.969156	9.702339	-1.162863
H	4.997803	5.803344	3.604779
H	8.082662	1.361697	3.019721
H	6.629660	-6.997087	1.640161
C	-2.934336	2.951974	-0.492718
C	-2.870413	1.579248	-0.499335
S	-4.632178	3.497873	-0.394901

C	-4.142084	0.957364	-0.450248
H	-1.948038	1.022568	-0.536379
C	-5.181889	1.868771	-0.387883
C	-4.618032	-0.391524	-0.484889
C	-6.029661	-0.448489	-0.444014
S	-6.766512	1.156378	-0.358662
C	-3.890237	-1.581997	-0.554260
H	-2.806510	-1.559208	-0.546474
C	-4.558628	-2.790917	-0.602836
H	-3.986721	-3.712878	-0.657044
C	-6.714194	-1.656629	-0.489210
H	-7.797469	-1.694345	-0.461127
C	-5.962833	-2.823042	-0.580474
C	-6.633405	-4.159177	-0.669635
F	-7.979185	-4.071205	-0.696917
F	-6.303241	-4.963718	0.369977
F	-6.256225	-4.826945	-1.799155
C	3.371655	-3.501207	-3.563375
C	2.695064	-2.524223	-4.259111
S	3.655688	-4.923719	-4.602437
C	2.388582	-2.892830	-5.596707
H	2.445506	-1.559463	-3.843627
C	2.850808	-4.160695	-5.912791
C	1.748849	-2.268332	-6.722673
S	2.568299	-4.653551	-7.554928

C	1.784315	-3.098433	-7.866958
C	1.159182	-1.004026	-6.828669
C	1.297477	-2.678915	-9.097398
H	1.127362	-0.336606	-5.973907
C	0.660571	-0.578469	-8.045937
H	1.364654	-3.311187	-9.976023
C	0.743056	-1.406903	-9.175941
H	0.221312	0.411490	-8.124168
C	0.299578	-0.870886	-10.504728
F	1.225002	-0.029246	-11.033051
F	-0.852199	-0.162158	-10.408996
F	0.092972	-1.849242	-11.415430
C	1.082378	0.650589	2.256169
C	-0.077689	-0.084418	2.260326
S	0.772392	2.358201	2.622886
C	-1.221932	0.696209	2.563064
H	-0.109341	-1.147180	2.055743
C	-0.912145	2.033923	2.764432
C	-2.626070	0.443151	2.723101
S	-2.280821	3.048695	3.112933
C	-3.336833	1.632145	3.017064
C	-3.331877	-0.761524	2.639323
C	-4.710783	1.638294	3.206537
H	-2.806742	-1.681605	2.399019
C	-4.700986	-0.767762	2.839655

H	-5.248443	2.556052	3.419635
C	-5.385005	0.424650	3.113072
H	-5.255058	-1.698132	2.764380
C	-6.858969	0.376723	3.384298
F	-7.491703	-0.543134	2.614709
F	-7.463053	1.568024	3.166430
F	-7.121471	0.039424	4.674865
C	4.311766	1.859673	-2.223807
C	3.986367	1.638407	-3.538771
S	5.545547	0.719371	-1.656993
C	4.719550	0.566261	-4.113610
H	3.241693	2.215257	-4.071576
C	5.582068	-0.029420	-3.203514
C	4.792048	-0.064560	-5.403115
S	6.472336	-1.388127	-3.825063
C	5.694366	-1.157423	-5.396305
C	4.132536	0.252136	-6.595243
C	5.917368	-1.934638	-6.522198
H	3.427938	1.078309	-6.618813
C	4.362819	-0.506231	-7.730597
H	6.599455	-2.778128	-6.500437
C	5.239452	-1.598145	-7.690416
H	3.840134	-0.270754	-8.652367
C	5.516778	-2.372768	-8.943745
F	4.437327	-2.431169	-9.760616

F	5.894353	-3.647899	-8.685449
F	6.518807	-1.812298	-9.670840
C	0.639419	-4.626654	-0.825418
C	1.292698	-4.963921	0.362802
C	0.857874	-5.438398	-1.940296
C	2.106234	-6.089331	0.441249
C	1.650632	-6.574018	-1.866481
C	2.279380	-6.897926	-0.671963
F	1.176049	-4.236690	1.470070
F	2.727343	-6.389165	1.581082
F	3.068252	-7.968856	-0.606179
F	1.827205	-7.351324	-2.938816
F	0.318291	-5.150876	-3.126086
C	-0.464770	3.780738	-4.396630
C	0.582670	4.671776	-4.633844
C	-1.704996	4.313500	-4.040700
C	0.389301	6.046120	-4.541700
F	1.810086	4.266857	-4.958501
C	-1.909671	5.682514	-3.953122
F	-2.737347	3.517728	-3.760846
C	-0.860024	6.553187	-4.215706
F	1.402074	6.883863	-4.774663
F	-3.106769	6.167650	-3.613917
F	-1.051402	7.868127	-4.157647
C	-2.968220	-6.352068	-4.672459

C	-1.923113	-6.766037	-5.501077
C	-1.011122	-5.840533	-6.004823
C	-1.120606	-4.506119	-5.658484
C	-2.180368	-4.068487	-4.836581
C	-3.113918	-5.014465	-4.360069
H	-3.666122	-7.079739	-4.270513
H	-1.819275	-7.817838	-5.752190
H	-0.203900	-6.161163	-6.655756
H	-0.413732	-3.788985	-6.062993
H	-3.912910	-4.701947	-3.695851
C	-2.308547	-2.681040	-4.494408
C	-1.157063	-1.789637	-4.493759
H	-1.320243	-0.951389	-5.188966
H	-0.205651	-2.283568	-4.671805
C	-3.614243	-2.129759	-4.085046
H	-3.717670	-2.423303	-3.018761
H	-4.421632	-2.655178	-4.609198
C	-3.782558	-0.612542	-4.190438
H	-3.148314	-0.102432	-3.453878
H	-3.432198	-0.270176	-5.172097
C	-5.236227	-0.188991	-4.001135
H	-5.614399	-0.576597	-3.046823
H	-5.853643	-0.648278	-4.786355
C	-5.388970	1.329110	-4.044806
H	-6.432807	1.628430	-3.908160
H	-4.794901	1.801003	-3.255282
H	-5.038747	1.732526	-5.001667
Si	-3.616504	-1.843015	-8.039376
H	-2.866408	-2.134589	-6.780021
C	-5.401635	-2.377003	-7.771583
H	-5.468826	-3.439703	-7.512643
H	-5.870198	-1.801241	-6.965963
H	-5.999783	-2.216918	-8.675886
C	-2.811209	-2.874239	-9.395656
H	-2.942463	-3.934097	-9.133228
H	-1.728519	-2.694846	-9.370532
C	-3.357817	-2.606354	-10.804820
H	-2.865261	-3.237943	-11.551903
H	-4.433904	-2.805684	-10.863727
H	-3.195218	-1.565404	-11.101812
C	-3.522100	0.003042	-8.358301
H	-4.053846	0.559801	-7.578655
H	-2.485881	0.353539	-8.363332
H	-3.971548	0.265039	-9.322675

IV

O	1.709928	2.414544	-2.354586
O	-0.206572	1.901904	-0.783983
O	1.465082	-1.657736	0.057076
O	2.895602	-1.283668	-2.006208

S	-0.200171	-3.789051	-1.347493
S	-0.123445	2.571271	-4.750831
O	-1.043943	-3.184785	-0.296508
O	-0.887579	-4.658587	-2.318985
O	1.238187	2.247339	-5.216659
O	-1.205983	2.442691	-5.737781
N	0.693164	-2.739805	-2.165606
N	-0.563702	1.784463	-3.418735
P	1.344125	-1.417384	-1.547551
N	0.674484	-0.090866	-2.014074
P	0.421266	1.435448	-2.208604
H	-0.984040	-1.009433	-3.989497
C	-0.528529	3.245511	-0.679771
C	2.467051	2.815577	-1.267005
C	2.459892	-1.048522	0.804486
C	3.702924	-2.367349	-1.687444
C	-1.905505	3.603341	-0.724272
C	4.086337	-3.247482	-2.739515
C	3.828426	2.398975	-1.235598
C	2.050478	-0.093292	1.776215
C	0.848345	7.873557	-0.503842
C	1.193903	6.543450	-0.515079
C	0.198950	5.537357	-0.498426
C	-1.175055	5.931291	-0.520522
C	-1.495634	7.312006	-0.484465
C	-0.506873	8.262422	-0.469153
H	1.625376	8.631391	-0.533385
H	2.239045	6.254049	-0.549022
C	0.505326	4.142736	-0.503687
C	-2.186467	4.955397	-0.606398
H	-2.542235	7.604495	-0.494757
H	-3.216770	5.301629	-0.607602
C	4.288559	4.699715	3.026965
C	4.823892	3.960455	2.002679
C	4.035426	3.584146	0.887543
C	2.675686	4.019679	0.814472
C	2.149851	4.761645	1.898939
C	2.934358	5.089203	2.978681
H	5.630700	2.521517	-0.087461
H	5.860406	3.636266	2.034104
C	4.581167	2.800563	-0.148053
C	1.896311	3.652995	-0.329456
H	1.109203	5.065389	1.878691
H	2.506232	5.653326	3.801977
C	7.119812	-0.349091	1.910931
C	6.173906	-1.008380	1.163276
C	4.792382	-0.775165	1.365917
C	4.406516	0.197229	2.340194
C	5.405439	0.840822	3.111266
C	6.734969	0.572942	2.905770

H	8.173742	-0.540760	1.732532
H	6.486462	-1.710568	0.398934
C	3.770851	-1.438583	0.613206
C	3.044926	0.503583	2.528025
H	5.094331	1.564677	3.859527
H	2.777789	1.224913	3.296870
C	6.335359	-5.963058	0.625607
C	6.033829	-5.693696	-0.684842
C	5.269930	-4.551255	-1.034846
C	4.849388	-3.656859	-0.002345
C	5.150764	-3.979994	1.341967
C	5.874081	-5.107654	1.648124
H	5.245200	-5.015340	-3.121373
H	6.355913	-6.366594	-1.475083
H	4.794379	-3.330463	2.134844
H	6.082827	-5.347991	2.686249
C	4.899160	-4.309037	-2.371348
C	4.106151	-2.496762	-0.373477
H	-0.764869	9.316989	-0.459121
H	4.902230	4.980377	3.878094
H	7.492793	1.074053	3.501105
H	6.906758	-6.849940	0.882064
C	-2.961416	2.607194	-0.872813
C	-2.924007	1.231861	-0.895296
S	-4.638989	3.190506	-1.033617

C	-4.202640	0.637064	-1.036010
H	-2.021660	0.651297	-0.779420
C	-5.220207	1.573773	-1.109956
C	-4.707737	-0.704478	-1.053742
C	-6.121386	-0.724509	-1.104803
S	-6.820723	0.897918	-1.182511
C	-4.013731	-1.918494	-0.974932
H	-2.929624	-1.934214	-0.912002
C	-4.720391	-3.104898	-0.919694
H	-4.182514	-4.043924	-0.821972
C	-6.840716	-1.911248	-1.048550
H	-7.924834	-1.915176	-1.060441
C	-6.125096	-3.098145	-0.949877
C	-6.841383	-4.411186	-0.894429
F	-6.534963	-5.188844	-1.979610
F	-8.183511	-4.278002	-0.882975
F	-6.495227	-5.135849	0.193863
C	3.654012	-3.052934	-4.119478
C	2.860834	-2.091244	-4.702431
S	4.186828	-4.240928	-5.340832
C	2.653946	-2.291375	-6.088278
H	2.448615	-1.247331	-4.172575
C	3.323000	-3.402255	-6.570839
C	1.924190	-1.613456	-7.119238
S	3.146357	-3.662986	-8.280018

C	2.104553	-2.235469	-8.374454	H	-5.813704	1.813202	2.892403
C	1.129465	-0.466449	-7.037205	C	-5.845506	-0.311166	2.519325
C	1.515620	-1.739650	-9.528553	H	-5.604663	-2.420425	2.143651
H	1.005884	0.048368	-6.089780	C	-7.331875	-0.420665	2.682470
C	0.535452	0.037459	-8.179023	F	-7.876263	-1.306615	1.810006
H	1.666496	-2.217443	-10.490425	F	-7.959487	0.762690	2.491987
C	0.726876	-0.599872	-9.414949	F	-7.674748	-0.849393	3.924821
H	-0.069798	0.937397	-8.114351	C	4.397311	1.608496	-2.319708
C	-0.003092	-0.076215	-10.612593	C	4.089874	1.603165	-3.657278
F	-1.325209	-0.423899	-10.576124	S	5.720060	0.483202	-1.952927
F	0.487018	-0.557803	-11.776412	C	4.896344	0.708170	-4.403958
F	0.031031	1.273017	-10.685741	H	3.310899	2.214532	-4.093447
C	0.642671	0.235167	1.974393	C	5.804253	0.023845	-3.609322
C	-0.477724	-0.552239	1.878118	C	4.991620	0.317118	-5.782061
S	0.235076	1.893335	2.453959	S	6.779981	-1.137242	-4.459336
C	-1.671574	0.150106	2.194231	C	5.966829	-0.692339	-5.966864
H	-0.446067	-1.594920	1.586562	C	4.280853	0.781666	-6.893361
C	-1.434366	1.485738	2.489491	C	6.222695	-1.246460	-7.211977
C	-3.070098	-0.169243	2.280517	H	3.517676	1.544528	-6.768435
S	-2.860695	2.426326	2.809626	C	4.537382	0.243080	-8.142011
C	-3.846213	0.977305	2.579652	H	6.962013	-2.029649	-7.341758
C	-3.720817	-1.399704	2.137113	C	5.494245	-0.769193	-8.296994
C	-5.228165	0.924079	2.684512	H	3.979034	0.590801	-9.005437
H	-3.144358	-2.292405	1.912571	C	5.787843	-1.296800	-9.669429
C	-5.096822	-1.468891	2.266372	F	4.676289	-1.357159	-10.443489

F	6.325452	-2.538952	-9.641155
F	6.672816	-0.506838	-10.333760
C	1.016955	-4.862034	-0.509927
C	1.195047	-4.899537	0.872436
C	1.800115	-5.713893	-1.291337
C	2.090217	-5.790296	1.455879
C	2.686289	-6.613862	-0.719043
C	2.820166	-6.663416	0.662647
F	0.520378	-4.101407	1.701181
F	2.243583	-5.816657	2.781966
F	3.648990	-7.540415	1.223677
F	3.410839	-7.430170	-1.488512
F	1.720463	-5.691079	-2.622712
C	-0.109925	4.338862	-4.280690
C	1.045152	5.119409	-4.239640
C	-1.328294	4.968360	-4.022081
C	0.977526	6.488782	-4.002907
F	2.261708	4.607985	-4.430633
C	-1.407896	6.333116	-3.790947
F	-2.466466	4.271638	-3.993219
C	-0.250594	7.100671	-3.800864
F	2.093007	7.222067	-3.977386
F	-2.589285	6.911598	-3.557716
F	-0.317001	8.416284	-3.610100
C	-1.064269	-3.632544	-8.389590
C	-0.258959	-4.590089	-7.777671
C	-0.101705	-4.572497	-6.396416
C	-0.772197	-3.630077	-5.624764
C	-1.612752	-2.682783	-6.226287
C	-1.730207	-2.686140	-7.623832
H	-1.168176	-3.613319	-9.470551
H	0.260227	-5.331389	-8.377606
H	0.549936	-5.289926	-5.905984
H	-0.623667	-3.649576	-4.550862
H	-2.339685	-1.946313	-8.130854
C	-2.414581	-1.728539	-5.385170
C	-1.940741	-1.544474	-3.962953
H	-1.781505	-2.487497	-3.441254
H	-2.639129	-0.936395	-3.386556
C	-2.868532	-0.434659	-6.052224
H	-3.413629	-0.657957	-6.977541
H	-1.952255	0.094630	-6.352131
C	-3.715545	0.483602	-5.170724
H	-4.462295	-0.109591	-4.617875
H	-3.078640	0.967009	-4.421381
C	-4.434976	1.565611	-5.971840
H	-5.038452	1.101937	-6.766906
H	-3.685853	2.193164	-6.469110
C	-5.327878	2.425841	-5.081627
H	-5.825905	3.214574	-5.654179

H	-6.102678	1.818191	-4.596559
H	-4.739852	2.903746	-4.291301
Si	-4.649131	-3.518553	-5.144972
H	-3.512121	-2.287888	-5.225405
C	-4.816702	-3.945293	-6.942822
H	-5.673191	-4.623981	-7.057750
H	-5.013031	-3.062188	-7.558809
H	-3.923386	-4.448918	-7.321863
C	-6.068772	-2.560048	-4.414970
H	-5.685507	-1.974455	-3.569535
H	-6.738089	-3.306984	-3.960194
C	-6.835344	-1.674657	-5.405280
H	-7.628694	-1.123992	-4.890518
H	-6.182426	-0.935248	-5.879940
H	-7.301600	-2.267711	-6.198160
C	-3.790988	-4.765929	-4.090783
H	-4.511455	-5.554408	-3.834931
H	-2.945737	-5.220188	-4.616003
H	-3.420497	-4.341807	-3.153734

IV’

O	1.568145	2.765143	-2.358214
O	-0.153144	2.319340	-0.534959
O	1.615032	-1.506807	0.513823
O	2.850617	-1.451455	-1.691129
S	-0.590838	-3.217736	-0.854384
S	-0.450172	2.001168	-4.504351
O	-0.973934	-2.857719	0.520102
O	-1.674182	-3.655031	-1.751533
O	0.861159	1.712600	-5.118687
O	-1.641588	1.581597	-5.263203
N	0.246802	-2.082148	-1.629848
N	-0.596415	1.461182	-2.998526
P	1.383376	-1.131391	-1.046945
N	1.274643	0.408485	-1.272351
P	0.509909	1.634823	-1.858691
H	-1.101307	-2.005498	-3.553813
C	-0.509781	3.657337	-0.576923
C	2.429502	3.254273	-1.381815
C	2.692288	-0.883344	1.131889
C	3.561795	-2.561259	-1.279599
C	-1.895137	3.994456	-0.518177
C	3.775444	-3.603538	-2.229640
C	3.765390	2.770831	-1.356123
C	2.428438	0.233140	1.972282
C	0.751265	8.296321	-1.119969
C	1.129325	6.982305	-0.978310
C	0.164371	5.975716	-0.739551
C	-1.214274	6.344292	-0.685256
C	-1.567244	7.711407	-0.810796

C	-0.605654	8.667332	-1.018254	H	8.485572	-0.648657	1.419808
H	1.502913	9.053976	-1.319942	H	6.603896	-1.920137	0.496438
H	2.174777	6.703617	-1.063133	C	3.947544	-1.385776	0.878151
C	0.504384	4.598090	-0.600187	C	3.528440	0.873321	2.511801
C	-2.196975	5.347794	-0.546950	H	5.764649	2.022167	3.397897
H	-2.617983	7.985536	-0.763906	H	3.387099	1.729957	3.166922
H	-3.232751	5.673766	-0.508476	C	6.026747	-6.123355	1.282034
C	4.476463	5.409556	2.670915	C	5.630782	-5.990891	-0.024751
C	4.946497	4.569212	1.693300	C	4.968682	-4.817644	-0.465109
C	4.104612	4.140089	0.637362	C	4.747572	-3.753326	0.461302
C	2.758452	4.618888	0.579940	C	5.148556	-3.931250	1.806321
C	2.300938	5.469585	1.613855	C	5.770607	-5.089512	2.205825
C	3.138482	5.853552	2.633625	H	4.720652	-5.519359	-2.465656
H	5.613164	2.917395	-0.286136	H	5.801022	-6.793589	-0.737678
H	5.971623	4.208291	1.715802	H	4.945619	-3.145879	2.526553
C	4.577567	3.244394	-0.342309	H	6.057566	-5.214134	3.245608
C	1.919696	4.166261	-0.485363	C	4.503759	-4.697264	-1.787851
H	1.271355	5.810561	1.603619	C	4.091175	-2.573500	-0.000362
H	2.764628	6.501437	3.420886	H	-0.889022	9.709481	-1.130641
C	7.467927	-0.353403	1.657467	H	5.129610	5.729775	3.477414
C	6.413809	-1.069536	1.142354	H	8.081898	1.315779	2.893667
C	5.077591	-0.702792	1.426746	H	6.520620	-7.032142	1.612307
C	4.845692	0.449930	2.241311	C	-2.949869	2.991972	-0.427154
C	5.955137	1.156110	2.769011	C	-2.915518	1.619428	-0.484088
C	7.239626	0.764653	2.485657	S	-4.630322	3.567071	-0.241271

C	-4.196431	1.021019	-0.406399
H	-2.009500	1.044537	-0.583385
C	-5.213348	1.949488	-0.269414
C	-4.699739	-0.318156	-0.464637
C	-6.108591	-0.349189	-0.358148
S	-6.809098	1.266812	-0.190358
C	-3.999658	-1.519969	-0.601842
H	-2.917041	-1.517616	-0.660964
C	-4.693076	-2.713874	-0.640169
H	-4.142980	-3.644473	-0.744315
C	-6.818133	-1.542751	-0.392471
H	-7.898891	-1.561292	-0.307599
C	-6.094310	-2.720660	-0.543023
C	-6.795625	-4.042729	-0.605321
F	-8.141066	-3.923318	-0.604689
F	-6.464965	-4.842125	0.439260
F	-6.459010	-4.731956	-1.731630
C	3.281999	-3.529360	-3.603530
C	2.627030	-2.539774	-4.300967
S	3.600813	-4.932301	-4.660120
C	2.376790	-2.877867	-5.658132
H	2.357578	-1.585240	-3.874663
C	2.852845	-4.137363	-5.984752
C	1.811499	-2.215107	-6.800279
S	2.666224	-4.579329	-7.655627
C	1.915744	-3.009676	-7.967065
C	1.237196	-0.942574	-6.903517
C	1.514880	-2.545912	-9.212712
H	1.147863	-0.302980	-6.031078
C	0.826426	-0.473150	-8.137793
H	1.638200	-3.150129	-10.104887
C	0.976667	-1.265892	-9.287131
H	0.408142	0.526177	-8.213919
C	0.622563	-0.688993	-10.622901
F	1.578934	0.156729	-11.077736
F	-0.531710	0.033031	-10.575530
F	0.452316	-1.638865	-11.570868
C	1.063880	0.669876	2.232097
C	-0.102419	-0.055404	2.240491
S	0.771767	2.374806	2.628549
C	-1.236100	0.729947	2.566339
H	-0.147412	-1.115280	2.024850
C	-0.913756	2.061352	2.786179
C	-2.639452	0.482903	2.740509
S	-2.269890	3.078015	3.175823
C	-3.336626	1.669322	3.073322
C	-3.354253	-0.714883	2.636832
C	-4.705845	1.678333	3.293572
H	-2.839686	-1.630597	2.359465
C	-4.718983	-0.718055	2.865733

H	-5.233301	2.593485	3.540705	F	5.969074	-3.452888	-8.979088
C	-5.388747	0.470473	3.187793	F	6.555638	-1.559571	-9.875022
H	-5.280450	-1.642783	2.777129	C	0.547207	-4.641132	-0.731522
C	-6.854469	0.424079	3.499877	C	1.197735	-4.990663	0.453235
F	-7.514484	-0.480170	2.735264	C	0.769831	-5.435421	-1.858463
F	-7.459483	1.621653	3.319671	C	2.008580	-6.119247	0.520253
F	-7.081265	0.067568	4.792588	C	1.561817	-6.572045	-1.796259
C	4.244888	1.794069	-2.327722	C	2.181643	-6.914812	-0.602146
C	3.923842	1.613921	-3.649759	F	1.082466	-4.272170	1.567026
S	5.445109	0.611172	-1.779381	F	2.629508	-6.433552	1.657355
C	4.641512	0.542611	-4.247571	F	2.969423	-7.988378	-0.547840
H	3.194629	2.222322	-4.169846	F	1.754508	-7.328586	-2.881672
C	5.477095	-0.099691	-3.343263	F	0.243938	-5.121257	-3.041057
C	4.740063	-0.036541	-5.559507	C	-0.571867	3.820233	-4.371121
S	6.352310	-1.454494	-3.993343	C	0.490506	4.685316	-4.634688
C	5.620432	-1.147275	-5.572426	C	-1.790036	4.383210	-3.985403
C	4.139793	0.354242	-6.761588	C	0.333222	6.064160	-4.541794
C	5.869897	-1.876072	-6.725362	F	1.699099	4.249652	-4.990778
H	3.459705	1.200951	-6.774006	C	-1.958767	5.757305	-3.897341
C	4.404150	-0.349194	-7.924059	F	-2.834827	3.614421	-3.678181
H	6.533466	-2.734342	-6.718071	C	-0.895406	6.602064	-4.188167
C	5.250985	-1.465491	-7.902033	F	1.360148	6.875903	-4.804446
H	3.934463	-0.050197	-8.856012	F	-3.135490	6.272399	-3.531727
C	5.562942	-2.177868	-9.183429	F	-1.054120	7.921597	-4.131178
F	4.492520	-2.227481	-10.015880	C	-2.706598	-6.479698	-4.465855

C	-1.607823	-6.930985	-5.188161
C	-0.800937	-6.015711	-5.859887
C	-1.089488	-4.660782	-5.797192
C	-2.197746	-4.193913	-5.077387
C	-3.008790	-5.123194	-4.418205
H	-3.331962	-7.184222	-3.925751
H	-1.373592	-7.991011	-5.220487
H	0.066465	-6.353621	-6.419276
H	-0.440135	-3.959990	-6.315526
H	-3.855175	-4.793865	-3.825931
C	-2.465904	-2.720482	-4.986242
C	-1.267719	-1.877888	-4.629159
H	-1.437984	-0.817618	-4.825782
H	-0.362798	-2.208342	-5.138569
C	-3.747220	-2.296434	-4.296451
H	-3.648049	-2.614936	-3.248879
H	-4.597933	-2.852798	-4.711501
C	-4.025869	-0.794642	-4.337374
H	-3.340981	-0.263361	-3.664651
H	-3.808768	-0.390776	-5.338013
C	-5.466898	-0.454890	-3.967882
H	-5.708300	-0.910518	-2.999914
H	-6.152985	-0.911474	-4.697110
C	-5.698002	1.052535	-3.906973
H	-6.731436	1.288979	-3.635837
H	-5.039781	1.514222	-3.162952
H	-5.479467	1.524973	-4.871873
Si	-3.189257	-1.984213	-7.706788
H	-2.669092	-2.390630	-6.170283
C	-4.978090	-2.470564	-7.588794
H	-5.090227	-3.510151	-7.263671
H	-5.528699	-1.819984	-6.903033
H	-5.439397	-2.377770	-8.580877
C	-2.120460	-3.152263	-8.682833
H	-2.268759	-4.175930	-8.318503
H	-1.066771	-2.901861	-8.510329
C	-2.447725	-3.055914	-10.188028
H	-1.778518	-3.706550	-10.759262
H	-3.474879	-3.370352	-10.400819
H	-2.314176	-2.039696	-10.572477
C	-2.823792	-0.176321	-7.872551
H	-3.723959	0.426015	-7.716372
H	-2.076231	0.159942	-7.144746
H	-2.425006	0.023894	-8.873908

2a

C	-1.121043	-3.204043	-7.916284
C	-1.807323	-4.378768	-7.618057
C	-2.407910	-4.521093	-6.372229
C	-2.322021	-3.495466	-5.434042

C	-1.637698	-2.313415	-5.719179
C	-1.037564	-2.183799	-6.975747
H	-0.648193	-3.082583	-8.887001
H	-1.872665	-5.176042	-8.352713
H	-2.946860	-5.432417	-6.128295
H	-2.797932	-3.611915	-4.462781
H	-0.498977	-1.272978	-7.226045
C	-1.551223	-1.199244	-4.696235
C	-2.228208	0.089484	-5.193731
H	-1.691628	0.459676	-6.079522
H	-2.112406	0.862682	-4.420520
C	-3.708106	-0.080812	-5.526915
H	-3.821803	-0.849936	-6.303272
H	-4.239290	-0.459916	-4.640911
C	-4.367492	1.215417	-5.995827
H	-3.834407	1.590384	-6.880443
H	-4.251812	1.984929	-5.219762
C	-5.847960	1.036898	-6.325321
H	-6.302471	1.974902	-6.660183
H	-5.985855	0.294856	-7.119779
H	-6.405582	0.689162	-5.448242
H	-2.098863	-1.534964	-3.803213
C	-0.097514	-0.930602	-4.285599
H	0.385289	-1.844924	-3.926671
H	0.486313	-0.554163	-5.132911
H	-0.053483	-0.179325	-3.489454

[Si]-X*

O	2.398726	2.899675	-1.792718
O	0.377318	2.338156	-0.335101
O	1.965256	-1.499493	0.628647
O	3.627547	-1.253137	-1.281579
S	0.452579	-2.137416	-3.062246
S	0.777237	2.612219	-4.227123
O	-1.005958	-2.320199	-2.994995
O	0.955803	-0.998010	-3.863451
O	2.062497	2.258763	-4.821019
O	-0.371291	2.477028	-5.265087
N	1.082390	-2.205605	-1.585578
N	0.304126	1.915265	-2.916261
P	2.036100	-1.113362	-0.944372
N	1.802118	0.426282	-1.146657
P	1.238354	1.781662	-1.589284
C	0.063115	3.692250	-0.286516
C	3.103307	3.263167	-0.641537
C	2.876140	-0.861259	1.469540
C	4.359489	-2.329690	-0.800152
C	-1.307010	4.074234	-0.397147
C	4.907808	-3.254528	-1.737720
C	4.420847	2.760744	-0.461020

C	2.397111	0.164526	2.333711
C	1.506686	8.306943	-0.116316
C	1.827077	6.971175	-0.068748
C	0.812691	5.984613	-0.094585
C	-0.547988	6.401070	-0.210168
C	-0.845185	7.787645	-0.232214
C	0.159182	8.720135	-0.180710
H	2.297682	9.050662	-0.109704
H	2.866131	6.661754	-0.020773
C	1.092041	4.586581	-0.052705
C	-1.564287	5.436759	-0.327624
H	-1.883969	8.097332	-0.310754
H	-2.585943	5.798890	-0.400988
C	4.521326	5.059366	3.832029
C	5.135328	4.305034	2.864512
C	4.453024	3.957910	1.672188
C	3.123655	4.439954	1.465432
C	2.511516	5.195971	2.492907
C	3.192489	5.494510	3.648641
H	6.087302	2.811491	0.882935
H	6.152016	3.946994	3.001301
C	5.068016	3.143887	0.701503
C	2.460538	4.096758	0.245880
H	1.488483	5.533135	2.367160
H	2.701904	6.069917	4.427981
C	7.418489	-0.199275	3.012713
C	6.534807	-0.882752	2.212829
C	5.147377	-0.606024	2.259635
C	4.692769	0.431009	3.129583
C	5.625787	1.099503	3.960597
C	6.961213	0.790631	3.907251
H	8.479317	-0.424075	2.954634
H	6.902943	-1.637381	1.527128
C	4.186868	-1.285804	1.444832
C	3.328660	0.774939	3.154951
H	5.260139	1.871273	4.632513
H	3.007235	1.542197	3.855152
C	6.586797	-5.844717	2.034251
C	6.468599	-5.644009	0.682597
C	5.792565	-4.505817	0.175887
C	5.257657	-3.548869	1.089809
C	5.376299	-3.796997	2.477670
C	6.023517	-4.918804	2.937130
H	6.076430	-5.048860	-1.868289
H	6.878704	-6.363091	-0.021773
H	4.938922	-3.096847	3.181490
H	6.095040	-5.098401	4.005682
C	5.632606	-4.309711	-1.206756
C	4.596687	-2.400404	0.559434
H	-0.078561	9.779081	-0.212147

H	5.052611	5.316729	4.743697
H	7.669189	1.309694	4.546966
H	7.099419	-6.723988	2.412636
C	-2.393970	3.117647	-0.565481
C	-2.400118	1.750064	-0.715431
S	-4.061151	3.757472	-0.616981
C	-3.696020	1.205664	-0.898071
H	-1.508921	1.142014	-0.702991
C	-4.687030	2.171382	-0.857708
C	-4.230281	-0.099599	-1.163286
C	-5.638001	-0.066815	-1.275788
S	-6.299157	1.566766	-1.090123
C	-3.564582	-1.318557	-1.328293
H	-2.481132	-1.367780	-1.292705
C	-4.295520	-2.465768	-1.571003
H	-3.780276	-3.409581	-1.718292
C	-6.384718	-1.215305	-1.501127
H	-7.466584	-1.184260	-1.564190
C	-5.696525	-2.414104	-1.643407
C	-6.450392	-3.694998	-1.847856
F	-5.912942	-4.443082	-2.840469
F	-7.749645	-3.488202	-2.157825
F	-6.429919	-4.467941	-0.729325
C	4.720225	-3.126221	-3.176622
C	4.044234	-2.194708	-3.927518
S	5.418302	-4.392071	-4.228592
C	4.032350	-2.488509	-5.313652
H	3.555385	-1.325327	-3.517902
C	4.731199	-3.643126	-5.620047
C	3.453998	-1.885712	-6.478470
S	4.731174	-4.061598	-7.307678
C	3.754667	-2.622943	-7.645566
C	2.716581	-0.701746	-6.590969
C	3.343819	-2.204882	-8.904931
H	2.477340	-0.128273	-5.702350
C	2.319557	-0.269117	-7.839954
H	3.585491	-2.771551	-9.797019
C	2.627001	-1.018302	-8.986763
H	1.765038	0.658693	-7.938428
C	2.195112	-0.482876	-10.318118
F	2.351815	-1.376557	-11.316926
F	2.901671	0.627158	-10.662951
F	0.888259	-0.111922	-10.310793
C	0.993322	0.552864	2.381726
C	-0.146671	-0.175030	2.143860
S	0.606673	2.205010	2.904389
C	-1.336615	0.561599	2.386793
H	-0.124275	-1.210214	1.828489
C	-1.075548	1.865925	2.781802
C	-2.747939	0.294345	2.340517

S	-2.487974	2.836935	3.071102
C	-3.504259	1.443267	2.677535
C	-3.427675	-0.889560	2.035475
C	-4.890937	1.432648	2.691603
H	-2.871709	-1.782972	1.767859
C	-4.810701	-0.914721	2.057333
H	-5.460791	2.321858	2.939018
C	-5.535510	0.241693	2.374997
H	-5.339576	-1.828586	1.805702
C	-7.032714	0.171395	2.443213
F	-7.456476	-0.330847	3.632490
F	-7.549693	-0.631669	1.482385
F	-7.611874	1.386675	2.307055
C	5.062005	1.885985	-1.434310
C	4.918302	1.803303	-2.798207
S	6.281397	0.741351	-0.841590
C	5.786465	0.847442	-3.388689
H	4.226468	2.416860	-3.360195
C	6.565282	0.190729	-2.445696
C	6.076004	0.395119	-4.722228
S	7.640898	-1.009806	-3.095729
C	7.059485	-0.623900	-4.721395
C	5.548494	0.817149	-5.947058
C	7.496610	-1.225552	-5.892041
H	4.785669	1.589908	-5.970618

C	5.986081	0.231802	-7.121950
H	8.242460	-2.012973	-5.878810
C	6.947726	-0.786861	-7.092332
H	5.565456	0.545346	-8.072417
C	7.447603	-1.356176	-8.387316
F	6.455333	-1.509352	-9.296473
F	8.036431	-2.563189	-8.226768
F	8.372798	-0.543303	-8.963186
C	1.074647	-3.641167	-3.894489
C	1.793296	-4.674082	-3.294000
C	0.805542	-3.751857	-5.260507
C	2.246188	-5.759007	-4.038354
C	1.260902	-4.821850	-6.015176
C	1.990088	-5.830203	-5.399202
F	2.089770	-4.687007	-1.995240
F	2.936783	-6.734165	-3.442837
F	2.439415	-6.861793	-6.109945
F	1.001127	-4.887777	-7.321844
F	0.089915	-2.819622	-5.892840
C	0.726609	4.401582	-3.967709
C	1.880197	5.187847	-3.957133
C	-0.500213	5.021274	-3.714235
C	1.801495	6.558957	-3.739608
F	3.090513	4.673059	-4.151509
C	-0.587578	6.390383	-3.516932

F	-1.629751	4.313985	-3.664911
C	0.567677	7.163138	-3.539258
F	2.908301	7.298680	-3.733376
F	-1.770722	6.965560	-3.310248
F	0.493033	8.476507	-3.370545
Si	-1.729991	1.411625	-5.625852
C	-1.048397	0.067414	-6.702531
H	-1.806144	-0.710254	-6.853687
H	-0.741234	0.436651	-7.686266
H	-0.191349	-0.410883	-6.219418
C	-2.489874	0.796559	-4.058669
H	-1.839597	0.080991	-3.547794
H	-2.724646	1.618730	-3.377229
H	-3.425555	0.275162	-4.296421
C	-2.832071	2.602298	-6.556964
H	-2.242980	3.081344	-7.350534
H	-3.150484	3.404218	-5.877840
C	-4.059286	1.899086	-7.161828
H	-4.685561	1.444442	-6.386655
H	-4.686596	2.604808	-7.715518
H	-3.764993	1.106333	-7.858045

TS1

O	1.755447654061	2.580668615224	-2.213786520090
O	-0.117809920925	2.014908291765	-0.606201777887
O	1.505186498409	-1.641771581281	0.153916373881
O	2.912471542398	-1.275690314595	-1.918751321997
S	-0.311519337708	-3.642575874017	-1.264712058686
S	-0.072954968330	2.815506935377	-4.576157185130
O	-1.084056357308	-3.016926558256	-0.173826940665
O	-1.050767943688	-4.452069443515	-2.238607571681
O	1.280358990054	2.561681964554	-5.079389843882
O	-1.198135421637	2.678861332048	-5.505395953500
N	0.597363833035	-2.588777872665	-2.086260746031
N	-0.458931119848	1.862386405693	-3.275718764865
P	1.369160779020	-1.361171876643	-1.436964178865
N	0.797582880265	0.040534383447	-1.843292180879
P	0.535670115546	1.547694415817	-2.007261409880
H	-1.284621081454	0.825868498191	-3.605743507227
C	-0.419187524380	3.360097707677	-0.435382546385
C	2.559686871306	2.928267858019	-1.123829329414
C	2.522916991692	-1.061790517724	0.897961886345
C	3.691987771486	-2.387937499647	-1.623083697570
C	-1.793914511580	3.727506956955	-0.397025210554
C	4.029437849876	-3.266810015131	-2.691024963376
C	3.902846928772	2.461892442993	-1.130308795246
C	2.145457854312	-0.105180607215	1.881741733071
C	1.009749982411	7.961512268355	-0.091472493280
C	1.341132636484	6.630833458696	-0.184098028123
C	0.337613887332	5.633523389092	-0.169315367073
C	-1.031902878358	6.037301311824	-0.102491223922
C	-1.336735665685	7.417080791446	0.018051774014
C	-0.338568438220	8.357865140529	0.029990129190
H	1.791776209184	8.714120930745	-0.120688308126

H	2.380741002893	6.335362056704	-0.281691791623
C	0.629802940595	4.239041109709	-0.256509364988
C	-2.054366491620	5.073587588452	-0.187203850447
H	-2.379549622248	7.717251982538	0.076886575818
H	-3.080770619930	5.422955451925	-0.113219220947
C	4.498928549569	4.597262316331	3.201681560860
C	4.998236253515	3.888745402047	2.138699000821
C	4.178293674241	3.571588425854	1.027730139632
C	2.827290195321	4.037597818382	1.000455481138
C	2.337424927373	4.742856487690	2.125376669119
C	3.151247690498	5.011587098489	3.199485324688
H	5.723969917668	2.495106122670	-0.007884606516
H	6.028193385479	3.543288040563	2.135417879309
C	4.683416552444	2.808978205650	-0.042533344906
C	2.020160992059	3.732834242828	-0.142636196462
H	1.302453477784	5.064717768668	2.142163987452
H	2.751326881854	5.548335062886	4.054543041380
C	7.211220483607	-0.488331143015	1.944224950895
C	6.239251301348	-1.118424056492	1.204819158265
C	4.867195546783	-0.852239793517	1.428325183119
C	4.517137386698	0.121628519490	2.414957439412
C	5.542537475248	0.733551350532	3.177501370669
C	6.862159961449	0.434877953056	2.951242288709
H	8.257628453894	-0.705106828093	1.751195153165
H	6.523036370857	-1.824406966525	0.432456025955
C	3.819739906859	-1.483854485475	0.684733898685
C	3.165986286095	0.459209138998	2.625132924333
H	5.259306410453	1.456565501237	3.937518863064
H	2.929176173903	1.178009414837	3.406326385925
C	6.239076045240	-6.089702997649	0.609714835736
C	5.928015937701	-5.795913144186	-0.693227784478
C	5.197804054423	-4.624981017230	-1.019625253011
C	4.824387039185	-3.727888511094	0.029296573553

C	5.134844099106	-4.075266242105	1.365175301911
C	5.822645182809	-5.230875938275	1.648237113301
H	5.129017019511	-5.063133522656	-3.112176847536
H	6.215114203043	-6.471077904019	-1.494882234291
H	4.812277000621	-3.423294912363	2.170452593186
H	6.037533748933	-5.490435043580	2.680403466129
C	4.814971210433	-4.356830176711	-2.348094697086
C	4.111807683118	-2.541183044241	-0.317374349112
H	-0.585081502843	9.412502155393	0.106038675170
H	5.135510258069	4.832492398580	4.049670298414
H	7.640004645657	0.911154766687	3.541079296100
H	6.782600528521	-6.998849412383	0.848018332172
C	-2.867580319919	2.760272310390	-0.589426232748
C	-2.858658344090	1.385649407242	-0.675227215803
S	-4.523703576414	3.387674249882	-0.784310741736
C	-4.138792203978	0.827214059827	-0.918380910172
H	-1.973156360892	0.781178623886	-0.543739904806
C	-5.131867211378	1.790997962944	-0.985190974922
C	-4.652808656239	-0.490568203189	-1.168476762243
C	-6.047268099092	-0.465504741913	-1.385895213470
S	-6.722588031895	1.169022203717	-1.293446023676
C	-3.979339871630	-1.716181466787	-1.232406558347
H	-2.910082257892	-1.769998397043	-1.054198346858
C	-4.687461412246	-2.872407755208	-1.496207916163
H	-4.161324833421	-3.821028917580	-1.551401680653
C	-6.773884820476	-1.622606241709	-1.636581920271
H	-7.847508958888	-1.595275611239	-1.784816937573
C	-6.077444856229	-2.822998052451	-1.691400453515
C	-6.808542865353	-4.110432804234	-1.937181969948
F	-6.236207438934	-4.828425190061	-2.932394893843
F	-8.102813323897	-3.914975236799	-2.275471824834
F	-6.802241242062	-4.903628514380	-0.835005562288
C	3.578804256823	-3.036776392844	-4.058820053904

C	2.862085215802	-2.004635626179	-4.620009461408
S	3.977723107466	-4.259501908821	-5.294668003760
C	2.618117590057	-2.172672346703	-6.002644114304
H	2.531570220887	-1.132969892256	-4.078576202035
C	3.171966309120	-3.336622912306	-6.504830195850
C	1.952815803796	-1.410714502642	-7.016352126404
S	2.948123691519	-3.558355667252	-8.215287001524
C	2.062410913750	-2.026073504190	-8.282505158762
C	1.276475139793	-0.193859913782	-6.906897436321
C	1.534094231491	-1.443846024615	-9.426063042870
H	1.194430980329	0.298230903678	-5.943744359249
C	0.742604588396	0.395892109374	-8.036350715353
H	1.635855269863	-1.911931095023	-10.399009175933
C	0.876897009031	-0.226028771433	-9.287382150467
H	0.223542345059	1.346232495290	-7.952740657047
C	0.227179642444	0.413127126215	-10.476020983462
F	0.671031606322	-0.090637198410	-11.647916008780
F	0.429199800163	1.751239502374	-10.508245800165
F	-1.125529514925	0.231134278120	-10.458939166805
C	0.747792087371	0.250779789726	2.098590844896
C	-0.394773643832	-0.495882824096	1.944441490966
S	0.379111092470	1.884620631524	2.688672811345
C	-1.571199825713	0.217705413438	2.292471888005
H	-0.394425647972	-1.519599893446	1.591928087583
C	-1.302535696621	1.519686374818	2.689452175476
C	-2.981344688263	-0.060470370094	2.299392450463
S	-2.708979659306	2.473100604102	3.056655430621
C	-3.731706693834	1.080963702213	2.672435241227
C	-3.661773466745	-1.241460943206	1.986327211103
C	-5.117824374028	1.072211917910	2.700552297681
H	-3.104935895863	-2.125031273962	1.687607446316
C	-5.045302663773	-1.264128559616	2.024162814228
H	-5.684428831898	1.958576670353	2.965369575303

C	-5.766273713447	-0.111997808657	2.364084760421
H	-5.577805395425	-2.171708326825	1.758068255392
C	-7.264272171077	-0.171766246242	2.424687807314
F	-7.780477455233	-0.989985978868	1.477330035306
F	-7.832567537714	1.045928988277	2.258201987803
F	-7.701109970802	-0.643707479873	3.621586895188
C	4.433827420877	1.667138663052	-2.230626940001
C	4.140361132509	1.702204482243	-3.571609356984
S	5.695761915118	0.473769361231	-1.872154924431
C	4.913079527420	0.784313802719	-4.328947471178
H	3.399435876938	2.364065139676	-4.000972959418
C	5.774980650257	0.040599286103	-3.534834124032
C	5.008805607263	0.409185376349	-5.712816340258
S	6.695171973389	-1.161081990416	-4.387573551902
C	5.924753018646	-0.654460310654	-5.897249476593
C	4.348575093055	0.929641136459	-6.830524208222
C	6.159982900705	-1.214470464234	-7.143797235661
H	3.637228247363	1.741520032032	-6.711156355564
C	4.586036782390	0.387029105711	-8.081086005956
H	6.849598197013	-2.041959167391	-7.271506813506
C	5.475513370413	-0.685119919375	-8.232627211729
H	4.059484917194	0.774652005092	-8.947493795256
C	5.744004843514	-1.226554424117	-9.605732937560
F	4.630659334117	-1.232153042460	-10.378365169445
F	6.218920920360	-2.493050391945	-9.576072614509
F	6.666619436104	-0.479982789883	-10.267757058690
C	0.899405000026	-4.766234217809	-0.488635621381
C	1.099557537283	-4.858736017106	0.888442705624
C	1.641026817907	-5.613474444226	-1.314469719883
C	1.977272866220	-5.796297012420	1.423043278953
C	2.511335686794	-6.557564804172	-0.790782236747
C	2.667549625355	-6.660436301089	0.585677193770
F	0.467419479310	-4.069470644305	1.757651861614

F	2.154023278315	-5.873834497282	2.744083035779
F	3.482204111057	-7.577947037864	1.100123294067
F	3.202653020582	-7.362605765312	-1.601151392748
F	1.537961557266	-5.539496491764	-2.641615231162
C	-0.102762152657	4.517412747405	-3.950868218808
C	1.033454574607	5.330224122092	-3.907977840910
C	-1.334754994110	5.094460516314	-3.633577524936
C	0.927455416989	6.685770811384	-3.616788320058
F	2.256124757144	4.863525687877	-4.146537500704
C	-1.447895194584	6.444677001157	-3.341660876091
F	-2.446359078149	4.360082221297	-3.609776385251
C	-0.314631874571	7.248449070908	-3.357741521061
F	2.017932945207	7.451443113371	-3.593990305710
F	-2.635841114272	6.973006075495	-3.047773147882
F	-0.416397990193	8.549962325630	-3.116340828143
C	-0.639076645457	-3.632539658554	-5.898938302275
C	-0.759853032746	-3.625541177842	-7.285629343687
C	-1.409998601941	-2.568839501380	-7.922056333312
C	-1.935689756201	-1.525491914472	-7.179819178157
C	-1.829239434825	-1.517152007494	-5.774054075131
C	-1.170907865856	-2.594969738156	-5.151070384016
H	-0.145114603915	-4.452796536494	-5.386695808093
H	-0.349539310287	-4.442748019781	-7.872095874633
H	-1.494355971719	-2.551478641509	-9.004283993096
H	-2.412948936862	-0.703759596057	-7.702417691775
H	-1.100235735585	-2.658899106715	-4.073135167333
C	-2.441144346833	-0.460257625669	-4.975779544121
C	-2.108734901192	-0.256757480908	-3.644877013831
H	-1.490580963371	-0.993376006611	-3.135525553376
H	-2.862041981915	0.229214754395	-3.028802723505
C	-3.432127939473	0.449841407965	-5.635468632015
H	-4.148377661249	-0.169632536305	-6.193434486350
H	-2.879719589651	1.017599832615	-6.400819836762

C	-4.178953172195	1.438407017511	-4.744898123102
H	-4.828066867937	0.895018049759	-4.042127235683
H	-3.467575086433	2.018207601211	-4.144915708618
C	-5.018758396928	2.411442657213	-5.571963676707
H	-5.709610287563	1.848675211697	-6.215280975943
H	-4.350988359346	2.968992217567	-6.243007462482
C	-5.805276116726	3.387920511447	-4.701458337272
H	-6.386168021697	4.087062881239	-5.311338834385
H	-6.502138698770	2.853796439266	-4.045396663179
H	-5.131955593722	3.973696865904	-4.065973255186

TS2

O	1.749022678943	2.416589345230	-2.373414763778
O	-0.173108065393	1.899303481112	-0.813927732462
O	1.466408028964	-1.632753707188	0.046828698853
O	2.918850918277	-1.267191537716	-2.000061134039
S	-0.169768082759	-3.790779627991	-1.354931369579
S	-0.075038201660	2.626978025122	-4.764496071284
O	-1.005704030057	-3.190204190392	-0.297457064637
O	-0.853018256699	-4.656063417157	-2.330230888074
O	1.291190835137	2.331378001052	-5.234699773231
O	-1.151353552141	2.505956593670	-5.763133708706
N	0.714020313787	-2.729496012505	-2.177343628461
N	-0.515504286544	1.793290851213	-3.461215336898
P	1.362866195137	-1.406549182075	-1.559151602069
N	0.692482440632	-0.090431234228	-2.063301179548
P	0.456278047480	1.443266535580	-2.238398517829
H	-1.060411750052	-1.061156688651	-3.572534423411
C	-0.495240436856	3.242779049787	-0.696831484248
C	2.502506298128	2.814966920452	-1.280946104415
C	2.458662515127	-1.030991335460	0.804792030571
C	3.723955859761	-2.350651108727	-1.675946850669
C	-1.872603344700	3.598146802740	-0.736637282355

C	4.113712326404	-3.230107973202	-2.725602743930
C	3.864208716671	2.399783891876	-1.248557939431
C	2.042152987216	-0.080575528321	1.778188135579
C	0.876075034427	7.869630319943	-0.480083573110
C	1.223333738831	6.540019171526	-0.504600721798
C	0.229871689439	5.532621841214	-0.494669174202
C	-1.144945386817	5.924572076892	-0.509319285111
C	-1.467171022727	7.304406921367	-0.458420648600
C	-0.479545641649	8.256030509758	-0.437111482490
H	1.651933886789	8.628796547442	-0.504875564255
H	2.268821348259	6.252374833929	-0.544196006436
C	0.538138326767	4.138325307090	-0.513351500935
C	-2.155517207138	4.948353436831	-0.602059103628
H	-2.514210498833	7.595528838924	-0.462217455044
H	-3.186830000030	5.291396951898	-0.592808521393
C	4.314083898952	4.681684023980	3.024520390398
C	4.852525063667	3.948590547718	1.997508841650
C	4.066189844837	3.575661123850	0.879874624146
C	2.705770105373	4.008815159119	0.806625242842
C	2.176457298568	4.743473933771	1.894348117002
C	2.958806274858	5.067261817287	2.976748503607
H	5.664306889419	2.518790216328	-0.096918904017
H	5.889595977089	3.626335140817	2.028918346343
C	4.614624627238	2.797153225700	-0.158056183178
C	1.928825970692	3.647635877608	-0.340916498145
H	1.135154959634	5.044618684298	1.874482169079
H	2.528010977878	5.625754362923	3.802457696292
C	7.110682515312	-0.333593374740	1.943789140841
C	6.169715928536	-0.991464622215	1.188591861978
C	4.786862230646	-0.760388304738	1.384385466626
C	4.394342710540	0.209572537448	2.358398588881
C	5.388128982340	0.850737324311	3.137957302302
C	6.719064208459	0.584185422672	2.939825541843

H	8.165811134357	-0.523586220795	1.770830391211
H	6.487549413300	-1.691026873464	0.424037157508
C	3.770642277267	-1.423444486089	0.624107687951
C	3.031317876883	0.514546047806	2.538323820616
H	5.072149730664	1.571647859010	3.886940676096
H	2.758104903590	1.232731236206	3.307956870188
C	6.344105029915	-5.945223416983	0.650095541477
C	6.053275817243	-5.673629958682	-0.662337534338
C	5.289045415169	-4.532667208044	-1.015993622808
C	4.857162026611	-3.641616275193	0.015101469663
C	5.147731602075	-3.967165765385	1.361069624229
C	5.871333873904	-5.093743204903	1.670562034135
H	5.282514658122	-4.991981559586	-3.104806505388
H	6.383724108570	-6.344008618407	-1.451241759733
H	4.782531794237	-3.320555985570	2.152321497204
H	6.071459047107	-5.336313345765	2.709846695230
C	4.927822908841	-4.289341192448	-2.355232827785
C	4.115542256418	-2.481389042406	-0.359386300605
H	-0.739099015021	9.310044991362	-0.415975550752
H	4.925902380334	4.959706061699	3.877828919149
H	7.472824233488	1.083362538700	3.541876076872
H	6.915433771229	-6.831260080552	0.909481280139
C	-2.924834607591	2.601972623438	-0.901184677645
C	-2.888555546248	1.225898311182	-0.891442901078
S	-4.593770810481	3.183446462227	-1.128330652910
C	-4.162822430403	0.629117500602	-1.058204343266
H	-1.990916345454	0.649352602983	-0.726888307091
C	-5.175786274667	1.565595668726	-1.185080490752
C	-4.669765980010	-0.712739862909	-1.069311652124
C	-6.079916819997	-0.731089636844	-1.166496673935
S	-6.773734144813	0.891363968257	-1.295935737965
C	-3.981911740855	-1.926776648241	-0.957981122475
H	-2.900153133014	-1.945388636675	-0.865378986087

C	-4.691839971505	-3.111757129811	-0.918135033673
H	-4.157961052581	-4.051644366112	-0.808720876039
C	-6.803529288410	-1.915039244755	-1.118904767782
H	-7.886486797655	-1.918150868988	-1.169851072652
C	-6.093951157209	-3.102596996189	-0.990445760547
C	-6.819812513566	-4.410991925403	-0.907731944157
F	-6.335581724715	-5.309201257100	-1.805965959480
F	-8.143989496544	-4.288982314123	-1.144100836280
F	-6.682873070649	-4.983716070263	0.315370752328
C	3.676364717331	-3.038774491223	-4.104343498170
C	2.911596148320	-2.056562400208	-4.690042683290
S	4.147573056521	-4.263648069795	-5.313804463301
C	2.677572764484	-2.269411632802	-6.069501909197
H	2.536858222487	-1.191625744821	-4.166244377396
C	3.297601795766	-3.411696596419	-6.544593440505
C	1.937962329309	-1.592689208271	-7.093586940182
S	3.062547330065	-3.704507112002	-8.241846684123
C	2.058494633678	-2.249426382939	-8.338340236828
C	1.172564424359	-0.425650201368	-7.011957351366
C	1.438774537014	-1.768284559645	-9.482578303497
H	1.101930518770	0.118617007147	-6.075211032193
C	0.542129706489	0.060217474437	-8.142513874520
H	1.545609159701	-2.271065328615	-10.437767280465
C	0.674444953373	-0.611864839487	-9.368050648981
H	-0.042304867463	0.973921609498	-8.078724162127
C	-0.094494571909	-0.111020736667	-10.551854712578
F	-1.407487495929	-0.488646091100	-10.480492945153
F	0.378413054177	-0.591641173312	-11.722587174809
F	-0.091173329964	1.236815708035	-10.633825580128
C	0.632144441438	0.241981105553	1.971579969748
C	-0.482996954718	-0.553814344093	1.880681258927
S	0.213402420318	1.899397400003	2.442833163515
C	-1.681886987069	0.141421609969	2.194059598712

H	-0.443836819113	-1.598069611257	1.595262283996
C	-1.453629909734	1.480442128432	2.481318932019
C	-3.079427096589	-0.185737872770	2.277069259310
S	-2.886517913152	2.415181482515	2.788106135248
C	-3.863051561506	0.958683061947	2.563091793228
C	-3.723210441826	-1.420167293994	2.137329345938
C	-5.245664220713	0.900043606061	2.655978234020
H	-3.141312810534	-2.311818188762	1.923246991741
C	-5.100204149235	-1.494728079900	2.252971649656
H	-5.837262920914	1.787837233100	2.851870594090
C	-5.855866413903	-0.338637189860	2.490827640648
H	-5.602919530292	-2.448855204559	2.129210033876
C	-7.344531119843	-0.452866032148	2.633489836127
F	-7.873286160177	-1.341431392463	1.756135637967
F	-7.972176783968	0.728888780437	2.431987416856
F	-7.702083637915	-0.879147279694	3.872629495839
C	4.436230755302	1.613228066297	-2.334337493239
C	4.135550405436	1.616021701471	-3.673372261289
S	5.749680756835	0.477702351534	-1.965664510854
C	4.937492459382	0.715891128484	-4.419581183039
H	3.363393631308	2.235906965806	-4.110258769450
C	5.835858055804	0.021164212080	-3.622941336170
C	5.024497746375	0.316447286023	-5.795877228988
S	6.793482071049	-1.157761213035	-4.469324890756
C	5.980744577521	-0.712013790479	-5.976483792015
C	4.316640272274	0.783645948869	-6.908040551589
C	6.214561171656	-1.287063178709	-7.216473433906
H	3.571511129637	1.564874558124	-6.787337674773
C	4.551546894175	0.224114107244	-8.151680718619
H	6.936648861773	-2.086810701544	-7.342240775105
C	5.484468248404	-0.811141367635	-8.301065534517
H	3.994569112359	0.573320675848	-9.015435357288
C	5.747366649813	-1.369410426583	-9.667762510672

F	4.622416892338	-1.421198392403	-10.423441050114
F	6.258764834456	-2.622040005288	-9.623214742269
F	6.637607148709	-0.610997621439	-10.359845829399
C	1.057353737328	-4.859307737824	-0.526811190286
C	1.235197858180	-4.906416465910	0.855460672078
C	1.837325630734	-5.709143793927	-1.313362502179
C	2.125285173354	-5.805945692286	1.433315243415
C	2.719804068966	-6.616197389358	-0.747089705776
C	2.851726278466	-6.676792082292	0.634393855004
F	0.565910660015	-4.109172027743	1.688914531541
F	2.278257423732	-5.841601516513	2.758969483443
F	3.676855683570	-7.560800291875	1.189483102788
F	3.444986215883	-7.426955014730	-1.521889734875
F	1.759163622793	-5.676468985455	-2.645295042787
C	-0.093816416747	4.382269669602	-4.262702423276
C	1.048320958654	5.181237063530	-4.209913915308
C	-1.325172662552	4.987473072271	-4.006114479771
C	0.955447753031	6.547887312707	-3.967238311842
F	2.273734008279	4.689538360889	-4.393940140319
C	-1.428643101450	6.349060165929	-3.766574397366
F	-2.450416679536	4.270210322200	-3.987942219885
C	-0.284583998199	7.136651646937	-3.768378076766
F	2.057622429673	7.300037668325	-3.933654860453
F	-2.619791090776	6.905611761690	-3.532661178766
F	-0.375147708152	8.449366972503	-3.571012822596
C	-1.092536706502	-3.602686925740	-8.197438143421
C	-0.287454257874	-4.547553736365	-7.556347907921
C	-0.072052805706	-4.466252406971	-6.183328532807
C	-0.683626466597	-3.469240600449	-5.442491935596
C	-1.515215141273	-2.515569422632	-6.070374630391
C	-1.693179557444	-2.594848968021	-7.469866245285
H	-1.252031642303	-3.658096787911	-9.269871188148
H	0.174083389648	-5.344322500293	-8.132036365808

H	0.570801581796	-5.182052087957	-5.680084327823
H	-0.496539856113	-3.432440936374	-4.375921246041
H	-2.327431549473	-1.885528689412	-7.988395689707
C	-2.180949881507	-1.503741247553	-5.278321279099
C	-2.037669999818	-1.517864075283	-3.808283906196
H	-2.034902908502	-2.522791477293	-3.383831539498
H	-2.795370420509	-0.904605438997	-3.322229030594
C	-2.699376623381	-0.260257449267	-5.894225231343
H	-3.071904802309	-0.442603275220	-6.906893202866
H	-1.794531249315	0.366160123420	-6.034258024250
C	-3.703771594809	0.561806154489	-5.081264511372
H	-4.442990413594	-0.110424968353	-4.617955516465
H	-3.183499147678	1.083967935044	-4.268925909981
C	-4.430216560384	1.587422147889	-5.947487712791
H	-4.962587474294	1.068894227873	-6.758793277017
H	-3.690375535342	2.245265814626	-6.419603191171
C	-5.416916847816	2.416426673230	-5.130219475115
H	-5.942988617800	3.142666787362	-5.757631888111
H	-6.168092074352	1.776661908678	-4.650388823759
H	-4.896588689626	2.968351592276	-4.340063822619
Si	-4.844589535390	-3.468255086449	-5.322398549048
H	-3.911948415859	-2.266429349104	-5.274368734311
C	-4.955537545247	-3.964561700849	-7.128960448264
H	-5.709289887563	-4.751793831846	-7.254059620347
H	-5.243351222629	-3.124651503597	-7.769822244167
H	-3.999591013516	-4.357527252114	-7.489562186402
C	-6.481208485418	-2.830332541136	-4.657109434540
H	-6.291403253657	-2.412325735312	-3.660826796710
H	-7.158109019755	-3.682529764834	-4.500150570453
C	-7.140824329478	-1.775208629635	-5.555896553537
H	-8.055279496854	-1.379512906411	-5.101273913379
H	-6.470773533730	-0.925264269519	-5.732685478912
H	-7.411015433757	-2.189911043431	-6.532671944765

C	-4.035393370690	-4.794544516339	-4.287276082970
H	-4.648061024541	-5.703453256749	-4.274295468417
H	-3.049596207364	-5.057829073811	-4.686824299179
H	-3.897726847867	-4.473080221617	-3.251015673333

TS2’

O	1.597418918773	2.785303119706	-2.344903596052
O	-0.146677815265	2.325704906981	-0.549978609328
O	1.625099192050	-1.505117914556	0.511438860832
O	2.881726415244	-1.436319213042	-1.680891348474
S	-0.550708282806	-3.218481804050	-0.891085584088
S	-0.365514320981	2.007260951659	-4.530951799274
O	-0.944748348572	-2.889650841674	0.487266926825
O	-1.629168520744	-3.642618010640	-1.803534898265
O	0.968143144422	1.733433363210	-5.102102792713
O	-1.528442917500	1.572693658181	-5.320135997365
N	0.272872771107	-2.058719004619	-1.644536459485
N	-0.544148180258	1.460859573432	-3.025021150182
P	1.413730639003	-1.112990633442	-1.046416139991
N	1.300878782010	0.425198131765	-1.264135038256
P	0.538345821727	1.648553281642	-1.866061618643
H	-0.887595237160	-1.762667795428	-3.518332757555
C	-0.510998540906	3.661693991982	-0.591218112952
C	2.441753758332	3.277978928410	-1.355425304983
C	2.697805567322	-0.886137708605	1.145532987660
C	3.592416062254	-2.547673273759	-1.269022002588
C	-1.899044671209	3.989218847973	-0.545077341073
C	3.815476095499	-3.583355597758	-2.223940169709
C	3.779443361265	2.800530372017	-1.310997996936
C	2.424480137562	0.224919811873	1.989464207371
C	0.724012434630	8.310229891981	-1.105259507091
C	1.109516654836	6.998440759974	-0.963560962262
C	0.148706977281	5.984552181474	-0.739743202285

C	-1.233104396944	6.343612427413	-0.700633281538
C	-1.594001267732	7.708627906811	-0.825820928740
C	-0.636503869253	8.671673845995	-1.018516963398
H	1.472682448057	9.073700743670	-1.293893103287
H	2.157756489798	6.727148558466	-1.037040675122
C	0.497099824256	4.609082033064	-0.599875213536
C	-2.210444717452	5.340184067709	-0.573802245740
H	-2.647029560821	7.975550081686	-0.790618302759
H	-3.248615084692	5.659306875510	-0.541563836474
C	4.414433270190	5.432259539243	2.733673617746
C	4.903812014006	4.597170475933	1.761015895445
C	4.081037910752	4.168218139001	0.690013533828
C	2.733831728478	4.641493230952	0.612653401800
C	2.256177454868	5.487170096574	1.641480841180
C	3.075481973157	5.871093444577	2.676015526893
H	5.609784767781	2.955173611803	-0.212086849784
H	5.930161157137	4.240919483589	1.798590600027
C	4.573590931148	3.277146622148	-0.284266839751
C	1.913729959950	4.186399439862	-0.466091002910
H	1.225517099146	5.824072075860	1.615909731254
H	2.686438674418	6.515156202718	3.459033250786
C	7.467167554768	-0.346388887115	1.711825408171
C	6.419294140630	-1.063463168697	1.185674961882
C	5.079827209148	-0.702048177333	1.461591266659
C	4.838591807625	0.446595781007	2.279053738664
C	5.941752924336	1.154258220823	2.817851722822
C	7.229426527587	0.767650386451	2.542738006162
H	8.487413284330	-0.637306656769	1.480131933699
H	6.617203236142	-1.910125085103	0.537051091576
C	3.956086706228	-1.384908022226	0.899513805366
C	3.518153002377	0.865392464328	2.540892738979
H	5.743840643102	2.017292429958	3.448606384633
H	3.368790759713	1.718612432717	3.198629924753

C	6.074973604947	-6.107036452703	1.279614204242
C	5.685422723377	-5.967798678323	-0.028374452996
C	5.012476099843	-4.798355110536	-0.462508462009
C	4.774061119223	-3.744429750886	0.471401281437
C	5.167452393763	-3.930165017835	1.817559348662
C	5.800337110290	-5.084722805286	2.210882819500
H	4.783449821755	-5.485895938612	-2.470385420726
H	5.869831826610	-6.761821622917	-0.747504849141
H	4.951055570018	-3.153712693879	2.543521948993
H	6.082107761210	-5.215254567760	3.251337357781
C	4.553247200440	-4.672518601285	-1.786592920799
C	4.111840858223	-2.565752638348	0.014037827133
H	-0.925585822839	9.712218456186	-1.130991510393
H	5.052931891566	5.752315761779	3.551867286426
H	8.067000044272	1.319692597902	2.958990955734
H	6.577990400183	-7.012592575555	1.604917062905
C	-2.946025990786	2.977660253372	-0.463901656278
C	-2.893610429812	1.604648339143	-0.481138750648
S	-4.637516239821	3.536486827122	-0.336330170013
C	-4.168969419968	0.992764978858	-0.417240458704
H	-1.976902437101	1.040548132167	-0.536914925449
C	-5.200300912949	1.911581704224	-0.331592900847
C	-4.654972192501	-0.352379643265	-0.456655647833
C	-6.065973682912	-0.399289047422	-0.392960700521
S	-6.789641098678	1.210768799541	-0.283942700577
C	-3.936623665014	-1.547066141818	-0.548623113787
H	-2.852636179361	-1.529717242428	-0.567114903248
C	-4.614018134978	-2.750564793552	-0.590984447570
H	-4.050149847128	-3.676312492057	-0.660220154488
C	-6.759618679216	-1.602218241160	-0.432833904086
H	-7.842424596537	-1.632907072705	-0.385533426458
C	-6.017593481895	-2.773124356927	-0.542821652242
C	-6.698971923515	-4.104741547141	-0.618059673086

F	-8.044616843745	-4.005742483398	-0.641353321806
F	-6.372881349740	-4.901554524412	0.429521750012
F	-6.331414179486	-4.786972787832	-1.740879546898
C	3.314505553457	-3.512674800928	-3.594846380148
C	2.664354305312	-2.521268299974	-4.295099487035
S	3.598553196268	-4.931405465398	-4.639146638410
C	2.386603277315	-2.872489417807	-5.643508820695
H	2.417338528532	-1.556932130346	-3.876711655405
C	2.838164638585	-4.143351566519	-5.961520364659
C	1.793976954148	-2.222664417754	-6.780539087049
S	2.600791290229	-4.610050332665	-7.618758687395
C	1.853990615186	-3.037989195149	-7.934655250128
C	1.225287059588	-0.948217399457	-6.887075916173
C	1.410318200989	-2.595706203537	-9.173627538645
H	1.170077824734	-0.292198836841	-6.024224804044
C	0.770017278097	-0.500986679885	-8.113188553016
H	1.492084322574	-3.218714535338	-10.057532248951
C	0.873834433604	-1.316179945347	-9.251128630189
H	0.348418621672	0.496643698426	-8.192291738066
C	0.450259352664	-0.764840715003	-10.579309506651
F	1.367161598702	0.104573761895	-11.074831333017
F	-0.718862685635	-0.080752734607	-10.492934207723
F	0.278883224089	-1.730202430628	-11.510618147522
C	1.056947332787	0.658589353502	2.240240710691
C	-0.106133645067	-0.071612556210	2.254073145368
S	0.757540286984	2.366341894743	2.615502257077
C	-1.243956136383	0.712677061639	2.569841953629
H	-0.144464819307	-1.133941474620	2.048845629296
C	-0.926966535634	2.048325923207	2.772915295229
C	-2.647452394758	0.464128436453	2.741672699113
S	-2.288219430450	3.066677244232	3.139250018004
C	-3.350532083993	1.654239999428	3.048584245399
C	-3.358514136443	-0.737342642045	2.656754105722

C	-4.722399132585	1.664388334545	3.252399194561
H	-2.839354705933	-1.657758193277	2.404954798153
C	-4.725623009640	-0.739472294851	2.870285094695
H	-5.254488713337	2.582880499119	3.476011014998
C	-5.402005470683	0.453788258976	3.158782334597
H	-5.284081667531	-1.667113932815	2.793857893644
C	-6.873128463751	0.409838305149	3.445641876634
F	-7.517988487692	-0.502733722807	2.677635557190
F	-7.474978229277	1.604819022684	3.241874123279
F	-7.122852994197	0.065723183608	4.737087686959
C	4.275118547570	1.823562678648	-2.273840688890
C	3.958757340509	1.626827631694	-3.594787000864
S	5.485008972901	0.656632274908	-1.711505541462
C	4.684535700128	0.554050390865	-4.179291162062
H	3.226413377420	2.222959465849	-4.124194510621
C	5.525866668087	-0.070163483433	-3.268048492206
C	4.775875409707	-0.048694504571	-5.481214759250
S	6.407784304693	-1.428768803756	-3.901095213047
C	5.662343883555	-1.154514058396	-5.480521008619
C	4.155065534250	0.310093081273	-6.682478690277
C	5.900590996034	-1.907976386550	-6.619572615436
H	3.467474567870	1.150520099591	-6.704163709979
C	4.404588134795	-0.421223013522	-7.831197582927
H	6.568740626463	-2.762563184348	-6.601908186537
C	5.259625861645	-1.530466418898	-7.795753088498
H	3.914412176176	-0.150590154278	-8.761227690038
C	5.554641581742	-2.276214296814	-9.062453826942
F	4.481593155024	-2.326749196114	-9.889268598844
F	5.940851691024	-3.553007336323	-8.828913276579
F	6.556350608490	-1.690751183004	-9.769677112469
C	0.591035854494	-4.639456045195	-0.785050468301
C	1.252192416462	-4.985347943272	0.395751175963
C	0.800846421742	-5.441648418718	-1.908698153136

C	2.061675191072	-6.114691157286	0.460715170989
C	1.591386288553	-6.579394376658	-1.848631389862
C	2.224536624778	-6.915499287455	-0.659651673610
F	1.147960792069	-4.262478624713	1.507137472647
F	2.689877263247	-6.425063192513	1.593916161068
F	3.010272846415	-7.989609169571	-0.606840949197
F	1.763134705376	-7.346582764164	-2.929286697529
F	0.257080508916	-5.139760354649	-3.088227791608
C	-0.508038994096	3.824095027081	-4.391601777330
C	0.548702138955	4.701941932979	-4.635403625564
C	-1.737632399281	4.371748481716	-4.021027148003
C	0.374659710187	6.078451667353	-4.537198604356
F	1.767271392455	4.281515227693	-4.974405923314
C	-1.922924292801	5.743185411529	-3.927061421043
F	-2.777973623210	3.589215686109	-3.733565791448
C	-0.864667237814	6.600903841179	-4.197824805411
F	1.396208469565	6.903290769760	-4.778606739722
F	-3.109940278598	6.243139393711	-3.574053765883
F	-1.038284388249	7.918218612365	-4.134769162274
C	-2.854725733722	-6.391039297956	-4.514716333465
C	-1.815849117238	-6.842160345262	-5.327503793653
C	-0.957670873673	-5.930354566690	-5.939680488614
C	-1.121368422419	-4.574250244445	-5.721311897690
C	-2.169367774605	-4.101566442744	-4.907308745559
C	-3.045771894604	-5.034153686516	-4.319390178272
H	-3.514282995285	-7.102032752878	-4.027032015932
H	-1.673987415333	-7.907841515085	-5.482738304496
H	-0.152650932071	-6.276462108759	-6.580489671748
H	-0.456547821124	-3.874813337987	-6.218448682827
H	-3.838602489976	-4.698427461069	-3.659783079625
C	-2.325762570001	-2.682764096375	-4.649963649989
C	-1.148857627466	-1.816590012113	-4.594757231970
H	-1.354843951643	-0.790902997078	-4.909411500872

H	-0.275706176169	-2.226935024859	-5.096155207103
C	-3.601433119032	-2.160533760079	-4.100604105501
H	-3.583899835502	-2.466850562133	-3.036409761154
H	-4.449007013807	-2.694894431839	-4.546195158941
C	-3.800176319934	-0.646312462039	-4.173354706969
H	-3.161120253635	-0.143266428434	-3.435795915447
H	-3.469587478461	-0.273242958563	-5.151598271512
C	-5.255893539591	-0.243596936177	-3.956965014576
H	-5.618588961041	-0.653551544336	-3.006258745990
H	-5.877363765309	-0.696277087499	-4.743386831520
C	-5.425015577441	1.273551321676	-3.971784330526
H	-6.468597407637	1.561472851873	-3.811224980996
H	-4.820109720556	1.738029104971	-3.185849302657
H	-5.094584901496	1.695681469598	-4.927726338774
Si	-3.380931944751	-1.985048186893	-7.788162484664
H	-2.779773737949	-2.161329094035	-6.406772380589
C	-5.176324901830	-2.513932258494	-7.643798671325
H	-5.265299501960	-3.546628908210	-7.288569982356
H	-5.727992862680	-1.865195108601	-6.955093124598
H	-5.674363426617	-2.454202437686	-8.618778981955
C	-2.403040428282	-3.146736796981	-8.896937995710
H	-2.546540221038	-4.172185341148	-8.527961707766
H	-1.333733835799	-2.928071908807	-8.775920460384
C	-2.794148799591	-3.053377969900	-10.380823977251
H	-2.207701852529	-3.750007649417	-10.989153441353
H	-3.852212902225	-3.293019016797	-10.534439188389
H	-2.616448103624	-2.048463015993	-10.776274412350
C	-3.219908476593	-0.176598193595	-8.239129831095
H	-3.857241400283	0.443039239723	-7.599549188873
H	-2.190735535795	0.172666716721	-8.117323533503
H	-3.515248899017	-0.006238748542	-9.280822272488

13. References

(1) He, T.; Qu, Z. W.; Klare, H. F. T.; Grimme, S.; Oestreich, M. Intermolecular Carbosilylation of α-Olefins with C(Sp3)─C(Sp) Bond Formation Involving Silylium-Ion Regeneration. *Angew. Chem., Int. Ed.* **2022**, *61*.

(2) Pyne, S. G. Asymmetric Conjugate Addition of Organometallic Reagents to Chiral Vinyl Sulfoximines. *J. Org. Chem.* **1986**, *51*, 81–87.

(3) Lu, P.; Ren, X.; Xu, H.; Lu, D.; Sun, Y.; Lu, Z. Iron-Catalyzed Highly Enantioselective Hydrogenation of Alkenes. *J. Am. Chem. Soc.* **2021**, *143*, 12433–12438.

(4) Goering, H. L.; Tseng, C. C. Alkylation of Allylic Derivatives. 9. On the Stereochemistry of Alkylation of Acyclic Allylic Alcohols by the Murahashi Method. *J. Org. Chem.* **1985**, *50*, 1597–1599.

(5) Friedfeld, M. R.; Shevlin, M.; Margulieux, G. W.; Campeau, L. C.; Chirik, P. J. Cobalt-Catalyzed Enantioselective Hydrogenation of Minimally Functionalized Alkenes: Isotopic Labeling Provides Insight into the Origin of Stereoselectivity and Alkene Insertion Preferences. *J. Am. Chem. Soc.* **2016**, *138*, 3314–3324.

(6) Magrez, M.; Le Guen, Y.; Baslé, O.; Crévisy, C.; Mauduit, M. Bidentate Hydroxyalkyl NHC Ligands for the Copper-Catalyzed Asymmetric Allylic Substitution of Allyl Phosphates with Grignard Reagents. *Chem. - A Eur. J.* **2013**, *19*, 1199–1203.

(7) Tang, X.; Qian, L.; Liu, G.; Huang, Z. Iridium-Catalyzed Asymmetric Transfer Hydrogenation of 1-Aryl-1-Alkylethenes with Ethanol. *Org. Lett.* **2023**, *25*, 4950–4954.

(8) Rafiq, S. M.; Sivasakthikumaran, R.; Mohanakrishnan, A. K. Lewis Acid/Brönsted Acid Mediated Benz-Annulation of Thiophenes and Electron-Rich Arenes. *Org. Lett.* **2014**, *16*, 2720–2723.

(9) Maji, R.; Ghosh, S.; Grossmann, O.; Zhang, P.; Leutzsch, M.; Tsuji, N.; List, B. A Catalytic Asymmetric Hydrolactonization. *J. Am. Chem. Soc.* **2023**, *145*, 8788–8793.

(10) Gatzenmeier, T.; Turberg, M.; Yepes, D.; Xie, Y.; Neese, F.; Bistoni, G.; List, B. Scalable and Highly Diastereo- and Enantioselective Catalytic Diels-Alder Reaction of α,β-Unsaturated Methyl Esters. *J. Am. Chem. Soc.* **2018**, *140*, 12671–12676.

(11) Scharf, M. J. Catalytic Asymmetric Pictet-Spengler Reactions toward Tetrahydroisoquinolines. Doctoral Dissertation, University of Cologne, Cologne, 2023.

(12) Gutsulyak, D. V.; Vyboishchikov, S. F.; Nikonov, G. I. Cationic Silane δ-Complexes of Ruthenium with Relevance to Catalysis. *J. Am. Chem. Soc.* **2010**, *132*, 5950–5951.

(13) Klussmann, M.; Iwamura, H.; Mathew, S. P.; Wells, D. H.; Pandya, U.; Armstrong, A.; Blackmond, D. G. Thermodynamic Control of Asymmetric Amplification in Amino Acid Catalysis. *Nature* **2006**, *441*, 621–623.

(14) Hayashi, Y.; Matsuzawa, M.; Yamaguchi, J.; Yonehara, S.; Matsumoto, Y.; Shoji, M.; Hashizume, D.; Koshino, H. Large Nonlinear Effect Observed in the Enantiomeric Excess of Proline in Solution and That in the Solid State. *Angew. Chem.* **2006**, *118*, 4709–4713.

(15) Geiger, Y.; Bellemin-Laponnaz, S. Non-Linear Effects in Asymmetric Catalysis: Impact of Catalyst Precipitation. *ChemCatChem* **2022**, *14*.

(16) Maeda, S.; Harabuchi, Y.; Takagi, M.; Taketsugu, T.; Morokuma, K. Artificial Force Induced Reaction (AFIR) Method for Exploring Quantum Chemical Potential Energy Surfaces. *Chem. Rec.* **2016**, *16*, 2232–2248.

(17) Maeda, S.; Ohno, K.; Morokuma, K. Systematic Exploration of the Mechanism of Chemical Reactions: The Global Reaction Route Mapping (GRRM) Strategy Using the ADDF and AFIR Methods. *Phys. Chem. Chem. Phys.* **2013**, *15*, 3683–3701.

(18) Bannwarth, C.; Ehlert, S.; Grimme, S. GFN2-XTB - An Accurate and Broadly Parametrized Self-Consistent Tight-Binding Quantum Chemical Method with Multipole Electrostatics and Density-Dependent Dispersion Contributions. *J. Chem. Theory Comput.* **2019**, *15*, 1652–1671.

(19) Neese, F. The ORCA Program System. *Wiley Interdiscip. Rev. Comput. Mol. Sci.* **2012**, *2*, 73–78.

(20) Grimme, S.; Hansen, A.; Ehlert, S.; Mewes, J. M. R2SCAN-3c: A "Swiss Army Knife" Composite Electronic-Structure Method. *J. Chem. Phys.* **2021**, *154*, 64103.

(21) Neese, F. Software Update: The ORCA Program System—Version 5.0. *Wiley Interdiscip. Rev. Comput. Mol. Sci.* John Wiley & Sons, Ltd September 1, 2022, p e1606.

(22) Neese, F. Software Update: The ORCA Program System—Version 6.0. *Wiley Interdiscip. Rev. Comput. Mol. Sci.* John Wiley & Sons, Ltd March 1, 2025, p e70019.

(23) Mardirossian, N.; Head-Gordon, M. Ωb97X-V: A 10-Parameter, Range-Separated Hybrid, Generalized Gradient Approximation Density Functional with Nonlocal Correlation, Designed by a Survival-of-the-Fittest Strategy. *Phys. Chem. Chem. Phys.* **2014**, *16*, 9904–9924.

(24) Weigend, F.; Ahlrichs, R. Balanced Basis Sets of Split Valence, Triple Zeta Valence and Quadruple Zeta Valence Quality for H to Rn: Design and Assessment of Accuracy. *Phys. Chem. Chem. Phys.* **2005**, *7*, 3297–3305.

(25) Lu, T.; Chen, Q. Independent Gradient Model Based on Hirshfeld Partition: A New Method for Visual Study of Interactions in Chemical Systems. *J. Comput. Chem.* **2022**, *43*, 539–555.

(26) Lu, T.; Chen, F. Multiwfn: A Multifunctional Wavefunction Analyzer. *J. Comput. Chem.* **2012**, *33*, 580–592.

(27) Frisch, M. J.; Trucks, G. W.; Schlegel, H. B.; Scuseria, G. E.; Robb, M. A.; Cheeseman, J. R.; Scalmani, G.; Barone, V.; Petersson, G. A.; Nakatsuji, H.; Li, X.; Caricato, M.; Marenich, A. V; Bloino, J.; Janesko, B. G.; Gomperts, R.; Mennucci, B.; Hratchian, H. P.; Ortiz, J. V; Izmaylov, A. F.; Sonnenberg, J. L.; Williams-Young, D.; Ding, F.; Lipparini, F.; Egidi, F.; Goings, J.; Peng, B.; Petrone, A.; Henderson, T.; Ranasinghe, D.; Zakrzewski, V. G.; Gao, J.; Rega, N.; Zheng, G.; Liang, W.; Hada, M.; Ehara, M.; Toyota, K.; Fukuda, R.; Hasegawa, J.; Ishida, M.; Nakajima, T.; Honda, Y.; Kitao, O.; Nakai, H.; Vreven, T.; Throssell, K.; Montgomery, J. A., J.; Peralta, J. E.; Ogliaro, F.; Bearpark, M. J.; Heyd, J. J.; Brothers, E. N.; Kudin, K. N.; Staroverov, V. N.; Keith, T. A.; Kobayashi, R.; Normand, J.; Raghavachari, K.; Rendell, A. P.; Burant, J. C.; Iyengar, S. S.; Tomasi, J.; Cossi, M.; Millam, J. M.; Klene, M.; Adamo, C.; Cammi, R.; Ochterski, J. W.; Martin, R. L.; Morokuma, K.; Farkas, O.; Foresman, J. B.; Fox, D. J. Gaussian 16, Revision A.03. Gaussian, Inc., Wallingford CT 2016.

(28) Meng, E. C.; Goddard, T. D.; Pettersen, E. F.; Couch, G. S.; Pearson, Z. J.; Morris, J. H.; Ferrin, T. E. UCSF ChimeraX: Tools for Structure Building and Analysis. *Protein Sci.* **2023**, *32*, e4792.

(29) Blender Online Community, "Blender - a 3D modelling and rendering package" (Stichting Blender Foundation, 2018) https://www.blender.org.

(30) Peng, Q.; Duarte, F.; Paton, R. S. Computing Organic Stereoselectivity-from Concepts to Quantitative Calculations and Predictions. *Chem. Soc. Rev.* **2016**, *45*, 6093–6107.

(31) Bickelhaupt, F. M.; Houk, K. N. Analyzing Reaction Rates with the Distortion/Interaction-Activation Strain Model. *Angew. Chemie Int. Ed.* **2017**, *56*, 10070–10086.
